# BARHL1 Is Downregulated in Alzheimer’s Disease and May Regulate Cognitive Functions through ESR1 and Multiple Pathways

**DOI:** 10.3390/genes8100245

**Published:** 2017-09-28

**Authors:** Debmalya Barh, María E. García-Solano, Sandeep Tiwari, Antaripa Bhattacharya, Neha Jain, Daniel Torres-Moreno, Belén Ferri, Artur Silva, Vasco Azevedo, Preetam Ghosh, Kenneth Blum, Pablo Conesa-Zamora, George Perry

**Affiliations:** 1 Centre for Genomics and Applied Gene Technology, Institute of Integrative Omics and Applied Biotechnology, Nonakuri, Purba Medinipur, West Bengal 721172, India; sandip_sbtbi@yahoo.com (S.T.); antaripa1210@gmail.com (A.B.); mymailtoneha@gmail.com (N.J.); 2 Instituto de Ciências Biológicas, Universidade Federal de Minas Gerais, Belo Horizonte, MG 31270-901, Brazil; vascoariston@gmail.com; 3 Department of Pathology, Santa Lucía General University Hospital (HGUSL), C/Mezquita s/n, 30202 Cartagena, Spain; garciasolano2002@yahoo.com (M.E.G.-S.); daniel-torres@orange.es (D.T.-M.); pablo.conesa@carm.es (P.C.-Z.); 4 Catholic University of Murcia (UCAM), 30107 Murcia, Spain; 5 Department of Pathology, Virgen Arrixaca University Hospital (HUVA), Ctra. Madrid Cartagena sn, 30120 El Palmar, Spain; belen.ferri@carm.es; 6 Instituto de Ciências Biológicas, Universidade Federal do Pará, Rua Augusto Corrêa, 01-Guamá, Belém, PA 66075-110, Brazil; arturluizdasilva@gmail.com; 7 Department of Computer Science, Virginia Commonwealth University, Richmond, VA 23284, USA; pghosh@vcu.edu; 8 Department of Psychiatry & McKnight Brain Institute, University of Florida College of Medicine, Gainesville, FL 32610, USA; drd2gene@gmail.com; 9 UTSA Neurosciences Institute and Department of Biology, University of Texas at San Antonio, San Antonio, TX 78249, USA; george.perry@case.edu; 10 Department of Pathology, School of Medicine, Case Western Reserve University, Cleveland, OH 44106, USA

**Keywords:** Alzheimer’s disease, bioinformatics, estrogen, microRNA, signaling

## Abstract

The Transcription factor BarH like homeobox 1 (BARHL1) is overexpressed in medulloblastoma and plays a role in neurogenesis. However, much about the BARHL1 regulatory networks and their functions in neurodegenerative and neoplastic disorders is not yet known. In this study, using a tissue microarray (TMA), we report for the first time that BARHL1 is downregulated in hormone-negative breast cancers and Alzheimer’s disease (AD). Furthermore, using an integrative bioinformatics approach and mining knockout mouse data, we show that: (i) BARHL1 and Estrogen Receptor 1 (ESR1) may constitute a network that regulates Neurotrophin 3 (NTF3)- and Brain Derived Neurotrophic Factor (BDNF)-mediated neurogenesis and neural survival; (ii) this is probably linked to AD pathways affecting aberrant post-translational modifications including SUMOylation and ubiquitination; (iii) the BARHL1-ESR1 network possibly regulates β-amyloid metabolism and memory; and (iv) hsa-mir-18a, having common key targets in the BARHL1-ESR1 network and AD pathway, may modulate neuron death, reduce β-amyloid processing and might also be involved in hearing and cognitive decline associated with AD. We have also hypothesized why estrogen replacement therapy improves AD condition. In addition, we have provided a feasible new mechanism to explain the abnormal function of mossy fibers and cerebellar granule cells related to memory and cognitive decline in AD apart from the Tau and amyloid pathogenesis through our BARHL1-ESR1 axis.

## 1. Introduction

Alzheimer’s disease (AD) is one of the “most common forms of neurodegenerative dementia” [1]. The global prevalence of AD is as high as 24 million, and in the USA, alone it is approximately 5.4 million including a younger-onset AD population of approximately 200,000 [2,3]. According to the Alzheimer’s Association, every 68 s, someone develops AD in the USA, which is projected to be one new case of AD every 33 s by 2050 [3]. Although, the deaths due to cardiovascular diseases and cancer have decreased to a certain extent, deaths from AD have increased significantly (66% in the USA) [3].

Although several molecular markers and targets have been implemented for pathogenesis, diagnosis and therapy for this highly heterogeneous condition, the challenges remain unsolved, and the search for new molecular mechanisms is still ongoing [4]. In AD, cerebellar granule cells are decreased in number [5,6]; cerebellar volume is reduced [7]; spatial memory is disrupted [8,9,10]; and loss of hearing [11] is observed. Development and maintenance of the nervous system requires coordinated actions of multiple transcription factors that may be affected in neurodegenerative disorders like AD [12]. Reports suggest that the homeodomain transcription factor BARHL1 plays an essential role in the migration and survival of cerebellar granule cells and precerebellar neurons [13], and its expression is upregulated during cerebellar development in humans [14]. Apart from the importance of BARHL1 in brain homeostasis, a role of this transcription factor in brain tumor development has been reported. Pöschl et al. [14] also found that the expression of BARHL1 was significantly upregulated in medulloblastoma samples, and its low expression was associated with a less favorable prognosis. This study also shows that the *BARHL1* knockout mice displayed increased mitotic activity and decreased neuronal differentiation.

Despite the important role of BARHL1 in brain development, no studies so far have assessed *BARHL1* expression in neurodegenerative disorders like AD or Parkinson’s disease (PD) or in neoplastic diseases other than brain tumors.

Previous reports in mice have demonstrated that *BARHL1* expression is influenced by thyroid hormone [15]. Therefore, it would not be surprising that *BARHL1* might be regulated by the estrogen hormone. In fact, the depletion of estrogen hormones in post-menopausal women has been associated with increased risk of AD and cognitive decline [16,17,18], and estrogen replacement therapy (ERT) has been found to improve the AD condition [19,20,21]. Therefore, we aim to investigate: (a) whether *BARHL1* expression is associated with neurodegenerative diseases like AD, Parkinson’s disease (PD) or amyotrophic lateral sclerosis (ALS); (b) if there is any relationship between *BARHL1* expression and hormone-induced tumor of the breast; i.e., estrogen-positive breast carcinomas, HER2-positive and triple negative breast carcinomas being included as controls; and (c) an in-depth bioinformatics analysis to demonstrate a probable role of BARHL1 in AD patho-physiology and the molecules possibly associated with this regulation.

## 2. Materials and Methods

### 2.1. Tissue Samples

Neurodegenerative disease tissue samples comprising ten hippocampi affected by Alzheimer’s disease (AD) were collected from the Spanish National Brain Bank Network. Nerve tissue tumors were procured from Virgen de la Arrixaca University Hospital, Murcia, Spain, and breast cancer samples from the tissue bank of Santa Lucia University Hospital, Cartagena, Spain. Age- and sex-matched normal tissues were procured from necropsy and biopsy specimens that are not affected by the studied diseases. The study was approved by the hospital ethics committees, Santa Lucía General University Hospital (HGUSL), C/Mezquita s/n, 30202, Cartagena, Spain, Catholic University of Murcia (UCAM), Spain, and has been performed in accordance with the ethical standards laid down in the 1964 Declaration of Helsinki and its later amendments. Written informed consent was obtained from the participants. Table 1 describes the type and number of neurodegenerative and cancer samples evaluated in this study.

### 2.2. Tissue Microarray and Immunohistochemistry

A representative region of the tumors was selected for a tissue microarray (TMA), which was constructed as described by García-Solano et al. [22]. Immunostaining was evaluated independently by two pathologists. Inter-observer discordance (<15%) was resolved using a multi-headed microscope, and a consensus score was reached. TMA slides were pre-treated at 60 °C for 30 min, and the antigen retrieval was performed at 95 °C in a PT Link (Dako, Glostrup, Denmark) for 20 min using the EnVision FLEX target retrieval solution pH 6 buffer (Ref: K8005, Dako) according to the manufacturer´s instructions. Endogenous peroxidase activity was blocked using EnVision FLEX peroxidase-blocking reagent (Ref: SM801, Dako) for 5 min. Immunostaining was performed in a Dakocytomation machine (Dako). TMA sections were incubated 18 h at room temperature with polyclonal 1:500 diluted anti-BARHL1 (Ref: HPA004809, Sigma Aldrich, Taufkirchen, Germany) and were subsequently incubated with peroxidase-labelled polymer (EnVision FLEX/HRP, Ref: SM802, Dako) for 20 min at room temperature. For visualization of the antigen, the sections were immersed for ten min each in fresh 3,3′-diaminobenzidine (DAB) baths (EnVision FLEX DAB + chromogen, Dako) and counterstained with EnVision FLEX hematoxylin (Ref: SM806, Dako) for 7 min. Staining scores were calculated by considering the staining intensity score (A = no staining, B = weak, C = moderate, D = strong) in a given area and the stained area score (0 ≤ 5%, 1 = 5–50%, 2 = 50–75%, 3 ≥ 75%, 4 = 100%) as previously described by Conesa-Zamora et al. [23]. Staining was considered positive only when the staining was moderate or intense in more than 5% of the cells.

### 2.3. Statistical Analysis

Statistical analysis of BARHL1 expression in different tissues was performed using the Epidat computer program Version 3.1, Xunta de Galicia, A Coruña, Spain; Pan American Health Organization (PAHO), Washington DC, USA, 2005. Fisher´s exact test or Pearson´s χ^2^ were used to evaluate statistical significance as indicated.

### 2.4. Integrative Bioinformatics Approach

#### 2.4.1. BARHL1 Association with Alzheimer’s Disease

To elucidate the association and function of BARHL1 in AD, we took an integrative bioinformatics approach consisting of literature mining, pathway mapping, promoter binding prediction and protein-protein interaction (PPI) analysis. We also attempted to explore miRNA-mediated regulation of the BARHL1 circuit and AD pathway.

#### 2.4.2. Construction of the BARHL1 Regulatory Circuit

PubMed was extensively searched to collect literature describing experimentally-validated interactions, regulations and functions for BARHL1. Each article was manually scanned, and based on the manually-curated information, a BARHL1 regulatory circuit correlating with its reported function (neurogenesis, neuron migration, neuron survival) was made. Next, the proteins/genes involved in this interaction were used to expand the network and to identify additional proteins/genes directly or indirectly linked to this network. The protein-protein interaction (PPI) and visualization tool VisANT [24] were used for this purpose. Human, mouse and rat PPI databases were used. Both the validated and predicted interactions were considered. The network was expanded two times to get both the direct and indirect interactions. The Comparative Toxicogenomics Database (CTD) [25] was also used to find the regulators of BARHL1 circuit genes.

#### 2.4.3. Collection of Alzheimer’s Disease, Parkinson’s Disease and Amyotrophic Lateral Sclerosis Pathway-Specific Genes

AD, PD and ALS are all neurodegenerative diseases and share common molecular profiles. For this reason, we collected all AD (Pathway ID: hsa05010), PD (Pathway ID: hsa05012) and ALS (Pathway ID: hsa05014) pathway-specific genes from the Kyoto Encyclopedia of Genes and Genomes (KEGG) pathway database [26]. Next, we screened and grouped accordingly both the unique and common genes of these neurodegenerative disease pathways. Since our main aim is to understand BARHL1 function in AD, those unique genes from the AD pathway were finally selected for further analysis.

### 2.5. Promoter Analysis

We wanted to identify the regulation of BARHL1 circuit genes at the transcriptional level and also to investigate whether the BARHL1 circuit and AD pathway are linked. To find out if any BARHL1 circuit-associated transcription factors (TFs) bind to the promoter of any key gene of AD, thus regulating the pathway or vice versa, we used the MatInspector module (promoter analysis) [27] of the Genomatix suite (www.genomatix.de) following the user guidelines. Both the AD pathway-specific genes and the BARHL1 network genes were used in this analysis. Briefly, promoters of these genes were retrieved, and all of the TFs from each pathway (BARHL1 and AD) were identified using MatInspector. Using the same module, we next checked which of the TFs from these pathways have binding sites in the retrieved gene promoters and what the effect of the binding is.

### 2.6. Protein-Protein Interaction Analysis

In order to find the links between the BARHL1 regulatory circuit and the AD pathological pathway, PPIs were identified using BARHL1 circuit proteins (intra-PPIs), whereas inter-PPIs were detected from the key AD pathway and the BARHL1 circuit proteins with the help of the VisANT tool [24]. To select the key genes in the network based on the topological measures of gene priorities, each of these intra-PPI and inter-PPI maps was analyzed for centrality. The key nodes were selected based on the shortest pathways. Functional enrichment and annotation of the BARHL1 circuit proteins and the linker proteins of inter-PPI (BARHL1-AD pathways) were carried out using the ToppFun option of the ToppGene suite [28]. Further, extensive literature mining was done to correlate the functions of these genes in AD patho-physiology.

### 2.7. micro RNA Analysis

The micro RNAs (miRNAs) that are deregulated (up- and down-regulated) in AD were collected from PubMed indexed literature. miRNAs that target the BARHL1 circuit and key AD pathway genes were identified using the Validated Targets module of miRWalk [29]. In cases where the validated targets of miRNAs were not available, predicted targets of miRNAs were collected using the Predicted Targets option of miRWalk. The miRNAs having targets in common between the BARHL1 circuit and key AD pathways were listed, and the tool for annotations of human miRNAs (TAM) Vs.2 [30] was used to annotate the functions of these common miRNAs considering all as AD-associated miRNAs collected from PubMed as the background. *p*-values were used to quantify statistical significance, and ToppFun analysis of the ToppGene suit [28] was applied to annotate the function of miRNAs based on their targets as described in Barh et al. [31]. Finally, based on the literature survey, a correlation was made between the expression levels of the miRNAs and their corresponding targets in AD condition.

### 2.8. Mining the BARHL1 Knockout Mouse Phenotype for Alzheimer’s Disease Symptoms

To understand the BARHL1 functions in AD patho-physiology, we attempted to identify the AD symptoms in BARHL1 knockout (KO) mice from the published literature. We searched PubMed for literature describing the BARHL1KO mice phenotype and the literature on AD symptoms in patients. Then, we carefully mined the information for the KO phenotype-AD symptom associations.

## 3. Results and Discussion

### 3.1. Expression of BARHL1 in the Nervous System and Breast Tumors

**Figure 1:**
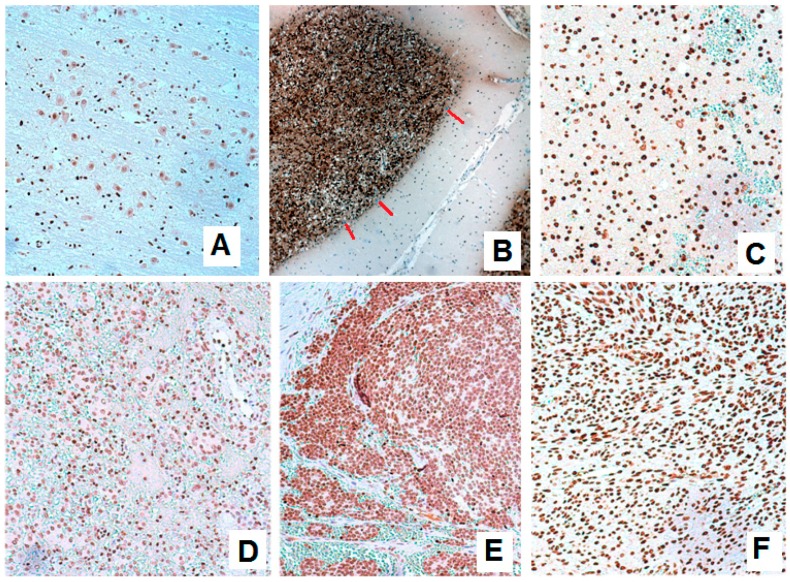
BarH like homeobox 1 (BARHL1) expression in normal and neoplastic specimens from the nervous system. (**A**) Staining in brain cortex. (**B**) Cerebellum showing a strong positive expression in granular layer cells (note: the absence of expression in Purkinje cells, indicated by red lines). Specimens from glioma (**C**), meningioma (**D**), neuroblastoma (**E**) and malignant peripheral nerve sheath tumors (**F**) showing strong positivity for BARHL1 expression. ×20 original magnifications.

In our experiments, first we checked the expression status of BARHL1 in the nervous system and breast cancers. BARHL1 expression was reported in medulloblastoma [14]. In our neuron cancer samples, BARHL1 was found overexpressed in all samples as compared to their normal counterpart tissues, although the degree of overexpression varied depending on the type of cancer. In the tumors of the peripheral and central nervous system, an intense and constant expression was observed (Figure 1A–D). All cases showed BARHL1 expression in more than 75% of cells. The percentage of glioma cases showing expression in 100% of cells was higher (75%) than in meningioma (20%) or peripheral nerve sheath tumors (20%).

**Figure 2:**
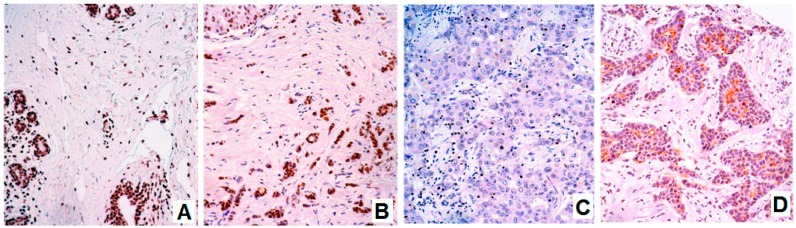
BARHL1 expression in normal and neoplastic breast tissue. (**A**) Ductal epithelial and basal cells showing positive staining, as well as stromal cells. (**B**) Positive staining for a lobular breast carcinoma. (**C**) Negative expression in a ductal poorly-differentiated breast carcinoma. (**D**) Well-differentiated breast carcinoma. Note: cytoplasmic apical lumen staining. ×20 original magnifications.

To the best of our knowledge, the expression of BARHL1 in breast cancer has not been reported so far. In our breast tumor samples, variable expression of BARHL1 was observed (Figure 2A–D). Surprisingly, we found that the expression is downregulated in those tumors with no expression of hormone receptors (20 out of 44 vs. 8 out of 33; *p*(Fisher) = 0.046). Consistently, more BARHL1 negative cases were found amongst the so-called triple negative (hormone receptors and HER2 negative) cases than in those with positive expression of both hormone receptor and HER2 (12 out of 23 vs. 2 out of 13; *p*(Fisher) = 0.0319).

### 3.2. BARHL1 Expression in Alzheimer’s Disease

Estrogen or its receptor ESR1 is downregulated in AD [32], and it has been reported that the decreased estrogen levels in post-menopausal women increase the risk of AD [21]. Short-term estrogen replacement therapy improves cognition, i.e., visual and semantic memory in AD-affected postmenopausal women [33]. Based on these reports and our observations in hormone-negative breast cancer samples, we postulated that the expression of BARHL1 may be under the regulation of estrogen. To investigate if there is a regulatory link between estrogen and BARHL1 in AD, we first tested the expression of BARHL1 in AD samples. As expected, the expression of BARHL1 in AD hippocampus was more limited (≤75%: 100% vs. 0%; *p*(Fisher) = 0.001) and less intense (A: 60% vs. 0%; *p*(Pearson) = 0.04) than in control matched brain tissue (Table 2). In Parkinson’s disease samples, we did not find much difference in BARHL1 expression with the controls, and in lateral amyotrophic sclerosis samples, BARHL1 is more intense than the controls (Table 2).

### 3.3. Putative Estrogen-BARHL1 Axis in Alzheimer’s Disease

To explore possible molecular mechanisms elucidating the implication of the estrogen-BARHL1 axis in AD patho-physiology, we employed multiple bioinformatics approaches.

### 3.4. Estrogen-BARHL1 Network

**Figure 3:**
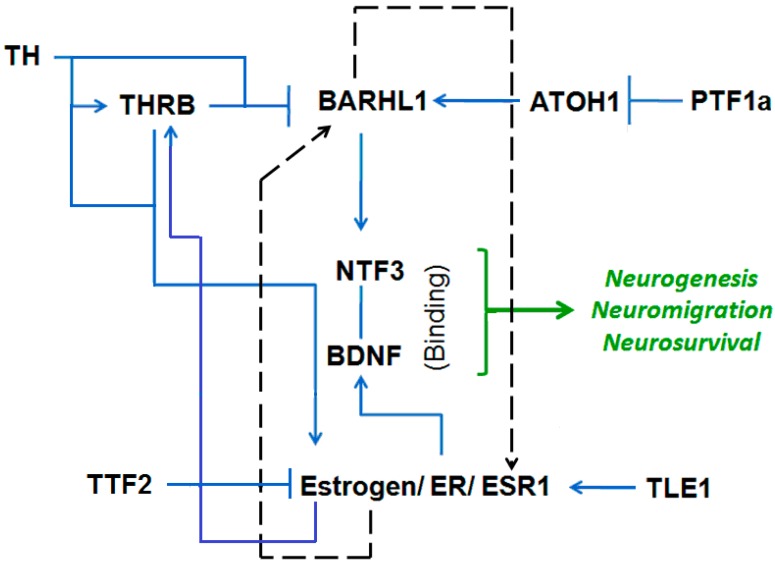
The estrogen-BARHL1 network (see the text for detail). The solid arrow indicates activation/positive regulation; T denotes inhibition; and the dotted arrow designates newly-suggested regulatory networks based on our analysis. TH: thyroid hormone; THRB: thyroid hormone receptor beta; ATOH1: atonal BHLH transcription factor 1; PTF1a: pancreas specific transcription factor 1a; NTF3: neurotrophin 3; BDNF: brain-derived neurotrophic factor; TTF2: transcription termination factor 2; Estrogen/ER/ESR1: estrogen/estrogen receptor/estrogen receptor 1; TLE1: transducin like enhancer of split 1.

BARHL1 positively regulates neurogenesis, neural survival and neuron migration in developing brain. We developed an estrogen-BARHL1 regulatory network based on literature mining (Figure 3). The main findings and references of each work based on which the network is developed are described in Table 3. From this network, we observed that Neurotrophin 3 (NTF3) probably binds to the Brain Derived Neurotrophic Factor (BDNF) and jointly regulate neurogenesis and neural survival. While BARHL1 activates NTF3 [13], BDNF is positively regulated by estrogen [41]. Thus, both BARHL1 and estrogen positively regulate neurogenesis in brain through NTF3 and BDNF, respectively. Although it is unclear that BARHL1 and estrogen regulate each other, it was found that thyroid hormone (TH) is a common regulator for both of these genes. TH on the one hand inhibits BARHL1 expression through THRB [15] and on the other hand directly induces estrogen receptor (ESR1) signaling [37]. Therefore, by combining the literature mining and our expression analysis (low expression of BARHL1 in estrogen-negative conditions), it can be concluded that: (i) there are two sub-regulatory networks behind neurogenesis and neuron migration; one implying BARHL1 (through NTF3) and the other by estrogen (through BDNF); (ii) when both the ER and BARHL1 are downregulated in AD (as found in our expression studies), both the NTF3- and BDNF-mediated neurogenesis and neural survival are blocked, causing neuron degeneration in AD.

### 3.5. Estrogen and BARHL1 Regulate Each Other

Since from the literature mining-based BARHL1 circuit, we could not conclude that BARHL1 is under the control of estrogen, we searched estrogen-regulated high throughput gene expression data in CTD [25]. We observed that ethynyl estradiol, which is a derivative of 17β-estradiol (E2), increases the expression of BARHL1 mRNA in mouse uterine tissue in a microarray analysis [43]. Further, in human hepatocytes, ethynyl estradiol upregulates *BARHL1* gene expression [44]. Therefore, estrogen promotes the expression of BARHL1.

**Figure 4:**
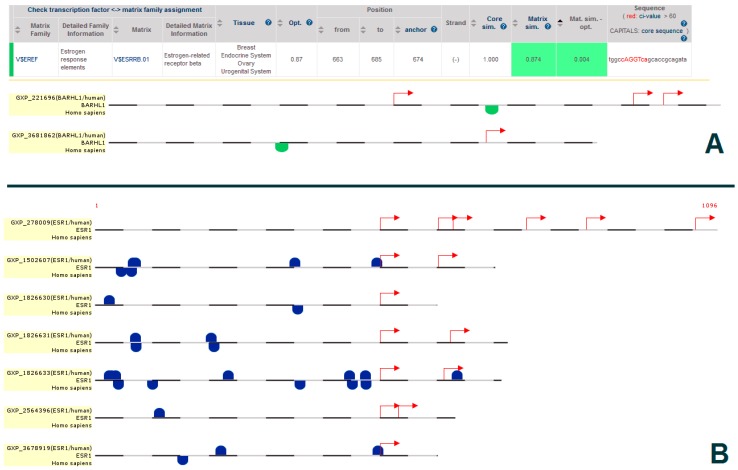
(**A**) ESR1 binding sites in the *BARHL1* promoter and (**B**) BARHL1 binding sites in the *ESR1* promoter. The green dots show V$EREF matrix family binding sites at BARHL1 promoter and blue dots indicate the V$HOMF matrix family binding sites at ESR1 promoter. The red arrows indicate the transcription start sites.

Moreover, to find if the estrogen-mediated BARHL1 expression is a direct effect of ESR1, we performed BARHL1 promoter analysis to check if there are ESR1 binding sites. From the MatInspector module [27] of the Genomatix suite (www.genomatix.de), we could find that ESR1 can bind to the *BARHL1* promoter at nucleotide positions 663–668 having the sequence tggccAGGTcagcaccgcagata, and this positively regulates BARHL1 expression (Figure 4A); thus, suggesting that BARHL1 is under direct control of estrogen. Therefore, our analysis supports the previous findings of estrogen regulation by BARHL1 [25,44]. Additionally, we also found that BARHL1, which is a Homeodomain group of transcription factors, has multiple binding sites in the *ESR1* promoter (Figure 4B), adding further support to the possibility that estrogen and BARHL1 regulate each other (Figure 3, dotted arrow).

### 3.6. The BARHL1-ESR1 Axis May Regulate a Subset of the Alzheimer’s Disease Pathway

Next, we checked if the key TFs from the BARHL1 network bind to the promoter of unique genes from the AD pathway or, alternatively, AD pathway-associated TFs could bind to promoters from the BARHL1 network genes so that these two pathways interact with each other. In the BARHL1 pathway, BARHL1, ESR1, BDNF, THRB and ATOH1 are the TFs; whereas TLE1 (Transducin like enhancer of split 1) is a transcription corepressor. Out of 57 AD pathway-specific genes from KEGG, only ATF6 was found to be a TF (Table S1, unique genes).

Based on the MatInspector analysis, we found that the HOMF matrix family, which consists of homeodomain TFs including BARHL1, and the EREF matrix family, consisting of estrogen response elements including ESR1, bind to the promoters of 50 out of 57 unique genes of the AD pathway (Figure S1). More specifically, it was found that BARHL1 have binding sites in the promoters of GNAQ (G protein subunit alpha q), ATP2A2 (ATPase sarcoplasmic/endoplasmic reticulum Ca2+ transporting 2) and PLCB4 (Phospholipase C beta 4) and ESR1 binds to promoters of 37 genes of the AD pathway (Tables S2 and S3). Furthermore, we used the ToppFun module of ToppGene to annotate these ESR1 and BARHL1-regulated genes to understand how these two genes modulate AD biology. We found that the BARHL1-regulated genes GNAQ, ATP2A2 and PLCB4 (Table S2) are specifically involved in potassium ion transport and the glutamate receptor group I pathway (Table S4), which are impaired in AD [45]. The 37 genes (Table S3) of the ESR1 targets are involved in calcium ion transport (Table S5), which is deregulated in AD [46]. Therefore, at the transcriptional level, it may be inferred that the BARHL1-ESR1 and AD networks interplay with each other, and the downregulation of BARHL1 and ESR1 may impair the potassium and calcium transport that is downregulated in AD. However, we could not find any BARHL1-ESR network gene that is regulated by ATF6 in the MatInspector analysis. Thus, BARHL1 and ESR1 are probably involved in AD patho-physiology by modulating potassium and calcium ion transport and glutamate receptor group I pathways.

### 3.7. Role of the BARHL1-ESR1 Network in Alzheimer’s Disease

The functional enrichment of the nine genes implicated in the BARHL1 network (Table S6, Column 1) obtained by ToppFun shows that the BARHL1-ESR1 network is involved in neuron death/apoptosis, sensory perception of sound and mechanical stimulus, brain development and behavior (Table S7). Furthermore, the enriched disease showed that the BARHL1-ESR1 network is associated with several neurological disorders including AD (Table S7). Baseline hearing loss increases the risk of AD [47], and central auditory processing dysfunction is observed in AD subjects [48]. Therefore, deregulation of the BARHL1-ESR1 network may be involved in hearing impairment in AD severity, and replacement therapy with estrogen could improve AD condition [49,50] by restoring the network and therefore improving the cognitive ability.

### 3.8. Links between the BARHL1-ESR Axis and the Alzheimer’s Disease Pathway

We generated VisANT-based intra-PPIs for the BARHL1-ESR1 network of nine genes (Table S6, Column 1) and inter-PPIs among the key AD pathway (20 genes) (Table S6, Column 3) and the nine genes of the BARHL1-ESR1 circuit. The resultant networks were analyzed for centrality, and using VisANT, the identification of the shortest pathway-based key nodes was carried out. We observed that MED7 (Mediator complex subunit 7) is the key node and that ESR1-MED7-BARHL1 constitutes the shortest path in the BARHL1 intra-network (Table S8, highlighted with yellow). There are 41 linker proteins in the shortest pathways that link BARHL1-ESR1 and AD pathways (Table S6, Column 5 and Table S8). Out of these 41 genes, 25 genes are directly associated with AD as per previous reports (Table S6, Columns 5 and 6). Considering these inter-PPIs between BARHL1-ESR1 and key AD pathway proteins, ubiquitin C (UBC) is found to be one of the key nodes (Table S8, highlighted with turquoise). While the BARHL1-ESR1 network’s BARHL1 is linked to the AD network through the BARHL1-MED7-UBC-APP (Amyloid beta precursor protein) shortest path (Table S8, red bold), ESR1 within that circuit is directly connected with the AD pathway through UBC (Table S8, blue bold highlighted with green). Furthermore, BARHL1 is also linked to key proteins from the AD pathway through TLE1 (Table S8).

**Figure 5:**
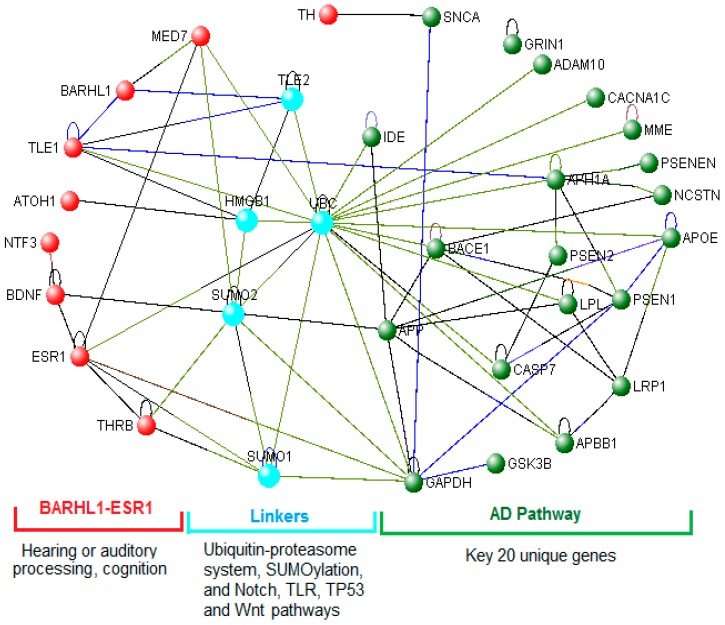
Key linkers (UBC, SUMO1, SUMO2, HMGB1 and TLE2) between the BARHL1-ESR1 axis and the Alzheimer’s Disease (AD) pathway.

Based on shortest pathways combined with centrality analysis, it was observed that the key linkers/nodes between the BARHL1-ESR1 and key AD pathways are: UBC, SUMO1 (Small ubiquitin-like modifier 1), SUMO2 (Small ubiquitin-like modifier 2), HMGB1 (High mobility group box 1) and TLE2 (Transducin like enhancer of split 2) (Figure 5). Except TLE2, the other four proteins are directly associated with AD (Table S6, Columns 5 and 6, bold highlighted). ToppFun-based functionality/Gene Ontology (GO) analysis shows that all five genes regulate transcription, three of which (UBC, SUMO1, SUMO2) are involved in proteasome-mediated ubiquitin-dependent protein catabolism. Two genes (SUMO1, SUMO2) regulate SUMOylation, and two (UBC, TLE2) are involved in Notch signaling. The pathway annotations by ToppFun also showed similar results as GO. In addition, it also gives the p53 pathway (HMGB1, SUMO1, SUMO2), TLR (Toll-like receptor) and apoptosis (HMGB1, UBC) and the Wnt signaling pathway (SUMO1, SUMO2) (Supplementary Table S9). The ubiquitin-proteasome system (UPS), which could be a potential therapeutic target [51], is involved in APP metabolism, and defects in this system have been documented in AD [52]. Similarly, the SUMOylation [53], Notch [54], TLR [55] and Wnt signaling [56] pathways are deregulated and TP53 [57] upregulated in AD.

Taken together, our results suggest that BARHL1-ESR1 may play an important role in hearing or auditory processing function, and deregulation of this network potentially increases the severity of AD. Furthermore, the BARHL1-ESR1 network modulates the AD pathogenic pathway through the ubiquitin-proteasome system, SUMOylation and the Notch, TLR and Wnt signaling components that are directly reported to be associated with AD.

### 3.9. miRNAs Regulate the BARHL1-ESR1 Axis and the Alzheimer’s Disease Network

To explore if the BARHL1 and AD networks are interlinked at the miRNA level and regulated by common miRNAs, we used our reverse transcriptomics approach [31] in combination with miRNome and miRNA pathway analysis. To understand the correlations, we first collected the deregulated miRNAs in AD from published literature available in PubMed. In total, 187 miRNAs (50 upregulated and 137 downregulated) were found to be associated with AD (Table S10). Next, we attempted to identify the miRNAs that target BARHL1-ESR network genes (nine genes) and the AD pathway’s 20 key unique genes (Table S6, Columns 1 and 3, respectively) using miRwalk [29].

We found that, out of the 20 key unique genes of AD pathway, only 13 genes (*APP*, *ADAM10*, *GAPDH*, *BACE1*, *LRP1*, *APOE*, *MME*, *SNCA*, *GRIN1*, *CACNA1C*, *CASP7*, *GSK3B* and *LPL*) have validated targeting miRNAs; whereas, out of nine genes of the BARHL1-ESR network, only five genes (*ESR*, *BDNF*, *TLE1*, *ATOH1*, *THRB*) have validated targeting miRNAs in miRwalk (Table S11). For those genes that have no validated targeting miRNAs, the prediction option of miRwalk was chosen (Table S12).

Next, we identified common miRNAs that have targets in both the key AD pathway and BARHL1-ESR1 network genes by combining and comparing data from Tables S11 and S12. We found that 44 miRNAs have targets from both the AD and BARHL1 networks, and these miRNAs cumulatively may target most of the genes of these two pathways (six from the BARHL1-ESR network and 14 from the AD pathway). The 6 genes of the BARHL1-ESR network are *BDNF*, *TH*, *THRB*, *NTF3*, *BARHL1* and *ESR1*, and the 14 genes of the AD pathway are *BACE1*, *LRP1*, *NCSTN*, *APH1A*, *ADAM10*, *APOE*, *CACNA1C*, *LPL*, *IDE*, *GAPDH*, *SNCA*, *GSK3B*, *APP* and *PSENEN* (Table S13).

### 3.10. Functions of the BARHL1 and Alzheimer’s Disease Networks Regulating miRNAs

To understand if these 44 miRNAs have any role in AD patho-physiology, we used the tool for annotations of human miRNAs (TAM) [30], which mines potential biological functions of a group of miRNAs. We used these 44 miRNAs as input and the entire set of AD associated miRNAs that are listed in Table S10 as background miRNAs. We set the overrepresentation (enriched miRNA families) and ‘Size of miRNA’ category between one and 44. According to the *p*-values, we observed that hsa-miR-181a is the best cluster (*p*-value: 0.0276); hsa-miR-30 is the best miRNA family (*p*-value: 2.91 × 10^−3^); and apoptosis having 10 miRNAs is one of the best functions (*p*-value: 7.97 × 10^−3^). hsa-miR-30 and hsa-miR-181 are downregulated [58], and apoptosis is a key mechanism in AD [59]. Similarly, the brain development that is impaired in AD [60,61] shows four miRNAs with a *p*-value: 0.2727 as per the TAM analysis. Moreover, the HMDD-supported [62] TAM annotations were enriched in schizophrenia (nine miRNAs, *p*-value: 8.39 × 10^−4^), PD (five miRNAs, *p*-value: 3.71 × 10^−3^) and AD (four miRNAs: hsa-mir-107, hsa-mir-17, hsa-mir-21, hsa-mir-29b; *p*-value: 0.0281). When we enriched the AD pathway and common genes associated with the BARHL1-ESR1 network (*ESR1*, *BDNF*, *BACE1*, *TH*, *NCSTN*, *IDE*, *CACNA1C*, *APH1A*, *THRB*, *NTF3*) that are targets of these four miRNAs (hsa-mir-107, hsa-mir-17, hsa-mir-21, hsa-mir-29b) (Table S13) using ToppFun, we observed that these genes (and therefore, these miRNAs) regulate the β-amyloid metabolic process, behavior, learning or memory, the neuron apoptotic process, cognition and synaptic transmission at a *p*-value less than 5.604 × 10^−4^. A similar result was observed when we used the targets of hsa-let-7b, hsa-mir-18a, hsa-mir-21, hsa-mir-30b and hsa-mir-101 that have the most common targets from both the AD and BARHL1-ESR1 networks (Table S13) for ToppFun analysis. Similarly, we enriched the targets of known deregulated miRNAs in AD (Tables S10 and S14, respectively) using ToppFun and observed that the targets of the downregulated miRNAs in AD mostly work in programmed cell death (*p*-value: 1.008 × 10^−112^) or the apoptotic process (*p*-value: 1.483 × 10^−111^). Likewise, the upregulated miRNAs in AD are also involved in the regulation of cell death (*p*-value: 4.795 × 10^−105^) and programmed cell death (*p*-value: 4.670 × 10^−99^). Therefore, this miRNA-based analysis also suggests that the BARHL1-ESR1 network has an important role in AD patho-physiology by regulating β-amyloid metabolism, neuronal death and memory.

### 3.11. hsa-mir-18a May Regulate the BARHL1-AD Network and Alzheimer’s Disease Patho-Physiology

Next, we tried to correlate if the miRNA deregulation and the expression of their targets correlate in AD by applying the general rule that if an miRNA is upregulated, its targets will be downregulated. In AD, *SNCA* [63], *BACE1* [64], *APH1A* [65] and *APP* [66] are upregulated; while *LRP1* [67], *IDE* [68], *PSENEN* [65], *GAPDH* [69] and *LPL* [70] are downregulated. *NCSTN* mutation is observed in AD patients [71] and downregulated in a mouse model of AD [72]. For the 44 miRNAs having targets from both the AD and BARHL1 pathways (Table S13), we observed that only hsa-mir-18a meets these criteria. has-mir-18a is upregulated [73,74], and its targets *BDNF* [75], *TH* [76] and *NCSTN* [72] are downregulated in AD. hsa-mir-18a also targets *ESR1*, and *BARHL1* is its putative target (Table S13). The available literature suggests that BDNF provides neuroprotection [77,78] by reducing the level of β-amyloid [79]; TH regulates normal development and functions of the central nervous system [80]; and NCSTN modulates Notch signaling and is essential for APP cleavage and regulation of β-amyloid processing [81,82]. Similarly, downregulation of ESR1 correlated with cognitive decline in AD [83,84], and BARHL1 regulates expression of ESR1 (as per our study). A ToppFun analysis of *ESR1*, *BDNF*, *TH*, *BARHL1* and NCSTN shows that these genes are involved in negative regulation of neuron death (*p*-value: 7.677 × 10^−6^), learning (*p*-value: 4.622 × 10^−4^), sensory perception of sound (*p*-value: 6.185 × 10^−4^) and Alzheimer’s disease (*p*-value: 4.232 × 10^−6^). Since hsa-mir-18a targets all five genes, it may regulate the neuronal death and cognitive impairment associated with AD. Therefore, the role of this miRNA needs to be further explored for its precise function in AD patho-physiology.

### 3.12. BARHL1 Knockout Mimics Alzheimer’s Disease Symptoms

*BARHL1* KO mice show visual disturbances and cognitive decline in mid-to-late stage AD resulting from high accumulation of Tau, amyloid and neuritic plaques in subcortical visual centers like the superior colliculus [85,86,87]. Alzheimer’s and Parkinson’s diseases that share some common symptoms of neuron apoptosis [88] show degeneration of basal forebrain cholinergic cell groups including superior colliculus [89]. Abnormal Tau phosphorylation/accumulation/cleavage causes neurotoxicity and subsequent neural death in AD [59,90], and β-amyloid induces neuronal apoptosis through multiple mechanisms [59,91,92,93].

Cerebellar granule cell numbers are dramatically decreased in AD [5,6], are shorter, less branched and have fewer spines [94]. A 12.7% reduction in total cerebellar volume was observed in AD patients [7]. Amyloid precursor proteins are deposited in cerebellar granule cells of AD [95], and increased β-amyloid deposition causes apoptosis of these cerebellar granule cells [96].

Data also suggest that mossy fibers that regulate spatial memory [8] are disrupted in a mouse model of familial AD [9]. Ultrastructural alterations in mossy fibers are observed in AD patients, which may cause memory impairment [10]. Aberrant Tau phosphorylation in these fibers is observed in AD, which may lead to death of these cells and memory impairment [97].

Our literature mining revealed that the *BARHL1* KO mouse exhibits loss of many neurons due to apoptosis of the zonal layer of the superior colliculus [98] and the cerebellar granule cells that project mossy fibers [13]. Knockout also causes defective radial migration of cerebellar granule cells [13]. Other studies show that knockout of *BARHL1* causes age-related progressive degeneration of both outer and inner hair cells of the organ of Corti, which leads to complete hearing loss at 10 months of age [99]. Hearing loss is associated with AD [11], and persons with hearing loss are at risk of AD [100]. Although loss of the central auditory system is observed in AD, no evidence is reported about hair cell loss in AD, although this loss is observed in presbycusis [101].

Taken together, BARHL1 plays an important role in the survival and migration of cerebellar and precerebellar neurons and long-term maintenance of superior colliculus neurons in brain, and thus, deregulation of BARHL1 in AD may regulate cognitive functions.

### 3.13. Estrogen-ESR1 and Alzheimer’s Disease

ESR1 polymorphism is associated with the risk of AD [84,102,103] and cognitive decline [17,83]. The *PvuII* (rs2234693) and *XbaI* (rs9340799) polymorphisms in the intron-1 of *ESR1* cause reduced expression of the *ESR1* gene [16] associated with increased risk of AD, faster cognitive decline [17,18] and differential response to cholinesterase inhibitor (Donepezil and Rivastigmine) treatment in AD women [104]. The *ESR1* non-coding deletion 1 polymorphism is also a risk factor for AD in women [103] that causes differential expression of ESR1 isoforms and is associated with response to estradiol stimulus. Reports suggest that estrogen deficiency may lead to AD and that ERT reduces the risk [19,20,21].

Our results suggest that BARHL1 and ESR1 regulate each other’s expression, and therefore, downregulation of BARHL1 in AD (as shown in our TMA study) downregulates the expression of ESR1 and, therefore, decreases estradiol response and contributes to AD pathogenesis. Further, 17β-estradiol was found to alter hippocampal function through increased expression of BDNF in the mossy fibers pathway. Estradiol or estrogen requires normal ESR1 expression for its optimal function, the interaction between BDNF and estradiol in the mossy fibers being important for the normal function of hippocampus [105]. 17β-estradiol reduces Aβ-amyloid-induced neurotoxicity and apoptosis thus protecting cerebellar granule cells [106,107]. The downregulation of ESR1 caused by decreased BARHL1 expression or vice versa in AD hampers this interaction; it also provides a possible new mechanism to explain the abnormal function of mossy fibers and cerebellar granule cells related to memory and cognitive decline in AD apart from the Tau and amyloid pathogenesis.

## 4. Conclusions

This in silico study demonstrates an association between BARHL1 and estrogen receptor signaling, which might be related to memory and cognitive decline in AD. Immunohistochemical studies support this bioinformatic observation as BARHL1 expression is downregulated in the hippocampus of patients with AD compared to the normal hippocampus. Moreover, the immunohistochemical study also demonstrated an association between BARHL1 downregulation and estrogen hormone negative breast cancers, which favors the BARHL-ESR1 link and might explain why estrogen replacement therapy improves the cognate condition of AD patients. A *BARHL1/ESR1* double knockout mouse model may be generated as a potential new AD model to verify these findings and to test the efficacy of estrogen replacement therapy using this new AD model. Further, the diagnostic and therapeutic potential of both BARHL1 and ESR1 alone or in combination in AD needs to be validated using a large cohort of AD patients.

## Figures and Tables

**Table 1:** Number and types of cancer and neurodegenerative disease samples evaluated in this study.

Samples	Types	Number of Samples
Breast tumors	HR−/HER2−	23
	HR−/HER2+	21
	HR+/HER2−	20
	HR+/HER2+	13
	**Total**	**77**
Nervous system tumors	Neuroblastoma	4
	Meningioma	5
	Glioma	4
	Peripheral nerve sheath	3
	**Total**	**16**
Neurodegenerative diseases	Alzheimer’s disease	10
	Lateral amyotrophic sclerosis	6
	Parkinson’s disease	1
	**Total**	**17**
	**Total**	**110**

HR: Hormone receptor; HER2: Human epidermal growth factor receptor 2.

**Table 2:** BarH like homeobox 1 (BARHL1) staining features in neurodegenerative diseases.

		Percentage of Cells (%)					Intensity			
**Type of tissue**		0	<50	50–75	>75	100	A	B	C	D
**Control**	Hippocampus	0	0	0	2	2	0	1	1	2
	Olfactory bulb	0	0	0	1	1	0	0	2	0
	Medulla	1	0	1	1	1	1	0	2	1
**Disease**	Alzheimer’s disease	5	3	2	0	0	5	2	2	0
	Parkinson’s disease	0	1	0	0	0	0	1	0	0
	Lateral amyotrophic sclerosis	0	1	2	0	3	0	2	2	2

A: no staining; B: weak staining; C: moderate staining; D: strong staining.

**Table 3:** Regulation of BARHL1 and Estrogen Receptor 1 (ESR1) as evidenced from the literature.

Regulation of BARHL1 and ESR1	References
BARHL1 upregulates NT-3 (neuro tropin 3 in mouse cerebellum) and thereby regulates the survival of cerebellar granule cells.	Li et al., 2004 [13]
The BARHL1 promoter has a TRβ binding site, and T3 (thyroid hormone) inhibits the expression of BARHL1. Thus, Brahl1 plays a role in impaired neuro-development caused by hypothyroidism.	Dong et al., 2011 [15]
ATOH1/MATH1 upregulates BARHL1 in inner ear and central nervous system.	Chellappa et al., 2008 [34]
ATOH1/MATH1 is repressed by PTF1a.	Pascual et al., 2007 [35]
TLE1 positively regulates ER-mediated gene expression and cell division.	Holmes et al., 2012 [36]
Thyroid hormone (T3) phosphorylates and activates ERα.	Meng et al., 2011 [37]
Estrogen positively regulates THRB in fish.	Filby et al., 2006 [38]
TTF2 inhibits transactivation of estrogen receptor-alpha in breast cancer cells.	Park et al., 2012 [39]
Estrogen increased the expression of NTF3, BDNF and NGF proteins.	Bimonte et al., 2004 [40]
Estrogens increase BDNF levels in the medial prefrontal cortex (PFC) and the hippocampus.	Luine et al., 2013 [41]
Estradiol induces the BDNF expression and positively regulates dendritic growth, spinogenesis and synaptogenesis in the developing Purkinje cell.	Zhu et al., 2013 [42]

## Supplementary Materials

The following are available online at www.mdpi.com/2073-4425/8/10/245/s1. Figure S1: HOMF and EREF matrix family TFs bind to 50 AD genes; Table S1: Unique and common genes of AD, PD and ALS collected from KEGG; Table S2: BARHL1 specifically binding to 3 AD-specific genes as per MatInspector; Table S3: ESR1 specifically binding to 37 AD-specific genes as per MatInspector; Table S4: Functional enrichment and annotation analysis of GNAQ, ATP2A2 and PLCB4 by ToppGene (ToppFun); Table S5: Functional enrichment and annotation analysis of 37 ESR1 target genes (Table S3) by ToppGene (ToppFun); Table S6: BARHL1-ESR1 and AD pathway genes along with protein-protein interaction-derived shortest pathway linkers and their AD-related functions; Table S7: Functional enrichment of the BARHL1 network (nine genes) by ToppFun; Table S8: Protein-protein interaction (PPI)-based shortest pathway and key node analysis between BARHL1-ESR1 and AD pathway key proteins; Table S9: Functional enrichment and annotation analysis of UBC, SUMO1, SUMO2, HMGB1 and TLE2 by ToppFun; Table S10: List of up-/down-regulated miRNAs in AD collected from the PubMed literature; Table S11: Validated targeting miRNAs of BARHL1-ESR1 and AD pathway key genes derived from miRWalk; Table S12: Predicted targeting miRNAs of BARHL1-ESR1 and AD pathway-related key genes having no validated target in miRwalk; Table S13: Common miRNAs targeting AD and BARHL1-ESR1 pathway genes and their targets. The table also shows the regulation of targeting miRNAs in AD. Red genes are predicted targets; Table S14: Experimentally-validated targets of miRNAs deregulated in AD. The miRNAs are selected from Supplementary Table S10, and targets were identified using miRWalk.
