# Supplementary material for: BARHL1 Is Downregulated in Alzheimer’s Disease and May Regulate Cognitive Functions through ESR1 and Multiple Pathways
Source: Genes (Basel). 2017 Sep 28;8(10):245. doi: 10.3390/genes8100245 (PMC5664095; doi:10.3390/genes8100245)
Supplement: Supplementary file 1 [file genes-08-00245-s001.zip › Supplementary information_BARHL1_Debmalya_12-9-17 [R1].doc]

**Supplementary information**

Debmalya Barh ^1,2,^*, María E. García-Solano ^3,4^, Sandeep Tiwari ^1,2^, Antaripa Bhattacharya ^1^, Neha Jain ^1^, Daniel Torres-Moreno ^3,4^, Belén Ferri ^5^, Artur Silva ^6^, Vasco Azevedo ^2^, Preetam Ghosh ^1,7^, Kenneth Blum ^8^, Pablo Conesa-Zamora ^3,4^ and George Perry ^9,10^

^1^	Centre for Genomics and Applied Gene Technology, Institute of Integrative Omics and Applied Biotechnology, Nonakuri, Purba Medinipur, West Bengal 721172, India; sandip_sbtbi@yahoo.com (S.T.); antaripa1210@gmail.com (A.B.); [dr.barh@gmail.com](mailto:dr.barh@gmail.com) (D.B.); [mymailtoneha@gmail.com](mailto:mymailtoneha@gmail.com) (N.J.)

^2^	Instituto de Ciências Biológicas, Universidade Federal de Minas Gerais, Belo Horizonte, MG 31270-901, Brazil; vascoariston@gmail.com

^3^	Department of Pathology, Santa Lucía General University Hospital (HGUSL), C/Mezquita s/n, 30202, Cartagena, Spain; daniel-torres@orange.es (D.T.-M.); pablo.conesa@carm.es (P.C.-Z.); garciasolano2002@yahoo.com (M.E.G.-S.)

^4^	Catholic University of Murcia (UCAM), Murcia, Spain

^5^	Department of Pathology, Virgen Arrixaca University Hospital (HUVA), Ctra. Madrid Cartagena sn, 30120 El Palmar, Spain; belen.ferri@carm.es

^6^	Instituto de Ciências Biológicas, Universidade Federal do Pará, Rua Augusto Corrêa, 01-Guamá, Belém, PA 66075-110, Brazil; arturluizdasilva@gmail.com

^7^	Department of Computer Science, Virginia Commonwealth University, Richmond, VA 23284, USA; pghosh@vcu.edu

^8^	Department of Psychiatry & McKnight Brain Institute, University of Florida College of Medicine, Gainesville, FL 32610, USA; drd2gene@gmail.com

^9^	UTSA Neurosciences Institute and Department of Biology, University of Texas at San Antonio, San Antonio, TX 78249, USA; george.perry@case.edu

^10^	Department of Pathology, School of Medicine, Case Western Reserve University, Cleveland, OH 44106, USA

*****	Correspondence: dr.barh@gmail.com; Tel: +91-944-955-0032

**Contents**

**Supplementary figure-1**

**Supplementary table S1-S14**

**Supplementary Figure-1:** HOMF and EREF matrix family TFs bind to 50 AD genes.

**Supplementary Table S1:** Unique and common genes of AD, PD and ALS collected from KEGG.

| **UNIQUE GENES FROM KEGG PATHWAYS** | | | | | | | |
| --- | --- | --- | --- | --- | --- | --- | --- |
| **ALZHEIMER PATHWAY (hsa05010)** | | **PARKINSON PATHWAY (hsa05012)** | | | | **ALS PATHWAY (hsa05014)** | |
| **ALL GENES** | **UNIQUE GENES** | **ALL GENES** | | **UNIQUE GENES** | | **ALL GENES** | **UNIQUE GENES** |
| ADAM10 | ADAM10 | UBA1 | | SLC25A5 | | TP53 | CCS |
| ADAM17 | ADAM17 | UBA7 | | SLC25A6 | | PPP3CA | CAT |
| APP | APP | UBB | | UBA1 | | PPP3CB | PRPH |
| NAE1 | FADD | UBE2L3 | | UBA7 | | PPP3CC | PRPH2 |
| GAPDH | NAE1 | UBE2L6 | | UBB | | PPP3R1 | NEFL |
| BACE1 | GAPDH | UBE2J2 | | UBE2L3 | | PPP3R2 | NEFM |
| BACE2 | BACE1 | UBE2J1 | | UBE2L6 | | CHP1 | NEFH |
| PSENEN | BACE2 | UBE2G2 | | UBE2J2 | | CHP2 | ALS2 |
| PSEN1 | PSENEN | UBE2G1 | | UBE2J1 | | CASP1 | RAB5A |
| PSEN2 | PSEN1 | PARK2 | | UBE2G2 | | BID | RAC1 |
| NCSTN | PSEN2 | SNCA | | UBE2G1 | | BCL2 | SLC1A2 |
| APH1A | NCSTN | GPR37 | | PARK2 | | BCL2L1 | GRIA1 |
| IDE | APH1A | CASP3 | | GPR37 | | BAX | GRIA2 |
| MME | IDE | SNCAIP | | SLC25A4 | | BAD | GPX1 |
| NDUFV1 | MME | UCHL1 | | SLC25A31 | | APAF1 | TP53 |
| NDUFV2 | LPL | TH | | SNCAIP | | CYCS | CASP1 |
| NDUFV3 | APOE | SLC6A3 | | UCHL1 | | CASP9 | TNFRSF1B |
| NDUFA1 | LRP1 | SLC18A1 | | TH | | CASP3 | BCL2 |
| NDUFA2 | FAS | SLC18A2 | | SLC6A3 | | SOD1 | BCL2L1 |
| NDUFA3 | CASP8 | ND1 | | SLC18A1 | | TOMM40 | BAX |
| NDUFA4 | CALML3 | ND2 | | SLC18A2 | | TOMM40L | SOD1 |
| NDUFA4L2 | CALM2 | ND3 | | ND1 | | DERL1 | TOMM40 |
| NDUFA5 | CALM3 | ND4 | | ND2 | | CASP12 | TOMM40L |
| NDUFA6 | CALM1 | ND4L | | ND3 | | MAP3K5 | DERL1 |
| NDUFA7 | CALML6 | ND5 | | ND4 | | TNF | DAXX |
| NDUFA8 | CALML5 | ND6 | | ND4L | | TNFRSF1A | MAP2K3 |
| NDUFA9 | GNAQ | NDUFV1 | | ND5 | | TNFRSF1B | MAP2K6 |
| NDUFA10 | PLCB1 | NDUFV2 | | ND6 | | DAXX | MAPK11 |
| NDUFAB1 | PLCB2 | NDUFV3 | | KCTD14 | | MAP2K3 | MAPK12 |
| NDUFA11 | PLCB3 | NDUFA1 | | LRRK2 | | MAP2K6 | MAPK13 |
| NDUFA12 | PLCB4 | NDUFA2 | | PINK1 | | MAPK11 | MAPK14 |
| NDUFA13 | CACNA1C | NDUFA3 | | PARK7 | | MAPK12 | MAP3K5 |
| NDUFB1 | CACNA1D | NDUFA4 | | HTRA2 | | MAPK13 |  |
| NDUFB2 | CACNA1F | NDUFA4L2 | | VDAC1 | | MAPK14 |  |
| NDUFB3 | CACNA1S | NDUFA5 | | VDAC2 | | NOS1 |  |
| NDUFB4 | MAPK1 | NDUFA6 | | VDAC3 | | CCS |  |
| NDUFB5 | MAPK3 | NDUFA7 | | PPIF | | CAT |  |
| NDUFB6 | RYR3 | NDUFA8 | |  | | PRPH |  |
| NDUFB7 | ITPR1 | NDUFA9 | |  | | PRPH2 |  |
| NDUFB8 | ITPR2 | NDUFA10 | |  | | NEFL |  |
| NDUFB9 | ITPR3 | NDUFAB1 | |  | | NEFM |  |
| NDUFB10 | ATP2A1 | NDUFA11 | |  | | NEFH |  |
| NDUFB11 | ATP2A3 | NDUFA12 | |  | | ALS2 |  |
| NDUFS1 | ATP2A2 | NDUFA13 | |  | | RAB5A |  |
| NDUFS2 | ATF6 | NDUFB1 | |  | | RAC1 |  |
| NDUFS3 | ERN1 | NDUFB2 | |  | | SLC1A2 |  |
| NDUFS4 | EIF2AK3 | NDUFB3 | |  | | GRIA1 |  |
| NDUFS5 | CAPN1 | NDUFB4 | |  | | GRIA2 |  |
| NDUFS6 | CAPN2 | NDUFB5 | |  | | GRIN1 |  |
| NDUFS7 | CDK5R1 | NDUFB6 | |  | | GRIN2A |  |
| NDUFS8 | CDK5 | NDUFB7 | |  | | GRIN2B |  |
| NDUFC1 | MAPT | NDUFB8 | |  | | GRIN2C |  |
| NDUFC2 | GSK3B | NDUFB9 | |  | | GRIN2D |  |
| NDUFC2 | CASP7 | NDUFB10 | |  | | GPX1 |  |
| SDHA | IL1B | NDUFB11 | |  | |  |  |
| SDHB |  | NDUFS1 | |  | |  |  |
| SDHC |  | NDUFS2 | |  | |  |  |
| SDHD |  | NDUFS3 | |  | |  |  |
| UQCRFS1 |  | NDUFS4 | |  | |  |  |
| CYTB |  | NDUFS5 | |  | |  |  |
| CYC1 |  | NDUFS6 | |  | |  |  |
| UQCRC1 |  | NDUFS7 | |  | |  |  |
| UQCRC2 |  | NDUFS8 | |  | |  |  |
| UQCRH |  | NDUFC1 | |  | |  |  |
| UQCRHL |  | NDUFC2 | |  | |  |  |
| UQCRB |  | NDUFC2-KCTD14 | |  | |  |  |
| UQCRQ |  | SDHA | |  | |  |  |
| UQCR10 |  | SDHB | |  | |  |  |
| UQCR11 |  | SDHC | |  | |  |  |
| COX3 |  | SDHD | |  | |  |  |
| COX1 |  | UQCRFS1 | |  | |  |  |
| COX2 |  | CYTB | |  | |  |  |
| COX4I2 |  | CYC1 | |  | |  |  |
| COX4I1 |  | UQCRC1 | |  | |  |  |
| COX5A |  | UQCRC2 | |  | |  |  |
| COX5B |  | UQCRH | |  | |  |  |
| COX6A1 |  | UQCRHL | |  | |  |  |
| COX6A2 |  | UQCRB | |  | |  |  |
| COX6B1 |  | UQCRQ | |  | |  |  |
| COX6B2 |  | UQCR10 | |  | |  |  |
| COX6C |  | UQCR11 | |  | |  |  |
| COX7A1 |  | COX3 | |  | |  |  |
| COX7A2 |  | COX1 | |  | |  |  |
| COX7A2L |  | COX2 | |  | |  |  |
| COX7B |  | COX4I2 | |  | |  |  |
| COX7B2 |  | COX4I1 | |  | |  |  |
| COX7C |  | COX5A | |  | |  |  |
| COX8C |  | COX5B | |  | |  |  |
| COX8A |  | COX6A1 | |  | |  |  |
| ATP5A1 |  | COX6A2 | |  | |  |  |
| ATP5B |  | COX6B1 | |  | |  |  |
| ATP5C1 |  | COX6B2 | |  | |  |  |
| ATP5D |  | COX6C | |  | |  |  |
| ATP5E |  | COX7A1 | |  | |  |  |
| ATP6 |  | COX7A2 | |  | |  |  |
| ATP5F1 |  | COX7A2L | |  | |  |  |
| ATP5G1 |  | COX7B | |  | |  |  |
| ATP5G2 |  | COX7B2 | |  | |  |  |
| ATP5G3 |  | COX7C | |  | |  |  |
| ATP5H |  | COX8C | |  | |  |  |
| ATP5O |  | COX8A | |  | |  |  |
| ATP5J |  | ATP5A1 | |  | |  |  |
| ATP8 |  | ATP5B | |  | |  |  |
| LPL |  | ATP5C1 | |  | |  |  |
| APOE |  | ATP5D | |  | |  |  |
| LRP1 |  | ATP5E | |  | |  |  |
| FAS |  | ATP6 | |  | |  |  |
| TNFRSF1A |  | ATP5F1 | |  | |  |  |
| FADD |  | ATP5G1 | |  | |  |  |
| CASP8 |  | ATP5G2 | |  | |  |  |
| BID |  | ATP5G3 | |  | |  |  |
| CALML3 |  | ATP5H | |  | |  |  |
| CALM2 |  | ATP5O | |  | |  |  |
| CALM3 |  | ATP5J | |  | |  |  |
| CALM1 |  | ATP8 | |  | |  |  |
| CALML6 |  | LRRK2 | |  | |  |  |
| CALML5 |  | PINK1 | |  | |  |  |
| PPP3CA |  | PARK7 | |  | |  |  |
| PPP3CB |  | HTRA2 | |  | |  |  |
| PPP3CC |  | VDAC1 | |  | |  |  |
| PPP3R1 |  | VDAC2 | |  | |  |  |
| PPP3R2 |  | VDAC3 | |  | |  |  |
| CHP1 |  | SLC25A4 | |  | |  |  |
| CHP2 |  | SLC25A5 | |  | |  |  |
| BAD |  | SLC25A6 | |  | |  |  |
| CYCS |  | SLC25A31 | |  | |  |  |
| APAF1 |  | PPIF | |  | |  |  |
| CASP9 |  | CYCS | |  | |  |  |
| CASP3 |  | APAF1 | |  | |  |  |
| GNAQ |  | CASP9 | |  | |  |  |
| PLCB1 |  |  | |  | |  |  |
| PLCB2 |  |  | |  | |  |  |
| PLCB3 |  |  | |  | |  |  |
| PLCB4 |  |  | |  | |  |  |
| GRIN1 |  |  | |  | |  |  |
| GRIN2A |  |  | |  | |  |  |
| GRIN2B |  |  | |  | |  |  |
| GRIN2C |  |  | |  | |  |  |
| GRIN2D |  |  | |  | |  |  |
| CACNA1C |  |  | |  | |  |  |
| CACNA1D |  |  | |  | |  |  |
| CACNA1F |  |  | |  | |  |  |
| CACNA1S |  |  | |  | |  |  |
| MAPK1 |  |  | |  | |  |  |
| MAPK3 |  |  | |  | |  |  |
| RYR3 |  |  | |  | |  |  |
| ITPR1 |  |  | |  | |  |  |
| ITPR2 |  |  | |  | |  |  |
| ITPR3 |  |  | |  | |  |  |
| ATP2A1 |  |  | |  | |  |  |
| ATP2A3 |  |  | |  | |  |  |
| ATP2A2 |  |  | |  | |  |  |
| ATF6 |  |  | |  | |  |  |
| ERN1 |  |  | |  | |  |  |
| EIF2AK3 |  |  | |  | |  |  |
| CASP12 |  |  | |  | |  |  |
| NOS1 |  |  | |  | |  |  |
| CAPN1 |  |  | |  | |  |  |
| CAPN2 |  |  | |  | |  |  |
| CDK5R1 |  |  | |  | |  |  |
| CDK5 |  |  | |  | |  |  |
| MAPT |  |  | |  | |  |  |
| GSK3B |  |  | |  | |  |  |
| CASP7 |  |  | |  | |  |  |
| SNCA |  |  | |  | |  |  |
| TNF |  |  | |  | |  |  |
| IL1B |  |  | |  | |  |  |
| **COMMON GENES FROM KEGG PATHWAYS** | | | | | | | |
| **Between Alzheimer and Parkinson** | | | **Between ALS and Alzheimer** | | **Among Parkinson, ALS and Alzheimer** | | |
| SNCA | | | TNFRSF1A | | CYCS | | |
| NDUFV1 | | | BID | | APAF1 | | |
| NDUFV2 | | | PPP3CA | | CASP9 | | |
| NDUFV3 | | | PPP3CB | | CASP3 | | |
| NDUFA1 | | | PPP3CC | |  | | |
| NDUFA2 | | | PPP3R1 | |  | | |
| NDUFA3 | | | PPP3R2 | |  | | |
| NDUFA4 | | | CHP1 | |  | | |
| NDUFA4L2 | | | CHP2 | |  | | |
| NDUFA5 | | | BAD | |  | | |
| NDUFA6 | | | GRIN1 | |  | | |
| NDUFA7 | | | GRIN2A | |  | | |
| NDUFA8 | | | GRIN2B | |  | | |
| NDUFA9 | | | GRIN2C | |  | | |
| NDUFAB1 | | | GRIN2D | |  | | |
| NDUFA10 | | | CASP12 | |  | | |
| NDUFA11 | | | NOS1 | |  | | |
| NDUFA12 | | | TNF | |  | | |
| NDUFA13 | | |  | |  | | |
| NDUFB1 | | |  | |  | | |
| NDUFB2 | | |  | |  | | |
| NDUFB3 | | |  | |  | | |
| NDUFB4 | | |  | |  | | |
| NDUFB5 | | |  | |  | | |
| NDUFB6 | | |  | |  | | |
| NDUFB7 | | |  | |  | | |
| NDUFB8 | | |  | |  | | |
| NDUFB9 | | |  | |  | | |
| NDUFB10 | | |  | |  | | |
| NDUFB11 | | |  | |  | | |
| NDUFS1 | | |  | |  | | |
| NDUFS2 | | |  | |  | | |
| NDUFS3 | | |  | |  | | |
| NDUFS4 | | |  | |  | | |
| NDUFS3 | | |  | |  | | |
| NDUFS4 | | |  | |  | | |
| NDUFS5 | | |  | |  | | |
| NDUFS6 | | |  | |  | | |
| NDUFS7 | | |  | |  | | |
| NDUFS8 | | |  | |  | | |
| NDUFC1 | | |  | |  | | |
| NDUFC2 | | |  | |  | | |
| SDHA | | |  | |  | | |
| SDHB | | |  | |  | | |
| SDHC | | |  | |  | | |
| SDHD | | |  | |  | | |
| UQCRFS1 | | |  | |  | | |
| CYTB | | |  | |  | | |
| CYC1 | | |  | |  | | |
| UQCRC1 | | |  | |  | | |
| UQCRC2 | | |  | |  | | |
| UQCRH | | |  | |  | | |
| UQCRHL | | |  | |  | | |
| UQCRB | | |  | |  | | |
| UQCRQ | | |  | |  | | |
| UQCR10 | | |  | |  | | |
| UQCR11 | | |  | |  | | |
| COX3 | | |  | |  | | |
| COX1 | | |  | |  | | |
| COX2 | | |  | |  | | |
| COX4I2 | | |  | |  | | |
| COX4I1 | | |  | |  | | |
| COX5A | | |  | |  | | |
| COX5B | | |  | |  | | |
| COX6A1 | | |  | |  | | |
| COX6A2 | | |  | |  | | |
| COX6B2 | | |  | |  | | |
| COX6B1 | | |  | |  | | |
| COX6C | | |  | |  | | |
| COX7A1 | | |  | |  | | |
| COX7A2 | | |  | |  | | |
| COX7A2L | | |  | |  | | |
| COX7B | | |  | |  | | |
| COX7B2 | | |  | |  | | |
| COX7C | | |  | |  | | |
| COX8C | | |  | |  | | |
| COX8A | | |  | |  | | |
| ATP5A1 | | |  | |  | | |
| ATP5B | | |  | |  | | |
| ATP5C1 | | |  | |  | | |
| ATP5D | | |  | |  | | |
| ATP5E | | |  | |  | | |
| ATP6 | | |  | |  | | |
| ATP5F1 | | |  | |  | | |
| ATP5G1 | | |  | |  | | |
| ATP5G2 | | |  | |  | | |
| ATP5G3 | | |  | |  | | |
| ATP5H | | |  | |  | | |
| ATP5O | | |  | |  | | |
| ATP5J | | |  | |  | | |
| ATP8 | | |  | |  | | |

**Supplementary Table S2:** BARHL1 specifically binding to AD specific 3 genes as per MatInspector.

| **Genes of AD** | **Matrix Family** | **Detailed Family Information** | **Matrix** | **Detailed Matrix Information** | **From** | **To** | **Anchor** | **strand** | **matrix sim** | **Evidence** | **Sequence** |
| --- | --- | --- | --- | --- | --- | --- | --- | --- | --- | --- | --- |
| GNAQ | V$HOMF | Homeodomain transcription factors | V$BARHL1.01 | BarH-like homeobox 1 | 385 | 403 | 394 | (-) | 0.875 |  | gcccggcTAATtttttgta |
| ATP2A2 | V$HOMF | Homeodomain transcription factors | V$BARHL1.01 | BarH-like homeobox 1 | 284 | 302 | 293 | (+) | 0.878 | ATP2A2 is cocited with V$HOMF (TF: TLX2) | gtccagcTAATtttttgta |
| PLCB4 | V$HOMF | Homeodomain transcription factors | V$BARHL1.01 | BarH-like homeobox 1 | 318 | 336 | 327 | (-) | 0.875 |  | acctggcTAATtttttgta |

**Supplementary Table S3: ESR1 specifically binding to AD specific 37 genes as per MatInspector.**

| **Genes of AD** | **Matrix Family** | **Detailed Family Information** | **Matrix** | **Detailed Matrix Information** | **From** | **To** | **Anchor** | | **strand** | **matrix sim** | **Evidence** | **Sequence** |
| --- | --- | --- | --- | --- | --- | --- | --- | --- | --- | --- | --- | --- |
| BACE2 | V$EREF | Estrogen response elements | V$ER.04 | Estrogen response elements, IR3 sites | 80 | 102 | 91 | (+) | | 0.911 | BACE2 is cocited with V$EREF (TF: ESR1) | aggggagGTCAcgttggatcggg |
| APH1A | V$EREF | Estrogen response elements | V$ESRRA.02 | Estrogen-related receptor alpha (secondary DNA binding preference) | 651 | 673 | 662 | (+) | | 0.986 |  | gggaggGGTCagtggtgaacagg |
| NCSTN | V$EREF | Estrogen response elements | V$ERR.01 | Estrogen related receptor | 167 | 189 | 178 | (+) | | 0.935 | NCSTN is cocited with V$EREF (TF: ESR1) | gcccAAGGacacaccaaagaaga |
| APP | V$EREF | Estrogen response elements | V$ER.04 | Estrogen response elements, IR3 sites | 655 | 677 | 666 | (+) | | 0.879 | APP is cocited with V$EREF (TF: ESR1,ESR2) | ggcctggGTCAagttgtggtcac |
| BACE1 | V$EREF | Estrogen response elements | V$ESRRA.01 | Estrogen-related receptor alpha | 386 | 408 | 397 | (-) | | 0.898 |  | tggcAAGGtcttctacaggctac |
| FADD | V$EREF | Estrogen response elements | V$ESRRA.01 | Estrogen-related receptor alpha | 290 | 312 | 301 | (-) | | 0.874 |  | gaccAAGGttacttatgcattaa |
| GAPDH | V$EREF | Estrogen response elements | V$ESRRA.01 | Estrogen-related receptor alpha | 146 | 168 | 157 | (-) | | 0.901 | GAPDH is cocited with V$EREF (TF: ESR1,ESR2) | gcccAAGGtcttgaggcctgagc |
| NAE1 | V$EREF | Estrogen response elements | V$ESRRA.01 | Estrogen-related receptor alpha | 246 | 268 | 257 | (+) | | 0.873 |  | agacAAGGtaatatgggatatga |
| PSEN1 | V$EREF | Estrogen response elements | V$ESRRA.01 | Estrogen-related receptor alpha | 572 | 594 | 583 | (-) | | 0.994 | PSEN1 is cocited with V$EREF (TF: ESR1) | tatcAAGGtcataaaatcataat |
| PSEN2 | V$EREF | Estrogen response elements | V$ESRRA.01 | Estrogen-related receptor alpha | 472 | 494 | 483 | (+) | | 0.893 | PSEN2 is cocited with V$EREF (TF: ESR1) | gaacAAGGtccttgtgctccttt |
| ATF6 | V$EREF | Estrogen response elements | V$ESRRA.02 | Estrogen-related receptor alpha | 330 | 352 | 341 | (+) | | 0.949 |  | gggagaGGTCaattgaggtaaga |
| LRP1 | V$EREF | Estrogen response elements | V$ESRRA.01 | Estrogen-related receptor alpha | 441 | 463 | 452 | (-) | | 0.875 | LRP1 is cocited with V$EREF (TF: ESR1) |  |
| MAPT | V$EREF | Estrogen response elements | V$ERR.01 | Estrogen related receptor | 217 | 239 | 228 | (-) | | 0.894 | MAPT is cocited with V$EREF (TF: ESR1) | tgccAAGGccagcagagctcaca |
| ATP2A1 | V$EREF | Estrogen response elements | V$ESRRA.02 | Estrogen-related receptor alpha (secondary DNA binding preference) | 55 | 77 | 66 | (-) | | 0.925 | part of model match of EREF_SF1F_01 | ctcgggGGTCaggaacgggctcc |
| ATP2A3 | V$RXRF | RXR heterodimer binding sites | V$RAR_RXR.01 | Retinoic acid receptor / retinoid X receptor heterodimer, DR1 sites | 289 | 313 | 301 | (-) | | 0.866 |  | gaacccgggggtcagAGGTtgtagt |
| ATP2A2 | V$EREF | Estrogen response elements | V$ESRRA.01 | Estrogen-related receptor alpha | 212 | 234 | 223 | (-) | | 0.88 | ATP2A2 is cocited with V$EREF (TF: ESR2) | gaacAAGGttatatgcaagtact |
| CACNA1C | V$EREF | Estrogen response elements | V$ESRRA.01 | Estrogen-related receptor alpha | 367 | 389 | 378 | (-) | | 0.986 | part of model match of EREF_SF1F_01 | ttacAAGGtcatggggaaaattc |
| CACNA1F | V$EREF | Estrogen response elements | V$ESRRA.01 | Estrogen-related receptor alpha | 473 | 495 | 484 | (-) | | 0.887 |  | catcAAGGtgatgggggcccagg |
| CACNA1S | V$EREF | Estrogen response elements | V$ESRRA.02 | Estrogen-related receptor alpha (secondary DNA binding preference) | 312 | 334 | 323 | (-) | | 0.938 | part of model match of EREF_SF1F_01 | aacagaGGTCaggaataggtagc |
| CALM1 | V$EREF | Estrogen response elements | V$ESRRA.01 | Estrogen-related receptor alpha | 338 | 360 | 349 | (+) | | 0.873 | CALM1 interacts with V$EREF (TF: ESR1,ESR2,ESRRG)  CALM1 is cocited with V$EREF (TF: ESR1,ESR2,ESRRG) | tgacAAGGtaatccagcatctac |
| CALML6 | V$EREF | Estrogen response elements | V$ESRRB.01 | Estrogen-related receptor beta | 133 | 155 | 144 | (+) | | 0.952 | part of model match of EREF_SF1F_01 | gatgaAGGTcagggcctcccttg |
| CAPN2 | V$EREF | Estrogen response elements | V$ESRRA.02 | Estrogen-related receptor alpha (secondary DNA binding preference) | 332 | 354 | 343 | (+) | | 0.988 | CAPN2 is cocited with V$EREF (TF: ESR1) | agcaggGGTCagaaggacctcat |
| CASP7 | V$EREF | Estrogen response elements | V$ESRRA.02 | Estrogen-related receptor alpha (secondary DNA binding preference) | 441 | 463 | 452 | (+) | | 0.942 | part of model match of EREF_SF1F_01 | agatggGGTCatgcgtaagtttt |
| CDK5 | V$EREF | Estrogen response elements | V$ERR.01 | Estrogen related receptor | 519 | 541 | 530 | (+) | | 0.913 | CDK5 is cocited with V$EREF (TF: ESR1)  part of model match of NR2F_EREF_01 | gttcAAGGccaaaaaccgggaga |
| CDK5R1 | V$EREF | Estrogen response elements | V$ESRRA.02 | Estrogen-related receptor alpha (secondary DNA binding preference) | 262 | 284 | 273 | (-) | | 0.939 | part of model match of EREF_SF1F_01 | gggtggGGTCaccacatcgcctt |
| GSK3B | V$EREF | Estrogen response elements | V$ERR.01 | Estrogen related receptor | 250 | 272 | 261 | (+) | | 0.895 | GSK3B is cocited with V$EREF (TF: ESR1,ESR2) | tcacAAGGgcagagccattacct |
| EIF2AK3 | V$EREF | Estrogen response elements | V$ESRRA.01 | Estrogen-related receptor alpha | 124 | 146 | 135 | (+) | | 0.894 | part of model match of PARF_EREF_01 | tcacAAGGtcctttcctcataga |
| IL1B | V$EREF | Estrogen response elements | V$ERR.01 | Estrogen related receptor | 75 | 97 | 86 | (-) | | 0.932 | IL1B is cocited with V$EREF (TF: ESR1,ESR2,ESRRA) | actcAAGGgcacacagcaggtta |
| ITPR1 | V$EREF | Estrogen response elements | V$ESRRA.01 | Estrogen-related receptor alpha | 118 | 140 | 129 | (-) | | 0.888 | ITPR1 is cocited with V$EREF (TF: ESR1) | agacAAGGtctcgttctgccacc |
| ITPR2 | V$EREF | Estrogen response elements | V$ESRRA.01 | Estrogen-related receptor alpha | 527 | 549 | 538 | (-) | | 0.886 |  | tggcAAGGtccccggcctctggg |
| ITPR3 | V$EREF | Estrogen response elements | V$ESRRA.02 | Estrogen-related receptor alpha (secondary DNA binding preference) | 535 | 557 | 546 | (-) | | 0.921 | part of model match of NR2F_EREF_01 | gccagtGGTCaccatccctagcc |
| MAPK1 | V$EREF | Estrogen response elements | V$ESRRB.01 | Estrogen-related receptor beta | 557 | 579 | 568 | (+) | | 0.993 | MAPK1 is cocited with V$EREF (TF: ESR1,ESR2,ESRRA) | tctcaAGGTcagccaggttattt |
| MAPK3 | V$EREF | Estrogen response elements | V$ESRRA.02 | Estrogen-related receptor alpha (secondary DNA binding preference) | 326 | 348 | 337 | (-) | | 0.991 | MAPK3 is cocited with V$EREF (TF: ESR1,ESR2) | gtcaggGGTCacagggaagactg |
| PLCB1 | V$EREF | Estrogen response elements | V$ESRRA.02 | Estrogen-related receptor alpha (secondary DNA binding preference) | 183 | 205 | 194 | (+) | | 0.989 | part of model match of EREF_SF1F_01,NR2F_EREF_01 | gtcaggGGTCagggacccacttg |
| PLCB2 | V$EREF | Estrogen response elements | V$ESRRA.02 | Estrogen-related receptor alpha (secondary DNA binding preference) | 194 | 216 | 205 | (+) | | 0.937 | PLCB2 is cocited with V$EREF (TF: ESR1) | ctttggGGTCagatgcccatgct |
| PLCB3 | V$EREF | Estrogen response elements | V$ESRRA.02 | Estrogen-related receptor alpha (secondary DNA binding preference) | 454 | 476 | 465 | (+) | | 0.933 |  | aagtggGGTCagccatcagctta |
| RYR3 | V$EREF | Estrogen response elements | V$ESRRA.02 | Estrogen-related receptor alpha (secondary DNA binding preference) | 131 | 153 | 142 | (-) | | 0.994 |  | ctgaggGGTCaacttgagtattt |

**Supplementary Table S4:** Functional enrichment and annotation analysis GNAQ, ATP2A2, and PLCB4 by ToppGene (ToppFun)

**GO: Biological Process 305 annotations before applied cutoff / 18482 genes in category**

**ID	Name	pValue	FDR B&H	FDR B&Y	Bonferroni	Genes from Input	Genes in Annotation**

1	GO:0043267	negative regulation of K+ ion transport	3.686E-6	1.124E-3	7.082E-3	1.124E-3	2	21

2	GO:0043266	regulation of potassium ion transport	2.898E-5	4.419E-3	2.784E-2	8.838E-3	2	58

3	GO:0043271	negative regulation of ion transport	6.252E-5	5.911E-3	3.723E-2	1.907E-2	2	85

4	GO:0030001	metal ion transport	7.752E-5	5.911E-3	3.723E-2	2.364E-2	3	789

5	GO:0006812	cation transport	1.676E-4	1.023E-2	6.441E-2	5.113E-2	3	1020

**Pathway 134 annotations before applied cutoff / 10724 genes in category**

**ID	Name	Source	pValue	FDR B&H	FDR B&Y	Bonferroni	Genes from Input	Genes in Annotation**

1	672448	Alzheimers Disease	BioSystems: WikiPathways	3.848E-7	4.659E-5	2.552E-4	5.157E-5	3	79

2	169306	Pancreatic secretion	BioSystems: KEGG	6.953E-7	4.659E-5	2.552E-4	9.317E-5	3	96

3	908257	Adrenergic signaling in cardiomyocytes	BioSystems: KEGG	2.629E-6	1.174E-4	6.434E-4	3.523E-4	3	149

4	83097	Alzheimer's disease	BioSystems: KEGG	3.777E-6	1.265E-4	6.933E-4	5.062E-4	3	168

5	83050	Calcium signaling pathway	BioSystems: KEGG	4.730E-6	1.268E-4	6.945E-4	6.338E-4	3	181

6	P00041	Metabotropic glutamate receptor group I pathway	PantherDB	1.438E-5	3.212E-4	1.760E-3	1.927E-3	2	24

**Supplementary Table S5:** Functional enrichment and annotation analysis 37 ESR1 target genes (table S3) by ToppGene (ToppFun)

**GO: Biological Process 2059 annotations before applied cutoff / 18482 genes in category**

**ID	Name	pValue	FDR B&H	FDR B&Y	Bonferroni	Genes from Input	Genes in Annotation**

1	GO:0070588	calcium ion transmembrane transport	9.285E-15	1.912E-11	1.569E-10	1.912E-11	11	169

2	GO:0043085	positive regulation of catalytic activity	2.461E-14	2.534E-11	2.080E-10	5.068E-11	20	1371

3	GO:0007169	receptor protein tyrosine kinase signaling pathway	5.522E-14	3.790E-11	3.111E-10	1.137E-10	16	726

26	GO:0051402	neuron apoptotic process	3.659E-10	2.791E-8	2.290E-7	7.535E-7	9	233

50	GO:0007611	learning or memory	6.801E-9	2.801E-7	2.299E-6	1.400E-5	8	222

**Pathway 859 annotations before applied cutoff / 10724 genes in category**

**ID	Name	Source	pValue	FDR B&H	FDR B&Y	Bonferroni	Genes from Input	Genes in Annotation**

1	672448	Alzheimers Disease	BioSystems: WikiPathways	2.035E-76	1.748E-73	1.282E-72	1.748E-73	35	79

2	83097	Alzheimer's disease	BioSystems: KEGG	2.389E-69	1.026E-66	7.526E-66	2.052E-66	37	168

3	83091	GnRH signaling pathway	BioSystems: KEGG	1.705E-18	4.882E-16	3.580E-15	1.465E-15	13	92

4	83050	Calcium signaling pathway	BioSystems: KEGG	9.698E-18	2.083E-15	1.527E-14	8.331E-15	15	181

5	96530	Vascular smooth muscle contraction	BioSystems: KEGG	7.051E-17	1.211E-14	8.884E-14	6.057E-14	13	121

6	P00003	Alzheimer disease-amyloid secretase pathway	PantherDB	1.262E-16	1.806E-14	1.325E-13	1.084E-13	11	65

7	83085	Long-term potentiation	BioSystems: KEGG	1.803E-16	2.213E-14	1.623E-13	1.549E-13	11	67

8	525336	Serotonergic synapse	BioSystems: KEGG	1.709E-15	1.835E-13	1.346E-12	1.468E-12	12	114

**Supplementary Table S6:** BARHL1-ESR1 and AD pathway genes along with Protein-protein interaction derived shortest pathway linkers and their AD related functions.

| **BARHL1-ESR1 and AD pathway genes along with shortest pathway linkers and their AD related functions** | | | | | | |
| --- | --- | --- | --- | --- | --- | --- |
| **BARHL1-ESR1 genes (9)** | **All AD Genes from KEGG** | **20 Key AD genes from KEGG** | **Key linker genes (5)** | **41 Linker genes in shortest pathways between AD and BARHL1 net work** | **AD specific Alterations and Functions of shortest pathway linker genes** | **References** |
| ATOH1 | ADAM10 | ADAM10 | HMGB1 | ACTN1 | Aberrant expression may correlate with AD | PMID: 22982105 |
| BARHL1 | ADAM17 | APBB1 | SUMO1 | AR | Short CAG repeat polymorphism is associated with AD in men | PMID: 12668243 |
| BDNF | APAF1 | APH1A | SUMO2 | BCL2L1 | Upregulated in AD hippocampus | PMID: 17712163 |
| ESR1 | APH1A | APOE | TLE2 | CTNNB1 | Aberrant beta-catenin signaling correlates with neurodegradation in AD | PMID: 19812311; 23218566 |
| MED7 | APOE | APP | UBC | ELAVL1 | Downregulated the AD hippocampusm, inhibits ADAM10 protein expression | PMID:19221430 |
| NTF3 | APP | BACE1 |  | FHL2 | Interacts with Alzheimer's disease-associated PS2 | PMID: 11001931 |
| TH | ATF6 | CACNA1C |  | GPS2 |  |  |
| THRB | ATP2A1 | CASP7 |  | GRB2 | Overexpressed and binds to amyloid beta precursor protein and may play a pathological role | PMID: 20164575; 15054097 |
| TLE1 | ATP2A2 | GAPDH |  | HDAC2 | Induced inhibition completely rescue memory deficits in mouse model of AD | PMID: 20010553 |
|  | ATP2A3 | GRIN1 |  | HDAC3 | Upregulated and provides toxicity through GSK3β. Induced inhibition completely rescue memory deficits in mouse model of AD | PMID: 20010553 |
|  | ATP5A1 | GSK3B |  | HES1 | Protects nerve cells from the toxic effects of amyloid beta precursor protein | PMID: 22466002 |
|  | ATP5B | IDE |  | HGS | SNP increases risk of AD | PMID: 24439028 |
|  | ATP5C1 | LPL |  | **HMGB1** | **Upregulated AD and mediates memory impairement through TLR4 or RAGE** | **PMID:21884699; 22645697** |
|  | ATP5D | LRP1 |  | HRB |  |  |
|  | ATP5E | MME |  | HSPA4 | Overexpression lower levels of Aβ, Aβ plaque deposition, and neuronal and synaptic loss | PMID: 21471357 |
|  | ATP5F1 | NCSTN |  | HSPD1 | Mediates translocation of APP and beta-amyloid to the mitochondria | PMID: 22753410 |
|  | ATP5G1 | PSEN1 |  | LIG3 |  |  |
|  | ATP5G2 | PSEN2 |  | MAPK1 | Upregulated in late stage of AD and may play a role in the pathogenesis | PMID: 24334724 |
|  | ATP5G3 | PSENEN |  | MAPK3 |  |  |
|  | ATP5H | SNCA |  | MED1 |  |  |
|  | ATP5J |  |  | MED21 |  |  |
|  | ATP5O |  |  | MED25 |  |  |
|  | ATP6 |  |  | NTRK1 | rs6336 on NTRK1 is associated with early-onset of AD | PMID: 18780967 |
|  | ATP8 |  |  | NTRK2 | SNPs in NTRK2 are associated with the increased risk of AD | PMID: 17918233; 18780967 |
|  | BACE1 |  |  | PARP1 | Increased activity and expression correlats with AD. Genetic variants are associated with increased risk of AD | PMID: 22430645; 20486200; 17290104 |
|  | BACE2 |  |  | PDCD4 | May be associated with AD | PMID: 21163940 |
|  | BAD |  |  | PRKACA |  |  |
|  | BID |  |  | PTF1A |  |  |
|  | CACNA1C |  |  | PTPRA |  |  |
|  | CACNA1D |  |  | RARA | Amyloid beta (Aβ) down-regulates RARα signalling and thereby blocks inhibits retinoic acid synthesis and exarts its aetiopathology of AD | PMID: 23379615 |
|  | CACNA1F |  |  | SHC1 |  |  |
|  | CACNA1S |  |  | SRC | Regulates APP endocytic sorting pathway and thus modulates Aβ secretion. May regulate amyloid dependent microgliosis. deregulated Src-dependent signaling pathways may be involved in early stages of AD. SRC isoform FYN is upregulated in AD and is a potential target. | PMID: 22787047; 22673542; 25128699; 24495408 |
|  | CALM1 |  |  | **SUMO1** | **SUMO1 regulatesf BACE1 accumulation and Aβ generation. Covalently modifies lysines 587 and 595 of the APP protein and thereby negatively regulates levels of Aβ aggregates. Can be a potential therapeutic in AD.** | **PMID: 22975420; 18675254** |
|  | CALM2 |  |  | **SUMO2** | **Covalently modifies lysines 587 and 595 of the APP protein and thereby negatively regulates levels of Aβ aggregates.** | **PMID: 18675254** |
|  | CALM3 |  |  | TCF12 |  |  |
|  | CALML3 |  |  | TLE2 |  |  |
|  | CALML5 |  |  | TRIP13 |  |  |
|  | CALML6 |  |  | TTRAP/ TDP2 |  |  |
|  | CAPN1 |  |  | **UBC** | **Reduced ubiquitin proteasome degradation activity is associated with AD** | **PMID: 24008896** |
|  | CAPN2 |  |  | UBQLN1 | Genetic variants increase the risk of AD | PMID: 15745979 |
|  | CASP12 |  |  | VCAM1 | Increased level in blood correlates with AD | PMID: 23403534; 18597785 |
|  | CASP3 |  |  |  |  |  |
|  | CASP7 |  |  |  |  |  |
|  | CASP8 |  |  |  |  |  |
|  | CASP9 |  |  |  |  |  |
|  | CDK5 |  |  |  |  |  |
|  | CDK5R1 |  |  |  |  |  |
|  | CHP1 |  |  |  |  |  |
|  | CHP2 |  |  |  |  |  |
|  | COX1 |  |  |  |  |  |
|  | COX2 |  |  |  |  |  |
|  | COX3 |  |  |  |  |  |
|  | COX4I1 |  |  |  |  |  |
|  | COX4I2 |  |  |  |  |  |
|  | COX5A |  |  |  |  |  |
|  | COX5B |  |  |  |  |  |
|  | COX6A1 |  |  |  |  |  |
|  | COX6A2 |  |  |  |  |  |
|  | COX6B1 |  |  |  |  |  |
|  | COX6B2 |  |  |  |  |  |
|  | COX6C |  |  |  |  |  |
|  | COX7A1 |  |  |  |  |  |
|  | COX7A2 |  |  |  |  |  |
|  | COX7A2L |  |  |  |  |  |
|  | COX7B |  |  |  |  |  |
|  | COX7B2 |  |  |  |  |  |
|  | COX7C |  |  |  |  |  |
|  | COX8A |  |  |  |  |  |
|  | COX8C |  |  |  |  |  |
|  | CYC1 |  |  |  |  |  |
|  | CYCS |  |  |  |  |  |
|  | CYTB |  |  |  |  |  |
|  | EIF2AK3 |  |  |  |  |  |
|  | ERN1 |  |  |  |  |  |
|  | FADD |  |  |  |  |  |
|  | FAS |  |  |  |  |  |
|  | GAPDH |  |  |  |  |  |
|  | GNAQ |  |  |  |  |  |
|  | GRIN1 |  |  |  |  |  |
|  | GRIN2A |  |  |  |  |  |
|  | GRIN2B |  |  |  |  |  |
|  | GRIN2C |  |  |  |  |  |
|  | GRIN2D |  |  |  |  |  |
|  | GSK3B |  |  |  |  |  |
|  | IDE |  |  |  |  |  |
|  | IL1B |  |  |  |  |  |
|  | ITPR1 |  |  |  |  |  |
|  | ITPR2 |  |  |  |  |  |
|  | ITPR3 |  |  |  |  |  |
|  | LPL |  |  |  |  |  |
|  | LRP1 |  |  |  |  |  |
|  | MAPK1 |  |  |  |  |  |
|  | MAPK3 |  |  |  |  |  |
|  | MAPT |  |  |  |  |  |
|  | MME |  |  |  |  |  |
|  | NAE1 |  |  |  |  |  |
|  | NCSTN |  |  |  |  |  |
|  | NDUFA1 |  |  |  |  |  |
|  | NDUFA10 |  |  |  |  |  |
|  | NDUFA11 |  |  |  |  |  |
|  | NDUFA12 |  |  |  |  |  |
|  | NDUFA13 |  |  |  |  |  |
|  | NDUFA2 |  |  |  |  |  |
|  | NDUFA3 |  |  |  |  |  |
|  | NDUFA4 |  |  |  |  |  |
|  | NDUFA4L2 |  |  |  |  |  |
|  | NDUFA5 |  |  |  |  |  |
|  | NDUFA6 |  |  |  |  |  |
|  | NDUFA7 |  |  |  |  |  |
|  | NDUFA8 |  |  |  |  |  |
|  | NDUFA9 |  |  |  |  |  |
|  | NDUFAB1 |  |  |  |  |  |
|  | NDUFB1 |  |  |  |  |  |
|  | NDUFB10 |  |  |  |  |  |
|  | NDUFB11 |  |  |  |  |  |
|  | NDUFB2 |  |  |  |  |  |
|  | NDUFB3 |  |  |  |  |  |
|  | NDUFB4 |  |  |  |  |  |
|  | NDUFB5 |  |  |  |  |  |
|  | NDUFB6 |  |  |  |  |  |
|  | NDUFB7 |  |  |  |  |  |
|  | NDUFB8 |  |  |  |  |  |
|  | NDUFB9 |  |  |  |  |  |
|  | NDUFC1 |  |  |  |  |  |
|  | NDUFC2 |  |  |  |  |  |
|  | NDUFC2 |  |  |  |  |  |
|  | NDUFS1 |  |  |  |  |  |
|  | NDUFS2 |  |  |  |  |  |
|  | NDUFS3 |  |  |  |  |  |
|  | NDUFS4 |  |  |  |  |  |
|  | NDUFS5 |  |  |  |  |  |
|  | NDUFS6 |  |  |  |  |  |
|  | NDUFS7 |  |  |  |  |  |
|  | NDUFS8 |  |  |  |  |  |
|  | NDUFV1 |  |  |  |  |  |
|  | NDUFV2 |  |  |  |  |  |
|  | NDUFV3 |  |  |  |  |  |
|  | NOS1 |  |  |  |  |  |
|  | PLCB1 |  |  |  |  |  |
|  | PLCB2 |  |  |  |  |  |
|  | PLCB3 |  |  |  |  |  |
|  | PLCB4 |  |  |  |  |  |
|  | PPP3CA |  |  |  |  |  |
|  | PPP3CB |  |  |  |  |  |
|  | PPP3CC |  |  |  |  |  |
|  | PPP3R1 |  |  |  |  |  |
|  | PPP3R2 |  |  |  |  |  |
|  | PSEN1 |  |  |  |  |  |
|  | PSEN2 |  |  |  |  |  |
|  | PSENEN |  |  |  |  |  |
|  | RYR3 |  |  |  |  |  |
|  | SDHA |  |  |  |  |  |
|  | SDHB |  |  |  |  |  |
|  | SDHC |  |  |  |  |  |
|  | SDHD |  |  |  |  |  |
|  | SNCA |  |  |  |  |  |
|  | TNF |  |  |  |  |  |
|  | TNFRSF1A |  |  |  |  |  |
|  | UQCR10 |  |  |  |  |  |
|  | UQCR11 |  |  |  |  |  |
|  | UQCRB |  |  |  |  |  |
|  | UQCRC1 |  |  |  |  |  |
|  | UQCRC2 |  |  |  |  |  |
|  | UQCRFS1 |  |  |  |  |  |
|  | UQCRH |  |  |  |  |  |
|  | UQCRHL |  |  |  |  |  |
|  | UQCRQ |  |  |  |  |  |

**Supplementary Table S7:** Functional enrichment of BARHL1 network (9 genes) by ToppFun.

**GO: Biological Process 877 annotations before applied cutoff / 18482 genes in category**

**ID	Name	pValue	FDR B&H	FDR B&Y	Bonferroni	Genes from Input	Genes in Annotation**

1	GO:0060548	negative regulation of cell death	5.218E-7	2.748E-4	2.021E-3	4.576E-4	6	810

2	GO:1901215	negative regulation of neuron death	8.598E-7	2.748E-4	2.021E-3	7.541E-4	4	171

3	GO:0009887	organ morphogenesis	1.105E-6	2.748E-4	2.021E-3	9.691E-4	6	920

5	GO:0043523	regulation of neuron apoptotic process	1.915E-6	3.359E-4	2.471E-3	1.680E-3	4	209

6	GO:0006366	transcription from RNA polymerase II promoter	2.451E-6	3.583E-4	2.635E-3	2.150E-3	7	1797

7	GO:0051402	neuron apoptotic process	2.952E-6	3.593E-4	2.642E-3	2.589E-3	4	233

8	GO:0042490	mechanoreceptor differentiation	3.278E-6	3.593E-4	2.642E-3	2.874E-3	3	64

9	GO:1901214	regulation of neuron death	3.905E-6	3.805E-4	2.798E-3	3.424E-3	4	250

10	GO:0070997	neuron death	6.748E-6	5.918E-4	4.352E-3	5.918E-3	4	287

11	GO:0010628	positive regulation of gene expression	9.631E-6	7.273E-4	5.349E-3	8.446E-3	6	1332

12	GO:0043066	negative regulation of apoptotic process	1.089E-5	7.273E-4	5.349E-3	9.554E-3	5	736

13	GO:0042981	regulation of apoptotic process	1.163E-5	7.273E-4	5.349E-3	1.020E-2	6	1376

14	GO:0043069	negative regulation of programmed cell death	1.186E-5	7.273E-4	5.349E-3	1.041E-2	5	749

15	GO:0043067	regulation of programmed cell death	1.244E-5	7.273E-4	5.349E-3	1.091E-2	6	1392

16	GO:0010941	regulation of cell death	1.680E-5	9.207E-4	6.771E-3	1.473E-2	6	1466

20	GO:0007605	sensory perception of sound	3.998E-5	1.753E-3	1.289E-2	3.507E-2	3	147

22	GO:0050954	sensory perception of mechanical stimulus	5.848E-5	2.314E-3	1.702E-2	5.129E-2	3	167

25	GO:0050793	regulation of developmental process	7.750E-5	2.685E-3	1.975E-2	6.796E-2	6	1912

35	GO:0007420	brain development	1.619E-4	3.974E-3	2.923E-2	1.420E-1	4	646

38	GO:0007610	behavior	2.054E-4	4.635E-3	3.409E-2	1.801E-1	4	687

**Disease 103 annotations before applied cutoff / 8028 genes in category**

**ID	Name	Source	pValue	FDR B&H	FDR B&Y	Bonferroni	Genes from Input	Genes in Annotation**

1	ctd:D004830	Epilepsy, Tonic-Clonic	CTD	2.089E-5	2.152E-3	1.123E-2	2.152E-3	2	10

2	ctd:D009410	Nerve Degeneration	CTD	4.958E-4	4.052E-3	2.114E-2	5.106E-2	47

26	ctd:D000544	Alzheimer Disease	CTD	1.542E-3	6.109E-3	3.187E-2	1.588E-1	2	83

33	ctd:D008607	Intellectual Disability	CTD	3.518E-3	1.098E-2	5.728E-2	3.623E-1	2	126

47	ctd:D012559	Schizophrenia	CTD	6.765E-3	1.483E-2	7.734E-2	6.968E-1	2	176

49	ctd:D011618	Psychotic Disorders	CTD	9.680E-3	1.955E-2	1.020E-1	9.970E-1	1	13

**Supplementary Table S8**: Protein-protein interactions (PPI) based shortest pathway and key node analysis between BARHL1-ESR1 and AD pathway key proteins.

BARHL1-TLE1-GSK3B-APP

**BARHL1-MED7-HGS-APP**

**BARHL1-MED7-UBC-APP**

APP-GAPDH-ESR1

APP-MYH9-ESR1

APP-HSPA8-ESR1

APP-HSPA4-ESR1

APP-SHC1-ESR1

APP-CAV1-ESR1

APP-HSPD1-ESR1

APP-HTATIP-ESR1

APP-GRB2-ESR1

APP-ACTB-ESR1

APP-STUB1-ESR1

**APP-UBC-ESR1**

APP-SPTAN1-ESR1

APP-SUMO1-ESR1

APP-HSP90AA1-ESR1

APP-SHC1-NTRK3-NTF3

APP-SHC1-NTRK2-NTF3

APP-**UBC**-NTRK2-NTF3

APP-HSPA4-NTRK1-NTF3

APP-SHC1-NTRK1-NTF3

APP-CAV1-NTRK1-NTF3

APP-GRB2-NTRK1-NTF3

APP-CDC37-NTRK1-NTF3

APP-ABL1-NTRK1-NTF3

APP-**UBC**-NTRK1-NTF3

APP-HSP90AA1-NTRK1-NTF3

APP-GAPDH-ESR1-BDNF

APP-MYH9-ESR1-BDNF

APP-HSPA8-ESR1-BDNF

APP-HSPA4-ESR1-BDNF

APP-SHC1-ESR1-BDNF

APP-CAV1-ESR1-BDNF

APP-HSPD1-ESR1-BDNF

APP-HTATIP-ESR1-BDNF

APP-GRB2-ESR1-BDNF

APP-ACTB-ESR1-BDNF

APP-STUB1-ESR1-BDNF

**APP-UBC-ESR1-BDNF**

APP-SPTAN1-ESR1-BDNF

APP-SUMO1-ESR1-BDNF

APP-HSP90AA1-ESR1-BDNF

APP-YWHAG-ELAVL1-BDNF

APP-PPIA-ELAVL1-BDNF

APP-CASP3-ELAVL1-BDNF

APP-NCSTN-ELAVL1-BDNF

APP-CALR-ELAVL1-BDNF

APP-DLG4-ELAVL1-BDNF

APP-GRB2-ELAVL1-BDNF

APP-GSK3B-ELAVL1-BDNF

APP-ACTB-ELAVL1-BDNF

APP-**UBC**-ELAVL1-BDNF

APP-SUMO1-ELAVL1-BDNF

APP-SET-ELAVL1-BDNF

APP-SHC1-NTRK2-BDNF

APP-**UBC**-NTRK2-BDNF

ATOH1-HMGB1-CASP3-APP

ATOH1-HMGB1-PDIA3-APP

ATOH1-HMGB1-HSPA8-APP

APOE-**UBC**-NTRK2-NTF3

APOE-**UBC**-NTRK1-NTF3

APOE-CDC37-NTRK1-NTF3

APOE-**UBC**-NTRK2

APOE-**UBC**-NTRK1

APOE-CDC37-NTRK1

APOE-CDC37

APOE-**UBC**-HMGB1-ATOH1

APOE-VCAM1-HMGB1-ATOH1

APOE-**UBC**-EIF3I-THRB

APOE-VCAM1-EIF3I-THRB

APOE-**UBC**-C1D-THRB

APOE-**UBC**-PPARGC1A-THRB

APOE-**UBC**-FOXO1-THRB

APOE-**UBC**-NRIP1-THRB

APOE-**UBC**-ZNHIT3-THRB

APOE-**UBC**-PARP1-THRB

APOE-VCAM1-PARP1-THRB

APOE-**UBC**-MPG-THRB

APOE-PRNP-MPG-THRB

APOE-**UBC**-RARA-THRB

APOE-**UBC**-PSMC5-THRB

APOE-VCAM1-PSMC5-THRB

APOE-**UBC**-SUMO2-THRB

APOE-ST13-SUMO2-THRB

APOE-PDCD4-SUMO2-THRB

APOE-**UBC**-SUMO1-THRB

APOE-APP-SUMO1-THRB

**APOE-UBC-ESR1-THRB**

APOE-SCARB1-PRMT2-THRB

APOE-**UBC**-PRMT1-THRB

APOE-VCAM1-PRMT1-THRB

APOE-CDC37-PRMT1-THRB

APOE-**UBC**-TRIP6-THRB

APOE-**UBC**-TRIP4-THRB

APOE-**UBC**-GPS2-THRB

APOE-**UBC**-HMGN3-THRB

APOE-**UBC**-SMC1A-THRB

APOE-**UBC**-BRD8-THRB

APOE-**UBC**-SRC-THRB

APOE-MAP2-SRC-THRB

APOE-CDC37-SRC-THRB

APOE-LRP1-SRC-THRB

APOE-**UBC**-JMJD1C-THRB

APOE-**UBC**-CCND1-THRB

APOE-**UBC**-NCOR2-THRB

APOE-**UBC**-NCOR1-THRB

APOE-**UBC**-HSPA4-THRB

APOE-PRNP-HSPA4-THRB

APOE-APP-HSPA4-THRB

APOE-MAPT-HSPA4-THRB

APOE-HP-TBL1X-THRB

APOE-**UBC**-TSG101-THRB

APOE-**UBC**-TP53-THRB

APOE-**UBC**-CAMK2B-THRB

APOE-**UBC**-CTCF-THRB

APOE-**UBC**-TBL1XR1-THRB

APOE-**UBC**-XRCC6-THRB

APOE-VCAM1-XRCC6-THRB

APOE-**UBC**-XRCC5-THRB

APOE-VCAM1-XRCC5-THRB

APOE-**UBC**-NR2F6-THRB

APOE-**UBC**-RXRB-THRB

APOE-**UBC**-NCOA3-THRB

APOE-ALB-NCOA3-THRB

APOE-**UBC**-NCOA2-THRB

APOE-**UBC**-NCOA1-THRB

APOE-**UBC**-MED25-THRB

APOE-**UBC**-MED21-THRB

APOE-**UBC**-PRKDC-THRB

APOE-VCAM1-PRKDC-THRB

APOE-**UBC**-LIG3-THRB

APOE-**UBC**-C1ORF103-THRB

APOE-**UBC**-TRIP13-THRB

APOE-LOXL4-TRIP13-THRB

APOE-**UBC**-TRIP12-THRB

APOE-**UBC**-TRIP10-THRB

APOE-**UBC**-HDAC3-THRB

APOE-**UBC**-HDAC2-THRB

APOE-**UBC**-COPS2-THRB

APOE-**UBC**-CNOT1-THRB

APOE-VCAM1-CNOT1-THRB

APOE-ALB-CNOT1-THRB

APOE-**UBC**-MED1-THRB

IDE-**UBC**-DDR1

IDE-NR3C1-NTRK2-NTF3

IDE-**UBC**-NTRK2-NTF3

IDE-**UBC**-NTRK1-NTF3f

NTF3-NTRK1-**UBC**-IDE

LPL-**UBC**-MED7-BARHL1

**LPL-UBC-ESR1**

LPL-RPL18A-ESR1

LPL-CCND1-ESR1

LPL-CUL3-ESR1

LPL-EMD-ESR1

LPL-**UBC**-NTRK2-NTF3

LPL-**UBC**-NTRK1-NTF3

**LPL-UBC-ESR1-BDNF**

LPL-RPL18A-ESR1-BDNF

LPL-CCND1-ESR1-BDNF

LPL-CUL3-ESR1-BDNF

LPL-EMD-ESR1-BDNF

LPL-CALR-ELAVL1-BDNF

LPL-**UBC**-ELAVL1-BDNF

LPL-CCND1-ELAVL1-BDNF

LPL-CUL3-ELAVL1-BDNF

LPL-LUC7L2-ELAVL1-BDNF

LPL-**UBC**-NTRK2-BDNF

LPL-**UBC**-HMGB1-ATOH1

LPL-**UBC**-TCF12-PTF1A

LPL-CCND1-THRB

LPL-**UBC**-RPS6KA5-TH

LPL-**UBC**-CSNK2A1-TH

LPL-**UBC**-MAPK3-TH

LPL-**UBC**-MAPK1-TH

LPL-CCND1-MAPK1-TH

LPL-**UBC**-CAMK2G-TH

LPL-CUL3-CAMK2G-TH

LPL-**UBC**-PRKACA-TH

LPL-CCND1-PRKACA-TH

LPL-CUL3-PRKACA-TH

LPL-LRP1-PRKACA-TH

LPL-CUL3-MAPKAPK2-TH

LPL-CALR-YWHAZ-TH

LPL-**UBC**-YWHAZ-TH

LPL-KIAA1377-YWHAZ-TH

LPL-CUL3-YWHAZ-TH

LPL-LUC7L2-YWHAZ-TH

LPL-**UBC**-SNCA-TH

NCSTN-APH1A-TLE1-BARHL1

NCSTN-**UBC**-MED7-BARHL1

**NCSTN-UBC-ESR1**

**BACE1-UBC-ESR1**

BACE1-HNF4A-ESR1

SNCA-GAPDH-ESR1

SNCA-HSPA1A-ESR1

SNCA-HSPA8-ESR1

SNCA-MAPK3-ESR1

SNCA-HSPA4-ESR1

SNCA-MAPK1-ESR1

SNCA-CLTC-ESR1

SNCA-STUB1-ESR1

SNCA-CALM1-ESR1

**SNCA-UBC-ESR1**

SNCA-GSK3B-TLE1-BARHL1

SNCA-BCL2L1-TLE1-BARHL1

SNCA-**UBC**-MED7-BARHL1

SNCA-SUMO2-MED7-BARHL1fv

BARHL1-TLE1-GSK3B-SNCA

BARHL1-TLE1-BCL2L1-SNCA

**BARHL1-MED7-UBC-SNCA**

**BARHL1-MED7-SUMO2-SNCA**

**BARHL1-MED7-UBC-PSEN2**

ESR1-ACTN1-PSEN1

**ESR1-UBC-PSEN2**

ESR1-CTNNB1-PSEN2

ESR1-FHL2-PSEN2

**BARHL1-MED7 -ESR1**

GAPDH-RPA2-TLE1-BARHL1

GAPDH-PAFAH1B3-TLE1-BARHL1

GAPDH-SMN2-TLE1-BARHL1

GAPDH-SMN1-TLE1-BARHL1

GAPDH-GADD45A-TLE1-BARHL1

GAPDH-CDKN1A-TLE1-BARHL1

GAPDH-TK1-TLE1-BARHL1

GAPDH-HES1-TLE1-BARHL1

GAPDH-**UBC**-MED7-BARHL1

GAPDH-HNF4A-MED7-BARHL1

GAPDH-ESR1-MED7-BARHL1

APH1A-TLE1-BARHL1

THRB-SUMO2-TTRAP-PSEN1

THRB-SUMO1-TTRAP-PSEN1

THRB-PRMT1-CDC37-PSEN1

THRB-SRC-CDC37-PSEN1

THRB-RARA-UBQLN1-PSEN1

THRB-RXRA-GSK3B-PSEN1

THRB-NCOA3-GSK3B-PSEN1

THRB-PPARGC1A-GSK3B-PSEN1

THRB-HSPA4-GSK3B-PSEN1

THRB-TP53-GSK3B-PSEN1

THRB-CCND1-GSK3B-PSEN1

THRB-FOXO1-GSK3B-PSEN1

THRB-XRCC6-NOTCH1-PSEN1

THRB-HSPA4-APP-PSEN1

THRB-SUMO1-APP-PSEN1

THRB-TP53-BCL2L1-PSEN1

THRB-TP53-GAPDH-PSEN1

THRB-ESR1-GAPDH-PSEN1

THRB-CAMK2B-GAPDH-PSEN1

THRB-SUMO1-GAPDH-PSEN1

THRB-TP53-CDK5-PSEN1

THRB-SRC-CDK5-PSEN1

THRB-SUMO1-CASP8-PSEN1

THRB-PARP1-CASP7-PSEN1

THRB-SMC1A-CASP4-PSEN1

THRB-TP53>BCL2-PSEN1

THRB-PARP1-BCL2-PSEN1

THRB-PRKDC-CASP3-PSEN1

THRB-PARP1-CASP3-PSEN1

THRB-PARP1-CASP1-PSEN1

THRB-HSPA4-MAPT-PSEN1

THRB-RXRA-PIK3R1-PSEN1

THRB-LIG3-PIK3R1-PSEN1

THRB-HDAC3-PIK3R1-PSEN1

THRB-ESR1-PIK3R1-PSEN1

THRB-SRC-PIK3R1-PSEN1

THRB-RXRA-CTNNB1-PSEN1

THRB-TRIP10-CTNNB1-PSEN1

THRB-NCOA2-CTNNB1-PSEN1

THRB-HDAC2-CTNNB1-PSEN1

THRB-TBL1X-CTNNB1-PSEN1

THRB-ESR1-CTNNB1-PSEN1

THRB-FOXO1-CTNNB1-PSEN1

THRB-SRC-CTNNB1-PSEN1

THRB-SUMO1-CTNNB1-PSEN1

THRB-ESR1-ACTN1-PSEN1

THRB-SRC-ACTN1-PSEN1

THRB-SUMO1-ACTN1-PSEN1

THRB-NCOA3-PRKCZ-PSEN1

THRB-ESR1-PRKCZ-PSEN1

THRB-SRC-PRKCZ-PSEN1

THRB-RXRB-**UBC**-PSEN1

THRB-LIG3-**UBC**-PSEN1

THRB-TRIP13-**UBC**-PSEN1

THRB-TRIP12-**UBC**-PSEN1

THRB-NR2F6-**UBC**-PSEN1

THRB-TBL1XR1-**UBC**-PSEN1

THRB-TRIP10-**UBC**-PSEN1

THRB-CTCF-**UBC**-PSEN1

THRB-PRKDC-**UBC**-PSEN1

THRB-COPS2-**UBC**-PSEN1

THRB-MPG-**UBC**-PSEN1

THRB-NRIP1-**UBC**-PSEN1

THRB-ZNHIT3-**UBC**-PSEN1

THRB-NCOA3-**UBC**-PSEN1

THRB-PPARGC1A-**UBC**-PSEN1

THRB-NCOA2-**UBC**-PSEN1

THRB-GPS2-**UBC**-PSEN1

THRB-NCOA1-**UBC**-PSEN1

THRB-HDAC3-**UBC**-PSEN1

THRB-HDAC2-**UBC**-PSEN1

THRB-HSPA4-**UBC**-PSEN1

THRB-TP53-**UBC**-PSEN1

THRB-XRCC6-**UBC**-PSEN1

THRB-XRCC5-**UBC**-PSEN1

THRB-CCND1-**UBC**-PSEN1

**THRB-ESR1-UBC-PSEN1**

THRB-SMC1A-**UBC**-PSEN1

THRB-MED25-**UBC**-PSEN1

THRB-C1D-**UBC**-PSEN1

THRB-TRIP6-**UBC**-PSEN1

THRB-C1ORF103-**UBC**-PSEN1

THRB-MED21-**UBC**-PSEN1

THRB-EIF3I-**UBC**-PSEN1

THRB-TRIP4-**UBC**-PSEN1

THRB-CNOT1-**UBC**-PSEN1

THRB-PARP1-**UBC**-PSEN1

THRB-CAMK2B-**UBC**-PSEN1

THRB-HMGN3-**UBC**-PSEN1

THRB-RARA-**UBC**-PSEN1

THRB-NCOR2-**UBC**-PSEN1

THRB-NCOR1-**UBC**-PSEN1

THRB-FOXO1-**UBC**-PSEN1

THRB-BRD8-**UBC**-PSEN1

THRB-PSMC5-**UBC**-PSEN1

THRB-MED1-**UBC**-PSEN1

THRB-PRMT1-**UBC**-PSEN1

THRB-SRC-**UBC**-PSEN1

THRB-JMJD1C-**UBC**-PSEN1

THRB-TSG101-**UBC**-PSEN1

THRB-SUMO2-**UBC**-PSEN1

THRB-SUMO1-**UBC**-PSEN1

THRB-HSPA4-TRAF6-PSEN1

THRB-EIF3I-TRAF6-PSEN1

THRB-PARP1-TRAF6-PSEN1

THRB-PSMC5-TRAF6-PSEN1

THRB-PRMT1-TRAF6-PSEN1

THRB-SRC-TRAF6-PSEN1

THRB-SUMO1-TRAF6-PSEN1

THRB-SUMO2-ST13-PSEN1

THRB-TRIP10-PRKACA-PSEN1

THRB-CCND1-PRKACA-PSEN1

THRB-ESR1-PRKACA-PSEN1

THRB-SRC-PRKACA-PSEN1

THRB-RARA-UBQLN1-PSEN2

THRB-TP53-CSNK1D-PSEN2

THRB-XRCC6-NOTCH1-PSEN2

THRB-HSPA4-APP-PSEN2

THRB-SUMO1-APP-PSEN2

THRB-TP53-CAPN1-PSEN2

THRB-SUMO2-CAPN1-PSEN2

THRB-TP53-BCL2L1-PSEN2

THRB-SUMO1-CASP8-PSEN2

THRB-PARP1-CASP7-PSEN2

THRB-PRKDC-CASP3-PSEN2

THRB-PARP1-CASP3-PSEN2

THRB-PARP1-CASP1-PSEN2

THRB-SUMO2-PDCD4-PSEN2

THRB-TP53-CFLAR-PSEN2

THRB-XRCC6-CFLAR-PSEN2

THRB-RXRA-CTNNB1-PSEN2

THRB-TRIP10-CTNNB1-PSEN2

THRB-NCOA2-CTNNB1-PSEN2

THRB-HDAC2-CTNNB1-PSEN2

THRB-TBL1X-CTNNB1-PSEN2

THRB-ESR1-CTNNB1-PSEN2

THRB-FOXO1-CTNNB1-PSEN2

THRB-SRC-CTNNB1-PSEN2

THRB-SUMO1-CTNNB1-PSEN2

THRB-PRKDC-CSNK2A1-PSEN2

THRB-COPS2-CSNK2A1-PSEN2

THRB-HDAC3-CSNK2A1-PSEN2

THRB-HDAC2-CSNK2A1-PSEN2

THRB-HSPA4-CSNK2A1-PSEN2

THRB-TP53-CSNK2A1-PSEN2

THRB-XRCC6-CSNK2A1-PSEN2

THRB-XRCC5-CSNK2A1-PSEN2

THRB-SRC-CSNK2A1-PSEN2

THRB-SUMO1-CSNK2A1-PSEN2

THRB-RXRB-**UBC**-PSEN2

THRB-LIG3-**UBC**-PSEN2

THRB-TRIP13-**UBC**-PSEN2

THRB-TRIP12-**UBC**-PSEN2

THRB-NR2F6-**UBC**-PSEN2

tTHRB-TBL1XR1-**UBC**-PSEN2

THRB-TRIP10-**UBC**-PSEN2

THRB-CTCF-**UBC**-PSEN2

THRB-PRKDC-**UBC**-PSEN2

THRB-COPS2-**UBC**-PSEN2

THRB-MPG-**UBC**-PSEN2

THRB-NRIP1-**UBC**-PSEN2

THRB-ZNHIT3-**UBC**-PSEN2

THRB-NCOA3-**UBC**-PSEN2

THRB-PPARGC1A-**UBC**-PSEN2

THRB-NCOA2-**UBC**-PSEN2

THRB-GPS2-**UBC**-PSEN2

THRB-NCOA1-**UBC**-PSEN2

THRB-HDAC3-**UBC**-PSEN2

THRB-HDAC2-**UBC**-PSEN2

THRB-HSPA4-**UBC**-PSEN2

THRB-TP53-**UBC**-PSEN2

THRB-XRCC6-**UBC**-PSEN2

THRB-XRCC5-**UBC**-PSEN2

THRB-CCND1-**UBC**-PSEN2

**THRB-ESR1-UBC-PSEN2**

THRB-SMC1A-**UBC**-PSEN2

THRB-MED25-**UBC**-PSEN2

THRB-C1D-**UBC**-PSEN2

THRB-TRIP6-**UBC**-PSEN2

THRB-C1ORF103-**UBC**-PSEN2

THRB-MED21-**UBC**-PSEN2

THRB-EIF3I-**UBC**-PSEN2

THRB-TRIP4-**UBC**-PSEN2

THRB-CNOT1-**UBC**-PSEN2

THRB-PARP1-**UBC**-PSEN2

THRB-CAMK2B-**UBC**-PSEN2

THRB-HMGN3-**UBC**-PSEN2

THRB-RARA-**UBC**-PSEN2

THRB-NCOR2-**UBC**-PSEN2

THRB-NCOR1-**UBC**-PSEN2

THRB-FOXO1-**UBC**-PSEN2

THRB-BRD8-**UBC**-PSEN2

THRB-PSMC5-**UBC**-PSEN2

THRB-MED1-**UBC**-PSEN2

THRB-PRMT1-**UBC**-PSEN2

THRB-SRC-**UBC**-PSEN2

THRB-JMJD1C-**UBC**-PSEN2

THRB-TSG101-**UBC**-PSEN2

THRB-SUMO2-**UBC**-PSEN2

THRB-SUMO1-**UBC**-PSEN2

THRB-ESR1-FHL2-PSEN2

THRB-FOXO1-FHL2-PSEN2

PSEN1-TRAF6-NTRK2-NTF3

PSEN1-**UBC**-NTRK2-NTF3

PSEN1-PIK3R1-NTRK2-NTF3

PSEN1-CDC37-NTRK1-NTF3

PSEN1-TRAF6-NTRK1-NTF3

PSEN1-**UBC**-NTRK1-NTF3

PSEN1-PIK3R1-NTRK1-NTF3

PSEN1-ACTN1-ESR1-BDNF

PSEN1-GAPDH-ESR1-BDNF

PSEN1-PRKCZ-ESR1-BDNF

PSEN1-CTNNB1-ESR1-BDNF

PSEN1-PRKACA-ESR1-BDNF

PSEN1-**UBC**-ESR1-BDNF

PSEN1-PIK3R1-ESR1-BDNF

PSEN1-APH1A-ELAVL1-BDNF

PSEN1-CASP3-ELAVL1-BDNF

PSEN1-NCSTN-ELAVL1-BDNF

PSEN1-GSK3B-ELAVL1-BDNF

PSEN1-ST13-ELAVL1-BDNF

PSEN1-**UBC**-ELAVL1-BDNF

PSEN1-TRAF6-NTRK2-BDNF

PSEN1-**UBC**-NTRK2-BDNF

PSEN1-PIK3R1-NTRK2-BDNF

PSEN1-CASP3-HMGB1-ATOH1

PSEN1-**UBC**-HMGB1-ATOH1

PSEN2-BCL2L1-TLE1-BARHL1

PSEN2-CTNNB1-TLE1-BARHL1

PSEN2-**UBC**-MED7-BARHL1

PSEN2-FHL2-ESR1

**PSEN2-UBC-ESR1**

PSEN2-CTNNB1-ESR1

PSEN2-**UBC**-NTRK2-NTF3

PSEN2-**UBC**-NTRK1-NTF3

PSEN2-FHL2-ESR1-BDNF

**PSEN2-UBC-ESR1-BDNF**

PSEN2-CTNNB1-ESR1-BDNF

PSEN2-**UBC**-ELAVL1-BDNF

PSEN2-CASP3-ELAVL1-BDNF

PSEN2-APH1A-ELAVL1-BDNF

PSEN2-CSNK2A1-ELAVL1-BDNF

PSEN2-PDCD4-ELAVL1-BDNF

PSEN2-NCSTN-ELAVL1-BDNF

PSEN2-**UBC**-NTRK2-BDNF

PSEN2-**UBC**-HMGB1-ATOH1

PSEN2-CASP3-HMGB1-ATOH1

**Supplementary Table S9:** Functional enrichment and annotation analysis of UBC, SUMO1, SUMO2, HMGB1, TLE2 by ToppFun

**GO: Biological Process 471 annotations before applied cutoff / 18482 genes in category**

**ID	Name	pValue	FDR B&H	FDR B&Y	Bonferroni	Genes from Input	Genes in Annotation**

1	GO:0045892	negative regulation of transcription, DNA-templated	4.258E-7	5.887E-5	3.964E-4	2.006E-4	5	985

2	GO:1902679	negative regulation of RNA biosynthetic process	4.710E-7	5.887E-5	3.964E-4	2.218E-4	5	1005

5	GO:0010629	negative regulation of gene expression	8.213E-7	5.887E-5	3.964E-4	3.868E-4	5	1123

6	GO:0045934	nucleobase-containing compound metabolic process 9.051E-7	5.887E-5	3.964E-4	4.263E-4	5	1145

8	GO:0051172	nitrogen compound metabolic process	1.000E-6	5.887E-5	3.964E-4	4.710E-4	5	1168

13	GO:0016485	protein processing	8.716E-6	3.158E-4	2.126E-3	4.105E-3	4	678

14	GO:0051604	protein maturation	9.674E-6	3.255E-4	2.191E-3	4.556E-3	4	696

17	GO:0043161	proteasome-mediated ubiquitin-dependent protein catabolic process 2.918E-5	8.085E-4	5.444E-3	3	267

18	GO:0010498	proteasomal protein catabolic process	3.621E-5	9.475E-4	6.380E-3	1.706E-2	3	287

19	GO:0016925	protein sumoylation	4.321E-5	1.071E-3	7.213E-3	2.035E-2	2	39

20	GO:0009057	macromolecule catabolic process	5.212E-5	1.227E-3	8.265E-3	2.455E-2	4	1064

21	GO:0032436	proteasomal ubiquitin-dependent protein catabolic process 6.299E-5 1.413E-3	9.512E-3	2.967E-2	2	47

22	GO:1901800	positive regulation of proteasomal protein catabolic process 7.426E-5 1.581E-3	1.065E-2	3.498E-2	2	51

23	GO:1903052	proteolysis involved in cellular protein catabolic process 7.722E-5	1.581E-3	1.065E-2	3.637E-2	2	52

24	GO:0006281	DNA repair	1.225E-4	2.403E-3	1.618E-2	5.768E-2	3	432

25	GO:0006511	ubiquitin-dependent protein catabolic process	1.438E-4	2.629E-3	1.770E-2	6.773E-2	3	456

26	GO:0006508	proteolysis	1.485E-4	2.629E-3	1.770E-2	6.994E-2	4	1387

27	GO:0019941	modification-dependent protein catabolic process	1.524E-4	2.629E-3	1.770E-2	7.177E-2	3	465

28	GO:0043632	modification-dependent macromolecule catabolic process 1.563E-4 2.629E-3	1.770E-2	7.362E-2	3	469

29	GO:0032434	regulation of proteasomal ubiquitin-dependent protein catabolic process 1.699E-4 2.760E-3	1.858E-2	2	77

30	GO:0051603	proteolysis involved in cellular protein catabolic process 1.924E-4	2.929E-3	1.972E-2	9.061E-2	3	503

36	GO:2000426	negative regulation of apoptotic cell clearance	2.705E-4	3.539E-3	2.383E-2	1.274E-1	1	1

49	GO:0007219	Notch signaling pathway	8.454E-4	8.126E-3	5.471E-2	3.982E-1	2	172

50	GO:0045087	innate immune response	1.011E-3	9.522E-3	6.411E-2	4.761E-1	3	883

**Pathway: 213 annotations before applied cutoff / 10724 genes in category**

**ID	Name	Source	pValue	FDR B&H	FDR B&Y	Bonferroni	Genes from Input	Genes in Annotation**

1	833808	Conjugation of SUMO to E1 (UBA2:SAE1)	BioSystems: REACTOME	2.607E-6	2.027E-4	1.204E-3	5.553E-4	2	6

2	833809	Transfer of SUMO from E1 to E2 (UBE2I, UBC9)	BioSystems: REACTOME	3.649E-6	2.027E-4	1.204E-3	7.772E-4	2	7

3	833807	Proteolytic Processing of SUMO	BioSystems: REACTOME	3.649E-6	2.027E-4	1.204E-3	7.772E-4	2	7

4	P00059	p53 pathway	PantherDB	3.807E-6	2.027E-4	1.204E-3	8.110E-4	3	79

5	833806	Processing and Activation of SUMO	BioSystems: REACTOME	7.815E-6	2.774E-4	1.648E-3	1.665E-3	2	10

6	833805	SUMOylation	BioSystems: REACTOME	7.815E-6	2.774E-4	1.648E-3	1.665E-3	2	10

7	576271	NOTCH1 Intracellular Domain Regulates Transcription	BioSystems: REACTOME	1.945E-4	5.919E-3	3.516E-2	4.143E-2	2	48

8	576255	Cytosolic sensors of pathogen-associated DNA	BioSystems: REACTOME	3.913E-4	6.405E-3	3.805E-2	8.336E-2	2	68

9	771592	Signaling by NOTCH1 PEST Domain Mutants in Cancer	BioSystems: REACTOME	4.510E-4	6.405E-3	3.805E-2	9.607E-2	2	73

10	771594	Signaling by NOTCH1 HD+PEST Domain Mutants in Cancer	BioSystems: REACTOME	4.510E-4	6.405E-3	3.805E-2	9.607E-2	2	73

11	771588	Signaling by NOTCH1 t(7;9)(NOTCH1:M1580 K2555) Translocation Mutant REACTOME	4.510E-4	6.405E-3	3.805E-2	9.607E-2	2	73

12	771587	Signaling by NOTCH1 in Cancer	BioSystems: REACTOME	4.510E-4	6.405E-3	3.805E-2	9.607E-2	2	73

13	576269	Signaling by NOTCH1	BioSystems: REACTOME	4.510E-4	6.405E-3	3.805E-2	9.607E-2	2	73

14	771590	Signaling by NOTCH1 HD Domain Mutants in Cancer	BioSystems: REACTOME	4.510E-4	6.405E-3	3.805E-2	9.607E-2	2	73

15	771596	FBXW7 Mutants and NOTCH1 in Cancer	BioSystems: REACTOME	4.510E-4	6.405E-3	3.805E-2	9.607E-2	2	73

16	187097	RIG-I/MDA5 mediated induction of IFN-alpha/beta pathways	BioSystems: REACTOME	5.416E-4	6.854E-3	4.072E-2	1.154E-1	2	80

17	187082	TRAF6 mediated induction of NFkB and MAP kinases upon TLR7/8 or 9 activation REACTOME	5.830E-4	6.854E-3	4.072E-2	1.242E-1	2	83

18	187081	MyD88 dependent cascade initiated on endosome	BioSystems: REACTOME	6.113E-4	6.854E-3	4.072E-2	1.302E-1	2	85

19	106396	Toll Like Receptor 7/8 (TLR7/8) Cascade	BioSystems: REACTOME	6.113E-4	6.854E-3	4.072E-2	1.302E-1	2	85

20	106397	Toll Like Receptor 9 (TLR9) Cascade	BioSystems: REACTOME	6.701E-4	7.137E-3	4.240E-2	1.427E-1	2	89

21	106401	MyD88-independent cascade	BioSystems: REACTOME	7.955E-4	7.702E-3	4.576E-2	1.694E-1	2	97

22	106391	Toll Like Receptor 3 (TLR3) Cascade	BioSystems: REACTOME	7.955E-4	7.702E-3	4.576E-2	1.694E-1	2	97

23	106485	Signaling by NOTCH	BioSystems: REACTOME	1.097E-3	9.906E-3	5.885E-2	2.337E-1	2	114

24	198799	Wnt Signaling Pathway NetPath	BioSystems: WikiPathways	1.116E-3	9.906E-3	5.885E-2	2.378E-1	2	115

27	105648	Apoptosis	BioSystems: REACTOME	2.282E-3	1.801E-2	1.070E-1	4.862E-1	2	165

28	187103	Interferon Signaling	BioSystems: REACTOME	2.535E-3	1.928E-2	1.146E-1	5.399E-1	2	174

30	530745	Downregulation of ERBB4 signaling	BioSystems: REACTOME	4.190E-3	2.975E-2	1.767E-1	8.925E-1	1	9

31	M7239	Apoptotic DNA fragmentation and tissue homeostasis	MSigDB C2: BioCarta	4.655E-3	3.107E-2	1.846E-1	9.914E-1	1	10

32	187086	IRAK2 mediated activation of TAK1 complex	BioSystems: REACTOME	5.119E-3	3.107E-2	1.846E-1	1.000E0	1	11

36	M13917	Role of Parkin i

**Supplementary Table S10:** List of Up/Down regulated miRNAs in AD collected from PubMed literature

| **Upregulated miRNAs** | **PMID** | **Downregulated miRNAs** | **PMID** |
| --- | --- | --- | --- |
| miR-519e | PMID:20936480 | miR-485-3p | PMID:20936480 |
| miR-574-5p | PMID:20936480 | miR-381 | PMID:20936480 |
| miR-155 | PMCID: PMC3547332 | miR-124 | PMID:17314675 |
| miR-490-3p | PMID:20936480 | miR-129-5p | PMID:20936480 |
| miR-549 | PMID:20936480 | miR-143 | PMID:20936480 |
| miR-516a-5p | PMID:20936480 | miR-136 | PMID:20936480 |
| miR-510 | PMID:20936480 | miR-138 | PMID:20936480 |
| miR-184 | PMID:20936480 | miR-129-3p | PMID:20936480 |
| miR-184 | PMID:20936480 | miR-128 | PMID:20936480 |
| miR-516b | PMID:20936480 | miR-379 | PMID:20936480 |
| miR-298 | PMID:20936480 | miR-299-5p | PMID:20936480 |
| miR-214 | PMID:20936480 | miR-218 | PMID:20936480 |
| miR-198 | PMID:20936480 | miR-149 | PMID:20936480 |
| miR-451 | PMID:20936480 | miR-135a | PMID:20936480 |
| miR-144 | PMID:20936480 | miR-7 | PMID:20936480 |
| miR-424 | PMID:20936480 | miR-126 | PMID:20936480 |
| let-7e | PMID:20936480 | miR-411 | PMID:20936480 |
| miR-208a | PMID:20936480 | miR-335 | PMID:20936480 |
| miR-574-3p | PMID:20936480 | miR-378 | PMID:20936480 |
| miR-576-5p | PMID:20936480 | miR-488 | PMID:20936480 |
| miR-215 | PMID:20936480 | miR-127-5p | PMID:20936480 |
| miR-132 | **PMID: 18525125** | miR-127-3p | PMID:20936480 |
| miR-34a | PMID:18525125 | miR-491-5p | PMID:20936480 |
| miR-200a | PMID:19881909 | miR-377 | PMID:20936480 |
| let-7f | **PMID:19881909** | miR-376c | PMID:20936480 |
| miR-26a | PMID:18525125 | miR-222 | PMID:20936480 |
| miR-27a | PMID:18525125 | miR-95 | PMID:20936480 |
| miR-381 | PMID:18525125 | miR-495 | PMID:20936480 |
| miR-27b | PMID: 18525125 | miR-329 | PMID:20936480 |
| miR-422a | PMID:18525125 | miR-195 | PMID:20936480 |
| miR-200c | **PMID: 18525125** | miR-551b | PMID:20936480 |
| miR-146a | **PMID: 20937840** | miR-30b | PMID:20936480 |
| miR-30c | **PMID: 18525125** | miR-107 | PMID:18234899 |
| miR-100 | **PMID: 18525125** | miR-221 | PMID:20936480 |
| miR-125b | PMID:17314675,PMID:17629564 | miR-139-5p | PMID:20936480 |
| miR-145 | PMID:17314675,PMID:17629564,PMID:18525125 | miR-146b-5p | PMID:20936480 |
| miR-148a | **PMID: 18525125** | miR-29c | PMID:20936480 |
| miR-29a | **PMID: 18525125** | miR-30a | PMID:20936480 |
| miR-29b | **PMID: 18525125** | miR-582-5p | PMID:20936480 |
| miR-617 | **PMID:20126538** | miR-103 | PMID:20936480 |
| miR-134 | **PMID:20126538** | miR-342-3p | PMID:20936480 |
| miR-601 | **PMID:20126538** | miR-30c | PMID:20936480 |
| miR-185 | **PMID:20126538** | miR-30d | PMID:20936480 |
| miR-575 | **PMID:20126538** | miR-382 | PMID:20936480 |
| miR-765 | **PMID:20126538** | miR-22 | PMID:20936480 |
| miR-572 | **PMID:20126538** | miR-125a-5p | PMID:20936480 |
| miR-197 | **PMID:19110058** | miR-425 | PMID:20936480 |
| miR-511 | **PMID:19110058** | miR-191 | PMID:20936480 |
| miR-9 | **PMID:19406203** | miR-519d | PMID:20936480 |
|  |  | let-7g | PMID:20936480 |
|  |  | miR-98 | PMID:20936480 |
|  |  | miR-99a | PMID:20936480 |
|  |  | miR-30e | PMID:20936480 |
|  |  | miR-101 | PMCID: PMC3547332 |
|  |  | miR-212 | PMID:23585551 |
|  |  | miR-210 | PMID:18525125 |
|  |  | miR-425 | PMID:18525125 |
|  |  | miR-15a | PMID:18434550 |
|  |  | miR-95 | PMID:20936480 |
|  |  | miR-130a | PMID:20126538 |
|  |  | miR-376a | **PMID:20126538** |
|  |  | miR-598 | **PMID:20126538** |
|  |  | miR-494 | **PMID:20126538** |
|  |  | miR-20b | **PMID:20126538** |
|  |  | miR-181c | **PMID:19110058** |
|  |  | let-7i | **PMID:19110058** |
|  |  | miR-26b | **PMID:19110058** |
|  |  | miR-363 | **PMID:19110058** |
|  |  | miR-93 | **PMID:19110058** |
|  |  | miR-491-3p | PMID:20936480 |
|  |  | miR-423-5p | PMID:20936480 |
|  |  | miR-34b | PMID:20936480 |
|  |  | miR-34c-5p | PMID:20936480 |
|  |  | miR-422a | PMID:20936480 |
|  |  | miR-219-5p | PMID:20936480 |
|  |  | miR-23a | PMID:20936480 |
|  |  | miR-584 | PMID:20936480 |
|  |  | miR-326 | PMID:20936480 |
|  |  | miR-338-5p | PMID:20936480 |
|  |  | miR-219-2-3p | PMID:20936480 |
|  |  | miR-181a | PMID:20936480 |
|  |  | miR-181b | PMID:20936480 |
|  |  | let-7b | PMID:20936480 |
|  |  | miR-19a | PMID:20936480 |
|  |  | miR-151-3p | PMID:20936480 |
|  |  | miR-197 | PMID:20936480 |
|  |  | miR-211 | PMCID: PMC3657400 |
|  |  | miR-20a | PMID:20936480 |
|  |  | miR-17 | PMID:20936480 |
|  |  | miR-106a | PMID:20936480 |
|  |  | miR-32 | PMID:20936480 |
|  |  | miR-340 | PMID:20936480 |
|  |  | miR-19b | PMID:20936480 |
|  |  | miR-21 | PMID:20936480 |
|  |  | miR-151-5p | PMID:20936480 |
|  |  | miR-194 | PMID:20936480 |
|  |  | let-7c | PMID:20936480 |
|  |  | miR-330-3p | PMID:20936480 |
|  |  | miR-27b | PMID:20936480 |
|  |  |  |  |
|  |  |  |  |
|  |  | miR-339-5p | PMID:20936480 |
|  |  | miR-193b | PMID:20936480 |
|  |  | miR-106b | PMID:20936480 |
|  |  | miR-16 | PMID:20936480 |
|  |  | miR-320a | PMID:20936480 |
|  |  | miR-23b | PMID:20936480 |
|  |  | miR-15b | PMID:20936480 |
|  |  | miR-320d | PMID:20936480 |
|  |  | miR-320b | PMID:20936480 |
|  |  | miR-320c | PMID:20936480 |
|  |  | miR-557 | PMID:20936480 |
|  |  | miR-33a | PMID:20936480 |
|  |  | miR-24 | PMID:20936480 |
|  |  | miR-140-3p | PMID:20936480 |
|  |  | miR-374b | PMID:20936480 |
|  |  | miR-374a | PMID:20936480 |
|  |  | miR-140-5p | PMID:20936480 |
|  |  | miR-26a | PMID:20936480 |
|  |  | miR-513a-5p | PMID:20936480 |
|  |  | miR-142-5p | PMID:20936480 |
|  |  | miR-142-3p | PMID:20936480 |
|  |  | miR-520d-5p | PMID:20936480 |
|  |  | miR-193a-3p | PMID:20936480 |
|  |  | miR-92b, | PMID:20936480 |
|  |  | miR-330-5p | PMID:20936480 |
|  |  | miR-186 | PMID:20936480 |
|  |  | miR-223 | PMID:20936480 |
|  |  | miR-412 | PMID:20936480 |
|  |  | miR-148b | PMID:20936480 |
|  |  | miR-99b | PMID:20936480 |
|  |  | miR-589 | PMID:20936480 |
|  |  | miR-361-3p | PMID:20936480 |
|  |  | miR-361-5p | PMID:20936480 |
|  |  | miR-423-3p | PMID:20936480 |
|  |  | miR-190 | PMID:20936480 |
|  |  | miR-301a | PMID:20936480 |
|  |  | miR-365 | PMID:20936480 |
|  |  | miR-331-3p | PMID:20936480 |

**Supplementary Table S11:** Validated targeting miRNAs of BARH-ESR and AD path key genes derived from miRWalk.

| **BARHL1-ESR Pathway gene targeting validated miRs from miRWalk** | | | | | |
| --- | --- | --- | --- | --- | --- |
| **Gene** | **MicroRNA Name** | **Pubmed ID** | **Gene** | **MicroRNA Name** | **Pubmed ID** |
| [ESR1](http://www.ncbi.nlm.nih.gov/sites/entrez?Db=gene&Cmd=ShowDetailView&TermToSearch=2099&ordinalpos=1&itool=EntrezSystem2.PEntrez.Gene.Gene_ResultsPanel.Gene_RVDocSum) | [hsa-let-7a](http://www.mirbase.org/cgi-bin/mirna_entry.pl?acc=MI0000060) | [22081076](http://www.ncbi.nlm.nih.gov/sites/entrez?db=pubmed&cmd=search&term=22081076) | [BDNF](http://www.ncbi.nlm.nih.gov/sites/entrez?Db=gene&Cmd=ShowDetailView&TermToSearch=627&ordinalpos=1&itool=EntrezSystem2.PEntrez.Gene.Gene_ResultsPanel.Gene_RVDocSum) | [hsa-let-7d](http://www.mirbase.org/cgi-bin/mirna_entry.pl?acc=MI0000065) | [19703567](http://www.ncbi.nlm.nih.gov/sites/entrez?db=pubmed&cmd=search&term=19703567) |
|  | [hsa-let-7a](http://www.mirbase.org/cgi-bin/mirna_entry.pl?acc=MI0000061) | [22081076](http://www.ncbi.nlm.nih.gov/sites/entrez?db=pubmed&cmd=search&term=22081076) |  | [hsa-let-7d*](http://www.mirbase.org/cgi-bin/mirna_entry.pl?acc=MI0000065) | [19703567](http://www.ncbi.nlm.nih.gov/sites/entrez?db=pubmed&cmd=search&term=19703567) |
|  | [hsa-let-7a](http://www.mirbase.org/cgi-bin/mirna_entry.pl?acc=MI0000062) | [22081076](http://www.ncbi.nlm.nih.gov/sites/entrez?db=pubmed&cmd=search&term=22081076) |  | [hsa-miR-1](http://www.mirbase.org/cgi-bin/mirna_entry.pl?acc=MI0000437) | [14697198](http://www.ncbi.nlm.nih.gov/sites/entrez?db=pubmed&cmd=search&term=14697198) |
|  | [hsa-let-7a](http://www.mirbase.org/cgi-bin/mirna_entry.pl?acc=MI0000060) | [20600201](http://www.ncbi.nlm.nih.gov/sites/entrez?db=pubmed&cmd=search&term=20600201) |  | [hsa-miR-1](http://www.mirbase.org/cgi-bin/mirna_entry.pl?acc=MI0000651) | [14697198](http://www.ncbi.nlm.nih.gov/sites/entrez?db=pubmed&cmd=search&term=14697198) |
|  | [hsa-let-7a](http://www.mirbase.org/cgi-bin/mirna_entry.pl?acc=MI0000061) | [20600201](http://www.ncbi.nlm.nih.gov/sites/entrez?db=pubmed&cmd=search&term=20600201) |  | [hsa-miR-101](http://www.mirbase.org/cgi-bin/mirna_entry.pl?acc=MI0000739) | [14697198](http://www.ncbi.nlm.nih.gov/sites/entrez?db=pubmed&cmd=search&term=14697198) |
|  | [hsa-let-7a](http://www.mirbase.org/cgi-bin/mirna_entry.pl?acc=MI0000060) | [19528081](http://www.ncbi.nlm.nih.gov/sites/entrez?db=pubmed&cmd=search&term=19528081) |  | [hsa-miR-101](http://www.mirbase.org/cgi-bin/mirna_entry.pl?acc=MI0000103) | [14697198](http://www.ncbi.nlm.nih.gov/sites/entrez?db=pubmed&cmd=search&term=14697198) |
|  | [hsa-let-7a](http://www.mirbase.org/cgi-bin/mirna_entry.pl?acc=MI0000062) | [20600201](http://www.ncbi.nlm.nih.gov/sites/entrez?db=pubmed&cmd=search&term=20600201) |  | [hsa-miR-107](http://www.mirbase.org/cgi-bin/mirna_entry.pl?acc=MI0000114) | [18632683](http://www.ncbi.nlm.nih.gov/sites/entrez?db=pubmed&cmd=search&term=18632683) |
|  | [hsa-let-7a](http://www.mirbase.org/cgi-bin/mirna_entry.pl?acc=MI0000061) | [19528081](http://www.ncbi.nlm.nih.gov/sites/entrez?db=pubmed&cmd=search&term=19528081) |  | [hsa-miR-10a](http://www.mirbase.org/cgi-bin/mirna_entry.pl?acc=MI0000266) | [20309390](http://www.ncbi.nlm.nih.gov/sites/entrez?db=pubmed&cmd=search&term=20309390) |
|  | [hsa-let-7a](http://www.mirbase.org/cgi-bin/mirna_entry.pl?acc=MI0000062) | [19528081](http://www.ncbi.nlm.nih.gov/sites/entrez?db=pubmed&cmd=search&term=19528081) |  | [hsa-miR-10a*](http://www.mirbase.org/cgi-bin/mirna_entry.pl?acc=MI0000266) | [20309390](http://www.ncbi.nlm.nih.gov/sites/entrez?db=pubmed&cmd=search&term=20309390) |
|  | [hsa-let-7a*](http://www.mirbase.org/cgi-bin/mirna_entry.pl?acc=MI0000060) | [22081076](http://www.ncbi.nlm.nih.gov/sites/entrez?db=pubmed&cmd=search&term=22081076) |  | [hsa-miR-124](http://www.mirbase.org/cgi-bin/mirna_entry.pl?acc=MI0000443) | [19703567](http://www.ncbi.nlm.nih.gov/sites/entrez?db=pubmed&cmd=search&term=19703567) |
|  | [hsa-let-7a*](http://www.mirbase.org/cgi-bin/mirna_entry.pl?acc=MI0000062) | [22081076](http://www.ncbi.nlm.nih.gov/sites/entrez?db=pubmed&cmd=search&term=22081076) |  | [hsa-miR-124](http://www.mirbase.org/cgi-bin/mirna_entry.pl?acc=MI0000444) | [19703567](http://www.ncbi.nlm.nih.gov/sites/entrez?db=pubmed&cmd=search&term=19703567) |
|  | [hsa-let-7a*](http://www.mirbase.org/cgi-bin/mirna_entry.pl?acc=MI0000060) | [19528081](http://www.ncbi.nlm.nih.gov/sites/entrez?db=pubmed&cmd=search&term=19528081) |  | [hsa-miR-124](http://www.mirbase.org/cgi-bin/mirna_entry.pl?acc=MI0000445) | [19703567](http://www.ncbi.nlm.nih.gov/sites/entrez?db=pubmed&cmd=search&term=19703567) |
|  | [hsa-let-7a*](http://www.mirbase.org/cgi-bin/mirna_entry.pl?acc=MI0000062) | [19528081](http://www.ncbi.nlm.nih.gov/sites/entrez?db=pubmed&cmd=search&term=19528081) |  | [hsa-miR-130a](http://www.mirbase.org/cgi-bin/mirna_entry.pl?acc=MI0000448) | [14697198](http://www.ncbi.nlm.nih.gov/sites/entrez?db=pubmed&cmd=search&term=14697198) |
|  | [hsa-let-7b](http://www.mirbase.org/cgi-bin/mirna_entry.pl?acc=MI0000063) | [19528081](http://www.ncbi.nlm.nih.gov/sites/entrez?db=pubmed&cmd=search&term=19528081) |  | [hsa-miR-130a*](http://www.mirbase.org/cgi-bin/mirna_entry.pl?acc=MI0000448) | [14697198](http://www.ncbi.nlm.nih.gov/sites/entrez?db=pubmed&cmd=search&term=14697198) |
|  | [hsa-let-7b](http://www.mirbase.org/cgi-bin/mirna_entry.pl?acc=MI0000063) | [22081076](http://www.ncbi.nlm.nih.gov/sites/entrez?db=pubmed&cmd=search&term=22081076) |  | [hsa-miR-132](http://www.mirbase.org/cgi-bin/mirna_entry.pl?acc=MI0000449) | [19958814](http://www.ncbi.nlm.nih.gov/sites/entrez?db=pubmed&cmd=search&term=19958814) |
|  | [hsa-let-7b*](http://www.mirbase.org/cgi-bin/mirna_entry.pl?acc=MI0000063) | [19528081](http://www.ncbi.nlm.nih.gov/sites/entrez?db=pubmed&cmd=search&term=19528081) |  | [hsa-miR-132](http://www.mirbase.org/cgi-bin/mirna_entry.pl?acc=MI0000449) | [17994015](http://www.ncbi.nlm.nih.gov/sites/entrez?db=pubmed&cmd=search&term=17994015) |
|  | [hsa-let-7b*](http://www.mirbase.org/cgi-bin/mirna_entry.pl?acc=MI0000063) | [22081076](http://www.ncbi.nlm.nih.gov/sites/entrez?db=pubmed&cmd=search&term=22081076) |  | [hsa-miR-132](http://www.mirbase.org/cgi-bin/mirna_entry.pl?acc=MI0000449) | [21723895](http://www.ncbi.nlm.nih.gov/sites/entrez?db=pubmed&cmd=search&term=21723895) |
|  | [hsa-let-7c](http://www.mirbase.org/cgi-bin/mirna_entry.pl?acc=MI0000064) | [19528081](http://www.ncbi.nlm.nih.gov/sites/entrez?db=pubmed&cmd=search&term=19528081) |  | [hsa-miR-132](http://www.mirbase.org/cgi-bin/mirna_entry.pl?acc=MI0000449) | [20307261](http://www.ncbi.nlm.nih.gov/sites/entrez?db=pubmed&cmd=search&term=20307261) |
|  | [hsa-let-7c](http://www.mirbase.org/cgi-bin/mirna_entry.pl?acc=MI0000064) | [22081076](http://www.ncbi.nlm.nih.gov/sites/entrez?db=pubmed&cmd=search&term=22081076) |  | [hsa-miR-132*](http://www.mirbase.org/cgi-bin/mirna_entry.pl?acc=MI0000449) | [19958814](http://www.ncbi.nlm.nih.gov/sites/entrez?db=pubmed&cmd=search&term=19958814) |
|  | [hsa-let-7c*](http://www.mirbase.org/cgi-bin/mirna_entry.pl?acc=MI0000064) | [19528081](http://www.ncbi.nlm.nih.gov/sites/entrez?db=pubmed&cmd=search&term=19528081) |  | [hsa-miR-132*](http://www.mirbase.org/cgi-bin/mirna_entry.pl?acc=MI0000449) | [17994015](http://www.ncbi.nlm.nih.gov/sites/entrez?db=pubmed&cmd=search&term=17994015) |
|  | [hsa-let-7c*](http://www.mirbase.org/cgi-bin/mirna_entry.pl?acc=MI0000064) | [22081076](http://www.ncbi.nlm.nih.gov/sites/entrez?db=pubmed&cmd=search&term=22081076) |  | [hsa-miR-132*](http://www.mirbase.org/cgi-bin/mirna_entry.pl?acc=MI0000449) | [21723895](http://www.ncbi.nlm.nih.gov/sites/entrez?db=pubmed&cmd=search&term=21723895) |
|  | [hsa-let-7d](http://www.mirbase.org/cgi-bin/mirna_entry.pl?acc=MI0000065) | [19528081](http://www.ncbi.nlm.nih.gov/sites/entrez?db=pubmed&cmd=search&term=19528081) |  | [hsa-miR-132*](http://www.mirbase.org/cgi-bin/mirna_entry.pl?acc=MI0000449) | [20307261](http://www.ncbi.nlm.nih.gov/sites/entrez?db=pubmed&cmd=search&term=20307261) |
|  | [hsa-let-7d](http://www.mirbase.org/cgi-bin/mirna_entry.pl?acc=MI0000065) | [20600201](http://www.ncbi.nlm.nih.gov/sites/entrez?db=pubmed&cmd=search&term=20600201) |  | [hsa-miR-134](http://www.mirbase.org/cgi-bin/mirna_entry.pl?acc=MI0000474) | [20622856](http://www.ncbi.nlm.nih.gov/sites/entrez?db=pubmed&cmd=search&term=20622856) |
|  | [hsa-let-7d](http://www.mirbase.org/cgi-bin/mirna_entry.pl?acc=MI0000065) | [22081076](http://www.ncbi.nlm.nih.gov/sites/entrez?db=pubmed&cmd=search&term=22081076) |  | [hsa-miR-134](http://www.mirbase.org/cgi-bin/mirna_entry.pl?acc=MI0000474) | [21723895](http://www.ncbi.nlm.nih.gov/sites/entrez?db=pubmed&cmd=search&term=21723895) |
|  | [hsa-let-7d*](http://www.mirbase.org/cgi-bin/mirna_entry.pl?acc=MI0000065) | [22081076](http://www.ncbi.nlm.nih.gov/sites/entrez?db=pubmed&cmd=search&term=22081076) |  | [hsa-miR-138](http://www.mirbase.org/cgi-bin/mirna_entry.pl?acc=MI0000455) | [21168126](http://www.ncbi.nlm.nih.gov/sites/entrez?db=pubmed&cmd=search&term=21168126) |
|  | [hsa-let-7d*](http://www.mirbase.org/cgi-bin/mirna_entry.pl?acc=MI0000065) | [19528081](http://www.ncbi.nlm.nih.gov/sites/entrez?db=pubmed&cmd=search&term=19528081) |  | [hsa-miR-138](http://www.mirbase.org/cgi-bin/mirna_entry.pl?acc=MI0000476) | [21168126](http://www.ncbi.nlm.nih.gov/sites/entrez?db=pubmed&cmd=search&term=21168126) |
|  | [hsa-let-7d*](http://www.mirbase.org/cgi-bin/mirna_entry.pl?acc=MI0000065) | [20600201](http://www.ncbi.nlm.nih.gov/sites/entrez?db=pubmed&cmd=search&term=20600201) |  | [hsa-miR-138-2*](http://www.mirbase.org/cgi-bin/mirna_entry.pl?acc=MI0000455) | [21168126](http://www.ncbi.nlm.nih.gov/sites/entrez?db=pubmed&cmd=search&term=21168126) |
|  | [hsa-let-7e](http://www.mirbase.org/cgi-bin/mirna_entry.pl?acc=MI0000066) | [22081076](http://www.ncbi.nlm.nih.gov/sites/entrez?db=pubmed&cmd=search&term=22081076) |  | [hsa-miR-148a](http://www.mirbase.org/cgi-bin/mirna_entry.pl?acc=MI0000253) | [21168126](http://www.ncbi.nlm.nih.gov/sites/entrez?db=pubmed&cmd=search&term=21168126) |
|  | [hsa-let-7e](http://www.mirbase.org/cgi-bin/mirna_entry.pl?acc=MI0000066) | [19528081](http://www.ncbi.nlm.nih.gov/sites/entrez?db=pubmed&cmd=search&term=19528081) |  | [hsa-miR-148a*](http://www.mirbase.org/cgi-bin/mirna_entry.pl?acc=MI0000253) | [21168126](http://www.ncbi.nlm.nih.gov/sites/entrez?db=pubmed&cmd=search&term=21168126) |
|  | [hsa-let-7e*](http://www.mirbase.org/cgi-bin/mirna_entry.pl?acc=MI0000066) | [22081076](http://www.ncbi.nlm.nih.gov/sites/entrez?db=pubmed&cmd=search&term=22081076) |  | [hsa-miR-15a](http://www.mirbase.org/cgi-bin/mirna_entry.pl?acc=MI0000069) | [19416898](http://www.ncbi.nlm.nih.gov/sites/entrez?db=pubmed&cmd=search&term=19416898) |
|  | [hsa-let-7e*](http://www.mirbase.org/cgi-bin/mirna_entry.pl?acc=MI0000066) | [19528081](http://www.ncbi.nlm.nih.gov/sites/entrez?db=pubmed&cmd=search&term=19528081) |  | [hsa-miR-15a*](http://www.mirbase.org/cgi-bin/mirna_entry.pl?acc=MI0000069) | [19416898](http://www.ncbi.nlm.nih.gov/sites/entrez?db=pubmed&cmd=search&term=19416898) |
|  | [hsa-let-7f](http://www.mirbase.org/cgi-bin/mirna_entry.pl?acc=MI0000067) | [22081076](http://www.ncbi.nlm.nih.gov/sites/entrez?db=pubmed&cmd=search&term=22081076) |  | [hsa-miR-181a](http://www.mirbase.org/cgi-bin/mirna_entry.pl?acc=MI0000269) | [19703567](http://www.ncbi.nlm.nih.gov/sites/entrez?db=pubmed&cmd=search&term=19703567) |
|  | [hsa-let-7f](http://www.mirbase.org/cgi-bin/mirna_entry.pl?acc=MI0000068) | [22081076](http://www.ncbi.nlm.nih.gov/sites/entrez?db=pubmed&cmd=search&term=22081076) |  | [hsa-miR-181a](http://www.mirbase.org/cgi-bin/mirna_entry.pl?acc=MI0000289) | [19703567](http://www.ncbi.nlm.nih.gov/sites/entrez?db=pubmed&cmd=search&term=19703567) |
|  | [hsa-let-7f](http://www.mirbase.org/cgi-bin/mirna_entry.pl?acc=MI0000067) | [19528081](http://www.ncbi.nlm.nih.gov/sites/entrez?db=pubmed&cmd=search&term=19528081) |  | [hsa-miR-182](http://www.mirbase.org/cgi-bin/mirna_entry.pl?acc=MI0000272) | [19416898](http://www.ncbi.nlm.nih.gov/sites/entrez?db=pubmed&cmd=search&term=19416898) |
|  | [hsa-let-7f](http://www.mirbase.org/cgi-bin/mirna_entry.pl?acc=MI0000068) | [19528081](http://www.ncbi.nlm.nih.gov/sites/entrez?db=pubmed&cmd=search&term=19528081) |  | [hsa-miR-182*](http://www.mirbase.org/cgi-bin/mirna_entry.pl?acc=MI0000272) | [19416898](http://www.ncbi.nlm.nih.gov/sites/entrez?db=pubmed&cmd=search&term=19416898) |
|  | [hsa-let-7f-1*](http://www.mirbase.org/cgi-bin/mirna_entry.pl?acc=MI0000067) | [22081076](http://www.ncbi.nlm.nih.gov/sites/entrez?db=pubmed&cmd=search&term=22081076) |  | [hsa-miR-18a](http://www.mirbase.org/cgi-bin/mirna_entry.pl?acc=MI0000072) | [19416898](http://www.ncbi.nlm.nih.gov/sites/entrez?db=pubmed&cmd=search&term=19416898) |
|  | [hsa-let-7f-1*](http://www.mirbase.org/cgi-bin/mirna_entry.pl?acc=MI0000067) | [19528081](http://www.ncbi.nlm.nih.gov/sites/entrez?db=pubmed&cmd=search&term=19528081) |  | [hsa-miR-18a*](http://www.mirbase.org/cgi-bin/mirna_entry.pl?acc=MI0000072) | [19416898](http://www.ncbi.nlm.nih.gov/sites/entrez?db=pubmed&cmd=search&term=19416898) |
|  | [hsa-let-7f-2*](http://www.mirbase.org/cgi-bin/mirna_entry.pl?acc=MI0000068) | [19528081](http://www.ncbi.nlm.nih.gov/sites/entrez?db=pubmed&cmd=search&term=19528081) |  | [hsa-miR-191](http://www.mirbase.org/cgi-bin/mirna_entry.pl?acc=MI0000465) | [18632683](http://www.ncbi.nlm.nih.gov/sites/entrez?db=pubmed&cmd=search&term=18632683) |
|  | [hsa-let-7f-2*](http://www.mirbase.org/cgi-bin/mirna_entry.pl?acc=MI0000068) | [22081076](http://www.ncbi.nlm.nih.gov/sites/entrez?db=pubmed&cmd=search&term=22081076) |  | [hsa-miR-191*](http://www.mirbase.org/cgi-bin/mirna_entry.pl?acc=MI0000465) | [18632683](http://www.ncbi.nlm.nih.gov/sites/entrez?db=pubmed&cmd=search&term=18632683) |
|  | [hsa-let-7g](http://www.mirbase.org/cgi-bin/mirna_entry.pl?acc=MI0000433) | [19528081](http://www.ncbi.nlm.nih.gov/sites/entrez?db=pubmed&cmd=search&term=19528081) |  | [hsa-miR-195](http://www.mirbase.org/cgi-bin/mirna_entry.pl?acc=MI0000489) | [19121517](http://www.ncbi.nlm.nih.gov/sites/entrez?db=pubmed&cmd=search&term=19121517) |
|  | [hsa-let-7g](http://www.mirbase.org/cgi-bin/mirna_entry.pl?acc=MI0000433) | [22081076](http://www.ncbi.nlm.nih.gov/sites/entrez?db=pubmed&cmd=search&term=22081076) |  | [hsa-miR-195](http://www.mirbase.org/cgi-bin/mirna_entry.pl?acc=MI0000489) | [18632683](http://www.ncbi.nlm.nih.gov/sites/entrez?db=pubmed&cmd=search&term=18632683) |
|  | [hsa-let-7g*](http://www.mirbase.org/cgi-bin/mirna_entry.pl?acc=MI0000433) | [19528081](http://www.ncbi.nlm.nih.gov/sites/entrez?db=pubmed&cmd=search&term=19528081) |  | [hsa-miR-195*](http://www.mirbase.org/cgi-bin/mirna_entry.pl?acc=MI0000489) | [18632683](http://www.ncbi.nlm.nih.gov/sites/entrez?db=pubmed&cmd=search&term=18632683) |
|  | [hsa-let-7g*](http://www.mirbase.org/cgi-bin/mirna_entry.pl?acc=MI0000433) | [22081076](http://www.ncbi.nlm.nih.gov/sites/entrez?db=pubmed&cmd=search&term=22081076) |  | [hsa-miR-195*](http://www.mirbase.org/cgi-bin/mirna_entry.pl?acc=MI0000489) | [19121517](http://www.ncbi.nlm.nih.gov/sites/entrez?db=pubmed&cmd=search&term=19121517) |
|  | [hsa-let-7i](http://www.mirbase.org/cgi-bin/mirna_entry.pl?acc=MI0000434) | [19528081](http://www.ncbi.nlm.nih.gov/sites/entrez?db=pubmed&cmd=search&term=19528081) |  | [hsa-miR-19a](http://www.mirbase.org/cgi-bin/mirna_entry.pl?acc=MI0000073) | [14697198](http://www.ncbi.nlm.nih.gov/sites/entrez?db=pubmed&cmd=search&term=14697198) |
|  | [hsa-let-7i](http://www.mirbase.org/cgi-bin/mirna_entry.pl?acc=MI0000434) | [22081076](http://www.ncbi.nlm.nih.gov/sites/entrez?db=pubmed&cmd=search&term=22081076) |  | [hsa-miR-19a*](http://www.mirbase.org/cgi-bin/mirna_entry.pl?acc=MI0000073) | [14697198](http://www.ncbi.nlm.nih.gov/sites/entrez?db=pubmed&cmd=search&term=14697198) |
|  | [hsa-let-7i*](http://www.mirbase.org/cgi-bin/mirna_entry.pl?acc=MI0000434) | [19528081](http://www.ncbi.nlm.nih.gov/sites/entrez?db=pubmed&cmd=search&term=19528081) |  | [hsa-miR-210](http://www.mirbase.org/cgi-bin/mirna_entry.pl?acc=MI0000286) | [19826008](http://www.ncbi.nlm.nih.gov/sites/entrez?db=pubmed&cmd=search&term=19826008) |
|  | [hsa-let-7i*](http://www.mirbase.org/cgi-bin/mirna_entry.pl?acc=MI0000434) | [22081076](http://www.ncbi.nlm.nih.gov/sites/entrez?db=pubmed&cmd=search&term=22081076) |  | [hsa-miR-212](http://www.mirbase.org/cgi-bin/mirna_entry.pl?acc=MI0000288) | [20711185](http://www.ncbi.nlm.nih.gov/sites/entrez?db=pubmed&cmd=search&term=20711185) |
|  | [hsa-miR-100](http://www.mirbase.org/cgi-bin/mirna_entry.pl?acc=MI0000102) | [20078964](http://www.ncbi.nlm.nih.gov/sites/entrez?db=pubmed&cmd=search&term=20078964) |  | [hsa-miR-212](http://www.mirbase.org/cgi-bin/mirna_entry.pl?acc=MI0000288) | [20307261](http://www.ncbi.nlm.nih.gov/sites/entrez?db=pubmed&cmd=search&term=20307261) |
|  | [hsa-miR-100*](http://www.mirbase.org/cgi-bin/mirna_entry.pl?acc=MI0000102) | [20078964](http://www.ncbi.nlm.nih.gov/sites/entrez?db=pubmed&cmd=search&term=20078964) |  | [hsa-miR-22](http://www.mirbase.org/cgi-bin/mirna_entry.pl?acc=MI0000078) | [21168126](http://www.ncbi.nlm.nih.gov/sites/entrez?db=pubmed&cmd=search&term=21168126) |
|  | [hsa-miR-107](http://www.mirbase.org/cgi-bin/mirna_entry.pl?acc=MI0000114) | [20348243](http://www.ncbi.nlm.nih.gov/sites/entrez?db=pubmed&cmd=search&term=20348243) |  | [hsa-miR-22*](http://www.mirbase.org/cgi-bin/mirna_entry.pl?acc=MI0000078) | [21168126](http://www.ncbi.nlm.nih.gov/sites/entrez?db=pubmed&cmd=search&term=21168126) |
|  | [hsa-miR-122](http://www.mirbase.org/cgi-bin/mirna_entry.pl?acc=MI0000442) | [20600201](http://www.ncbi.nlm.nih.gov/sites/entrez?db=pubmed&cmd=search&term=20600201) |  | [hsa-miR-23a](http://www.mirbase.org/cgi-bin/mirna_entry.pl?acc=MI0000079) | [14697198](http://www.ncbi.nlm.nih.gov/sites/entrez?db=pubmed&cmd=search&term=14697198) |
|  | [hsa-miR-122*](http://www.mirbase.org/cgi-bin/mirna_entry.pl?acc=MI0000442) | [20600201](http://www.ncbi.nlm.nih.gov/sites/entrez?db=pubmed&cmd=search&term=20600201) |  | [hsa-miR-23a*](http://www.mirbase.org/cgi-bin/mirna_entry.pl?acc=MI0000079) | [14697198](http://www.ncbi.nlm.nih.gov/sites/entrez?db=pubmed&cmd=search&term=14697198) |
|  | [hsa-miR-125b](http://www.mirbase.org/cgi-bin/mirna_entry.pl?acc=MI0000446) | [20978686](http://www.ncbi.nlm.nih.gov/sites/entrez?db=pubmed&cmd=search&term=20978686) |  | [hsa-miR-23b](http://www.mirbase.org/cgi-bin/mirna_entry.pl?acc=MI0000439) | [14697198](http://www.ncbi.nlm.nih.gov/sites/entrez?db=pubmed&cmd=search&term=14697198) |
|  | [hsa-miR-125b](http://www.mirbase.org/cgi-bin/mirna_entry.pl?acc=MI0000470) | [20978686](http://www.ncbi.nlm.nih.gov/sites/entrez?db=pubmed&cmd=search&term=20978686) |  | [hsa-miR-23b*](http://www.mirbase.org/cgi-bin/mirna_entry.pl?acc=MI0000439) | [14697198](http://www.ncbi.nlm.nih.gov/sites/entrez?db=pubmed&cmd=search&term=14697198) |
|  | [hsa-miR-130a](http://www.mirbase.org/cgi-bin/mirna_entry.pl?acc=MI0000448) | [21712254](http://www.ncbi.nlm.nih.gov/sites/entrez?db=pubmed&cmd=search&term=21712254) |  | [hsa-miR-26a](http://www.mirbase.org/cgi-bin/mirna_entry.pl?acc=MI0000083) | [14697198](http://www.ncbi.nlm.nih.gov/sites/entrez?db=pubmed&cmd=search&term=14697198) |
|  | [hsa-miR-130a*](http://www.mirbase.org/cgi-bin/mirna_entry.pl?acc=MI0000448) | [21712254](http://www.ncbi.nlm.nih.gov/sites/entrez?db=pubmed&cmd=search&term=21712254) |  | [hsa-miR-26a](http://www.mirbase.org/cgi-bin/mirna_entry.pl?acc=MI0000750) | [14697198](http://www.ncbi.nlm.nih.gov/sites/entrez?db=pubmed&cmd=search&term=14697198) |
|  | [hsa-miR-145](http://www.mirbase.org/cgi-bin/mirna_entry.pl?acc=MI0000461) | [19730444](http://www.ncbi.nlm.nih.gov/sites/entrez?db=pubmed&cmd=search&term=19730444) |  | [hsa-miR-30a](http://www.mirbase.org/cgi-bin/mirna_entry.pl?acc=MI0000088) | [18632683](http://www.ncbi.nlm.nih.gov/sites/entrez?db=pubmed&cmd=search&term=18632683) |
|  | [hsa-miR-145*](http://www.mirbase.org/cgi-bin/mirna_entry.pl?acc=MI0000461) | [19730444](http://www.ncbi.nlm.nih.gov/sites/entrez?db=pubmed&cmd=search&term=19730444) |  | [hsa-miR-30a](http://www.mirbase.org/cgi-bin/mirna_entry.pl?acc=MI0000088) | [23358924](http://www.ncbi.nlm.nih.gov/sites/entrez?db=pubmed&cmd=search&term=23358924) |
|  | [hsa-miR-146a](http://www.mirbase.org/cgi-bin/mirna_entry.pl?acc=MI0000477) | [22019503](http://www.ncbi.nlm.nih.gov/sites/entrez?db=pubmed&cmd=search&term=22019503) |  | [hsa-miR-30a*](http://www.mirbase.org/cgi-bin/mirna_entry.pl?acc=MI0000088) | [18632683](http://www.ncbi.nlm.nih.gov/sites/entrez?db=pubmed&cmd=search&term=18632683) |
|  | [hsa-miR-146a*](http://www.mirbase.org/cgi-bin/mirna_entry.pl?acc=MI0000477) | [22019503](http://www.ncbi.nlm.nih.gov/sites/entrez?db=pubmed&cmd=search&term=22019503) |  | [hsa-miR-30a*](http://www.mirbase.org/cgi-bin/mirna_entry.pl?acc=MI0000088) | [23358924](http://www.ncbi.nlm.nih.gov/sites/entrez?db=pubmed&cmd=search&term=23358924) |
|  | [hsa-miR-17](http://www.mirbase.org/cgi-bin/mirna_entry.pl?acc=MI0000071) | [19706389](http://www.ncbi.nlm.nih.gov/sites/entrez?db=pubmed&cmd=search&term=19706389) |  | [hsa-miR-30b](http://www.mirbase.org/cgi-bin/mirna_entry.pl?acc=MI0000441) | [19416898](http://www.ncbi.nlm.nih.gov/sites/entrez?db=pubmed&cmd=search&term=19416898) |
|  | [hsa-miR-17](http://www.mirbase.org/cgi-bin/mirna_entry.pl?acc=MI0000071) | [20080637](http://www.ncbi.nlm.nih.gov/sites/entrez?db=pubmed&cmd=search&term=20080637) |  | [hsa-miR-30b*](http://www.mirbase.org/cgi-bin/mirna_entry.pl?acc=MI0000441) | [19416898](http://www.ncbi.nlm.nih.gov/sites/entrez?db=pubmed&cmd=search&term=19416898) |
|  | [hsa-miR-17*](http://www.mirbase.org/cgi-bin/mirna_entry.pl?acc=MI0000071) | [19706389](http://www.ncbi.nlm.nih.gov/sites/entrez?db=pubmed&cmd=search&term=19706389) |  | [hsa-miR-339-3p](http://www.mirbase.org/cgi-bin/mirna_entry.pl?acc=MI0000815) | [21168126](http://www.ncbi.nlm.nih.gov/sites/entrez?db=pubmed&cmd=search&term=21168126) |
|  | [hsa-miR-17*](http://www.mirbase.org/cgi-bin/mirna_entry.pl?acc=MI0000071) | [20080637](http://www.ncbi.nlm.nih.gov/sites/entrez?db=pubmed&cmd=search&term=20080637) |  | [hsa-miR-339-5p](http://www.mirbase.org/cgi-bin/mirna_entry.pl?acc=MI0000815) | [21168126](http://www.ncbi.nlm.nih.gov/sites/entrez?db=pubmed&cmd=search&term=21168126) |
|  | [hsa-miR-181a](http://www.mirbase.org/cgi-bin/mirna_entry.pl?acc=MI0000289) | [19684618](http://www.ncbi.nlm.nih.gov/sites/entrez?db=pubmed&cmd=search&term=19684618) |  | [hsa-miR-34a](http://www.mirbase.org/cgi-bin/mirna_entry.pl?acc=MI0000268) | [14697198](http://www.ncbi.nlm.nih.gov/sites/entrez?db=pubmed&cmd=search&term=14697198) |
|  | [hsa-miR-181a](http://www.mirbase.org/cgi-bin/mirna_entry.pl?acc=MI0000269) | [19684618](http://www.ncbi.nlm.nih.gov/sites/entrez?db=pubmed&cmd=search&term=19684618) |  | [hsa-miR-34a*](http://www.mirbase.org/cgi-bin/mirna_entry.pl?acc=MI0000268) | [14697198](http://www.ncbi.nlm.nih.gov/sites/entrez?db=pubmed&cmd=search&term=14697198) |
|  | [hsa-miR-181b](http://www.mirbase.org/cgi-bin/mirna_entry.pl?acc=MI0000270) | [19684618](http://www.ncbi.nlm.nih.gov/sites/entrez?db=pubmed&cmd=search&term=19684618) |  | [hsa-miR-375](http://www.mirbase.org/cgi-bin/mirna_entry.pl?acc=MI0000783) | [20584986](http://www.ncbi.nlm.nih.gov/sites/entrez?db=pubmed&cmd=search&term=20584986) |
|  | [hsa-miR-181b](http://www.mirbase.org/cgi-bin/mirna_entry.pl?acc=MI0000683) | [19684618](http://www.ncbi.nlm.nih.gov/sites/entrez?db=pubmed&cmd=search&term=19684618) |  | [hsa-miR-488](http://www.mirbase.org/cgi-bin/mirna_entry.pl?acc=MI0003123) | [21168126](http://www.ncbi.nlm.nih.gov/sites/entrez?db=pubmed&cmd=search&term=21168126) |
|  | [hsa-miR-181b](http://www.mirbase.org/cgi-bin/mirna_entry.pl?acc=MI0000270) | [20347265](http://www.ncbi.nlm.nih.gov/sites/entrez?db=pubmed&cmd=search&term=20347265) |  | [hsa-miR-488*](http://www.mirbase.org/cgi-bin/mirna_entry.pl?acc=MI0003123) | [21168126](http://www.ncbi.nlm.nih.gov/sites/entrez?db=pubmed&cmd=search&term=21168126) |
|  | [hsa-miR-181b](http://www.mirbase.org/cgi-bin/mirna_entry.pl?acc=MI0000683) | [20347265](http://www.ncbi.nlm.nih.gov/sites/entrez?db=pubmed&cmd=search&term=20347265) |  | [hsa-miR-491-3p](http://www.mirbase.org/cgi-bin/mirna_entry.pl?acc=MI0003126) | [21168126](http://www.ncbi.nlm.nih.gov/sites/entrez?db=pubmed&cmd=search&term=21168126) |
|  | [hsa-miR-18a](http://www.mirbase.org/cgi-bin/mirna_entry.pl?acc=MI0000072) | [19706389](http://www.ncbi.nlm.nih.gov/sites/entrez?db=pubmed&cmd=search&term=19706389) |  | [hsa-miR-491-5p](http://www.mirbase.org/cgi-bin/mirna_entry.pl?acc=MI0003126) | [21168126](http://www.ncbi.nlm.nih.gov/sites/entrez?db=pubmed&cmd=search&term=21168126) |
|  | [hsa-miR-18a](http://www.mirbase.org/cgi-bin/mirna_entry.pl?acc=MI0000072) | [19027010](http://www.ncbi.nlm.nih.gov/sites/entrez?db=pubmed&cmd=search&term=19027010) |  | [hsa-miR-495](http://www.mirbase.org/cgi-bin/mirna_entry.pl?acc=MI0003135) | [18632683](http://www.ncbi.nlm.nih.gov/sites/entrez?db=pubmed&cmd=search&term=18632683) |
|  | [hsa-miR-18a](http://www.mirbase.org/cgi-bin/mirna_entry.pl?acc=MI0000072) | [19684618](http://www.ncbi.nlm.nih.gov/sites/entrez?db=pubmed&cmd=search&term=19684618) |  | [hsa-miR-9](http://www.mirbase.org/cgi-bin/mirna_entry.pl?acc=MI0000466) | [19958814](http://www.ncbi.nlm.nih.gov/sites/entrez?db=pubmed&cmd=search&term=19958814) |
|  | [hsa-miR-18a](http://www.mirbase.org/cgi-bin/mirna_entry.pl?acc=MI0000072) | [20080637](http://www.ncbi.nlm.nih.gov/sites/entrez?db=pubmed&cmd=search&term=20080637) |  | [hsa-miR-9](http://www.mirbase.org/cgi-bin/mirna_entry.pl?acc=MI0000467) | [19958814](http://www.ncbi.nlm.nih.gov/sites/entrez?db=pubmed&cmd=search&term=19958814) |
|  | [hsa-miR-18a*](http://www.mirbase.org/cgi-bin/mirna_entry.pl?acc=MI0000072) | [19706389](http://www.ncbi.nlm.nih.gov/sites/entrez?db=pubmed&cmd=search&term=19706389) |  | [hsa-miR-9](http://www.mirbase.org/cgi-bin/mirna_entry.pl?acc=MI0000468) | [19958814](http://www.ncbi.nlm.nih.gov/sites/entrez?db=pubmed&cmd=search&term=19958814) |
|  | [hsa-miR-18a*](http://www.mirbase.org/cgi-bin/mirna_entry.pl?acc=MI0000072) | [19027010](http://www.ncbi.nlm.nih.gov/sites/entrez?db=pubmed&cmd=search&term=19027010) |  | [hsa-miR-99a](http://www.mirbase.org/cgi-bin/mirna_entry.pl?acc=MI0000101) | [19416898](http://www.ncbi.nlm.nih.gov/sites/entrez?db=pubmed&cmd=search&term=19416898) |
|  | [hsa-miR-18a*](http://www.mirbase.org/cgi-bin/mirna_entry.pl?acc=MI0000072) | [19684618](http://www.ncbi.nlm.nih.gov/sites/entrez?db=pubmed&cmd=search&term=19684618) |  | [hsa-miR-99a*](http://www.mirbase.org/cgi-bin/mirna_entry.pl?acc=MI0000101) | [19416898](http://www.ncbi.nlm.nih.gov/sites/entrez?db=pubmed&cmd=search&term=19416898) |
|  | [hsa-miR-18a*](http://www.mirbase.org/cgi-bin/mirna_entry.pl?acc=MI0000072) | [20080637](http://www.ncbi.nlm.nih.gov/sites/entrez?db=pubmed&cmd=search&term=20080637) | [ATOH1](http://www.ncbi.nlm.nih.gov/sites/entrez?Db=gene&Cmd=ShowDetailView&TermToSearch=474&ordinalpos=1&itool=EntrezSystem2.PEntrez.Gene.Gene_ResultsPanel.Gene_RVDocSum) | [hsa-miR-182](http://www.mirbase.org/cgi-bin/mirna_entry.pl?acc=MI0000272) | [21360794](http://www.ncbi.nlm.nih.gov/sites/entrez?db=pubmed&cmd=search&term=21360794) |
|  | [hsa-miR-18b](http://www.mirbase.org/cgi-bin/mirna_entry.pl?acc=MI0001518) | [19684618](http://www.ncbi.nlm.nih.gov/sites/entrez?db=pubmed&cmd=search&term=19684618) |  | [hsa-miR-182*](http://www.mirbase.org/cgi-bin/mirna_entry.pl?acc=MI0000272) | [21360794](http://www.ncbi.nlm.nih.gov/sites/entrez?db=pubmed&cmd=search&term=21360794) |
|  | [hsa-miR-18b*](http://www.mirbase.org/cgi-bin/mirna_entry.pl?acc=MI0001518) | [19684618](http://www.ncbi.nlm.nih.gov/sites/entrez?db=pubmed&cmd=search&term=19684618) |  | [hsa-miR-183](http://www.mirbase.org/cgi-bin/mirna_entry.pl?acc=MI0000273) | [18427387](http://www.ncbi.nlm.nih.gov/sites/entrez?db=pubmed&cmd=search&term=18427387) |
|  | [hsa-miR-193b](http://www.mirbase.org/cgi-bin/mirna_entry.pl?acc=MI0003137) | [19684618](http://www.ncbi.nlm.nih.gov/sites/entrez?db=pubmed&cmd=search&term=19684618) |  | [hsa-miR-183](http://www.mirbase.org/cgi-bin/mirna_entry.pl?acc=MI0000273) | [21360794](http://www.ncbi.nlm.nih.gov/sites/entrez?db=pubmed&cmd=search&term=21360794) |
|  | [hsa-miR-193b*](http://www.mirbase.org/cgi-bin/mirna_entry.pl?acc=MI0003137) | [19684618](http://www.ncbi.nlm.nih.gov/sites/entrez?db=pubmed&cmd=search&term=19684618) |  | [hsa-miR-183*](http://www.mirbase.org/cgi-bin/mirna_entry.pl?acc=MI0000273) | [18427387](http://www.ncbi.nlm.nih.gov/sites/entrez?db=pubmed&cmd=search&term=18427387) |
|  | [hsa-miR-196b](http://www.mirbase.org/cgi-bin/mirna_entry.pl?acc=MI0001150) | [18096665](http://www.ncbi.nlm.nih.gov/sites/entrez?db=pubmed&cmd=search&term=18096665) |  | [hsa-miR-183*](http://www.mirbase.org/cgi-bin/mirna_entry.pl?acc=MI0000273) | [21360794](http://www.ncbi.nlm.nih.gov/sites/entrez?db=pubmed&cmd=search&term=21360794) |
|  | [hsa-miR-198](http://www.mirbase.org/cgi-bin/mirna_entry.pl?acc=MI0000240) | [20347265](http://www.ncbi.nlm.nih.gov/sites/entrez?db=pubmed&cmd=search&term=20347265) |  | [hsa-miR-96](http://www.mirbase.org/cgi-bin/mirna_entry.pl?acc=MI0000098) | [21360794](http://www.ncbi.nlm.nih.gov/sites/entrez?db=pubmed&cmd=search&term=21360794) |
|  | [hsa-miR-19a](http://www.mirbase.org/cgi-bin/mirna_entry.pl?acc=MI0000073) | [20080637](http://www.ncbi.nlm.nih.gov/sites/entrez?db=pubmed&cmd=search&term=20080637) |  | [hsa-miR-96*](http://www.mirbase.org/cgi-bin/mirna_entry.pl?acc=MI0000098) | [21360794](http://www.ncbi.nlm.nih.gov/sites/entrez?db=pubmed&cmd=search&term=21360794) |
|  | [hsa-miR-19a*](http://www.mirbase.org/cgi-bin/mirna_entry.pl?acc=MI0000073) | [20080637](http://www.ncbi.nlm.nih.gov/sites/entrez?db=pubmed&cmd=search&term=20080637) | [THRB](http://www.ncbi.nlm.nih.gov/sites/entrez?Db=gene&Cmd=ShowDetailView&TermToSearch=7068&ordinalpos=1&itool=EntrezSystem2.PEntrez.Gene.Gene_ResultsPanel.Gene_RVDocSum) | [hsa-miR-181a](http://www.mirbase.org/cgi-bin/mirna_entry.pl?acc=MI0000289) | [21159845](http://www.ncbi.nlm.nih.gov/sites/entrez?db=pubmed&cmd=search&term=21159845) |
|  | [hsa-miR-19b](http://www.mirbase.org/cgi-bin/mirna_entry.pl?acc=MI0000074) | [19706389](http://www.ncbi.nlm.nih.gov/sites/entrez?db=pubmed&cmd=search&term=19706389) |  | [hsa-miR-181a*](http://www.mirbase.org/cgi-bin/mirna_entry.pl?acc=MI0000289) | [21159845](http://www.ncbi.nlm.nih.gov/sites/entrez?db=pubmed&cmd=search&term=21159845) |
|  | [hsa-miR-19b](http://www.mirbase.org/cgi-bin/mirna_entry.pl?acc=MI0000075) | [19706389](http://www.ncbi.nlm.nih.gov/sites/entrez?db=pubmed&cmd=search&term=19706389) |  | [hsa-miR-204](http://www.mirbase.org/cgi-bin/mirna_entry.pl?acc=MI0000284) | [20691260](http://www.ncbi.nlm.nih.gov/sites/entrez?db=pubmed&cmd=search&term=20691260) |
|  | [hsa-miR-206](http://www.mirbase.org/cgi-bin/mirna_entry.pl?acc=MI0000490) | [19684618](http://www.ncbi.nlm.nih.gov/sites/entrez?db=pubmed&cmd=search&term=19684618) |  | [hsa-miR-21](http://www.mirbase.org/cgi-bin/mirna_entry.pl?acc=MI0000077) | [21159845](http://www.ncbi.nlm.nih.gov/sites/entrez?db=pubmed&cmd=search&term=21159845) |
|  | [hsa-miR-206](http://www.mirbase.org/cgi-bin/mirna_entry.pl?acc=MI0000490) | [20388878](http://www.ncbi.nlm.nih.gov/sites/entrez?db=pubmed&cmd=search&term=20388878) |  | [hsa-miR-21*](http://www.mirbase.org/cgi-bin/mirna_entry.pl?acc=MI0000077) | [21159845](http://www.ncbi.nlm.nih.gov/sites/entrez?db=pubmed&cmd=search&term=21159845) |
|  | [hsa-miR-206](http://www.mirbase.org/cgi-bin/mirna_entry.pl?acc=MI0000490) | [18593897](http://www.ncbi.nlm.nih.gov/sites/entrez?db=pubmed&cmd=search&term=18593897) |  | [hsa-miR-221](http://www.mirbase.org/cgi-bin/mirna_entry.pl?acc=MI0000298) | [21159845](http://www.ncbi.nlm.nih.gov/sites/entrez?db=pubmed&cmd=search&term=21159845) |
|  | [hsa-miR-206](http://www.mirbase.org/cgi-bin/mirna_entry.pl?acc=MI0000490) | [21983130](http://www.ncbi.nlm.nih.gov/sites/entrez?db=pubmed&cmd=search&term=21983130) |  | [hsa-miR-221*](http://www.mirbase.org/cgi-bin/mirna_entry.pl?acc=MI0000298) | [21159845](http://www.ncbi.nlm.nih.gov/sites/entrez?db=pubmed&cmd=search&term=21159845) |
|  | [hsa-miR-206](http://www.mirbase.org/cgi-bin/mirna_entry.pl?acc=MI0000490) | [17312270](http://www.ncbi.nlm.nih.gov/sites/entrez?db=pubmed&cmd=search&term=17312270) | [TLE1](http://www.ncbi.nlm.nih.gov/sites/entrez?Db=gene&Cmd=ShowDetailView&TermToSearch=7088&ordinalpos=1&itool=EntrezSystem2.PEntrez.Gene.Gene_ResultsPanel.Gene_RVDocSum) | [hsa-miR-125a-3p](http://www.mirbase.org/cgi-bin/mirna_entry.pl?acc=MI0000469) | [20937217](http://www.ncbi.nlm.nih.gov/sites/entrez?db=pubmed&cmd=search&term=20937217) |
|  | [hsa-miR-206](http://www.mirbase.org/cgi-bin/mirna_entry.pl?acc=MI0000490) | [20347265](http://www.ncbi.nlm.nih.gov/sites/entrez?db=pubmed&cmd=search&term=20347265) |  | [hsa-miR-125a-5p](http://www.mirbase.org/cgi-bin/mirna_entry.pl?acc=MI0000469) | [20937217](http://www.ncbi.nlm.nih.gov/sites/entrez?db=pubmed&cmd=search&term=20937217) |
|  | [hsa-miR-20b](http://www.mirbase.org/cgi-bin/mirna_entry.pl?acc=MI0001519) | [19706389](http://www.ncbi.nlm.nih.gov/sites/entrez?db=pubmed&cmd=search&term=19706389) |  | [hsa-miR-150](http://www.mirbase.org/cgi-bin/mirna_entry.pl?acc=MI0000479) | [20937217](http://www.ncbi.nlm.nih.gov/sites/entrez?db=pubmed&cmd=search&term=20937217) |
|  | [hsa-miR-20b*](http://www.mirbase.org/cgi-bin/mirna_entry.pl?acc=MI0001519) | [19706389](http://www.ncbi.nlm.nih.gov/sites/entrez?db=pubmed&cmd=search&term=19706389) |  | [hsa-miR-150*](http://www.mirbase.org/cgi-bin/mirna_entry.pl?acc=MI0000479) | [20937217](http://www.ncbi.nlm.nih.gov/sites/entrez?db=pubmed&cmd=search&term=20937217) |
|  | [hsa-miR-21](http://www.mirbase.org/cgi-bin/mirna_entry.pl?acc=MI0000077) | [19528081](http://www.ncbi.nlm.nih.gov/sites/entrez?db=pubmed&cmd=search&term=19528081) |  |  |  |
|  | [hsa-miR-21*](http://www.mirbase.org/cgi-bin/mirna_entry.pl?acc=MI0000077) | [19528081](http://www.ncbi.nlm.nih.gov/sites/entrez?db=pubmed&cmd=search&term=19528081) | BARHL1 |  |  |
|  | [hsa-miR-22](http://www.mirbase.org/cgi-bin/mirna_entry.pl?acc=MI0000078) | [18347104](http://www.ncbi.nlm.nih.gov/sites/entrez?db=pubmed&cmd=search&term=18347104) | TH |  |  |
|  | [hsa-miR-22](http://www.mirbase.org/cgi-bin/mirna_entry.pl?acc=MI0000078) | [18347134](http://www.ncbi.nlm.nih.gov/sites/entrez?db=pubmed&cmd=search&term=18347134) | PTF1A | NO RESULT |  |
|  | [hsa-miR-22](http://www.mirbase.org/cgi-bin/mirna_entry.pl?acc=MI0000078) | [20180843](http://www.ncbi.nlm.nih.gov/sites/entrez?db=pubmed&cmd=search&term=20180843) | NTF3 |  |  |
|  | [hsa-miR-22](http://www.mirbase.org/cgi-bin/mirna_entry.pl?acc=MI0000078) | [19414598](http://www.ncbi.nlm.nih.gov/sites/entrez?db=pubmed&cmd=search&term=19414598) |  |  |  |
|  | [hsa-miR-22](http://www.mirbase.org/cgi-bin/mirna_entry.pl?acc=MI0000078) | [20600201](http://www.ncbi.nlm.nih.gov/sites/entrez?db=pubmed&cmd=search&term=20600201) |  |  |  |
|  | [hsa-miR-22*](http://www.mirbase.org/cgi-bin/mirna_entry.pl?acc=MI0000078) | [18347134](http://www.ncbi.nlm.nih.gov/sites/entrez?db=pubmed&cmd=search&term=18347134) |  |  |  |
|  | [hsa-miR-22*](http://www.mirbase.org/cgi-bin/mirna_entry.pl?acc=MI0000078) | [20180843](http://www.ncbi.nlm.nih.gov/sites/entrez?db=pubmed&cmd=search&term=20180843) |  |  |  |
|  | [hsa-miR-22*](http://www.mirbase.org/cgi-bin/mirna_entry.pl?acc=MI0000078) | [19414598](http://www.ncbi.nlm.nih.gov/sites/entrez?db=pubmed&cmd=search&term=19414598) |  |  |  |
|  | [hsa-miR-22*](http://www.mirbase.org/cgi-bin/mirna_entry.pl?acc=MI0000078) | [20600201](http://www.ncbi.nlm.nih.gov/sites/entrez?db=pubmed&cmd=search&term=20600201) |  |  |  |
|  | [hsa-miR-22*](http://www.mirbase.org/cgi-bin/mirna_entry.pl?acc=MI0000078) | [18347104](http://www.ncbi.nlm.nih.gov/sites/entrez?db=pubmed&cmd=search&term=18347104) |  |  |  |
|  | [hsa-miR-221](http://www.mirbase.org/cgi-bin/mirna_entry.pl?acc=MI0000298) | [22527538](http://www.ncbi.nlm.nih.gov/sites/entrez?db=pubmed&cmd=search&term=22527538) |  |  |  |
|  | [hsa-miR-221](http://www.mirbase.org/cgi-bin/mirna_entry.pl?acc=MI0000298) | [18790736](http://www.ncbi.nlm.nih.gov/sites/entrez?db=pubmed&cmd=search&term=18790736) |  |  |  |
|  | [hsa-miR-221](http://www.mirbase.org/cgi-bin/mirna_entry.pl?acc=MI0000298) | [21057537](http://www.ncbi.nlm.nih.gov/sites/entrez?db=pubmed&cmd=search&term=21057537) |  |  |  |
|  | [hsa-miR-221](http://www.mirbase.org/cgi-bin/mirna_entry.pl?acc=MI0000298) | [20388878](http://www.ncbi.nlm.nih.gov/sites/entrez?db=pubmed&cmd=search&term=20388878) |  |  |  |
|  | [hsa-miR-221*](http://www.mirbase.org/cgi-bin/mirna_entry.pl?acc=MI0000298) | [22527538](http://www.ncbi.nlm.nih.gov/sites/entrez?db=pubmed&cmd=search&term=22527538) |  |  |  |
|  | [hsa-miR-221*](http://www.mirbase.org/cgi-bin/mirna_entry.pl?acc=MI0000298) | [18790736](http://www.ncbi.nlm.nih.gov/sites/entrez?db=pubmed&cmd=search&term=18790736) |  |  |  |
|  | [hsa-miR-221*](http://www.mirbase.org/cgi-bin/mirna_entry.pl?acc=MI0000298) | [21057537](http://www.ncbi.nlm.nih.gov/sites/entrez?db=pubmed&cmd=search&term=21057537) |  |  |  |
|  | [hsa-miR-221*](http://www.mirbase.org/cgi-bin/mirna_entry.pl?acc=MI0000298) | [20388878](http://www.ncbi.nlm.nih.gov/sites/entrez?db=pubmed&cmd=search&term=20388878) |  |  |  |
|  | [hsa-miR-222](http://www.mirbase.org/cgi-bin/mirna_entry.pl?acc=MI0000299) | [21057537](http://www.ncbi.nlm.nih.gov/sites/entrez?db=pubmed&cmd=search&term=21057537) |  |  |  |
|  | [hsa-miR-222](http://www.mirbase.org/cgi-bin/mirna_entry.pl?acc=MI0000299) | [20388878](http://www.ncbi.nlm.nih.gov/sites/entrez?db=pubmed&cmd=search&term=20388878) |  |  |  |
|  | [hsa-miR-222](http://www.mirbase.org/cgi-bin/mirna_entry.pl?acc=MI0000299) | [22527538](http://www.ncbi.nlm.nih.gov/sites/entrez?db=pubmed&cmd=search&term=22527538) |  |  |  |
|  | [hsa-miR-222](http://www.mirbase.org/cgi-bin/mirna_entry.pl?acc=MI0000299) | [18790736](http://www.ncbi.nlm.nih.gov/sites/entrez?db=pubmed&cmd=search&term=18790736) |  |  |  |
|  | [hsa-miR-222*](http://www.mirbase.org/cgi-bin/mirna_entry.pl?acc=MI0000299) | [21057537](http://www.ncbi.nlm.nih.gov/sites/entrez?db=pubmed&cmd=search&term=21057537) |  |  |  |
|  | [hsa-miR-222*](http://www.mirbase.org/cgi-bin/mirna_entry.pl?acc=MI0000299) | [20388878](http://www.ncbi.nlm.nih.gov/sites/entrez?db=pubmed&cmd=search&term=20388878) |  |  |  |
|  | [hsa-miR-222*](http://www.mirbase.org/cgi-bin/mirna_entry.pl?acc=MI0000299) | [22527538](http://www.ncbi.nlm.nih.gov/sites/entrez?db=pubmed&cmd=search&term=22527538) |  |  |  |
|  | [hsa-miR-222*](http://www.mirbase.org/cgi-bin/mirna_entry.pl?acc=MI0000299) | [18790736](http://www.ncbi.nlm.nih.gov/sites/entrez?db=pubmed&cmd=search&term=18790736) |  |  |  |
|  | [hsa-miR-24](http://www.mirbase.org/cgi-bin/mirna_entry.pl?acc=MI0000080) | [20347265](http://www.ncbi.nlm.nih.gov/sites/entrez?db=pubmed&cmd=search&term=20347265) |  |  |  |
|  | [hsa-miR-24](http://www.mirbase.org/cgi-bin/mirna_entry.pl?acc=MI0000081) | [20347265](http://www.ncbi.nlm.nih.gov/sites/entrez?db=pubmed&cmd=search&term=20347265) |  |  |  |
|  | [hsa-miR-24-1*](http://www.mirbase.org/cgi-bin/mirna_entry.pl?acc=MI0000080) | [20347265](http://www.ncbi.nlm.nih.gov/sites/entrez?db=pubmed&cmd=search&term=20347265) |  |  |  |
|  | [hsa-miR-24-2*](http://www.mirbase.org/cgi-bin/mirna_entry.pl?acc=MI0000081) | [20347265](http://www.ncbi.nlm.nih.gov/sites/entrez?db=pubmed&cmd=search&term=20347265) |  |  |  |
|  | [hsa-miR-26b](http://www.mirbase.org/cgi-bin/mirna_entry.pl?acc=MI0000084) | [20347265](http://www.ncbi.nlm.nih.gov/sites/entrez?db=pubmed&cmd=search&term=20347265) |  |  |  |
|  | [hsa-miR-26b*](http://www.mirbase.org/cgi-bin/mirna_entry.pl?acc=MI0000084) | [20347265](http://www.ncbi.nlm.nih.gov/sites/entrez?db=pubmed&cmd=search&term=20347265) |  |  |  |
|  | [hsa-miR-27a](http://www.mirbase.org/cgi-bin/mirna_entry.pl?acc=MI0000085) | [20382698](http://www.ncbi.nlm.nih.gov/sites/entrez?db=pubmed&cmd=search&term=20382698) |  |  |  |
|  | [hsa-miR-27a*](http://www.mirbase.org/cgi-bin/mirna_entry.pl?acc=MI0000085) | [20382698](http://www.ncbi.nlm.nih.gov/sites/entrez?db=pubmed&cmd=search&term=20382698) |  |  |  |
|  | [hsa-miR-29b](http://www.mirbase.org/cgi-bin/mirna_entry.pl?acc=MI0000105) | [19211935](http://www.ncbi.nlm.nih.gov/sites/entrez?db=pubmed&cmd=search&term=19211935) |  |  |  |
|  | [hsa-miR-29b](http://www.mirbase.org/cgi-bin/mirna_entry.pl?acc=MI0000107) | [19211935](http://www.ncbi.nlm.nih.gov/sites/entrez?db=pubmed&cmd=search&term=19211935) |  |  |  |
|  | [hsa-miR-29b](http://www.mirbase.org/cgi-bin/mirna_entry.pl?acc=MI0000105) | [19706389](http://www.ncbi.nlm.nih.gov/sites/entrez?db=pubmed&cmd=search&term=19706389) |  |  |  |
|  | [hsa-miR-29b](http://www.mirbase.org/cgi-bin/mirna_entry.pl?acc=MI0000107) | [19706389](http://www.ncbi.nlm.nih.gov/sites/entrez?db=pubmed&cmd=search&term=19706389) |  |  |  |
|  | [hsa-miR-29b-1*](http://www.mirbase.org/cgi-bin/mirna_entry.pl?acc=MI0000105) | [19211935](http://www.ncbi.nlm.nih.gov/sites/entrez?db=pubmed&cmd=search&term=19211935) |  |  |  |
|  | [hsa-miR-302c](http://www.mirbase.org/cgi-bin/mirna_entry.pl?acc=MI0000773) | [19684618](http://www.ncbi.nlm.nih.gov/sites/entrez?db=pubmed&cmd=search&term=19684618) |  |  |  |
|  | [hsa-miR-302c*](http://www.mirbase.org/cgi-bin/mirna_entry.pl?acc=MI0000773) | [19684618](http://www.ncbi.nlm.nih.gov/sites/entrez?db=pubmed&cmd=search&term=19684618) |  |  |  |
|  | [hsa-miR-30b](http://www.mirbase.org/cgi-bin/mirna_entry.pl?acc=MI0000441) | [20732949](http://www.ncbi.nlm.nih.gov/sites/entrez?db=pubmed&cmd=search&term=20732949) |  |  |  |
|  | [hsa-miR-30b](http://www.mirbase.org/cgi-bin/mirna_entry.pl?acc=MI0000441) | [20347265](http://www.ncbi.nlm.nih.gov/sites/entrez?db=pubmed&cmd=search&term=20347265) |  |  |  |
|  | [hsa-miR-30b*](http://www.mirbase.org/cgi-bin/mirna_entry.pl?acc=MI0000441) | [20732949](http://www.ncbi.nlm.nih.gov/sites/entrez?db=pubmed&cmd=search&term=20732949) |  |  |  |
|  | [hsa-miR-30b*](http://www.mirbase.org/cgi-bin/mirna_entry.pl?acc=MI0000441) | [20347265](http://www.ncbi.nlm.nih.gov/sites/entrez?db=pubmed&cmd=search&term=20347265) |  |  |  |
|  | [hsa-miR-30d](http://www.mirbase.org/cgi-bin/mirna_entry.pl?acc=MI0000255) | [20600201](http://www.ncbi.nlm.nih.gov/sites/entrez?db=pubmed&cmd=search&term=20600201) |  |  |  |
|  | [hsa-miR-30d*](http://www.mirbase.org/cgi-bin/mirna_entry.pl?acc=MI0000255) | [20600201](http://www.ncbi.nlm.nih.gov/sites/entrez?db=pubmed&cmd=search&term=20600201) |  |  |  |
|  | [hsa-miR-30e](http://www.mirbase.org/cgi-bin/mirna_entry.pl?acc=MI0000749) | [20347265](http://www.ncbi.nlm.nih.gov/sites/entrez?db=pubmed&cmd=search&term=20347265) |  |  |  |
|  | [hsa-miR-30e*](http://www.mirbase.org/cgi-bin/mirna_entry.pl?acc=MI0000749) | [20347265](http://www.ncbi.nlm.nih.gov/sites/entrez?db=pubmed&cmd=search&term=20347265) |  |  |  |
|  | [hsa-miR-335](http://www.mirbase.org/cgi-bin/mirna_entry.pl?acc=MI0000816) | [21618216](http://www.ncbi.nlm.nih.gov/sites/entrez?db=pubmed&cmd=search&term=21618216) |  |  |  |
|  | [hsa-miR-335*](http://www.mirbase.org/cgi-bin/mirna_entry.pl?acc=MI0000816) | [21618216](http://www.ncbi.nlm.nih.gov/sites/entrez?db=pubmed&cmd=search&term=21618216) |  |  |  |
|  | [hsa-miR-375](http://www.mirbase.org/cgi-bin/mirna_entry.pl?acc=MI0000783) | [20978187](http://www.ncbi.nlm.nih.gov/sites/entrez?db=pubmed&cmd=search&term=20978187) |  |  |  |
|  | [hsa-miR-378](http://www.mirbase.org/cgi-bin/mirna_entry.pl?acc=MI0000786) | [20078964](http://www.ncbi.nlm.nih.gov/sites/entrez?db=pubmed&cmd=search&term=20078964) |  |  |  |
|  | [hsa-miR-378*](http://www.mirbase.org/cgi-bin/mirna_entry.pl?acc=MI0000786) | [20078964](http://www.ncbi.nlm.nih.gov/sites/entrez?db=pubmed&cmd=search&term=20078964) |  |  |  |
|  | [hsa-miR-424](http://www.mirbase.org/cgi-bin/mirna_entry.pl?acc=MI0001446) | [20348243](http://www.ncbi.nlm.nih.gov/sites/entrez?db=pubmed&cmd=search&term=20348243) |  |  |  |
|  | [hsa-miR-424*](http://www.mirbase.org/cgi-bin/mirna_entry.pl?acc=MI0001446) | [20348243](http://www.ncbi.nlm.nih.gov/sites/entrez?db=pubmed&cmd=search&term=20348243) |  |  |  |
|  | [hsa-miR-453](http://www.mirbase.org/cgi-bin/mirna_entry.pl?acc=MI0001727) | [19028706](http://www.ncbi.nlm.nih.gov/sites/entrez?db=pubmed&cmd=search&term=19028706) |  |  |  |
|  | [hsa-miR-499-3p](http://www.mirbase.org/cgi-bin/mirna_entry.pl?acc=MI0003183) | [22019503](http://www.ncbi.nlm.nih.gov/sites/entrez?db=pubmed&cmd=search&term=22019503) |  |  |  |
|  | [hsa-miR-499-5p](http://www.mirbase.org/cgi-bin/mirna_entry.pl?acc=MI0003183) | [22019503](http://www.ncbi.nlm.nih.gov/sites/entrez?db=pubmed&cmd=search&term=22019503) |  |  |  |
|  | [hsa-miR-570](http://www.mirbase.org/cgi-bin/mirna_entry.pl?acc=MI0003577) | [20348243](http://www.ncbi.nlm.nih.gov/sites/entrez?db=pubmed&cmd=search&term=20348243) |  |  |  |
|  | [hsa-miR-618](http://www.mirbase.org/cgi-bin/mirna_entry.pl?acc=MI0003632) | [20348243](http://www.ncbi.nlm.nih.gov/sites/entrez?db=pubmed&cmd=search&term=20348243) |  |  |  |
|  | [hsa-miR-760](http://www.mirbase.org/cgi-bin/mirna_entry.pl?acc=MI0005567) | [20348243](http://www.ncbi.nlm.nih.gov/sites/entrez?db=pubmed&cmd=search&term=20348243) |  |  |  |
|  | [hsa-miR-92a](http://www.mirbase.org/cgi-bin/mirna_entry.pl?acc=MI0000093) | [20347265](http://www.ncbi.nlm.nih.gov/sites/entrez?db=pubmed&cmd=search&term=20347265) |  |  |  |
|  | [hsa-miR-92a](http://www.mirbase.org/cgi-bin/mirna_entry.pl?acc=MI0000094) | [20347265](http://www.ncbi.nlm.nih.gov/sites/entrez?db=pubmed&cmd=search&term=20347265) |  |  |  |
|  | [hsa-miR-92a](http://www.mirbase.org/cgi-bin/mirna_entry.pl?acc=MI0000093) | [19706389](http://www.ncbi.nlm.nih.gov/sites/entrez?db=pubmed&cmd=search&term=19706389) |  |  |  |
|  | [hsa-miR-92a](http://www.mirbase.org/cgi-bin/mirna_entry.pl?acc=MI0000094) | [19706389](http://www.ncbi.nlm.nih.gov/sites/entrez?db=pubmed&cmd=search&term=19706389) |  |  |  |
|  | [hsa-miR-92a-1*](http://www.mirbase.org/cgi-bin/mirna_entry.pl?acc=MI0000093) | [20347265](http://www.ncbi.nlm.nih.gov/sites/entrez?db=pubmed&cmd=search&term=20347265) |  |  |  |
|  | [hsa-miR-92a-2*](http://www.mirbase.org/cgi-bin/mirna_entry.pl?acc=MI0000094) | [20347265](http://www.ncbi.nlm.nih.gov/sites/entrez?db=pubmed&cmd=search&term=20347265) |  |  |  |
|  | [hsa-miR-98](http://www.mirbase.org/cgi-bin/mirna_entry.pl?acc=MI0000100) | [19528081](http://www.ncbi.nlm.nih.gov/sites/entrez?db=pubmed&cmd=search&term=19528081) |  |  |  |
| **Key AD pathway gene targeting validated miRs from miRWalk** | | | | | |
| **Gene** | **MicroRNA Name** | **Pubmed ID** | **Gene** | **MicroRNA Name** | **Pubmed ID** |
| [APP](http://www.ncbi.nlm.nih.gov/sites/entrez?Db=gene&Cmd=ShowDetailView&TermToSearch=351&ordinalpos=1&itool=EntrezSystem2.PEntrez.Gene.Gene_ResultsPanel.Gene_RVDocSum) | [hsa-miR-101](http://www.mirbase.org/cgi-bin/mirna_entry.pl?acc=MI0000739) | [20395292](http://www.ncbi.nlm.nih.gov/sites/entrez?db=pubmed&cmd=search&term=20395292) | [APOE](http://www.ncbi.nlm.nih.gov/sites/entrez?Db=gene&Cmd=ShowDetailView&TermToSearch=348&ordinalpos=1&itool=EntrezSystem2.PEntrez.Gene.Gene_ResultsPanel.Gene_RVDocSum) | [hsa-miR-125b](http://www.mirbase.org/cgi-bin/mirna_entry.pl?acc=MI0000446) | [21626108](http://www.ncbi.nlm.nih.gov/sites/entrez?db=pubmed&cmd=search&term=21626108) |
|  | [hsa-miR-101](http://www.mirbase.org/cgi-bin/mirna_entry.pl?acc=MI0000103) | [20395292](http://www.ncbi.nlm.nih.gov/sites/entrez?db=pubmed&cmd=search&term=20395292) |  | [hsa-miR-125b](http://www.mirbase.org/cgi-bin/mirna_entry.pl?acc=MI0000470) | [21626108](http://www.ncbi.nlm.nih.gov/sites/entrez?db=pubmed&cmd=search&term=21626108) |
|  | [hsa-miR-106a](http://www.mirbase.org/cgi-bin/mirna_entry.pl?acc=MI0000113) | [18684319](http://www.ncbi.nlm.nih.gov/sites/entrez?db=pubmed&cmd=search&term=18684319) |  | [hsa-miR-144](http://www.mirbase.org/cgi-bin/mirna_entry.pl?acc=MI0000460) | [21626108](http://www.ncbi.nlm.nih.gov/sites/entrez?db=pubmed&cmd=search&term=21626108) |
|  | [hsa-miR-106a*](http://www.mirbase.org/cgi-bin/mirna_entry.pl?acc=MI0000113) | [18684319](http://www.ncbi.nlm.nih.gov/sites/entrez?db=pubmed&cmd=search&term=18684319) |  | [hsa-miR-144*](http://www.mirbase.org/cgi-bin/mirna_entry.pl?acc=MI0000460) | [21626108](http://www.ncbi.nlm.nih.gov/sites/entrez?db=pubmed&cmd=search&term=21626108) |
|  | [hsa-miR-106b](http://www.mirbase.org/cgi-bin/mirna_entry.pl?acc=MI0000734) | [19110058](http://www.ncbi.nlm.nih.gov/sites/entrez?db=pubmed&cmd=search&term=19110058) |  | [hsa-miR-146a](http://www.mirbase.org/cgi-bin/mirna_entry.pl?acc=MI0000477) | [21263019](http://www.ncbi.nlm.nih.gov/sites/entrez?db=pubmed&cmd=search&term=21263019) |
|  | [hsa-miR-106b*](http://www.mirbase.org/cgi-bin/mirna_entry.pl?acc=MI0000734) | [19110058](http://www.ncbi.nlm.nih.gov/sites/entrez?db=pubmed&cmd=search&term=19110058) |  | [hsa-miR-146a*](http://www.mirbase.org/cgi-bin/mirna_entry.pl?acc=MI0000477) | [21263019](http://www.ncbi.nlm.nih.gov/sites/entrez?db=pubmed&cmd=search&term=21263019) |
|  | [hsa-miR-20a](http://www.mirbase.org/cgi-bin/mirna_entry.pl?acc=MI0000076) | [20458444](http://www.ncbi.nlm.nih.gov/sites/entrez?db=pubmed&cmd=search&term=20458444) |  | [hsa-miR-181a](http://www.mirbase.org/cgi-bin/mirna_entry.pl?acc=MI0000269) | [22956783](http://www.ncbi.nlm.nih.gov/sites/entrez?db=pubmed&cmd=search&term=22956783) |
|  | [hsa-miR-20a](http://www.mirbase.org/cgi-bin/mirna_entry.pl?acc=MI0000076) | [19110058](http://www.ncbi.nlm.nih.gov/sites/entrez?db=pubmed&cmd=search&term=19110058) |  | [hsa-miR-181a](http://www.mirbase.org/cgi-bin/mirna_entry.pl?acc=MI0000289) | [22956783](http://www.ncbi.nlm.nih.gov/sites/entrez?db=pubmed&cmd=search&term=22956783) |
|  | [hsa-miR-20a*](http://www.mirbase.org/cgi-bin/mirna_entry.pl?acc=MI0000076) | [20458444](http://www.ncbi.nlm.nih.gov/sites/entrez?db=pubmed&cmd=search&term=20458444) |  | [hsa-miR-192](http://www.mirbase.org/cgi-bin/mirna_entry.pl?acc=MI0000234) | [20393144](http://www.ncbi.nlm.nih.gov/sites/entrez?db=pubmed&cmd=search&term=20393144) |
|  | [hsa-miR-20a*](http://www.mirbase.org/cgi-bin/mirna_entry.pl?acc=MI0000076) | [19110058](http://www.ncbi.nlm.nih.gov/sites/entrez?db=pubmed&cmd=search&term=19110058) |  | [hsa-miR-192*](http://www.mirbase.org/cgi-bin/mirna_entry.pl?acc=MI0000234) | [20393144](http://www.ncbi.nlm.nih.gov/sites/entrez?db=pubmed&cmd=search&term=20393144) |
|  | [hsa-miR-520c-3p](http://www.mirbase.org/cgi-bin/mirna_entry.pl?acc=MI0003158) | [18684319](http://www.ncbi.nlm.nih.gov/sites/entrez?db=pubmed&cmd=search&term=18684319) |  | [hsa-miR-215](http://www.mirbase.org/cgi-bin/mirna_entry.pl?acc=MI0000291) | [20393144](http://www.ncbi.nlm.nih.gov/sites/entrez?db=pubmed&cmd=search&term=20393144) |
| ADAM10 | hsa-miR-122 | 19726678 |  | [hsa-miR-222](http://www.mirbase.org/cgi-bin/mirna_entry.pl?acc=MI0000299) | [23294923](http://www.ncbi.nlm.nih.gov/sites/entrez?db=pubmed&cmd=search&term=23294923) |
|  | hsa-miR-122* | 19726678 |  | [hsa-miR-222*](http://www.mirbase.org/cgi-bin/mirna_entry.pl?acc=MI0000299) | [23294923](http://www.ncbi.nlm.nih.gov/sites/entrez?db=pubmed&cmd=search&term=23294923) |
|  | hsa-miR-181b | 22009755 |  | [hsa-miR-29a](http://www.mirbase.org/cgi-bin/mirna_entry.pl?acc=MI0000087) | [21626108](http://www.ncbi.nlm.nih.gov/sites/entrez?db=pubmed&cmd=search&term=21626108) |
|  | hsa-miR-181b | 22009755 |  | [hsa-miR-29a*](http://www.mirbase.org/cgi-bin/mirna_entry.pl?acc=MI0000087) | [21626108](http://www.ncbi.nlm.nih.gov/sites/entrez?db=pubmed&cmd=search&term=21626108) |
|  | hsa-miR-221 | 22009755 |  | [hsa-miR-886-3p](http://www.mirbase.org/cgi-bin/mirna_entry.pl?acc=MI0005527) | [20413673](http://www.ncbi.nlm.nih.gov/sites/entrez?db=pubmed&cmd=search&term=20413673) |
|  | hsa-miR-221* | 22009755 |  | [hsa-miR-886-5p](http://www.mirbase.org/cgi-bin/mirna_entry.pl?acc=MI0005527) | [20413673](http://www.ncbi.nlm.nih.gov/sites/entrez?db=pubmed&cmd=search&term=20413673) |
|  | hsa-miR-222 | 22009755 | [MME](http://www.ncbi.nlm.nih.gov/sites/entrez?Db=gene&Cmd=ShowDetailView&TermToSearch=4311&ordinalpos=1&itool=EntrezSystem2.PEntrez.Gene.Gene_ResultsPanel.Gene_RVDocSum) | [hsa-miR-155](http://www.mirbase.org/cgi-bin/mirna_entry.pl?acc=MI0000681) | [20947507](http://www.ncbi.nlm.nih.gov/sites/entrez?db=pubmed&cmd=search&term=20947507) |
|  | hsa-miR-222* | 22009755 |  | [hsa-miR-155*](http://www.mirbase.org/cgi-bin/mirna_entry.pl?acc=MI0000681) | [20947507](http://www.ncbi.nlm.nih.gov/sites/entrez?db=pubmed&cmd=search&term=20947507) |
| GAPDH | hsa-miR-1 | 21177382 | [SNCA](http://www.ncbi.nlm.nih.gov/sites/entrez?Db=gene&Cmd=ShowDetailView&TermToSearch=6622&ordinalpos=1&itool=EntrezSystem2.PEntrez.Gene.Gene_ResultsPanel.Gene_RVDocSum) | [hsa-miR-30a](http://www.mirbase.org/cgi-bin/mirna_entry.pl?acc=MI0000088) | [21338582](http://www.ncbi.nlm.nih.gov/sites/entrez?db=pubmed&cmd=search&term=21338582) |
|  | hsa-miR-1 | 21177382 |  | [hsa-miR-30a*](http://www.mirbase.org/cgi-bin/mirna_entry.pl?acc=MI0000088) | [21338582](http://www.ncbi.nlm.nih.gov/sites/entrez?db=pubmed&cmd=search&term=21338582) |
|  | hsa-miR-132 | 20300990 |  | [hsa-miR-30b](http://www.mirbase.org/cgi-bin/mirna_entry.pl?acc=MI0000441) | [21338582](http://www.ncbi.nlm.nih.gov/sites/entrez?db=pubmed&cmd=search&term=21338582) |
|  | hsa-miR-132* | 20300990 |  | [hsa-miR-30b*](http://www.mirbase.org/cgi-bin/mirna_entry.pl?acc=MI0000441) | [21338582](http://www.ncbi.nlm.nih.gov/sites/entrez?db=pubmed&cmd=search&term=21338582) |
|  | hsa-miR-155 | 19603286 |  | [hsa-miR-30c](http://www.mirbase.org/cgi-bin/mirna_entry.pl?acc=MI0000736) | [21338582](http://www.ncbi.nlm.nih.gov/sites/entrez?db=pubmed&cmd=search&term=21338582) |
|  | hsa-miR-155 | 18758992 |  | [hsa-miR-30c](http://www.mirbase.org/cgi-bin/mirna_entry.pl?acc=MI0000254) | [21338582](http://www.ncbi.nlm.nih.gov/sites/entrez?db=pubmed&cmd=search&term=21338582) |
|  | hsa-miR-155* | 19603286 |  | [hsa-miR-30c-1*](http://www.mirbase.org/cgi-bin/mirna_entry.pl?acc=MI0000736) | [21338582](http://www.ncbi.nlm.nih.gov/sites/entrez?db=pubmed&cmd=search&term=21338582) |
|  | hsa-miR-155* | 18758992 |  | [hsa-miR-30c-2*](http://www.mirbase.org/cgi-bin/mirna_entry.pl?acc=MI0000254) | [21338582](http://www.ncbi.nlm.nih.gov/sites/entrez?db=pubmed&cmd=search&term=21338582) |
|  | hsa-miR-29a | 18390668 |  | [hsa-miR-30d](http://www.mirbase.org/cgi-bin/mirna_entry.pl?acc=MI0000255) | [21338582](http://www.ncbi.nlm.nih.gov/sites/entrez?db=pubmed&cmd=search&term=21338582) |
|  | hsa-miR-29a* | 18390668 |  | [hsa-miR-30d*](http://www.mirbase.org/cgi-bin/mirna_entry.pl?acc=MI0000255) | [21338582](http://www.ncbi.nlm.nih.gov/sites/entrez?db=pubmed&cmd=search&term=21338582) |
|  | hsa-miR-29c | 18390668 |  | [hsa-miR-30e](http://www.mirbase.org/cgi-bin/mirna_entry.pl?acc=MI0000749) | [21338582](http://www.ncbi.nlm.nih.gov/sites/entrez?db=pubmed&cmd=search&term=21338582) |
|  | hsa-miR-29c* | 18390668 |  | [hsa-miR-30e*](http://www.mirbase.org/cgi-bin/mirna_entry.pl?acc=MI0000749) | [21338582](http://www.ncbi.nlm.nih.gov/sites/entrez?db=pubmed&cmd=search&term=21338582) |
|  | hsa-miR-30a | 18758992 |  | [hsa-miR-433](http://www.mirbase.org/cgi-bin/mirna_entry.pl?acc=MI0001723) | [22617348](http://www.ncbi.nlm.nih.gov/sites/entrez?db=pubmed&cmd=search&term=22617348) |
|  | hsa-miR-30a* | 18758992 |  | [hsa-miR-7](http://www.mirbase.org/cgi-bin/mirna_entry.pl?acc=MI0000263) | [19628698](http://www.ncbi.nlm.nih.gov/sites/entrez?db=pubmed&cmd=search&term=19628698) |
|  | hsa-miR-30b | 18758992 |  | [hsa-miR-7](http://www.mirbase.org/cgi-bin/mirna_entry.pl?acc=MI0000264) | [19628698](http://www.ncbi.nlm.nih.gov/sites/entrez?db=pubmed&cmd=search&term=19628698) |
|  | hsa-miR-30b* | 18758992 |  | [hsa-miR-7](http://www.mirbase.org/cgi-bin/mirna_entry.pl?acc=MI0000265) | [19628698](http://www.ncbi.nlm.nih.gov/sites/entrez?db=pubmed&cmd=search&term=19628698) |
|  | hsa-miR-30c | 18758992 | [GRIN1](http://www.ncbi.nlm.nih.gov/sites/entrez?Db=gene&Cmd=ShowDetailView&TermToSearch=2902&ordinalpos=1&itool=EntrezSystem2.PEntrez.Gene.Gene_ResultsPanel.Gene_RVDocSum) | [hsa-miR-219-1-3p](http://www.mirbase.org/cgi-bin/mirna_entry.pl?acc=MI0000296) | [19196972](http://www.ncbi.nlm.nih.gov/sites/entrez?db=pubmed&cmd=search&term=19196972) |
|  | hsa-miR-30c | 18758992 |  | [hsa-miR-219-2-3p](http://www.mirbase.org/cgi-bin/mirna_entry.pl?acc=MI0000740) | [19196972](http://www.ncbi.nlm.nih.gov/sites/entrez?db=pubmed&cmd=search&term=19196972) |
|  | hsa-miR-30c-1* | 18758992 |  | [hsa-miR-219-5p](http://www.mirbase.org/cgi-bin/mirna_entry.pl?acc=MI0000296) | [19196972](http://www.ncbi.nlm.nih.gov/sites/entrez?db=pubmed&cmd=search&term=19196972) |
|  | hsa-miR-30c-2* | 18758992 |  | [hsa-miR-219-5p](http://www.mirbase.org/cgi-bin/mirna_entry.pl?acc=MI0000740) | [19196972](http://www.ncbi.nlm.nih.gov/sites/entrez?db=pubmed&cmd=search&term=19196972) |
|  | hsa-miR-30d | 18758992 | [CACNA1C](http://www.ncbi.nlm.nih.gov/sites/entrez?Db=gene&Cmd=ShowDetailView&TermToSearch=775&ordinalpos=1&itool=EntrezSystem2.PEntrez.Gene.Gene_ResultsPanel.Gene_RVDocSum) | [hsa-miR-1](http://www.mirbase.org/cgi-bin/mirna_entry.pl?acc=MI0000437) | [19136465](http://www.ncbi.nlm.nih.gov/sites/entrez?db=pubmed&cmd=search&term=19136465) |
|  | hsa-miR-30d* | 18758992 |  | [hsa-miR-1](http://www.mirbase.org/cgi-bin/mirna_entry.pl?acc=MI0000651) | [19136465](http://www.ncbi.nlm.nih.gov/sites/entrez?db=pubmed&cmd=search&term=19136465) |
|  | hsa-miR-30e | 18758992 |  | [hsa-miR-101](http://www.mirbase.org/cgi-bin/mirna_entry.pl?acc=MI0000103) | [21098446](http://www.ncbi.nlm.nih.gov/sites/entrez?db=pubmed&cmd=search&term=21098446) |
|  | hsa-miR-30e* | 18758992 |  | [hsa-miR-101](http://www.mirbase.org/cgi-bin/mirna_entry.pl?acc=MI0000739) | [21098446](http://www.ncbi.nlm.nih.gov/sites/entrez?db=pubmed&cmd=search&term=21098446) |
| [BACE1](http://www.ncbi.nlm.nih.gov/sites/entrez?Db=gene&Cmd=ShowDetailView&TermToSearch=23621&ordinalpos=1&itool=EntrezSystem2.PEntrez.Gene.Gene_ResultsPanel.Gene_RVDocSum) | [hsa-let-7a](http://www.mirbase.org/cgi-bin/mirna_entry.pl?acc=MI0000061) | [20413881](http://www.ncbi.nlm.nih.gov/sites/entrez?db=pubmed&cmd=search&term=20413881) |  | [hsa-miR-133a](http://www.mirbase.org/cgi-bin/mirna_entry.pl?acc=MI0000450) | [21109942](http://www.ncbi.nlm.nih.gov/sites/entrez?db=pubmed&cmd=search&term=21109942) |
|  | [hsa-let-7a](http://www.mirbase.org/cgi-bin/mirna_entry.pl?acc=MI0000062) | [20413881](http://www.ncbi.nlm.nih.gov/sites/entrez?db=pubmed&cmd=search&term=20413881) |  | [hsa-miR-133a](http://www.mirbase.org/cgi-bin/mirna_entry.pl?acc=MI0000451) | [21109942](http://www.ncbi.nlm.nih.gov/sites/entrez?db=pubmed&cmd=search&term=21109942) |
|  | [hsa-let-7a](http://www.mirbase.org/cgi-bin/mirna_entry.pl?acc=MI0000060) | [20413881](http://www.ncbi.nlm.nih.gov/sites/entrez?db=pubmed&cmd=search&term=20413881) |  | [hsa-miR-133a](http://www.mirbase.org/cgi-bin/mirna_entry.pl?acc=MI0000450) | [19136465](http://www.ncbi.nlm.nih.gov/sites/entrez?db=pubmed&cmd=search&term=19136465) |
|  | [hsa-miR-107](http://www.mirbase.org/cgi-bin/mirna_entry.pl?acc=MI0000114) | [20413881](http://www.ncbi.nlm.nih.gov/sites/entrez?db=pubmed&cmd=search&term=20413881) |  | [hsa-miR-133a](http://www.mirbase.org/cgi-bin/mirna_entry.pl?acc=MI0000451) | [19136465](http://www.ncbi.nlm.nih.gov/sites/entrez?db=pubmed&cmd=search&term=19136465) |
|  | [hsa-miR-107](http://www.mirbase.org/cgi-bin/mirna_entry.pl?acc=MI0000114) | [18234899](http://www.ncbi.nlm.nih.gov/sites/entrez?db=pubmed&cmd=search&term=18234899) |  | [hsa-miR-133b](http://www.mirbase.org/cgi-bin/mirna_entry.pl?acc=MI0000822) | [19136465](http://www.ncbi.nlm.nih.gov/sites/entrez?db=pubmed&cmd=search&term=19136465) |
|  | [hsa-miR-124](http://www.mirbase.org/cgi-bin/mirna_entry.pl?acc=MI0000443) | [18434550](http://www.ncbi.nlm.nih.gov/sites/entrez?db=pubmed&cmd=search&term=18434550) |  | [hsa-miR-137](http://www.mirbase.org/cgi-bin/mirna_entry.pl?acc=MI0000454) | [22883350](http://www.ncbi.nlm.nih.gov/sites/entrez?db=pubmed&cmd=search&term=22883350) |
|  | [hsa-miR-124](http://www.mirbase.org/cgi-bin/mirna_entry.pl?acc=MI0000443) | [20413881](http://www.ncbi.nlm.nih.gov/sites/entrez?db=pubmed&cmd=search&term=20413881) |  | [hsa-miR-155](http://www.mirbase.org/cgi-bin/mirna_entry.pl?acc=MI0000681) | [19136465](http://www.ncbi.nlm.nih.gov/sites/entrez?db=pubmed&cmd=search&term=19136465) |
|  | [hsa-miR-124](http://www.mirbase.org/cgi-bin/mirna_entry.pl?acc=MI0000444) | [18434550](http://www.ncbi.nlm.nih.gov/sites/entrez?db=pubmed&cmd=search&term=18434550) |  | [hsa-miR-155*](http://www.mirbase.org/cgi-bin/mirna_entry.pl?acc=MI0000681) | [19136465](http://www.ncbi.nlm.nih.gov/sites/entrez?db=pubmed&cmd=search&term=19136465) |
|  | [hsa-miR-124](http://www.mirbase.org/cgi-bin/mirna_entry.pl?acc=MI0000444) | [20413881](http://www.ncbi.nlm.nih.gov/sites/entrez?db=pubmed&cmd=search&term=20413881) |  | [hsa-miR-17](http://www.mirbase.org/cgi-bin/mirna_entry.pl?acc=MI0000071) | [19136465](http://www.ncbi.nlm.nih.gov/sites/entrez?db=pubmed&cmd=search&term=19136465) |
|  | [hsa-miR-124](http://www.mirbase.org/cgi-bin/mirna_entry.pl?acc=MI0000445) | [18434550](http://www.ncbi.nlm.nih.gov/sites/entrez?db=pubmed&cmd=search&term=18434550) |  | [hsa-miR-17*](http://www.mirbase.org/cgi-bin/mirna_entry.pl?acc=MI0000071) | [19136465](http://www.ncbi.nlm.nih.gov/sites/entrez?db=pubmed&cmd=search&term=19136465) |
|  | [hsa-miR-124](http://www.mirbase.org/cgi-bin/mirna_entry.pl?acc=MI0000445) | [20413881](http://www.ncbi.nlm.nih.gov/sites/entrez?db=pubmed&cmd=search&term=20413881) |  | [hsa-miR-21](http://www.mirbase.org/cgi-bin/mirna_entry.pl?acc=MI0000077) | [19136465](http://www.ncbi.nlm.nih.gov/sites/entrez?db=pubmed&cmd=search&term=19136465) |
|  | [hsa-miR-15a](http://www.mirbase.org/cgi-bin/mirna_entry.pl?acc=MI0000069) | [18434550](http://www.ncbi.nlm.nih.gov/sites/entrez?db=pubmed&cmd=search&term=18434550) |  | [hsa-miR-21*](http://www.mirbase.org/cgi-bin/mirna_entry.pl?acc=MI0000077) | [19136465](http://www.ncbi.nlm.nih.gov/sites/entrez?db=pubmed&cmd=search&term=19136465) |
|  | [hsa-miR-15a*](http://www.mirbase.org/cgi-bin/mirna_entry.pl?acc=MI0000069) | [18434550](http://www.ncbi.nlm.nih.gov/sites/entrez?db=pubmed&cmd=search&term=18434550) |  | [hsa-miR-223](http://www.mirbase.org/cgi-bin/mirna_entry.pl?acc=MI0000300) | [21098446](http://www.ncbi.nlm.nih.gov/sites/entrez?db=pubmed&cmd=search&term=21098446) |
|  | [hsa-miR-195](http://www.mirbase.org/cgi-bin/mirna_entry.pl?acc=MI0000489) | [22721728](http://www.ncbi.nlm.nih.gov/sites/entrez?db=pubmed&cmd=search&term=22721728) |  | [hsa-miR-223*](http://www.mirbase.org/cgi-bin/mirna_entry.pl?acc=MI0000300) | [21098446](http://www.ncbi.nlm.nih.gov/sites/entrez?db=pubmed&cmd=search&term=21098446) |
|  | [hsa-miR-195*](http://www.mirbase.org/cgi-bin/mirna_entry.pl?acc=MI0000489) | [22721728](http://www.ncbi.nlm.nih.gov/sites/entrez?db=pubmed&cmd=search&term=22721728) |  | [hsa-miR-328](http://www.mirbase.org/cgi-bin/mirna_entry.pl?acc=MI0000804) | [21098446](http://www.ncbi.nlm.nih.gov/sites/entrez?db=pubmed&cmd=search&term=21098446) |
|  | [hsa-miR-19b](http://www.mirbase.org/cgi-bin/mirna_entry.pl?acc=MI0000074) | [18434550](http://www.ncbi.nlm.nih.gov/sites/entrez?db=pubmed&cmd=search&term=18434550) |  | [hsa-miR-499-3p](http://www.mirbase.org/cgi-bin/mirna_entry.pl?acc=MI0003183) | [21098446](http://www.ncbi.nlm.nih.gov/sites/entrez?db=pubmed&cmd=search&term=21098446) |
|  | [hsa-miR-19b](http://www.mirbase.org/cgi-bin/mirna_entry.pl?acc=MI0000075) | [18434550](http://www.ncbi.nlm.nih.gov/sites/entrez?db=pubmed&cmd=search&term=18434550) |  | [hsa-miR-499-5p](http://www.mirbase.org/cgi-bin/mirna_entry.pl?acc=MI0003183) | [21098446](http://www.ncbi.nlm.nih.gov/sites/entrez?db=pubmed&cmd=search&term=21098446) |
|  | [hsa-miR-298](http://www.mirbase.org/cgi-bin/mirna_entry.pl?acc=MI0005523) | [23030236](http://www.ncbi.nlm.nih.gov/sites/entrez?db=pubmed&cmd=search&term=23030236) |  | [hsa-miR-664](http://www.mirbase.org/cgi-bin/mirna_entry.pl?acc=MI0006442) | [21098446](http://www.ncbi.nlm.nih.gov/sites/entrez?db=pubmed&cmd=search&term=21098446) |
|  | [hsa-miR-298](http://www.mirbase.org/cgi-bin/mirna_entry.pl?acc=MI0005523) | [20375468](http://www.ncbi.nlm.nih.gov/sites/entrez?db=pubmed&cmd=search&term=20375468) |  | [hsa-miR-664*](http://www.mirbase.org/cgi-bin/mirna_entry.pl?acc=MI0006442) | [21098446](http://www.ncbi.nlm.nih.gov/sites/entrez?db=pubmed&cmd=search&term=21098446) |
|  | [hsa-miR-298](http://www.mirbase.org/cgi-bin/mirna_entry.pl?acc=MI0005523) | [18986979](http://www.ncbi.nlm.nih.gov/sites/entrez?db=pubmed&cmd=search&term=18986979) | CASP7 | hsa-miR-125b | 18056640 |
|  | [hsa-miR-29a](http://www.mirbase.org/cgi-bin/mirna_entry.pl?acc=MI0000087) | [19462468](http://www.ncbi.nlm.nih.gov/sites/entrez?db=pubmed&cmd=search&term=19462468) |  | hsa-miR-125b | 18056640 |
|  | [hsa-miR-29a](http://www.mirbase.org/cgi-bin/mirna_entry.pl?acc=MI0000087) | [18434550](http://www.ncbi.nlm.nih.gov/sites/entrez?db=pubmed&cmd=search&term=18434550) | GSK3B | hsa-miR-26a | 20525681 |
|  | [hsa-miR-29a*](http://www.mirbase.org/cgi-bin/mirna_entry.pl?acc=MI0000087) | [19462468](http://www.ncbi.nlm.nih.gov/sites/entrez?db=pubmed&cmd=search&term=19462468) |  | hsa-miR-26a | 20525681 |
|  | [hsa-miR-29a*](http://www.mirbase.org/cgi-bin/mirna_entry.pl?acc=MI0000087) | [18434550](http://www.ncbi.nlm.nih.gov/sites/entrez?db=pubmed&cmd=search&term=18434550) |  | hsa-miR-26a-1* | 20525681 |
|  | [hsa-miR-29b](http://www.mirbase.org/cgi-bin/mirna_entry.pl?acc=MI0000105) | [18434550](http://www.ncbi.nlm.nih.gov/sites/entrez?db=pubmed&cmd=search&term=18434550) |  | hsa-miR-26a-2* | 20525681 |
|  | [hsa-miR-29b](http://www.mirbase.org/cgi-bin/mirna_entry.pl?acc=MI0000107) | [18434550](http://www.ncbi.nlm.nih.gov/sites/entrez?db=pubmed&cmd=search&term=18434550) |  | hsa-miR-26b | 20525681 |
|  | [hsa-miR-29b](http://www.mirbase.org/cgi-bin/mirna_entry.pl?acc=MI0000105) | [19462468](http://www.ncbi.nlm.nih.gov/sites/entrez?db=pubmed&cmd=search&term=19462468) |  | hsa-miR-26b* | 20525681 |
|  | [hsa-miR-29b](http://www.mirbase.org/cgi-bin/mirna_entry.pl?acc=MI0000107) | [19462468](http://www.ncbi.nlm.nih.gov/sites/entrez?db=pubmed&cmd=search&term=19462468) | LPL | hsa-let-7b | 18668040 |
|  | [hsa-miR-29b-1*](http://www.mirbase.org/cgi-bin/mirna_entry.pl?acc=MI0000105) | [19462468](http://www.ncbi.nlm.nih.gov/sites/entrez?db=pubmed&cmd=search&term=19462468) |  | hsa-let-7b* | 18668040 |
|  | [hsa-miR-29b-1*](http://www.mirbase.org/cgi-bin/mirna_entry.pl?acc=MI0000105) | [18434550](http://www.ncbi.nlm.nih.gov/sites/entrez?db=pubmed&cmd=search&term=18434550) |  | hsa-miR-1 | 18668040 |
|  | [hsa-miR-29b-2*](http://www.mirbase.org/cgi-bin/mirna_entry.pl?acc=MI0000107) | [19462468](http://www.ncbi.nlm.nih.gov/sites/entrez?db=pubmed&cmd=search&term=19462468) |  | hsa-miR-155 | 18668040 |
|  | [hsa-miR-29c](http://www.mirbase.org/cgi-bin/mirna_entry.pl?acc=MI0000735) | [19462468](http://www.ncbi.nlm.nih.gov/sites/entrez?db=pubmed&cmd=search&term=19462468) |  | hsa-miR-155* | 18668040 |
|  | [hsa-miR-29c](http://www.mirbase.org/cgi-bin/mirna_entry.pl?acc=MI0000735) | [21565331](http://www.ncbi.nlm.nih.gov/sites/entrez?db=pubmed&cmd=search&term=21565331) |  | hsa-miR-16 | 18668040 |
|  | [hsa-miR-29c*](http://www.mirbase.org/cgi-bin/mirna_entry.pl?acc=MI0000735) | [19462468](http://www.ncbi.nlm.nih.gov/sites/entrez?db=pubmed&cmd=search&term=19462468) |  | hsa-miR-30a | 18668040 |
|  | [hsa-miR-29c*](http://www.mirbase.org/cgi-bin/mirna_entry.pl?acc=MI0000735) | [21565331](http://www.ncbi.nlm.nih.gov/sites/entrez?db=pubmed&cmd=search&term=21565331) |  | hsa-miR-30a* | 18668040 |
|  | [hsa-miR-328](http://www.mirbase.org/cgi-bin/mirna_entry.pl?acc=MI0000804) | [20375468](http://www.ncbi.nlm.nih.gov/sites/entrez?db=pubmed&cmd=search&term=20375468) |  | hsa-miR-30b | 18668040 |
|  | [hsa-miR-328](http://www.mirbase.org/cgi-bin/mirna_entry.pl?acc=MI0000804) | [18986979](http://www.ncbi.nlm.nih.gov/sites/entrez?db=pubmed&cmd=search&term=18986979) |  | hsa-miR-30b* | 18668040 |
|  | [hsa-miR-485-5p](http://www.mirbase.org/cgi-bin/mirna_entry.pl?acc=MI0002469) | [20507594](http://www.ncbi.nlm.nih.gov/sites/entrez?db=pubmed&cmd=search&term=20507594) |  | hsa-miR-30c | 18668040 |
|  | [hsa-miR-9](http://www.mirbase.org/cgi-bin/mirna_entry.pl?acc=MI0000466) | [18434550](http://www.ncbi.nlm.nih.gov/sites/entrez?db=pubmed&cmd=search&term=18434550) |  | hsa-miR-30c | 18668040 |
|  | [hsa-miR-9](http://www.mirbase.org/cgi-bin/mirna_entry.pl?acc=MI0000467) | [18434550](http://www.ncbi.nlm.nih.gov/sites/entrez?db=pubmed&cmd=search&term=18434550) |  | hsa-miR-30c-1* | 18668040 |
|  | [hsa-miR-9](http://www.mirbase.org/cgi-bin/mirna_entry.pl?acc=MI0000468) | [18434550](http://www.ncbi.nlm.nih.gov/sites/entrez?db=pubmed&cmd=search&term=18434550) |  | hsa-miR-30c-2* | 18668040 |
| [LRP1](http://www.ncbi.nlm.nih.gov/sites/entrez?Db=gene&Cmd=ShowDetailView&TermToSearch=4035&ordinalpos=1&itool=EntrezSystem2.PEntrez.Gene.Gene_ResultsPanel.Gene_RVDocSum) | [hsa-let-7b](http://www.mirbase.org/cgi-bin/mirna_entry.pl?acc=MI0000063) | [18668040](http://www.ncbi.nlm.nih.gov/sites/entrez?db=pubmed&cmd=search&term=18668040) |  | hsa-miR-30d | 18668040 |
|  | [hsa-let-7b*](http://www.mirbase.org/cgi-bin/mirna_entry.pl?acc=MI0000063) | [18668040](http://www.ncbi.nlm.nih.gov/sites/entrez?db=pubmed&cmd=search&term=18668040) |  | hsa-miR-30d* | 18668040 |
|  | [hsa-miR-1](http://www.mirbase.org/cgi-bin/mirna_entry.pl?acc=MI0000437) | [18668040](http://www.ncbi.nlm.nih.gov/sites/entrez?db=pubmed&cmd=search&term=18668040) |  | hsa-miR-30e | 18668040 |
|  | [hsa-miR-155](http://www.mirbase.org/cgi-bin/mirna_entry.pl?acc=MI0000681) | [18668040](http://www.ncbi.nlm.nih.gov/sites/entrez?db=pubmed&cmd=search&term=18668040) |  | hsa-miR-30e* | 18668040 |
|  | [hsa-miR-155*](http://www.mirbase.org/cgi-bin/mirna_entry.pl?acc=MI0000681) | [18668040](http://www.ncbi.nlm.nih.gov/sites/entrez?db=pubmed&cmd=search&term=18668040) |  |  |  |
|  | [hsa-miR-16](http://www.mirbase.org/cgi-bin/mirna_entry.pl?acc=MI0000115) | [18668040](http://www.ncbi.nlm.nih.gov/sites/entrez?db=pubmed&cmd=search&term=18668040) |  |  |  |
|  | [hsa-miR-205](http://www.mirbase.org/cgi-bin/mirna_entry.pl?acc=MI0000285) | [19665999](http://www.ncbi.nlm.nih.gov/sites/entrez?db=pubmed&cmd=search&term=19665999) | APBB1 |  |  |
|  | [hsa-miR-30a](http://www.mirbase.org/cgi-bin/mirna_entry.pl?acc=MI0000088) | [18668040](http://www.ncbi.nlm.nih.gov/sites/entrez?db=pubmed&cmd=search&term=18668040) | PSENEN |  |  |
|  | [hsa-miR-30a*](http://www.mirbase.org/cgi-bin/mirna_entry.pl?acc=MI0000088) | [18668040](http://www.ncbi.nlm.nih.gov/sites/entrez?db=pubmed&cmd=search&term=18668040) | NCSTN | NO RESULT |  |
|  | [hsa-miR-30b](http://www.mirbase.org/cgi-bin/mirna_entry.pl?acc=MI0000441) | [18668040](http://www.ncbi.nlm.nih.gov/sites/entrez?db=pubmed&cmd=search&term=18668040) | IDE |  |  |
|  | [hsa-miR-30b*](http://www.mirbase.org/cgi-bin/mirna_entry.pl?acc=MI0000441) | [18668040](http://www.ncbi.nlm.nih.gov/sites/entrez?db=pubmed&cmd=search&term=18668040) | APH1A |  |  |
|  | [hsa-miR-30c](http://www.mirbase.org/cgi-bin/mirna_entry.pl?acc=MI0000736) | [18668040](http://www.ncbi.nlm.nih.gov/sites/entrez?db=pubmed&cmd=search&term=18668040) | PESN1 |  |  |
|  | [hsa-miR-30c-1*](http://www.mirbase.org/cgi-bin/mirna_entry.pl?acc=MI0000736) | [18668040](http://www.ncbi.nlm.nih.gov/sites/entrez?db=pubmed&cmd=search&term=18668040) |  |  |  |
|  | [hsa-miR-30c-2*](http://www.mirbase.org/cgi-bin/mirna_entry.pl?acc=MI0000254) | [18668040](http://www.ncbi.nlm.nih.gov/sites/entrez?db=pubmed&cmd=search&term=18668040) |  |  |  |
|  | [hsa-miR-30d](http://www.mirbase.org/cgi-bin/mirna_entry.pl?acc=MI0000255) | [18668040](http://www.ncbi.nlm.nih.gov/sites/entrez?db=pubmed&cmd=search&term=18668040) |  |  |  |
|  | [hsa-miR-30d*](http://www.mirbase.org/cgi-bin/mirna_entry.pl?acc=MI0000255) | [18668040](http://www.ncbi.nlm.nih.gov/sites/entrez?db=pubmed&cmd=search&term=18668040) |  |  |  |
|  | [hsa-miR-30e](http://www.mirbase.org/cgi-bin/mirna_entry.pl?acc=MI0000749) | [18668040](http://www.ncbi.nlm.nih.gov/sites/entrez?db=pubmed&cmd=search&term=18668040) |  |  |  |
|  | [hsa-miR-30e*](http://www.mirbase.org/cgi-bin/mirna_entry.pl?acc=MI0000749) | [18668040](http://www.ncbi.nlm.nih.gov/sites/entrez?db=pubmed&cmd=search&term=18668040) |  |  |  |
|  | [hsa-miR-338-5p](http://www.mirbase.org/cgi-bin/mirna_entry.pl?acc=MI0000814) | [19665999](http://www.ncbi.nlm.nih.gov/sites/entrez?db=pubmed&cmd=search&term=19665999) |  |  |  |
|  | [hsa-miR-545](http://www.mirbase.org/cgi-bin/mirna_entry.pl?acc=MI0003516) | [19665999](http://www.ncbi.nlm.nih.gov/sites/entrez?db=pubmed&cmd=search&term=19665999) |  |  |  |
|  | [hsa-miR-545*](http://www.mirbase.org/cgi-bin/mirna_entry.pl?acc=MI0003516) | [19665999](http://www.ncbi.nlm.nih.gov/sites/entrez?db=pubmed&cmd=search&term=19665999) |  |  |  |

**Supplementary Table S12: Predicted targeting miRs of BARHL1-ESR and AD pathway related key genes having no validated target in miRwalk**.

| **BARHL1-ESR1 Pathway Gene targeting predicted miRs from miRWalk** | | | | | |
| --- | --- | --- | --- | --- | --- |
| **Gene Name** | **MicroRNA name** | **score** | **Gene Name** | **MicroRNA name** | **Score** |
| BARHL1 | hsa-miR-507 | 7 | TH | hsa-miR-609 | 1 |
|  | hsa-miR-328 | 6 |  | hsa-miR-151-3p | 1 |
|  | hsa-miR-663 | 6 |  | hsa-miR-885-3p | 4 |
|  | hsa-miR-324-3p | 6 |  | hsa-miR-197 | 4 |
|  | hsa-miR-765 | 6 |  | hsa-miR-34c-5p | 4 |
|  | hsa-miR-647 | 6 |  | hsa-miR-140-3p | 4 |
|  | hsa-miR-602 | 6 |  | hsa-miR-148b | 4 |
|  | hsa-miR-608 | 6 |  | hsa-miR-148a | 4 |
|  | hsa-miR-557 | 6 |  | hsa-miR-504 | 4 |
|  | hsa-miR-661 | 6 |  | hsa-miR-449b | 4 |
|  | hsa-miR-615-3p | 5 |  | hsa-miR-922 | 4 |
|  | hsa-miR-541 | 5 |  | hsa-miR-658 | 4 |
|  | hsa-miR-566 | 5 |  | hsa-miR-152 | 4 |
|  | hsa-miR-515-5p | 5 |  | hsa-miR-449a | 4 |
|  | hsa-miR-619 | 5 |  | hsa-miR-509-3p | 4 |
|  | hsa-miR-920 | 5 |  | hsa-miR-34a | 4 |
|  | hsa-miR-579 | 5 |  | hsa-miR-758 | 4 |
|  | hsa-miR-654-5p | 5 |  | hsa-miR-486-3p | 4 |
|  | hsa-miR-939 | 5 |  | hsa-miR-361-3p | 4 |
|  | hsa-miR-423-5p | 5 |  | hsa-miR-34c-5p | 3 |
|  | hsa-miR-941 | 5 |  | hsa-miR-148a | 3 |
|  | hsa-miR-589 | 5 |  | hsa-miR-885-3p | 3 |
|  | hsa-miR-637 | 5 |  | hsa-miR-497 | 3 |
|  | hsa-miR-139-3p | 5 |  | hsa-miR-575 | 3 |
|  | hsa-miR-483-5p | 5 |  | hsa-miR-130a | 3 |
|  | hsa-miR-942 | 5 |  | hsa-miR-1300 | 3 |
|  | hsa-miR-596 | 5 |  | hsa-miR-486-3p | 3 |
|  | hsa-miR-491-5p | 5 |  | hsa-miR-504 | 3 |
|  | hsa-miR-24 | 5 |  | hsa-miR-603 | 3 |
|  | hsa-miR-886-5p | 5 |  | hsa-miR-214 | 3 |
|  | hsa-miR-296-5p | 5 |  | hsa-miR-214 | 3 |
|  | hsa-miR-185 | 5 |  | hsa-miR-658 | 3 |
|  | hsa-miR-886-3p | 5 |  | hsa-miR-301b | 3 |
|  | hsa-miR-658 | 4 |  | hsa-miR-301a | 3 |
|  | hsa-miR-941 | 4 |  | hsa-miR-34a | 3 |
|  | hsa-miR-1293 | 4 |  | hsa-miR-152 | 3 |
|  | hsa-miR-941 | 4 |  | hsa-miR-424 | 3 |
|  | hsa-miR-941 | 4 |  | hsa-miR-758 | 3 |
|  | hsa-miR-941 | 4 |  | hsa-miR-509-3p | 3 |
|  | hsa-miR-941 | 4 |  | hsa-miR-296-5p | 3 |
|  | hsa-miR-1268 | 4 |  | hsa-miR-449a | 3 |
|  | hsa-miR-485-5p | 4 |  | hsa-miR-509-3p | 3 |
|  | hsa-miR-604 | 4 |  | hsa-miR-148b | 3 |
|  | hsa-miR-941 | 4 |  | hsa-miR-941 | 3 |
|  | hsa-miR-1308 | 4 |  | hsa-miR-130b | 3 |
|  | hsa-miR-941 | 4 |  | hsa-miR-922 | 3 |
|  | hsa-miR-941 | 4 |  | hsa-miR-509-3p | 3 |
|  | hsa-miR-455-3p | 4 |  | hsa-miR-197 | 3 |
|  | hsa-miR-1321 | 4 |  | hsa-miR-1321 | 3 |
|  | hsa-miR-612 | 4 |  | hsa-miR-361-3p | 3 |
|  | hsa-miR-941 | 4 |  | hsa-miR-1226 | 3 |
|  | hsa-miR-941 | 4 |  | hsa-miR-509-3p | 3 |
|  | hsa-miR-539 | 4 |  | hsa-miR-449b | 3 |
|  | hsa-miR-650 | 4 |  | hsa-miR-509-3p | 3 |
|  | hsa-miR-615-5p | 4 |  | hsa-miR-1324 | 3 |
|  | hsa-miR-941 | 4 |  | hsa-miR-492 | 3 |
|  | hsa-miR-515-5p | 4 |  | hsa-miR-140-3p | 3 |
|  | hsa-miR-1291 | 4 |  | hsa-miR-1286 | 3 |
|  | hsa-miR-149 | 4 |  | hsa-miR-512-5p | 2 |
|  | hsa-miR-941 | 4 |  | hsa-miR-941 | 2 |
|  | hsa-miR-31 | 3 |  | hsa-miR-330-5p | 2 |
|  | hsa-miR-133b | 3 |  | hsa-miR-1226 | 2 |
|  | hsa-miR-135a | 3 |  | hsa-miR-412 | 2 |
|  | hsa-miR-1254 | 3 |  | hsa-miR-941 | 2 |
|  | hsa-miR-483-3p | 3 |  | hsa-miR-1324 | 2 |
|  | hsa-miR-1256 | 3 |  | hsa-miR-675 | 2 |
|  | hsa-miR-585 | 3 |  | hsa-miR-885-3p | 2 |
|  | hsa-miR-185* | 3 |  | hsa-miR-107 | 2 |
|  | hsa-miR-135b | 3 |  | hsa-miR-941 | 2 |
|  | hsa-miR-492 | 3 |  | hsa-miR-920 | 2 |
|  | hsa-miR-1271 | 3 |  | hsa-miR-575 | 2 |
|  | hsa-miR-744 | 3 |  | hsa-miR-1266 | 2 |
|  | hsa-miR-486-3p | 3 |  | hsa-miR-411 | 2 |
|  | hsa-miR-649 | 3 |  | hsa-miR-941 | 2 |
|  | hsa-miR-1203 | 3 |  | hsa-miR-301a | 2 |
|  | hsa-miR-133a | 3 |  | hsa-miR-941 | 2 |
|  | hsa-miR-567 | 3 |  | hsa-miR-941 | 2 |
|  | hsa-miR-519e* | 2 |  | hsa-miR-486-3p | 2 |
|  | hsa-miR-1276 | 2 |  | hsa-miR-214 | 2 |
|  | hsa-miR-146b-5p | 2 |  | hsa-miR-504 | 2 |
|  | hsa-miR-26b* | 2 |  | hsa-miR-941 | 2 |
|  | hsa-miR-450b-5p | 2 |  | hsa-miR-1254 | 2 |
|  | hsa-miR-1249 | 2 |  | hsa-miR-34a | 2 |
|  | hsa-miR-583 | 2 |  | hsa-miR-1253 | 2 |
|  | hsa-miR-874 | 2 |  | hsa-miR-941 | 2 |
|  | hsa-miR-149* | 2 |  | hsa-miR-326 | 2 |
|  | hsa-miR-1908 | 2 |  | hsa-miR-152 | 2 |
|  | hsa-miR-211 | 2 |  | hsa-miR-296-5p | 2 |
|  | hsa-miR-675 | 2 |  | hsa-miR-941 | 2 |
|  | hsa-miR-1307 | 2 |  | hsa-miR-658 | 2 |
|  | hsa-miR-92a-2* | 2 |  | hsa-miR-941 | 2 |
|  | hsa-miR-890 | 2 |  | hsa-miR-107 | 2 |
|  | hsa-miR-96 | 2 |  | hsa-miR-103 | 2 |
|  | hsa-miR-342-5p | 2 |  | hsa-miR-216a | 2 |
|  | hsa-miR-572 | 2 |  | hsa-miR-941 | 2 |
|  | hsa-miR-1233 | 2 |  | hsa-miR-941 | 2 |
|  | hsa-miR-1913 | 2 |  | hsa-miR-1266 | 2 |
|  | hsa-miR-937 | 2 |  | hsa-miR-492 | 2 |
|  | hsa-miR-505* | 2 |  | hsa-miR-148b | 2 |
|  | hsa-miR-204 | 2 |  | hsa-miR-941 | 2 |
|  | hsa-miR-629* | 2 |  | hsa-miR-1286 | 2 |
|  | hsa-miR-484 | 2 |  | hsa-miR-301b | 2 |
|  | hsa-miR-30c-2* | 2 |  | hsa-miR-758 | 2 |
|  | hsa-miR-220c | 2 |  | hsa-miR-941 | 2 |
|  | hsa-miR-182 | 2 |  | hsa-miR-646 | 2 |
|  | hsa-miR-1207-5p | 2 |  | hsa-miR-580 | 2 |
|  | hsa-miR-1262 | 2 |  | hsa-miR-920 | 2 |
|  | hsa-miR-662 | 2 |  | hsa-miR-509-3p | 2 |
|  | hsa-miR-644 | 2 |  | hsa-miR-941 | 2 |
|  | hsa-miR-106b* | 2 |  | hsa-miR-941 | 2 |
|  | hsa-miR-1237 | 2 |  | hsa-miR-449a | 2 |
|  | hsa-miR-1914* | 2 |  | hsa-miR-1308 | 2 |
|  | hsa-miR-525-3p | 2 |  | hsa-miR-769-5p | 2 |
|  | hsa-miR-184 | 2 |  | hsa-miR-509-3p | 2 |
|  | hsa-miR-631 | 2 |  | hsa-miR-130b | 2 |
|  | hsa-miR-564 | 2 |  | hsa-miR-580 | 2 |
|  | hsa-miR-1269 | 2 |  | hsa-miR-197 | 2 |
|  | hsa-miR-24 | 2 |  | hsa-miR-637 | 2 |
|  | hsa-miR-30c-1* | 2 |  | hsa-miR-941 | 2 |
|  | hsa-miR-125a-3p | 2 |  | hsa-miR-661 | 2 |
|  | hsa-miR-524-3p | 2 |  | hsa-miR-922 | 2 |
|  | hsa-miR-625 | 2 |  | hsa-miR-361-3p | 2 |
|  | hsa-miR-297 | 2 |  | hsa-miR-941 | 2 |
|  | hsa-miR-34a* | 2 |  | hsa-miR-941 | 2 |
|  | hsa-miR-760 | 2 |  | hsa-miR-603 | 2 |
|  | hsa-miR-1274a | 2 |  | hsa-miR-130a | 2 |
|  | hsa-miR-558 | 2 |  | hsa-miR-1321 | 2 |
|  | hsa-miR-453 | 2 |  | hsa-miR-103 | 2 |
|  | hsa-miR-1285 | 2 |  | hsa-miR-941 | 2 |
|  | hsa-miR-146a | 2 |  | hsa-miR-220b | 2 |
|  | hsa-miR-555 | 2 |  | hsa-miR-1253 | 2 |
|  | hsa-let-7g* | 2 |  | hsa-miR-646 | 2 |
|  | hsa-miR-938 | 2 |  | hsa-miR-941 | 2 |
|  | hsa-miR-122 | 2 |  | hsa-miR-140-3p | 2 |
|  | hsa-miR-1471 | 2 |  | hsa-miR-509-3p | 2 |
|  | hsa-miR-1275 | 2 |  | hsa-miR-941 | 2 |
|  | hsa-miR-490-3p | 2 |  | hsa-miR-449b | 2 |
|  | hsa-miR-15a* | 2 |  | hsa-miR-941 | 2 |
|  | hsa-miR-542-5p | 2 |  | hsa-miR-220b | 2 |
|  | hsa-miR-370 | 2 |  | hsa-miR-103 | 2 |
|  | hsa-miR-363* | 2 |  | hsa-miR-34c-5p | 2 |
|  | hsa-miR-1248 | 2 |  | hsa-miR-941 | 2 |
|  | hsa-miR-183 | 2 |  | hsa-miR-148a | 2 |
|  | hsa-miR-30b* | 2 |  | hsa-miR-769-5p | 2 |
|  | hsa-miR-1225-3p | 2 |  | hsa-miR-661 | 2 |
|  | hsa-miR-135a | 2 |  | hsa-miR-941 | 2 |
|  | hsa-miR-1539 | 2 |  | hsa-miR-943 | 1 |
|  | hsa-miR-376a | 1 |  | hsa-miR-597 | 1 |
|  | hsa-miR-515-3p | 1 |  | hsa-miR-766 | 1 |
|  | hsa-miR-331-3p | 1 |  | hsa-miR-943 | 1 |
|  | hsa-miR-1304 | 1 |  | hsa-miR-512-5p | 1 |
|  | hsa-miR-1236 | 1 |  | hsa-miR-28-5p | 1 |
|  | hsa-miR-205 | 1 |  | hsa-miR-411 | 1 |
|  | hsa-miR-326 | 1 |  | hsa-miR-145 | 1 |
|  | hsa-miR-422a | 1 |  | hsa-miR-744* | 1 |
|  | hsa-miR-194* | 1 |  | hsa-miR-486-5p | 1 |
|  | hsa-let-7f-2* | 1 |  | hsa-miR-1275 | 1 |
|  | hsa-miR-769-3p | 1 |  | hsa-miR-92a-1* | 1 |
|  | hsa-miR-183* | 1 |  | hsa-miR-1286 | 1 |
|  | hsa-miR-493 | 1 |  | hsa-miR-516a-3p | 1 |
|  | hsa-miR-1233 | 1 |  | hsa-miR-424 | 1 |
|  | hsa-miR-642 | 1 |  | hsa-miR-663 | 1 |
|  | hsa-miR-493* | 1 |  | hsa-miR-30c-1* | 1 |
|  | hsa-miR-541* | 1 |  | hsa-miR-18a* | 1 |
|  | hsa-miR-500 | 1 |  | hsa-miR-941 | 1 |
|  | hsa-miR-519e | 1 |  | hsa-miR-602 | 1 |
|  | hsa-miR-664* | 1 |  | hsa-miR-30b* | 1 |
|  | hsa-miR-198 | 1 |  | hsa-miR-545 | 1 |
|  | hsa-miR-617 | 1 |  | hsa-miR-127-5p | 1 |
|  | hsa-miR-657 | 1 |  | hsa-miR-342-5p | 1 |
|  | hsa-miR-18a* | 1 |  | hsa-miR-411 | 1 |
|  | hsa-miR-769-5p | 1 |  | hsa-miR-486-5p | 1 |
|  | hsa-miR-636 | 1 |  | hsa-miR-1909 | 1 |
|  | hsa-miR-648 | 1 |  | hsa-miR-611 | 1 |
|  | hsa-miR-138-2* | 1 |  | hsa-miR-214* | 1 |
|  | hsa-miR-588 | 1 |  | hsa-miR-145 | 1 |
|  | hsa-miR-193b* | 1 |  | hsa-miR-15a | 1 |
|  | hsa-miR-340 | 1 |  | hsa-miR-943 | 1 |
|  | hsa-miR-99b | 1 |  | hsa-miR-503 | 1 |
|  | hsa-miR-1470 | 1 |  | hsa-miR-18a* | 1 |
|  | hsa-miR-1306 | 1 |  | hsa-miR-1291 | 1 |
|  | hsa-miR-523 | 1 |  | hsa-miR-220c | 1 |
|  | hsa-miR-601 | 1 |  | hsa-miR-508-5p | 1 |
|  | hsa-miR-421 | 1 |  | hsa-miR-516a-3p | 1 |
|  | hsa-miR-23a* | 1 |  | hsa-miR-214* | 1 |
|  | hsa-miR-770-5p | 1 |  | hsa-miR-770-5p | 1 |
|  | hsa-miR-1301 | 1 |  | hsa-miR-301a | 1 |
|  | hsa-miR-191* | 1 |  | hsa-miR-512-5p | 1 |
|  | hsa-let-7a* | 1 |  | hsa-miR-342-5p | 1 |
|  | hsa-miR-378 | 1 |  | hsa-miR-424* | 1 |
|  | hsa-miR-299-3p | 1 |  | hsa-miR-876-3p | 1 |
|  | hsa-miR-582-5p | 1 |  | hsa-miR-377* | 1 |
|  | hsa-miR-1909 | 1 |  | hsa-miR-139-3p | 1 |
|  | hsa-miR-221* | 1 |  | hsa-miR-214* | 1 |
|  | hsa-let-7e | 1 |  | hsa-miR-582-5p | 1 |
|  | hsa-miR-589* | 1 |  | hsa-miR-941 | 1 |
|  | hsa-miR-611 | 1 |  | hsa-miR-194* | 1 |
|  | hsa-miR-452* | 1 |  | hsa-miR-611 | 1 |
|  | hsa-miR-616 | 1 |  | hsa-miR-148b* | 1 |
|  | hsa-miR-152 | 1 |  | hsa-miR-1300 | 1 |
|  | hsa-miR-412 | 1 |  | hsa-miR-125a-3p | 1 |
|  | hsa-miR-1296 | 1 |  | hsa-miR-657 | 1 |
|  | hsa-miR-521 | 1 |  | hsa-miR-770-5p | 1 |
|  | hsa-miR-15b | 1 |  | hsa-miR-525-3p | 1 |
|  | hsa-miR-1285 | 1 |  | hsa-miR-15a | 1 |
|  | hsa-miR-133a | 1 |  | hsa-miR-512-5p | 1 |
|  | hsa-let-7a* | 1 |  | hsa-miR-148a* | 1 |
|  | hsa-miR-766 | 1 |  | hsa-miR-411* | 1 |
|  | hsa-miR-22 | 1 |  | hsa-miR-145* | 1 |
|  | hsa-miR-1910 | 1 |  | hsa-miR-1301 | 1 |
|  | hsa-miR-15b* | 1 |  | hsa-miR-151-3p | 1 |
|  | hsa-miR-214 | 1 |  | hsa-miR-424* | 1 |
|  | hsa-miR-605 | 1 |  | hsa-miR-1182 | 1 |
|  | hsa-miR-497 | 1 |  | hsa-miR-24 | 1 |
|  | hsa-miR-1206 | 1 |  | hsa-miR-139-3p | 1 |
|  | hsa-miR-640 | 1 |  | hsa-miR-329 | 1 |
|  | hsa-miR-210 | 1 |  | hsa-miR-1201 | 1 |
|  | hsa-miR-575 | 1 |  | hsa-miR-941 | 1 |
|  | hsa-miR-561 | 1 |  | hsa-miR-609 | 1 |
|  | hsa-let-7b* | 1 |  | hsa-miR-128 | 1 |
|  | hsa-let-7b | 1 |  | hsa-miR-575 | 1 |
|  | hsa-miR-346 | 1 |  | hsa-miR-146a | 1 |
|  | hsa-miR-1915 | 1 |  | hsa-miR-877 | 1 |
|  | hsa-miR-623 | 1 |  | hsa-miR-411* | 1 |
|  | hsa-miR-638 | 1 |  | hsa-miR-484 | 1 |
|  | hsa-miR-614 | 1 |  | hsa-miR-1911* | 1 |
|  | hsa-miR-652 | 1 |  | hsa-miR-617 | 1 |
|  | hsa-miR-554 | 1 |  | hsa-miR-500* | 1 |
|  | hsa-miR-516b | 1 |  | hsa-miR-145* | 1 |
|  | hsa-miR-1300 | 1 |  | hsa-miR-1207-5p | 1 |
|  | hsa-miR-25* | 1 |  | hsa-miR-1909 | 1 |
|  | hsa-miR-324-5p | 1 |  | hsa-miR-103 | 1 |
|  | hsa-miR-629 | 1 |  | hsa-miR-886-5p | 1 |
|  | hsa-let-7f-1* | 1 |  | hsa-miR-505* | 1 |
|  | hsa-miR-206 | 1 |  | hsa-miR-24 | 1 |
|  | hsa-miR-345 | 1 |  | hsa-miR-195 | 1 |
|  | hsa-miR-432 | 1 |  | hsa-miR-744* | 1 |
|  | hsa-miR-622 | 1 |  | hsa-miR-877 | 1 |
|  | hsa-miR-639 | 1 |  | hsa-miR-455-3p | 1 |
| NTF3 | hsa-miR-182 | 1 |  | hsa-miR-16 | 1 |
|  | hsa-miR-675b | 1 |  | hsa-miR-675 | 1 |
|  | hsa-miR-21 | 7 |  | hsa-miR-296-5p | 1 |
|  | hsa-miR-200b | 7 |  | hsa-miR-525-3p | 1 |
|  | hsa-miR-448 | 7 |  | hsa-miR-151-3p | 1 |
|  | hsa-miR-429 | 7 |  | hsa-miR-16 | 1 |
|  | hsa-miR-200c | 6 |  | hsa-miR-18b | 1 |
|  | hsa-miR-221 | 6 |  | hsa-miR-550 | 1 |
|  | hsa-miR-338-5p | 6 |  | hsa-miR-27a | 1 |
|  | hsa-miR-548d-5p | 6 |  | hsa-miR-1267 | 1 |
|  | hsa-miR-222 | 6 |  | hsa-miR-941 | 1 |
|  | hsa-miR-31 | 6 |  | hsa-miR-411* | 1 |
|  | hsa-miR-296-3p | 6 |  | hsa-miR-30c-1* | 1 |
|  | hsa-miR-548b-5p | 6 |  | hsa-miR-617 | 1 |
|  | hsa-miR-590-5p | 6 |  | hsa-miR-338-3p | 1 |
|  | hsa-miR-132 | 6 |  | hsa-miR-1253 | 1 |
|  | hsa-miR-374a | 6 |  | hsa-miR-1301 | 1 |
|  | hsa-miR-548a-5p | 6 |  | hsa-miR-675 | 1 |
|  | hsa-miR-548c-5p | 6 |  | hsa-miR-524-3p | 1 |
|  | hsa-miR-559 | 6 |  | hsa-miR-16 | 1 |
|  | hsa-miR-374b | 6 |  | hsa-miR-525-3p | 1 |
|  | hsa-miR-212 | 6 |  | hsa-miR-139-3p | 1 |
|  | hsa-miR-222 | 5 |  | hsa-miR-146b-5p | 1 |
|  | hsa-miR-548i | 5 |  | hsa-miR-936 | 1 |
|  | hsa-miR-580 | 5 |  | hsa-miR-148b* | 1 |
|  | hsa-miR-548h | 5 |  | hsa-miR-508-5p | 1 |
|  | hsa-miR-548j | 5 |  | hsa-miR-18b | 1 |
|  | hsa-miR-410 | 5 |  | hsa-miR-939 | 1 |
|  | hsa-miR-21 | 5 |  | hsa-miR-24 | 1 |
|  | hsa-miR-221 | 5 |  | hsa-miR-1268 | 1 |
|  | hsa-miR-548d-5p | 4 |  | hsa-miR-1207-3p | 1 |
|  | hsa-miR-573 | 4 |  | hsa-miR-941 | 1 |
|  | hsa-miR-200b | 4 |  | hsa-miR-611 | 1 |
|  | hsa-miR-1183 | 4 |  | hsa-miR-143* | 1 |
|  | hsa-miR-1200 | 4 |  | hsa-miR-550 | 1 |
|  | hsa-miR-185 | 4 |  | hsa-miR-146a* | 1 |
|  | hsa-miR-543 | 4 |  | hsa-miR-877* | 1 |
|  | hsa-miR-200c | 4 |  | hsa-miR-486-5p | 1 |
|  | hsa-miR-1259 | 4 |  | hsa-miR-146b-5p | 1 |
|  | hsa-miR-338-5p | 3 |  | hsa-miR-18a | 1 |
|  | hsa-miR-548i | 3 |  | hsa-miR-637 | 1 |
|  | hsa-miR-369-3p | 3 |  | hsa-miR-216a | 1 |
|  | hsa-miR-548d-5p | 3 |  | hsa-miR-18b* | 1 |
|  | hsa-miR-548k | 3 |  | hsa-miR-362-3p | 1 |
|  | hsa-miR-548h | 3 |  | hsa-miR-1911* | 1 |
|  | hsa-miR-222* | 3 |  | hsa-miR-103 | 1 |
|  | hsa-miR-580 | 3 |  | hsa-miR-708 | 1 |
|  | hsa-miR-939 | 3 |  | hsa-miR-92b* | 1 |
|  | hsa-miR-548l | 3 |  | hsa-miR-143* | 1 |
|  | hsa-miR-296-3p | 3 |  | hsa-miR-24 | 1 |
|  | hsa-miR-548h | 3 |  | hsa-miR-665 | 1 |
|  | hsa-miR-548b-5p | 3 |  | hsa-miR-877* | 1 |
|  | hsa-miR-448 | 3 |  | hsa-miR-545* | 1 |
|  | hsa-miR-548d-3p | 3 |  | hsa-miR-30c-2* | 1 |
|  | hsa-miR-548h | 3 |  | hsa-miR-508-5p | 1 |
|  | hsa-miR-590-5p | 3 |  | hsa-miR-886-5p | 1 |
|  | hsa-miR-299-3p | 3 |  | hsa-miR-130b* | 1 |
|  | hsa-miR-132 | 3 |  | hsa-miR-524-3p | 1 |
|  | hsa-miR-320c | 3 |  | hsa-miR-148b* | 1 |
|  | hsa-miR-539 | 3 |  | hsa-miR-339-3p | 1 |
|  | hsa-miR-429 | 3 |  | hsa-miR-18b* | 1 |
|  | hsa-miR-374a | 3 |  | hsa-miR-550 | 1 |
|  | hsa-miR-548a-5p | 3 |  | hsa-miR-34a* | 1 |
|  | hsa-miR-526b | 3 |  | hsa-miR-216a | 1 |
|  | hsa-miR-320d | 3 |  | hsa-miR-15b | 1 |
|  | hsa-miR-21* | 3 |  | hsa-miR-1275 | 1 |
|  | hsa-miR-552 | 3 |  | hsa-miR-941 | 1 |
|  | hsa-miR-410 | 3 |  | hsa-miR-671-5p | 1 |
|  | hsa-miR-487a | 3 |  | hsa-miR-34b* | 1 |
|  | hsa-miR-320b | 3 |  | hsa-miR-637 | 1 |
|  | hsa-miR-548i | 3 |  | hsa-miR-1266 | 1 |
|  | hsa-miR-628-5p | 3 |  | hsa-miR-657 | 1 |
|  | hsa-miR-548c-5p | 3 |  | hsa-miR-454 | 1 |
|  | hsa-miR-1300 | 3 |  | hsa-miR-886-5p | 1 |
|  | hsa-miR-561 | 3 |  | hsa-miR-518e* | 1 |
|  | hsa-miR-559 | 3 |  | hsa-miR-25* | 1 |
|  | hsa-miR-374b | 3 |  | hsa-miR-524-3p | 1 |
|  | hsa-miR-212 | 3 |  | hsa-miR-34a* | 1 |
|  | hsa-miR-548m | 3 |  | hsa-miR-127-5p | 1 |
|  | hsa-miR-548i | 3 |  | hsa-miR-130b* | 1 |
|  | hsa-miR-548d-5p | 3 |  | hsa-miR-423-5p | 1 |
|  | hsa-miR-320a | 3 |  | hsa-miR-30c-2* | 1 |
|  | hsa-miR-221* | 3 |  | hsa-miR-128 | 1 |
|  | hsa-miR-135b* | 2 |  | hsa-miR-28-5p | 1 |
|  | hsa-miR-548o | 2 |  | hsa-miR-34a* | 1 |
|  | hsa-miR-548i | 2 |  | hsa-miR-940 | 1 |
|  | hsa-miR-643 | 2 |  | hsa-miR-941 | 1 |
|  | hsa-miR-548h | 2 |  | hsa-miR-617 | 1 |
|  | hsa-miR-518a-5p | 2 |  | hsa-miR-125a-3p | 1 |
|  | hsa-miR-570 | 2 |  | hsa-miR-550 | 1 |
|  | hsa-miR-1183 | 2 |  | hsa-miR-130b* | 1 |
|  | hsa-miR-493 | 2 |  | hsa-miR-146b-5p | 1 |
|  | hsa-miR-30d | 2 |  | hsa-miR-492 | 1 |
|  | hsa-miR-637 | 2 |  | hsa-miR-18a* | 1 |
|  | hsa-miR-513b | 2 |  | hsa-miR-505* | 1 |
|  | hsa-miR-124 | 2 |  | hsa-miR-130a* | 1 |
|  | hsa-miR-380 | 2 |  | hsa-miR-127-5p | 1 |
|  | hsa-miR-31 | 2 |  | hsa-miR-139-5p | 1 |
|  | hsa-miR-29a* | 2 |  | hsa-miR-1912 | 1 |
|  | hsa-miR-548d-3p | 2 |  | hsa-miR-220b | 1 |
|  | hsa-miR-30c-1* | 2 |  | hsa-miR-708* | 1 |
|  | hsa-miR-548h | 2 |  | hsa-miR-1229 | 1 |
|  | hsa-miR-527 | 2 |  | hsa-miR-28-5p | 1 |
|  | hsa-miR-200b* | 2 |  | hsa-miR-1197 | 1 |
|  | hsa-miR-577 | 2 |  | hsa-miR-301b | 1 |
|  | hsa-miR-299-5p | 2 |  | hsa-miR-545 | 1 |
|  | hsa-miR-1200 | 2 |  | hsa-miR-10a | 1 |
|  | hsa-miR-889 | 2 |  | hsa-miR-130a* | 1 |
|  | hsa-miR-320d | 2 |  | hsa-miR-130b | 1 |
|  | hsa-miR-20b* | 2 |  | hsa-miR-518e* | 1 |
|  | hsa-miR-514 | 2 |  | hsa-miR-424* | 1 |
|  | hsa-miR-340 | 2 |  | hsa-miR-602 | 1 |
|  | hsa-miR-30a | 2 |  | hsa-miR-183 | 1 |
|  | hsa-miR-320b | 2 |  | hsa-miR-130a* | 1 |
|  | hsa-miR-1253 | 2 |  | hsa-miR-1201 | 1 |
|  | hsa-miR-548h | 2 |  | hsa-miR-1308 | 1 |
|  | hsa-miR-505 | 2 |  | hsa-miR-941 | 1 |
|  | hsa-miR-586 | 2 |  | hsa-miR-767-5p | 1 |
|  | hsa-miR-144 | 2 |  | hsa-miR-367* | 1 |
|  | hsa-miR-548j | 2 |  | hsa-miR-646 | 1 |
|  | hsa-miR-320c | 2 |  | hsa-miR-1321 | 1 |
|  | hsa-miR-192* | 2 |  | hsa-miR-220c | 1 |
|  | hsa-miR-302c* | 2 |  | hsa-miR-518e | 1 |
|  | hsa-miR-548i | 2 |  | hsa-miR-92a-1* | 1 |
|  | hsa-miR-506 | 2 |  | hsa-miR-518e* | 1 |
|  | hsa-miR-127-3p | 2 |  | hsa-miR-183 | 1 |
|  | hsa-miR-30b | 2 |  | hsa-miR-671-5p | 1 |
|  | hsa-miR-200c* | 2 |  | hsa-miR-146a | 1 |
|  | hsa-miR-590-3p | 2 |  | hsa-miR-497 | 1 |
|  | hsa-miR-30e | 2 |  | hsa-miR-338-3p | 1 |
|  | hsa-miR-1300 | 2 |  | hsa-miR-455-3p | 1 |
|  | hsa-miR-185 | 2 |  | hsa-miR-10b | 1 |
|  | hsa-miR-543 | 2 |  | hsa-miR-183 | 1 |
|  | hsa-miR-30c-2* | 2 |  | hsa-miR-513a-5p | 1 |
|  | hsa-miR-548i | 2 |  | hsa-miR-1291 | 1 |
|  | hsa-miR-134 | 2 |  | hsa-miR-941 | 1 |
|  | hsa-miR-153 | 2 |  | hsa-miR-149* | 1 |
|  | hsa-miR-1201 | 2 |  | hsa-miR-602 | 1 |
|  | hsa-miR-1259 | 2 |  | hsa-miR-330-3p | 1 |
|  | hsa-miR-517b | 2 |  | hsa-miR-671-5p | 1 |
|  | hsa-miR-142-5p | 2 |  | hsa-miR-497* | 1 |
|  | hsa-miR-491-5p | 2 |  | hsa-miR-497 | 1 |
|  | hsa-miR-650 | 2 |  | hsa-miR-24 | 1 |
|  | hsa-miR-10b* | 2 |  | hsa-miR-130a | 1 |
|  | hsa-miR-1323 | 2 |  | hsa-miR-412 | 1 |
|  | hsa-miR-378* | 2 |  | hsa-miR-146a | 1 |
|  | hsa-miR-548i | 2 |  | hsa-miR-10a | 1 |
|  | hsa-miR-138 | 2 |  | hsa-miR-1915 | 1 |
|  | hsa-miR-661 | 2 |  | hsa-miR-920 | 1 |
|  | hsa-miR-633 | 2 |  | hsa-miR-877 | 1 |
|  | hsa-miR-1204 | 2 |  | hsa-miR-593* | 1 |
|  | hsa-miR-548h | 2 |  | hsa-miR-338-3p | 1 |
|  | hsa-miR-568 | 2 |  | hsa-miR-596 | 1 |
|  | hsa-miR-573 | 2 |  | hsa-miR-149* | 1 |
|  | hsa-miR-30c | 2 |  | hsa-miR-886-3p | 1 |
|  | hsa-miR-939 | 2 |  | hsa-miR-939 | 1 |
|  | hsa-miR-106b | 1 |  | hsa-miR-550 | 1 |
|  | hsa-miR-514 | 1 |  | hsa-miR-10b | 1 |
|  | hsa-miR-149* | 1 |  | hsa-miR-455-3p | 1 |
|  | hsa-miR-124 | 1 |  | hsa-miR-145 | 1 |
|  | hsa-miR-9* | 1 |  | hsa-miR-708 | 1 |
|  | hsa-miR-29a* | 1 |  | hsa-miR-518e | 1 |
|  | hsa-miR-374b* | 1 |  | hsa-miR-661 | 1 |
|  | hsa-miR-520g | 1 |  | hsa-miR-18b | 1 |
|  | hsa-miR-1243 | 1 |  | hsa-miR-412 | 1 |
|  | hsa-miR-30c-1* | 1 |  | hsa-miR-183* | 1 |
|  | hsa-let-7g* | 1 |  | hsa-miR-454 | 1 |
|  | hsa-miR-301a | 1 |  | hsa-miR-941 | 1 |
|  | hsa-miR-221* | 1 |  | hsa-miR-766 | 1 |
|  | hsa-miR-765 | 1 |  | hsa-miR-377* | 1 |
|  | hsa-miR-1275 | 1 |  | hsa-miR-1324 | 1 |
|  | hsa-miR-200a | 1 |  | hsa-miR-503 | 1 |
|  | hsa-miR-143 | 1 |  | hsa-miR-298 | 1 |
|  | hsa-miR-492 | 1 |  | hsa-miR-708 | 1 |
|  | hsa-miR-891b | 1 |  | hsa-miR-516a-3p | 1 |
|  | hsa-miR-510 | 1 |  | hsa-miR-518e | 1 |
|  | hsa-miR-320d | 1 |  | hsa-miR-183* | 1 |
|  | hsa-miR-514 | 1 |  | hsa-miR-767-5p | 1 |
|  | hsa-miR-20b* | 1 |  | hsa-miR-146a* | 1 |
|  | hsa-miR-154* | 1 |  | hsa-miR-497* | 1 |
|  | hsa-miR-320b | 1 |  | hsa-miR-221* | 1 |
|  | hsa-miR-9* | 1 |  | hsa-miR-339-3p | 1 |
|  | hsa-miR-23a* | 1 |  | hsa-miR-545* | 1 |
|  | hsa-miR-125b | 1 |  | hsa-miR-597 | 1 |
|  | hsa-miR-135b | 1 |  | hsa-miR-148a* | 1 |
|  | hsa-miR-23b* | 1 |  | hsa-miR-183* | 1 |
|  | hsa-miR-625 | 1 |  | hsa-miR-665 | 1 |
|  | hsa-miR-222* | 1 |  | hsa-miR-941 | 1 |
|  | hsa-miR-611 | 1 |  | hsa-miR-195 | 1 |
|  | hsa-miR-1290 | 1 |  | hsa-miR-603 | 1 |
|  | hsa-miR-144* | 1 |  | hsa-miR-342-5p | 1 |
|  | hsa-miR-494 | 1 |  | hsa-miR-767-5p | 1 |
|  | hsa-miR-7-1* | 1 |  | hsa-miR-512-5p | 1 |
|  | hsa-miR-7 | 1 |  | hsa-miR-497* | 1 |
|  | hsa-miR-423-5p | 1 |  | hsa-miR-24 | 1 |
|  | hsa-miR-515-3p | 1 |  | hsa-miR-92b* | 1 |
|  | hsa-miR-1287 | 1 |  | hsa-miR-484 | 1 |
|  | hsa-miR-320c | 1 |  | hsa-miR-146a* | 1 |
|  | hsa-miR-194 | 1 |  | hsa-miR-1302 | 1 |
|  | hsa-miR-135a* | 1 |  | hsa-miR-25* | 1 |
|  | hsa-miR-320c | 1 |  | hsa-miR-1226 | 1 |
|  | hsa-miR-125a-3p | 1 |  | hsa-miR-877* | 1 |
|  | hsa-miR-192* | 1 |  | hsa-miR-596 | 1 |
|  | hsa-miR-345 | 1 |  | hsa-miR-516a-3p | 1 |
|  | hsa-miR-940 | 1 |  | hsa-miR-339-3p | 1 |
|  | hsa-miR-302c* | 1 |  | hsa-miR-129-3p | 1 |
|  | hsa-miR-125b-2* | 1 |  | hsa-miR-194* | 1 |
|  | hsa-miR-27b* | 1 |  | hsa-miR-18a | 1 |
|  | hsa-miR-130b | 1 |  | hsa-miR-148a* | 1 |
|  | hsa-miR-200b* | 1 |  | hsa-miR-1267 | 1 |
|  | hsa-miR-663 | 1 |  | hsa-miR-940 | 1 |
|  | hsa-miR-19a | 1 |  | hsa-miR-550 | 1 |
|  | hsa-miR-132* | 1 |  | hsa-miR-221* | 1 |
|  | hsa-miR-944 | 1 |  | hsa-miR-545* | 1 |
|  | hsa-miR-181d | 1 |  | hsa-miR-145* | 1 |
|  | hsa-miR-7-2* | 1 |  | hsa-miR-708* | 1 |
|  | hsa-miR-769-5p | 1 |  | hsa-miR-330-3p | 1 |
|  | hsa-miR-101 | 1 |  | hsa-miR-18b* | 1 |
|  | hsa-miR-15b | 1 |  | hsa-miR-484 | 1 |
|  | hsa-miR-376c | 1 |  | hsa-miR-609 | 1 |
|  | hsa-miR-320d | 1 |  | hsa-miR-205 | 1 |
|  | hsa-miR-155* | 1 |  | hsa-miR-1296 | 1 |
|  | hsa-miR-138 | 1 |  | hsa-miR-1197 | 1 |
|  | hsa-miR-320b | 1 |  | hsa-miR-941 | 1 |
|  | hsa-miR-342-5p | 1 |  | hsa-miR-770-5p | 1 |
|  | hsa-miR-30c-2* | 1 |  | hsa-miR-517b | 1 |
|  | hsa-miR-32* | 1 |  | hsa-miR-18a | 1 |
|  | hsa-miR-378 | 1 |  | hsa-miR-1827 | 1 |
|  | hsa-miR-656 | 1 |  | hsa-miR-708* | 1 |
|  | hsa-miR-655 | 1 |  | hsa-miR-516a-3p | 1 |
|  | hsa-miR-1257 | 1 |  | hsa-miR-516a-3p | 1 |
|  | hsa-miR-130a | 1 |  | hsa-miR-205 | 1 |
|  | hsa-miR-132* | 1 |  | hsa-miR-766 | 1 |
|  | hsa-miR-658 | 1 |  | hsa-miR-512-5p | 1 |
|  | hsa-miR-330-3p | 1 |  | hsa-miR-330-3p | 1 |
|  | hsa-miR-153 | 1 |  | hsa-miR-27b | 1 |
|  | hsa-miR-1226* | 1 |  | hsa-miR-30b* | 1 |
|  | hsa-miR-181a-2* | 1 |  | hsa-miR-424 | 1 |
|  | hsa-miR-135a | 1 |  | hsa-miR-545 | 1 |
|  | hsa-miR-497 | 1 |  | hsa-miR-1207-3p | 1 |
|  | hsa-miR-1321 | 1 |  | hsa-miR-367* | 1 |
|  | hsa-miR-181b | 1 |  | hsa-miR-205 | 1 |
|  | hsa-miR-141 | 1 |  | hsa-miR-1254 | 1 |
|  | hsa-miR-10b* | 1 |  | hsa-miR-941 | 1 |
|  | hsa-miR-93* | 1 |  | hsa-miR-128 | 1 |
|  | hsa-miR-638 | 1 |  |  |  |
|  | hsa-miR-301b | 1 |  |  |  |
|  | hsa-miR-421 | 1 |  |  |  |
|  | hsa-miR-548n | 1 |  |  |  |
|  | hsa-miR-514 | 1 |  |  |  |
|  | hsa-miR-378* | 1 |  |  |  |
|  | hsa-miR-30b* | 1 |  |  |  |
|  | hsa-miR-20b | 1 |  |  |  |
|  | hsa-miR-200c* | 1 |  |  |  |
|  | hsa-miR-520h | 1 |  |  |  |
|  | hsa-miR-653 | 1 |  |  |  |
|  | hsa-miR-374a* | 1 |  |  |  |
|  | hsa-miR-302b* | 1 |  |  |  |
|  | hsa-miR-320a | 1 |  |  |  |
|  | hsa-miR-181b | 1 |  |  |  |
|  | hsa-miR-454 | 1 |  |  |  |
|  | hsa-miR-21* | 1 |  |  |  |
|  | hsa-miR-608 | 1 |  |  |  |
|  | hsa-miR-19b | 1 |  |  |  |
|  | hsa-miR-31* | 1 |  |  |  |
|  | hsa-miR-194 | 1 |  |  |  |
|  | hsa-miR-100* | 1 |  |  |  |
|  | hsa-miR-452 | 1 |  |  |  |
|  | hsa-miR-514 | 1 |  |  |  |
|  | hsa-miR-135b* | 1 |  |  |  |
|  | hsa-miR-124 | 1 |  |  |  |
|  | hsa-miR-93 | 1 |  |  |  |
|  | hsa-miR-302d* | 1 |  |  |  |
|  | hsa-miR-9* | 1 |  |  |  |
|  | hsa-miR-374a* | 1 |  |  |  |
|  | hsa-miR-198 | 1 |  |  |  |
|  | hsa-miR-374b* | 1 |  |  |  |
|  | hsa-miR-518a-5p | 1 |  |  |  |
|  | hsa-miR-187* | 1 |  |  |  |
|  | hsa-miR-1278 | 1 |  |  |  |
|  | hsa-miR-31* | 1 |  |  |  |
|  | hsa-miR-644 | 1 |  |  |  |
|  | hsa-miR-1276 | 1 |  |  |  |
|  | hsa-miR-142-3p | 1 |  |  |  |
|  | hsa-miR-493* | 1 |  |  |  |
|  | hsa-miR-30c | 1 |  |  |  |
|  | hsa-miR-555 | 1 |  |  |  |
| **Key AD Pathway gene tergeting predicted miRs from miRWalk** | | | | | |
| **Gene Name** | **MicroRNA** | **Score** | **Gene Name** | **MicroRNA** | **Score** |
| APBB1 | hsa-miR-370 | 3 | IDE | hsa-miR-7 | 4 |
|  | hsa-miR-661 | 3 |  | hsa-miR-590-3p | 4 |
|  | hsa-miR-1255a | 3 |  | hsa-miR-607 | 4 |
|  | hsa-miR-148b | 3 |  | hsa-miR-513a-3p | 4 |
|  | hsa-miR-1255b | 3 |  | hsa-miR-510 | 4 |
|  | hsa-miR-516a-3p | 3 |  | hsa-miR-634 | 4 |
|  | hsa-miR-199a-5p | 3 |  | hsa-miR-1276 | 4 |
|  | hsa-miR-1255b | 3 |  | hsa-miR-374a | 4 |
|  | hsa-miR-516a-3p | 3 |  | hsa-miR-532-5p | 4 |
|  | hsa-miR-324-3p | 3 |  | hsa-miR-556-3p | 4 |
|  | hsa-miR-199b-5p | 3 |  | hsa-miR-374b | 4 |
|  | hsa-miR-665 | 3 |  | hsa-miR-664 | 4 |
|  | hsa-miR-1324 | 3 |  | hsa-miR-760 | 3 |
|  | hsa-miR-423-5p | 3 |  | hsa-miR-579 | 3 |
|  | hsa-miR-1825 | 3 |  | hsa-miR-1179 | 3 |
|  | hsa-miR-508-5p | 3 |  | hsa-miR-409-3p | 3 |
|  | hsa-miR-152 | 3 |  | hsa-miR-1290 | 3 |
|  | hsa-miR-922 | 3 |  | hsa-miR-410 | 3 |
|  | hsa-miR-199a-5p | 3 |  | hsa-miR-20a | 3 |
|  | hsa-miR-494 | 3 |  | hsa-miR-1297 | 3 |
| PSENEN | hsa-miR-1255b | 1 |  | hsa-miR-610 | 3 |
|  | hsa-miR-1275 | 4 |  | hsa-miR-26a | 3 |
|  | hsa-miR-1302 | 3 |  | hsa-miR-1299 | 3 |
|  | hsa-miR-484 | 3 |  | hsa-miR-513a-3p | 3 |
|  | hsa-miR-1302 | 3 |  | hsa-miR-26b | 3 |
|  | hsa-miR-1302 | 3 |  | hsa-miR-1304 | 3 |
|  | hsa-miR-455-5p | 3 |  | hsa-miR-106b | 3 |
|  | hsa-miR-1302 | 3 |  | hsa-miR-633 | 3 |
|  | hsa-miR-1302 | 3 |  | hsa-miR-26a | 3 |
|  | hsa-miR-570 | 3 |  | hsa-miR-1252 | 3 |
|  | hsa-miR-1302 | 3 |  | hsa-miR-340 | 3 |
|  | hsa-miR-580 | 3 |  | hsa-miR-876-5p | 3 |
|  | hsa-miR-1302 | 3 |  | hsa-miR-7 | 3 |
|  | hsa-miR-1264 | 3 |  | hsa-miR-338-3p | 3 |
|  | hsa-miR-1302 | 3 |  | hsa-miR-7 | 3 |
|  | hsa-miR-298 | 3 |  | hsa-miR-571 | 3 |
|  | hsa-miR-1302 | 3 |  | hsa-miR-93 | 3 |
|  | hsa-miR-1302 | 3 |  | hsa-miR-514 | 2 |
|  | hsa-miR-296-5p | 3 |  | hsa-miR-129-5p | 2 |
|  | hsa-miR-922 | 3 |  | hsa-miR-202 | 2 |
|  | hsa-miR-1302 | 3 |  | hsa-miR-452* | 2 |
|  | hsa-miR-382 | 2 |  | hsa-miR-181a-2* | 2 |
|  | hsa-miR-1300 | 2 |  | hsa-miR-545 | 2 |
|  | hsa-miR-1258 | 2 |  | hsa-miR-1185 | 2 |
|  | hsa-miR-625 | 2 |  | hsa-miR-1305 | 2 |
|  | hsa-miR-16-1* | 2 |  | hsa-miR-181c* | 2 |
|  | hsa-miR-28-5p | 2 |  | hsa-miR-889 | 2 |
|  | hsa-miR-567 | 2 |  | hsa-miR-380 | 2 |
|  | hsa-miR-30c-2* | 2 |  | hsa-miR-1185 | 2 |
|  | hsa-miR-214 | 2 |  | hsa-miR-222* | 2 |
|  | hsa-miR-214* | 2 |  | hsa-miR-330-3p | 2 |
|  | hsa-miR-561 | 2 |  | hsa-miR-518c* | 2 |
|  | hsa-miR-1182 | 2 |  | hsa-miR-629 | 2 |
|  | hsa-miR-30b* | 2 |  | hsa-miR-519d | 2 |
|  | hsa-miR-1253 | 2 |  | hsa-miR-135a* | 2 |
|  | hsa-miR-185* | 2 |  | hsa-miR-613 | 2 |
|  | hsa-miR-30c-1* | 2 |  | hsa-miR-138 | 2 |
|  | hsa-miR-708 | 2 |  | hsa-miR-576-5p | 2 |
|  | hsa-miR-200c* | 1 |  | hsa-miR-324-5p | 2 |
|  | hsa-miR-596 | 1 |  | hsa-miR-105 | 2 |
|  | hsa-miR-1197 | 1 |  | hsa-miR-875-3p | 2 |
|  | hsa-miR-302b* | 1 |  | hsa-miR-29a* | 2 |
|  | hsa-miR-615-5p | 1 |  | hsa-miR-1257 | 2 |
|  | hsa-miR-1909 | 1 |  | hsa-miR-1256 | 2 |
|  | hsa-miR-302d* | 1 |  | hsa-miR-17 | 2 |
|  | hsa-miR-488 | 1 |  | hsa-miR-548d-3p | 2 |
|  | hsa-miR-1915 | 1 |  | hsa-miR-514 | 2 |
|  | hsa-miR-146b-5p | 1 |  | hsa-miR-105 | 2 |
|  | hsa-miR-651 | 1 |  | hsa-miR-873 | 2 |
|  | hsa-miR-505* | 1 |  | hsa-miR-33a* | 2 |
|  | hsa-miR-770-5p | 1 |  | hsa-miR-20b | 2 |
|  | hsa-miR-532-3p | 1 |  | hsa-miR-548d-3p | 2 |
|  | hsa-miR-665 | 1 |  | hsa-miR-514 | 2 |
|  | hsa-miR-219-1-3p | 1 |  | hsa-miR-650 | 2 |
|  | hsa-miR-576-3p | 1 |  | hsa-miR-106a | 2 |
|  | hsa-miR-143* | 1 |  | hsa-miR-127-5p | 2 |
|  | hsa-miR-589 | 1 |  | hsa-miR-192* | 2 |
|  | hsa-miR-1255a | 1 |  | hsa-miR-545* | 2 |
|  | hsa-miR-146a | 1 |  | hsa-miR-514 | 2 |
|  | hsa-miR-550* | 1 |  | hsa-miR-129-5p | 2 |
|  | hsa-miR-1255b | 1 |  | hsa-miR-1308 | 2 |
|  | hsa-miR-150 | 1 |  | hsa-miR-514 | 2 |
|  | hsa-miR-550* | 1 |  | hsa-miR-425 | 2 |
| NCSTN | hsa-miR-339-5p | 4 |  | hsa-miR-548c-3p | 2 |
|  | hsa-miR-504 | 4 |  | hsa-miR-369-3p | 2 |
|  | hsa-miR-186 | 4 |  | hsa-miR-1224-5p | 2 |
|  | hsa-miR-1302 | 3 |  | hsa-miR-299-3p | 1 |
|  | hsa-miR-320c | 3 |  | hsa-miR-586 | 1 |
|  | hsa-miR-320d | 3 |  | hsa-miR-136* | 1 |
|  | hsa-miR-199a-5p | 3 |  | hsa-miR-663b | 1 |
|  | hsa-miR-1302 | 3 |  | hsa-miR-450b-5p | 1 |
|  | hsa-miR-1275 | 3 |  | hsa-let-7g | 1 |
|  | hsa-miR-320b | 3 |  | hsa-miR-515-5p | 1 |
|  | hsa-miR-1825 | 3 |  | hsa-miR-922 | 1 |
|  | hsa-miR-1302 | 3 |  | hsa-miR-621 | 1 |
|  | hsa-miR-1253 | 3 |  | hsa-miR-1 | 1 |
|  | hsa-miR-592 | 3 |  | hsa-miR-376a* | 1 |
|  | hsa-miR-1302 | 3 |  | hsa-miR-1306 | 1 |
|  | hsa-miR-650 | 3 |  | hsa-miR-181d | 1 |
|  | hsa-miR-199b-5p | 3 |  | hsa-miR-9 | 1 |
|  | hsa-miR-1254 | 3 |  | hsa-miR-544 | 1 |
|  | hsa-miR-873 | 3 |  | hsa-miR-96 | 1 |
|  | hsa-miR-593 | 3 |  | hsa-miR-1271 | 1 |
|  | hsa-miR-24 | 3 |  | hsa-miR-147 | 1 |
|  | hsa-miR-1302 | 3 |  | hsa-miR-562 | 1 |
|  | hsa-miR-548n | 3 |  | hsa-miR-34a* | 1 |
|  | hsa-miR-599 | 3 |  | hsa-miR-186 | 1 |
|  | hsa-miR-24 | 3 |  | hsa-miR-548l | 1 |
|  | hsa-miR-1302 | 3 |  | hsa-miR-515-5p | 1 |
|  | hsa-miR-548d-3p | 3 |  | hsa-miR-625 | 1 |
|  | hsa-miR-1266 | 3 |  | hsa-miR-15b* | 1 |
|  | hsa-miR-31 | 3 |  | hsa-miR-379* | 1 |
|  | hsa-miR-1302 | 3 |  | hsa-miR-1827 | 1 |
|  | hsa-miR-140-5p | 3 |  | hsa-miR-520b | 1 |
|  | hsa-miR-1274b | 3 |  | hsa-miR-9 | 1 |
|  | hsa-miR-939 | 3 |  | hsa-miR-100* | 1 |
|  | hsa-miR-608 | 3 |  | hsa-miR-515-3p | 1 |
|  | hsa-miR-1302 | 3 |  | hsa-miR-453 | 1 |
|  | hsa-miR-548d-3p | 3 |  | hsa-miR-567 | 1 |
|  | hsa-miR-1274a | 3 |  | hsa-miR-181a | 1 |
|  | hsa-miR-455-5p | 3 |  | hsa-miR-188-5p | 1 |
|  | hsa-miR-1252 | 3 |  | hsa-miR-493* | 1 |
|  | hsa-miR-1225-5p | 3 |  | hsa-let-7i | 1 |
|  | hsa-miR-615-5p | 3 |  | hsa-miR-625* | 1 |
|  | hsa-miR-1302 | 3 |  | hsa-miR-628-3p | 1 |
|  | hsa-miR-658 | 3 |  | hsa-miR-124* | 1 |
|  | hsa-miR-539 | 3 |  | hsa-miR-330-5p | 1 |
|  | hsa-miR-1321 | 3 |  | hsa-miR-520c-3p | 1 |
|  | hsa-miR-1302 | 3 |  | hsa-miR-9 | 1 |
|  | hsa-miR-320d | 3 |  | hsa-miR-101* | 1 |
|  | hsa-miR-199a-5p | 3 |  | hsa-miR-204 | 1 |
|  | hsa-miR-1302 | 3 |  | hsa-miR-551b* | 1 |
|  | hsa-miR-320a | 3 |  | hsa-miR-142-3p | 1 |
|  | hsa-miR-320b | 3 |  | hsa-miR-181b | 1 |
|  | hsa-miR-558 | 3 |  | hsa-miR-195* | 1 |
|  | hsa-miR-320c | 3 |  | hsa-miR-1246 | 1 |
|  | hsa-miR-548k | 3 |  | hsa-miR-942 | 1 |
|  | hsa-miR-629* | 2 |  | hsa-miR-383 | 1 |
|  | hsa-miR-1224-3p | 2 |  | hsa-miR-124* | 1 |
|  | hsa-miR-199a-3p | 2 |  | hsa-miR-103 | 1 |
|  | hsa-miR-194* | 2 |  | hsa-miR-1298 | 1 |
|  | hsa-miR-548h | 2 |  | hsa-miR-211 | 1 |
|  | hsa-miR-1207-5p | 2 |  | hsa-miR-19a | 1 |
|  | hsa-miR-562 | 2 |  | hsa-miR-485-5p | 1 |
|  | hsa-miR-548l | 2 |  | hsa-miR-570 | 1 |
|  | hsa-miR-641 | 2 |  | hsa-miR-181c | 1 |
|  | hsa-miR-130b* | 2 |  | hsa-miR-206 | 1 |
|  | hsa-miR-548b-5p | 2 |  | hsa-miR-1248 | 1 |
|  | hsa-miR-647 | 2 |  | hsa-miR-326 | 1 |
|  | hsa-miR-199a-3p | 2 |  | hsa-miR-526b* | 1 |
|  | hsa-miR-342-5p | 2 |  | hsa-miR-630 | 1 |
|  | hsa-miR-548i | 2 |  | hsa-miR-516b | 1 |
|  | hsa-miR-892a | 2 |  | hsa-miR-452 | 1 |
|  | hsa-miR-1915 | 2 |  | hsa-miR-561 | 1 |
|  | hsa-let-7d | 2 |  | hsa-miR-103 | 1 |
|  | hsa-miR-548i | 2 |  | hsa-miR-124* | 1 |
|  | hsa-miR-103 | 2 |  | hsa-miR-541* | 1 |
|  | hsa-miR-548d-5p | 2 |  | hsa-miR-19b | 1 |
|  | hsa-miR-199b-3p | 2 |  | hsa-miR-365 | 1 |
|  | hsa-miR-524-5p | 2 |  | hsa-miR-182 | 1 |
|  | hsa-miR-202 | 2 |  | hsa-miR-1 | 1 |
|  | hsa-miR-548i | 2 |  | hsa-miR-1254 | 1 |
|  | hsa-miR-453 | 2 |  | hsa-miR-33b | 1 |
|  | hsa-miR-920 | 2 |  | hsa-miR-516b | 1 |
|  | hsa-miR-103 | 2 |  | hsa-miR-138-2* | 1 |
|  | hsa-miR-637 | 2 |  | hsa-miR-19b | 1 |
|  | hsa-miR-495 | 2 |  | hsa-miR-758 | 1 |
|  | hsa-miR-548i | 2 |  | hsa-miR-583 | 1 |
|  | hsa-miR-936 | 2 |  | hsa-miR-365 | 1 |
|  | hsa-miR-107 | 2 |  | hsa-miR-181a | 1 |
|  | hsa-miR-548a-5p | 2 |  | hsa-miR-155* | 1 |
|  | hsa-miR-145 | 2 |  | hsa-miR-494 | 1 |
|  | hsa-miR-548d-5p | 2 |  | hsa-miR-346 | 1 |
|  | hsa-miR-708 | 2 |  | hsa-miR-137 | 1 |
|  | hsa-miR-1270 | 2 |  | hsa-miR-500 | 1 |
|  | hsa-miR-32* | 2 |  | hsa-miR-217 | 1 |
|  | hsa-miR-149* | 2 |  | hsa-miR-299-5p | 1 |
|  | hsa-miR-555 | 2 |  | hsa-miR-144* | 1 |
|  | hsa-miR-92a-2* | 2 |  | hsa-miR-588 | 1 |
|  | hsa-miR-520d-5p | 2 |  | hsa-miR-219-1-3p | 1 |
|  | hsa-miR-185* | 2 |  | hsa-miR-181b | 1 |
|  | hsa-miR-548h | 2 |  | hsa-miR-548n | 1 |
|  | hsa-miR-1261 | 2 |  | hsa-miR-138 | 1 |
|  | hsa-miR-1226* | 2 |  | hsa-miR-26a-1* | 1 |
|  | hsa-miR-548c-5p | 2 |  | hsa-miR-29b | 1 |
|  | hsa-miR-656 | 2 |  | hsa-miR-576-3p | 1 |
|  | hsa-miR-93* | 2 |  | hsa-miR-936 | 1 |
|  | hsa-miR-548h | 2 |  | hsa-miR-591 | 1 |
|  | hsa-miR-1224-5p | 2 |  | hsa-miR-221 | 1 |
|  | hsa-miR-1227 | 2 |  | hsa-miR-26a-2* | 1 |
|  | hsa-miR-556-3p | 2 |  | hsa-miR-1266 | 1 |
|  | hsa-miR-569 | 2 |  | hsa-miR-489 | 1 |
|  | hsa-miR-548j | 2 |  | hsa-miR-501-5p | 1 |
|  | hsa-miR-548c-3p | 2 |  | hsa-miR-32* | 1 |
|  | hsa-miR-542-3p | 2 |  | hsa-miR-411 | 1 |
|  | hsa-miR-548h | 2 |  | hsa-miR-153 | 1 |
|  | hsa-miR-28-5p | 2 |  | hsa-miR-578 | 1 |
|  | hsa-miR-27b* | 1 |  | hsa-miR-107 | 1 |
|  | hsa-miR-149 | 1 |  | hsa-miR-1226 | 1 |
|  | hsa-miR-363* | 1 |  | hsa-miR-512-5p | 1 |
|  | hsa-miR-577 | 1 |  | hsa-miR-600 | 1 |
|  | hsa-miR-100* | 1 |  | hsa-miR-374a* | 1 |
|  | hsa-miR-98 | 1 |  | hsa-miR-222 | 1 |
|  | hsa-miR-1197 | 1 |  | hsa-miR-361-5p | 1 |
|  | hsa-miR-27b | 1 |  | hsa-miR-548p | 1 |
|  | hsa-miR-582-5p | 1 |  | hsa-miR-491-5p | 1 |
|  | hsa-miR-125b | 1 |  | hsa-miR-33a | 1 |
|  | hsa-miR-541 | 1 |  | hsa-miR-411* | 1 |
|  | hsa-miR-1301 | 1 |  | hsa-miR-101 | 1 |
|  | hsa-miR-186* | 1 |  | hsa-let-7e | 1 |
|  | hsa-miR-377* | 1 |  | hsa-miR-34c-3p | 1 |
|  | hsa-let-7a | 1 |  | hsa-miR-16-2* | 1 |
|  | hsa-miR-1208 | 1 |  | hsa-miR-134 | 1 |
|  | hsa-miR-101 | 1 |  | hsa-miR-1237 | 1 |
|  | hsa-miR-1827 | 1 |  | hsa-miR-512-5p | 1 |
|  | hsa-miR-584 | 1 |  | hsa-miR-921 | 1 |
|  | hsa-miR-138-2* | 1 |  | hsa-miR-223* | 1 |
|  | hsa-miR-23a | 1 |  | hsa-miR-1279 | 1 |
|  | hsa-miR-1283 | 1 |  | hsa-miR-497* | 1 |
|  | hsa-miR-214 | 1 |  | hsa-miR-22 | 1 |
|  | hsa-miR-1286 | 1 | APH1A | hsa-miR-151-5p | 4 |
|  | hsa-miR-195 | 1 |  | hsa-miR-1283 | 4 |
|  | hsa-miR-378* | 1 |  | hsa-miR-1233 | 3 |
|  | hsa-miR-491-5p | 1 |  | hsa-miR-516b | 3 |
|  | hsa-let-7a | 1 |  | hsa-miR-138 | 3 |
|  | hsa-miR-147 | 1 |  | hsa-miR-1184 | 3 |
|  | hsa-miR-455-3p | 1 |  | hsa-miR-220c | 3 |
|  | hsa-miR-590-5p | 1 |  | hsa-miR-1302 | 3 |
|  | hsa-miR-142-5p | 1 |  | hsa-miR-520d-5p | 3 |
|  | hsa-miR-424 | 1 |  | hsa-miR-1302 | 3 |
|  | hsa-miR-769-3p | 1 |  | hsa-miR-188-3p | 3 |
|  | hsa-let-7a | 1 |  | hsa-miR-365 | 3 |
|  | hsa-miR-1287 | 1 |  | hsa-miR-509-3-5p | 3 |
|  | hsa-miR-517* | 1 |  | hsa-miR-151-5p | 3 |
|  | hsa-miR-340 | 1 |  | hsa-miR-182 | 3 |
|  | hsa-miR-654-5p | 1 |  | hsa-miR-522 | 3 |
|  | hsa-miR-7 | 1 |  | hsa-miR-140-3p | 3 |
|  | hsa-miR-561 | 1 |  | hsa-miR-1184 | 3 |
|  | hsa-miR-600 | 1 |  | hsa-miR-1302 | 3 |
|  | hsa-miR-143 | 1 |  | hsa-miR-516b | 3 |
|  | hsa-miR-620 | 1 |  | hsa-miR-1302 | 3 |
|  | hsa-miR-765 | 1 |  | hsa-miR-548c-3p | 3 |
|  | hsa-miR-15b | 1 |  | hsa-miR-365 | 3 |
|  | hsa-let-7b | 1 |  | hsa-miR-939 | 3 |
|  | hsa-miR-1293 | 1 |  | hsa-miR-611 | 3 |
|  | hsa-miR-485-5p | 1 |  | hsa-miR-875-5p | 3 |
|  | hsa-miR-7-1* | 1 |  | hsa-miR-345 | 3 |
|  | hsa-miR-15a | 1 |  | hsa-miR-145 | 3 |
|  | hsa-miR-144 | 1 |  | hsa-miR-1224-3p | 3 |
|  | hsa-miR-628-5p | 1 |  | hsa-miR-220a | 3 |
|  | hsa-miR-450b-3p | 1 |  | hsa-miR-1825 | 3 |
|  | hsa-miR-222* | 1 |  | hsa-miR-1225-3p | 3 |
|  | hsa-miR-517* | 1 |  | hsa-miR-1302 | 3 |
|  | hsa-let-7c | 1 |  | hsa-miR-516b | 3 |
|  | hsa-miR-1294 | 1 |  | hsa-miR-1302 | 3 |
|  | hsa-miR-489 | 1 |  | hsa-miR-620 | 3 |
|  | hsa-miR-7 | 1 |  | hsa-miR-345 | 3 |
|  | hsa-miR-644 | 1 |  | hsa-miR-204 | 3 |
|  | hsa-miR-493 | 1 |  | hsa-miR-612 | 3 |
|  | hsa-let-7e | 1 |  | hsa-miR-876-3p | 3 |
|  | hsa-miR-1251 | 1 |  | hsa-miR-509-5p | 3 |
|  | hsa-miR-619 | 1 |  | hsa-miR-122 | 3 |
|  | hsa-miR-522 | 1 |  | hsa-miR-1228 | 3 |
|  | hsa-miR-16 | 1 |  | hsa-miR-1302 | 3 |
|  | hsa-miR-7-2* | 1 |  | hsa-miR-522 | 3 |
|  | hsa-miR-16 | 1 |  | hsa-miR-1302 | 3 |
|  | hsa-miR-661 | 1 |  | hsa-miR-412 | 3 |
|  | hsa-miR-23b | 1 |  | hsa-miR-1270 | 3 |
|  | hsa-miR-125a-5p | 1 |  | hsa-miR-211 | 3 |
|  | hsa-miR-18b | 1 |  | hsa-miR-615-5p | 3 |
|  | hsa-miR-708* | 1 |  | hsa-miR-1270 | 3 |
|  | hsa-miR-625 | 1 |  | hsa-miR-885-5p | 3 |
|  | hsa-let-7f | 1 |  | hsa-miR-532-3p | 3 |
|  | hsa-miR-1257 | 1 |  | hsa-miR-1301 | 3 |
|  | hsa-miR-27a | 1 |  | hsa-miR-138 | 3 |
|  | hsa-miR-10a | 1 |  | hsa-miR-1233 | 3 |
|  | hsa-miR-7 | 1 |  | hsa-miR-1302 | 3 |
|  | hsa-miR-198 | 1 |  | hsa-miR-1302 | 3 |
|  | hsa-miR-18a | 1 |  | hsa-miR-1224-3p | 3 |
|  | hsa-miR-323-3p | 1 |  | hsa-miR-1184 | 3 |
|  | hsa-miR-411 | 1 |  | hsa-miR-1278 | 3 |
|  | hsa-miR-497 | 1 |  | hsa-miR-197 | 3 |
|  | hsa-miR-125b | 1 |  | hsa-miR-326 | 3 |
|  | hsa-miR-1182 | 1 |  | hsa-miR-412 | 3 |
|  | hsa-miR-1258 | 1 |  | hsa-miR-539 | 3 |
|  | hsa-let-7f | 1 |  | hsa-miR-1283 | 3 |
|  | hsa-miR-559 | 1 |  | hsa-miR-140-3p | 3 |
|  | hsa-let-7g | 1 |  | hsa-miR-1233 | 3 |
|  | hsa-miR-517* | 1 |  | hsa-miR-875-5p | 3 |
|  | hsa-miR-101 | 1 |  | hsa-miR-361-3p | 3 |
|  | hsa-miR-940 | 1 |  | hsa-miR-1302 | 3 |
|  | hsa-miR-21 | 1 |  | hsa-miR-509-5p | 3 |
|  | hsa-miR-1283 | 1 |  | hsa-miR-1302 | 3 |
|  | hsa-miR-10b | 1 |  | hsa-miR-1184 | 3 |
|  | hsa-let-7i | 1 |  | hsa-miR-526b | 3 |
|  | hsa-miR-664 | 1 |  | hsa-miR-1283 | 3 |
|  | hsa-miR-636 | 1 |  | hsa-miR-548c-3p | 3 |
|  | hsa-miR-331-3p | 1 |  | hsa-miR-198 | 3 |
|  | hsa-miR-892b | 1 |  | hsa-miR-922 | 3 |
|  | hsa-miR-299-3p | 1 |  | hsa-miR-1302 | 3 |
|  | hsa-miR-1200 | 1 |  | hsa-miR-136 | 3 |
|  | hsa-miR-1280 | 1 |  | hsa-miR-145 | 3 |
|  | hsa-miR-570 | 1 |  | hsa-miR-876-3p | 3 |
|  |  |  |  | hsa-miR-362-5p | 3 |
|  |  |  |  | hsa-miR-1302 | 3 |
|  |  |  |  | hsa-miR-197 | 3 |
|  |  |  |  | hsa-miR-532-3p | 3 |
|  |  |  |  | hsa-miR-1301 | 3 |
|  |  |  |  | hsa-miR-1184 | 3 |
|  |  |  |  | hsa-miR-220a | 3 |
|  |  |  |  | hsa-miR-620 | 3 |
|  |  |  |  | hsa-miR-1278 | 3 |
|  |  |  |  | hsa-miR-509-3-5p | 3 |
|  |  |  |  | hsa-miR-1302 | 3 |
|  |  |  |  | hsa-miR-582-5p | 3 |
|  |  |  |  | hsa-miR-138 | 3 |
|  |  |  |  | hsa-miR-770-5p | 3 |
|  |  |  |  | hsa-miR-136 | 3 |
|  |  |  |  | hsa-miR-1302 | 3 |
|  |  |  |  | hsa-miR-885-5p | 3 |
|  |  |  |  | hsa-miR-365 | 3 |
|  |  |  |  | hsa-miR-198 | 3 |
|  |  |  |  | hsa-miR-1283 | 3 |
|  |  |  |  | hsa-miR-1225-3p | 3 |
|  |  |  |  | hsa-miR-524-5p | 3 |
|  |  |  |  | hsa-miR-1825 | 3 |
|  |  |  |  | hsa-miR-182 | 3 |
|  |  |  |  | hsa-miR-939 | 3 |
|  |  |  |  | hsa-miR-526b | 3 |
|  |  |  |  | hsa-miR-1302 | 3 |
|  |  |  |  | hsa-miR-138 | 3 |
|  |  |  |  | hsa-miR-1302 | 3 |
|  |  |  |  | hsa-miR-361-3p | 3 |
|  |  |  |  | hsa-miR-365 | 3 |
|  |  |  |  | hsa-miR-539 | 3 |
|  |  |  |  | hsa-miR-770-5p | 3 |
|  |  |  |  | hsa-miR-1228 | 3 |
|  |  |  |  | hsa-miR-520d-5p | 3 |
|  |  |  |  | hsa-miR-635 | 3 |
|  |  |  |  | hsa-miR-204 | 3 |
|  |  |  |  | hsa-miR-1302 | 3 |
|  |  |  |  | hsa-miR-1302 | 3 |
|  |  |  |  | hsa-miR-611 | 3 |
|  |  |  |  | hsa-miR-185 | 3 |
|  |  |  |  | hsa-miR-509-5p | 3 |
|  |  |  |  | hsa-miR-185 | 3 |
|  |  |  |  | hsa-miR-362-5p | 3 |
|  |  |  |  | hsa-miR-922 | 3 |
|  |  |  |  | hsa-miR-323-5p | 3 |
|  |  |  |  | hsa-miR-1233 | 3 |
|  |  |  |  | hsa-miR-516b | 3 |
|  |  |  |  | hsa-miR-122 | 3 |
|  |  |  |  | hsa-miR-211 | 3 |
|  |  |  |  | hsa-miR-1184 | 3 |
|  |  |  |  | hsa-miR-188-3p | 3 |
|  |  |  |  | hsa-miR-1302 | 3 |
|  |  |  |  | hsa-miR-524-5p | 3 |
|  |  |  |  | hsa-miR-1302 | 3 |
|  |  |  |  | hsa-miR-615-5p | 3 |
|  |  |  |  | hsa-miR-34a | 3 |
|  |  |  |  | hsa-miR-582-5p | 3 |
|  |  |  |  | hsa-miR-509-5p | 3 |
|  |  |  |  | hsa-miR-199a-5p | 2 |
|  |  |  |  | hsa-miR-654-5p | 2 |
|  |  |  |  | hsa-miR-218-1* | 2 |
|  |  |  |  | hsa-miR-513b | 2 |
|  |  |  |  | hsa-miR-195* | 2 |
|  |  |  |  | hsa-miR-920 | 2 |
|  |  |  |  | hsa-miR-24 | 2 |
|  |  |  |  | hsa-miR-616* | 2 |
|  |  |  |  | hsa-miR-642 | 2 |
|  |  |  |  | hsa-miR-548f | 2 |
|  |  |  |  | hsa-miR-606 | 2 |
|  |  |  |  | hsa-miR-548f | 2 |
|  |  |  |  | hsa-miR-541* | 2 |
|  |  |  |  | hsa-let-7a | 2 |
|  |  |  |  | hsa-miR-335* | 2 |
|  |  |  |  | hsa-miR-1234 | 2 |
|  |  |  |  | hsa-miR-199b-5p | 2 |
|  |  |  |  | hsa-miR-1285 | 2 |
|  |  |  |  | hsa-miR-548d-3p | 2 |
|  |  |  |  | hsa-miR-657 | 2 |
|  |  |  |  | hsa-miR-218-2* | 2 |
|  |  |  |  | hsa-miR-1321 | 2 |
|  |  |  |  | hsa-miR-888* | 2 |
|  |  |  |  | hsa-miR-194* | 2 |
|  |  |  |  | hsa-miR-24 | 2 |
|  |  |  |  | hsa-miR-199a-5p | 2 |
|  |  |  |  | hsa-miR-199a-5p | 2 |
|  |  |  |  | hsa-miR-920 | 2 |
|  |  |  |  | hsa-miR-195* | 2 |
|  |  |  |  | hsa-miR-1260 | 2 |
|  |  |  |  | hsa-miR-335* | 2 |
|  |  |  |  | hsa-miR-548f | 2 |
|  |  |  |  | hsa-miR-196a | 2 |
|  |  |  |  | hsa-miR-548f | 2 |
|  |  |  |  | hsa-miR-214 | 2 |
|  |  |  |  | hsa-miR-766 | 2 |
|  |  |  |  | hsa-let-7a | 2 |
|  |  |  |  | hsa-miR-1207-5p | 2 |
|  |  |  |  | hsa-miR-502-5p | 2 |
|  |  |  |  | hsa-miR-1285 | 2 |
|  |  |  |  | hsa-miR-548d-3p | 2 |
|  |  |  |  | hsa-miR-194* | 2 |
|  |  |  |  | hsa-miR-892b | 2 |
|  |  |  |  | hsa-miR-34b* | 2 |
|  |  |  |  | hsa-miR-330-5p | 2 |
|  |  |  |  | hsa-miR-92a-2* | 2 |
|  |  |  |  | hsa-miR-302b | 2 |
|  |  |  |  | hsa-miR-940 | 2 |
|  |  |  |  | hsa-miR-1266 | 2 |
|  |  |  |  | hsa-miR-17* | 2 |
|  |  |  |  | hsa-miR-1266 | 2 |
|  |  |  |  | hsa-miR-330-5p | 2 |
|  |  |  |  | hsa-let-7a | 2 |
|  |  |  |  | hsa-miR-196b | 2 |
|  |  |  |  | hsa-miR-548e | 2 |
|  |  |  |  | hsa-miR-214 | 2 |
|  |  |  |  | hsa-miR-9* | 2 |
|  |  |  |  | hsa-miR-1271 | 2 |
|  |  |  |  | hsa-miR-1909 | 2 |
|  |  |  |  | hsa-miR-302a | 2 |
|  |  |  |  | hsa-miR-541* | 2 |
|  |  |  |  | hsa-miR-34b | 2 |
|  |  |  |  | hsa-miR-98 | 2 |
|  |  |  |  | hsa-miR-449b | 2 |
|  |  |  |  | hsa-miR-548d-3p | 2 |
|  |  |  |  | hsa-miR-302c | 2 |
|  |  |  |  | hsa-miR-943 | 2 |
|  |  |  |  | hsa-miR-16-2* | 2 |
|  |  |  |  | hsa-miR-220b | 2 |
|  |  |  |  | hsa-let-7b | 2 |
|  |  |  |  | hsa-miR-449a | 2 |
|  |  |  |  | hsa-miR-1289 | 2 |
|  |  |  |  | hsa-miR-570 | 2 |
|  |  |  |  | hsa-miR-9* | 2 |
|  |  |  |  | hsa-miR-1910 | 2 |
|  |  |  |  | hsa-miR-34b* | 2 |
|  |  |  |  | hsa-miR-541 | 2 |
|  |  |  |  | hsa-miR-34c-5p | 2 |
|  |  |  |  | hsa-miR-326 | 2 |
|  |  |  |  | hsa-miR-16-2* | 2 |
|  |  |  |  | hsa-miR-502-5p | 2 |
|  |  |  |  | hsa-miR-548d-3p | 2 |
|  |  |  |  | hsa-miR-302d | 2 |
|  |  |  |  | hsa-miR-490-3p | 2 |
|  |  |  |  | hsa-miR-635 | 2 |
|  |  |  |  | hsa-miR-218-1* | 2 |
|  |  |  |  | hsa-miR-616* | 2 |
|  |  |  |  | hsa-miR-302e | 2 |
|  |  |  |  | hsa-miR-216b | 2 |
|  |  |  |  | hsa-let-7c | 2 |
|  |  |  |  | hsa-miR-1289 | 2 |
|  |  |  |  | hsa-miR-578 | 2 |
|  |  |  |  | hsa-miR-766 | 2 |
|  |  |  |  | hsa-miR-9* | 2 |
|  |  |  |  | hsa-miR-1915 | 2 |
|  |  |  |  | hsa-miR-34b | 2 |
|  |  |  |  | hsa-miR-196a | 2 |
|  |  |  |  | hsa-miR-1271 | 2 |
|  |  |  |  | hsa-miR-622 | 2 |
|  |  |  |  | hsa-miR-1280 | 2 |
|  |  |  |  | hsa-miR-373* | 2 |
|  |  |  |  | hsa-miR-218-2* | 2 |
|  |  |  |  | hsa-miR-548p | 2 |
|  |  |  |  | hsa-let-7d | 2 |
|  |  |  |  | hsa-miR-490-3p | 2 |
|  |  |  |  | hsa-miR-576-3p | 2 |
|  |  |  |  | hsa-miR-220b | 2 |
|  |  |  |  | hsa-miR-769-3p | 2 |
|  |  |  |  | hsa-miR-548e | 2 |
|  |  |  |  | hsa-miR-34c-5p | 2 |
|  |  |  |  | hsa-miR-1303 | 2 |
|  |  |  |  | hsa-miR-377 | 2 |
|  |  |  |  | hsa-miR-518c* | 2 |
|  |  |  |  | hsa-miR-513b | 2 |
|  |  |  |  | hsa-miR-501-5p | 2 |
|  |  |  |  | hsa-miR-622 | 2 |
|  |  |  |  | hsa-miR-10a* | 2 |
|  |  |  |  | hsa-let-7e | 2 |
|  |  |  |  | hsa-miR-491-5p | 2 |
|  |  |  |  | hsa-miR-570 | 2 |
|  |  |  |  | hsa-miR-1260 | 2 |
|  |  |  |  | hsa-miR-299-3p | 2 |
|  |  |  |  | hsa-miR-1243 | 2 |
|  |  |  |  | hsa-miR-455-3p | 2 |
|  |  |  |  | hsa-miR-1243 | 2 |
|  |  |  |  | hsa-miR-379 | 2 |
|  |  |  |  | hsa-let-7g | 2 |
|  |  |  |  | hsa-miR-625 | 2 |
|  |  |  |  | hsa-let-7f | 2 |
|  |  |  |  | hsa-miR-606 | 2 |
|  |  |  |  | hsa-miR-578 | 2 |
|  |  |  |  | hsa-miR-298 | 2 |
|  |  |  |  | hsa-miR-216b | 2 |
|  |  |  |  | hsa-miR-548f | 2 |
|  |  |  |  | hsa-miR-10a* | 2 |
|  |  |  |  | hsa-miR-548f | 2 |
|  |  |  |  | hsa-miR-372 | 2 |
|  |  |  |  | hsa-miR-330-3p | 2 |
|  |  |  |  | hsa-miR-1915 | 2 |
|  |  |  |  | hsa-let-7i | 2 |
|  |  |  |  | hsa-miR-1252 | 2 |
|  |  |  |  | hsa-miR-940 | 2 |
|  |  |  |  | hsa-miR-323-5p | 2 |
|  |  |  |  | hsa-let-7f | 2 |
|  |  |  |  | hsa-miR-518c* | 2 |
|  |  |  |  | hsa-miR-637 | 2 |
|  |  |  |  | hsa-miR-205 | 2 |
|  |  |  |  | hsa-miR-373* | 2 |
|  |  |  |  | hsa-miR-548f | 2 |
|  |  |  |  | hsa-miR-10b* | 2 |
|  |  |  |  | hsa-miR-576-3p | 2 |
|  |  |  |  | hsa-miR-548f | 2 |
|  |  |  |  | hsa-miR-298 | 2 |
|  |  |  |  | hsa-miR-199a-5p | 2 |
|  |  |  |  | hsa-miR-604 | 2 |
|  |  |  |  | hsa-miR-501-5p | 2 |
|  |  |  |  | hsa-miR-642 | 2 |
|  |  |  |  | hsa-miR-449b | 2 |
|  |  |  |  | hsa-miR-664* | 2 |
|  |  |  |  | hsa-miR-17* | 2 |
|  |  |  |  | hsa-miR-1280 | 2 |
|  |  |  |  | hsa-miR-185* | 2 |
|  |  |  |  | hsa-miR-450b-3p | 2 |
|  |  |  |  | hsa-miR-363* | 2 |
|  |  |  |  | hsa-miR-924 | 2 |
|  |  |  |  | hsa-miR-377 | 2 |
|  |  |  |  | hsa-miR-548f | 2 |
|  |  |  |  | hsa-miR-548f | 2 |
|  |  |  |  | hsa-miR-199b-5p | 2 |
|  |  |  |  | hsa-miR-374a* | 1 |
|  |  |  |  | hsa-miR-1323 | 1 |
|  |  |  |  | hsa-miR-1911 | 1 |
|  |  |  |  | hsa-let-7a | 1 |
|  |  |  |  | hsa-miR-501-3p | 1 |
|  |  |  |  | hsa-miR-508-5p | 1 |
|  |  |  |  | hsa-miR-1208 | 1 |
|  |  |  |  | hsa-miR-649 | 1 |
|  |  |  |  | hsa-miR-125b-2* | 1 |
|  |  |  |  | hsa-miR-449a | 1 |
|  |  |  |  | hsa-miR-371-5p | 1 |
|  |  |  |  | hsa-miR-483-3p | 1 |
|  |  |  |  | hsa-miR-29b | 1 |
|  |  |  |  | hsa-let-7f | 1 |
|  |  |  |  | hsa-miR-29b | 1 |
|  |  |  |  | hsa-miR-548a-3p | 1 |
|  |  |  |  | hsa-miR-135a | 1 |
|  |  |  |  | hsa-miR-589* | 1 |
|  |  |  |  | hsa-miR-1308 | 1 |
|  |  |  |  | hsa-miR-708* | 1 |
|  |  |  |  | hsa-miR-193a-3p | 1 |
|  |  |  |  | hsa-miR-583 | 1 |
|  |  |  |  | hsa-miR-1304 | 1 |
|  |  |  |  | hsa-miR-612 | 1 |
|  |  |  |  | hsa-miR-497* | 1 |
|  |  |  |  | hsa-miR-520a-3p | 1 |
|  |  |  |  | hsa-miR-941 | 1 |
|  |  |  |  | hsa-miR-214* | 1 |
|  |  |  |  | hsa-miR-378 | 1 |
|  |  |  |  | hsa-miR-454 | 1 |
|  |  |  |  | hsa-miR-627 | 1 |
|  |  |  |  | hsa-let-7a | 1 |
|  |  |  |  | hsa-miR-502-3p | 1 |
|  |  |  |  | hsa-miR-510 | 1 |
|  |  |  |  | hsa-miR-126* | 1 |
|  |  |  |  | hsa-miR-374a* | 1 |
|  |  |  |  | hsa-miR-202 | 1 |
|  |  |  |  | hsa-miR-934 | 1 |
|  |  |  |  | hsa-miR-487a | 1 |
|  |  |  |  | hsa-miR-588 | 1 |
|  |  |  |  | hsa-miR-142-3p | 1 |
|  |  |  |  | hsa-miR-590-5p | 1 |
|  |  |  |  | hsa-miR-1197 | 1 |
|  |  |  |  | hsa-miR-320a | 1 |
|  |  |  |  | hsa-miR-647 | 1 |
|  |  |  |  | hsa-miR-484 | 1 |
|  |  |  |  | hsa-miR-512-5p | 1 |
|  |  |  |  | hsa-miR-525-5p | 1 |
|  |  |  |  | hsa-miR-941 | 1 |
|  |  |  |  | hsa-miR-383 | 1 |
|  |  |  |  | hsa-let-7f-2* | 1 |
|  |  |  |  | hsa-miR-506 | 1 |
|  |  |  |  | hsa-miR-193a-5p | 1 |
|  |  |  |  | hsa-miR-214* | 1 |
|  |  |  |  | hsa-miR-545* | 1 |
|  |  |  |  | hsa-miR-150 | 1 |
|  |  |  |  | hsa-let-7i* | 1 |
|  |  |  |  | hsa-miR-193b | 1 |
|  |  |  |  | hsa-miR-941 | 1 |
|  |  |  |  | hsa-miR-148a | 1 |
|  |  |  |  | hsa-miR-148a | 1 |
|  |  |  |  | hsa-miR-548a-3p | 1 |
|  |  |  |  | hsa-miR-143* | 1 |
|  |  |  |  | hsa-miR-593* | 1 |
|  |  |  |  | hsa-miR-1470 | 1 |
|  |  |  |  | hsa-miR-934 | 1 |
|  |  |  |  | hsa-miR-155 | 1 |
|  |  |  |  | hsa-miR-649 | 1 |
|  |  |  |  | hsa-miR-1265 | 1 |
|  |  |  |  | hsa-miR-519c-3p | 1 |
|  |  |  |  | hsa-miR-512-5p | 1 |
|  |  |  |  | hsa-miR-520b | 1 |
|  |  |  |  | hsa-miR-941 | 1 |
|  |  |  |  | hsa-miR-661 | 1 |
|  |  |  |  | hsa-miR-216a | 1 |
|  |  |  |  | hsa-miR-632 | 1 |
|  |  |  |  | hsa-miR-320b | 1 |
|  |  |  |  | hsa-miR-21 | 1 |
|  |  |  |  | hsa-miR-508-5p | 1 |
|  |  |  |  | hsa-miR-551b* | 1 |
|  |  |  |  | hsa-miR-1293 | 1 |
|  |  |  |  | hsa-miR-1224-5p | 1 |
|  |  |  |  | hsa-miR-154 | 1 |
|  |  |  |  | hsa-miR-935 | 1 |
|  |  |  |  | hsa-miR-342-3p | 1 |
|  |  |  |  | hsa-miR-497* | 1 |
|  |  |  |  | hsa-miR-941 | 1 |
|  |  |  |  | hsa-miR-139-5p | 1 |
|  |  |  |  | hsa-miR-98 | 1 |
|  |  |  |  | hsa-miR-139-5p | 1 |
|  |  |  |  | hsa-miR-589* | 1 |
|  |  |  |  | hsa-miR-143 | 1 |
|  |  |  |  | hsa-miR-548a-3p | 1 |
|  |  |  |  | hsa-miR-320d | 1 |
|  |  |  |  | hsa-miR-99b | 1 |
|  |  |  |  | hsa-miR-29c* | 1 |
|  |  |  |  | hsa-miR-548o | 1 |
|  |  |  |  | hsa-miR-515-5p | 1 |
|  |  |  |  | hsa-miR-520c-3p | 1 |
|  |  |  |  | hsa-miR-941 | 1 |
|  |  |  |  | hsa-miR-217 | 1 |
|  |  |  |  | hsa-miR-342-3p | 1 |
|  |  |  |  | hsa-miR-21* | 1 |
|  |  |  |  | hsa-let-7c | 1 |
|  |  |  |  | hsa-let-7f-2* | 1 |
|  |  |  |  | hsa-miR-510 | 1 |
|  |  |  |  | hsa-miR-216a | 1 |
|  |  |  |  | hsa-miR-1299 | 1 |
|  |  |  |  | hsa-miR-1323 | 1 |
|  |  |  |  | hsa-miR-186 | 1 |
|  |  |  |  | hsa-miR-324-3p | 1 |
|  |  |  |  | hsa-miR-1226* | 1 |
|  |  |  |  | hsa-miR-512-5p | 1 |
|  |  |  |  | hsa-miR-941 | 1 |
|  |  |  |  | hsa-miR-342-5p | 1 |
|  |  |  |  | hsa-miR-7-1* | 1 |
|  |  |  |  | hsa-miR-7-1* | 1 |
|  |  |  |  | hsa-miR-125a-3p | 1 |
|  |  |  |  | hsa-miR-607 | 1 |
|  |  |  |  | hsa-miR-320c | 1 |
|  |  |  |  | hsa-miR-29c | 1 |
|  |  |  |  | hsa-miR-548p | 1 |
|  |  |  |  | hsa-miR-519b-3p | 1 |
|  |  |  |  | hsa-miR-519e* | 1 |
|  |  |  |  | hsa-miR-520d-3p | 1 |
|  |  |  |  | hsa-miR-1226* | 1 |
|  |  |  |  | hsa-miR-593* | 1 |
|  |  |  |  | hsa-miR-124 | 1 |
|  |  |  |  | hsa-miR-891b | 1 |
|  |  |  |  | hsa-miR-28-5p | 1 |
|  |  |  |  | hsa-let-7c* | 1 |
|  |  |  |  | hsa-miR-21* | 1 |
|  |  |  |  | hsa-miR-545* | 1 |
|  |  |  |  | hsa-miR-217 | 1 |
|  |  |  |  | hsa-miR-1304 | 1 |
|  |  |  |  | hsa-miR-377* | 1 |
|  |  |  |  | hsa-miR-186* | 1 |
|  |  |  |  | hsa-miR-1201 | 1 |
|  |  |  |  | hsa-miR-135b | 1 |
|  |  |  |  | hsa-miR-512-5p | 1 |
|  |  |  |  | hsa-miR-941 | 1 |
|  |  |  |  | hsa-miR-7-2* | 1 |
|  |  |  |  | hsa-miR-34a | 1 |
|  |  |  |  | hsa-miR-7-2* | 1 |
|  |  |  |  | hsa-miR-126* | 1 |
|  |  |  |  | hsa-miR-320d | 1 |
|  |  |  |  | hsa-miR-301a | 1 |
|  |  |  |  | hsa-miR-668 | 1 |
|  |  |  |  | hsa-miR-515-5p | 1 |
|  |  |  |  | hsa-miR-499-3p | 1 |
|  |  |  |  | hsa-miR-1226 | 1 |
|  |  |  |  | hsa-miR-1258 | 1 |
|  |  |  |  | hsa-miR-548a-3p | 1 |
|  |  |  |  | hsa-miR-124 | 1 |
|  |  |  |  | hsa-miR-188-5p | 1 |
|  |  |  |  | hsa-miR-135b | 1 |
|  |  |  |  | hsa-miR-888 | 1 |
|  |  |  |  | hsa-miR-29a | 1 |
|  |  |  |  | hsa-let-7d | 1 |
|  |  |  |  | hsa-miR-28-5p | 1 |
|  |  |  |  | hsa-miR-551b* | 1 |
|  |  |  |  | hsa-miR-124 | 1 |
|  |  |  |  | hsa-miR-580 | 1 |
|  |  |  |  | hsa-miR-1253 | 1 |
|  |  |  |  | hsa-miR-1911 | 1 |
|  |  |  |  | hsa-miR-1202 | 1 |
|  |  |  |  | hsa-miR-431 | 1 |
|  |  |  |  | hsa-miR-520e | 1 |
|  |  |  |  | hsa-miR-941 | 1 |
|  |  |  |  | hsa-miR-10b* | 1 |
|  |  |  |  | hsa-miR-149 | 1 |
|  |  |  |  | hsa-miR-623 | 1 |
|  |  |  |  | hsa-miR-130b | 1 |
|  |  |  |  | hsa-miR-767-3p | 1 |
|  |  |  |  | hsa-miR-1321 | 1 |
|  |  |  |  | hsa-miR-519a | 1 |
|  |  |  |  | hsa-miR-520a-5p | 1 |
|  |  |  |  | hsa-miR-10a | 1 |
|  |  |  |  | hsa-miR-1201 | 1 |
|  |  |  |  | hsa-miR-124 | 1 |
|  |  |  |  | hsa-miR-155 | 1 |
|  |  |  |  | hsa-miR-422a | 1 |
|  |  |  |  | hsa-miR-875-3p | 1 |
|  |  |  |  | hsa-miR-92a-1* | 1 |
|  |  |  |  | hsa-miR-29a | 1 |
|  |  |  |  | hsa-miR-124 | 1 |
|  |  |  |  | hsa-miR-582-3p | 1 |
|  |  |  |  | hsa-miR-486-3p | 1 |
|  |  |  |  | hsa-miR-891b | 1 |
|  |  |  |  | hsa-let-7c* | 1 |
|  |  |  |  | hsa-miR-624 | 1 |
|  |  |  |  | hsa-miR-1912 | 1 |
|  |  |  |  | hsa-miR-663b | 1 |
|  |  |  |  | hsa-miR-329 | 1 |
|  |  |  |  | hsa-miR-515-5p | 1 |
|  |  |  |  | hsa-miR-941 | 1 |
|  |  |  |  | hsa-miR-187* | 1 |
|  |  |  |  | hsa-miR-150 | 1 |
|  |  |  |  | hsa-miR-371-5p | 1 |
|  |  |  |  | hsa-miR-1224-5p | 1 |
|  |  |  |  | hsa-miR-1197 | 1 |
|  |  |  |  | hsa-miR-519a | 1 |
|  |  |  |  | hsa-miR-525-5p | 1 |
|  |  |  |  | hsa-miR-10b | 1 |
|  |  |  |  | hsa-miR-501-3p | 1 |
|  |  |  |  | hsa-miR-1202 | 1 |
|  |  |  |  | hsa-miR-623 | 1 |
|  |  |  |  | hsa-miR-135a | 1 |
|  |  |  |  | hsa-miR-29c* | 1 |
|  |  |  |  | hsa-miR-423-5p | 1 |
|  |  |  |  | hsa-miR-708 | 1 |
|  |  |  |  | hsa-miR-96 | 1 |
|  |  |  |  | hsa-let-7e | 1 |
|  |  |  |  | hsa-miR-92a-1* | 1 |
|  |  |  |  | hsa-miR-124 | 1 |
|  |  |  |  | hsa-miR-548a-3p | 1 |
|  |  |  |  | hsa-miR-1265 | 1 |
|  |  |  |  | hsa-miR-888 | 1 |
|  |  |  |  | hsa-miR-149* | 1 |
|  |  |  |  | hsa-miR-633 | 1 |
|  |  |  |  | hsa-miR-1914* | 1 |
|  |  |  |  | hsa-miR-1205 | 1 |
|  |  |  |  | hsa-miR-329 | 1 |
|  |  |  |  | hsa-miR-519e* | 1 |
|  |  |  |  | hsa-miR-941 | 1 |
|  |  |  |  | hsa-miR-196a* | 1 |
|  |  |  |  | hsa-miR-154 | 1 |
|  |  |  |  | hsa-miR-320b | 1 |
|  |  |  |  | hsa-miR-1470 | 1 |
|  |  |  |  | hsa-miR-556-5p | 1 |
|  |  |  |  | hsa-miR-499-3p | 1 |
|  |  |  |  | hsa-miR-181c* | 1 |
|  |  |  |  | hsa-miR-502-3p | 1 |
|  |  |  |  | hsa-miR-663b | 1 |
|  |  |  |  | hsa-miR-633 | 1 |
|  |  |  |  | hsa-miR-135a | 1 |
|  |  |  |  | hsa-miR-29c | 1 |
|  |  |  |  | hsa-miR-431 | 1 |
|  |  |  |  | hsa-miR-708* | 1 |
|  |  |  |  | hsa-miR-100* | 1 |
|  |  |  |  | hsa-miR-96 | 1 |
|  |  |  |  | hsa-miR-580 | 1 |
|  |  |  |  | hsa-miR-130a | 1 |
|  |  |  |  | hsa-miR-588 | 1 |
|  |  |  |  | hsa-miR-548o | 1 |
|  |  |  |  | hsa-miR-875-3p | 1 |
|  |  |  |  | hsa-miR-186 | 1 |
|  |  |  |  | hsa-miR-1208 | 1 |
|  |  |  |  | hsa-miR-556-5p | 1 |
|  |  |  |  | hsa-miR-452* | 1 |
|  |  |  |  | hsa-miR-515-5p | 1 |
|  |  |  |  | hsa-miR-941 | 1 |
|  |  |  |  | hsa-miR-373 | 1 |
|  |  |  |  | hsa-miR-320c | 1 |
|  |  |  |  | hsa-miR-1827 | 1 |
|  |  |  |  | hsa-let-7a | 1 |
|  |  |  |  | hsa-miR-220c | 1 |
|  |  |  |  | hsa-miR-196a* | 1 |
|  |  |  |  | hsa-miR-506 | 1 |
|  |  |  |  | hsa-miR-1205 | 1 |
|  |  |  |  | hsa-miR-143 | 1 |
|  |  |  |  | hsa-miR-362-3p | 1 |
|  |  |  |  | hsa-miR-452* | 1 |
|  |  |  |  | hsa-miR-301b | 1 |
|  |  |  |  | hsa-miR-29b | 1 |
|  |  |  |  | hsa-let-7f | 1 |
|  |  |  |  | hsa-miR-29b | 1 |
|  |  |  |  | hsa-miR-582-3p | 1 |
|  |  |  |  | hsa-miR-135a | 1 |
|  |  |  |  | hsa-miR-548a-3p | 1 |
|  |  |  |  | hsa-miR-708 | 1 |
|  |  |  |  | hsa-miR-186* | 1 |
|  |  |  |  | hsa-miR-638 | 1 |
|  |  |  |  | hsa-miR-1299 | 1 |
|  |  |  |  | hsa-miR-377* | 1 |
|  |  |  |  | hsa-miR-604 | 1 |
|  |  |  |  | hsa-miR-483-3p | 1 |
|  |  |  |  | hsa-miR-520a-5p | 1 |
|  |  |  |  | hsa-miR-941 | 1 |

**Supplementary Table S13:** Common miRNAs targeting AD and BARHL1-ESR1 pathway genes and their targets. The table also shows the regulation of targeting miRs in AD. Red genes are predicted targets.

|  | **Common validated miRNAs  in AD and BARHL1-ESR1 pathway** | **Targets from both the pathways (red are predicted by miRWalk)** | **Literature (PubMed) supporting  miRNA deregulation in AD** |
| --- | --- | --- | --- |
| 1 | hsa-let-7a-1 | BACE1, ESR1, BARHL1, NCSTN, APH1A | PMID: 22610069 (up)  [ let-7 family] |
| 2 | hsa-let-7a-2 | BACE1, ESR1 | NA |
| 3 | hsa-let-7a-3 | BACE1, ESR2 | NA |
| 4 | hsa-let-7b | ESR1, LRP1, LPL, BARHL1, NCSTN, APH1A | PMID:20936480 (dn) |
| 5 | hsa-mir-107 | ESR1,BDNF,BACE1, TH,NCSTN,IDE | PMID:20936480 (dn) |
| 6 | hsa-mir-122 | ESR1, ADAM10, APH1A,IDE | NA |
| 7 | hsa-mir-125b-1 | ESR1,APOE,CASP7, NTF3,NCSTN | PMID:17314675,PMID:17629564 (up) |
| 8 | hsa-mir-125b-2 | ESR1,APOE,CASP7, APH1A | PMID:17314675,PMID:17629564 (up) |
| 9 | hsa-mir-130a | ESR1,BDNF, TH,NTF3,APH1A | PMID:20126538 (dn) |
| 10 | hsa-mir-17 | ESR1,CACNA1C,IDE,APH1A | PMID:20936480 (dn) |
| 11 | hsa-mir-181a-1 | ESR1,BDNF,THRB,APOE,NTF3,IDE | PMID:20936480(dn) |
| 12 | hsa-mir-181a-2 | ESR1,BDNF,THRB,APOE,NTF3,IDE | PMID:20936480(dn) |
| 13 | hsa-mir-181b-1 | ESR1,ADAM10,NTF3,IDE | PMID:20936480 (dn) |
| 14 | hsa-mir-181b-2 | ESR1,ADAM10,NTF3,IDE | PMID:20936480 (dn) |
| **15** | **hsa-mir-18a** | **ESR1, BDNF, TH, BARHL1, NCSTN** | **PMID: 19936094, 24014289 (up)** |
| 16 | hsa-mir-19b-1 | ESR1,BACE1,NTF3,IDE | PMID:20936480 (dn) |
| 17 | hsa-mir-19b-2 | ESR1,BACE1,NTF3,IDE | PMID:20936480 (dn) |
| 18 | hsa-mir-21 | ESR1,THRB,CACNA1C,NTF3,APH1A,NCSTN | PMID:20936480 (dn) |
| 19 | hsa-mir-22 | ESR1,BDNF,BARHL1,IDE | PMID:20936480 (dn) |
| 20 | hsa-mir-221 | THRB,ADAM10,ESR1,BARHL1,NTF3,TH,IDE | PMID:20936480 (dn) |
| 21 | hsa-mir-222 | ADAM10,ESR1,APOE,NTF3,NCSTN,IDE | PMID:20936480 (dn) |
| 22 | hsa-mir-26b | ESR1,GSK3B,BARHL1,IDE | PMID:19110058 (dn) |
| 23 | hsa-mir-29b-1 | ESR1,BACE1,IDE,APH1A | PMID: 18525125 (up) |
| 24 | hsa-mir-29b-2 | ESR1,BACE1,IDE,APH1A | PMID: 18525125 (up) |
| **25** | **hsa-mir-30b** | **ESR1, BDNF, GAPDH, LRP1, SNCA, LPL, BARHL1, TH, NTF3,PSENEN** | **PMID:20936480 (dn)** |
| 26 | hsa-miR-30d | ESR1,GAPDH,LPL,SNCA,LRP1,NTF3 | PMID:20936480 (dn) |
| 27 | hsa-mir-30e | SNCA,LPL,LRP1,ESR1,NTF3 | PMID:20936480 (dn) |
| 28 | hsa-mir-1-2 | BDNF,GAPDH,LRP1,CACNA1C,LPL | PMID: 24022509 (up) |
| 29 | hsa-mir-1-1 | BDNF,GAPDH,LRP1,CACNA1C,LPL | NA |
| **30** | **hsa-mir-101-2** | **BDNF, CACNA1C, APP, NTF3, NCSTN, IDE** | **PMCID: PMC3547332 (dn)** |
| 31 | hsa-mir-101-1 | BDNF,APP,CACNA1C,NTF3,NCSTN,IDE | PMCID: PMC3547332 (dn) |
| 32 | hsa-mir-124-1 | BDNF,BACE1,NTF3,IDE,APH1A | PMID:17314675 (dn) |
| 33 | hsa-mir-124-2 | BDNF,BACE1,NTF3,IDE,APH1A | PMID:17314675 (dn) |
| 34 | hsa-mir-124-3 | BDNF,BACE1,NTF3,IDE,APH1A | PMID:17314675 (dn) |
| 35 | hsa-mir-132 | BDNF,GAPDH,NTF3, | PMID: 18525125 (up) |
| 36 | hsa-mir-15a | BDNF,BACE1,BARHL1,TH,NCSTN | PMID:18434550 (dn) |
| 37 | hsa-mir-195 | BDNF,BACE1,TH,NCSTN,IDE,APH1A | PMID:20936480 (dn) |
| 38 | hsa-mir-26a-1 | GSK3B,BDNF,IDE, | PMID:18525125 (up& dn) |
| 39 | hsa-mir-26a-2 | GSK3B,BDNF,IDE, | PMID:18525125 (up& dn) |
| 40 | hsa-mir-30a | BDNF,GAPDH,LRP1,SNCA,LPL,NTF3 | PMID:20936480 (dn) |
| 41 | hsa-mir-9-1 | BDNF,BACE1 | PMID:19406203 (up) |
| 42 | hsa-mir-9-2 | BDNF,BACE1 | PMID:19406203 (up) |
| 43 | hsa-mir-9-3 | BDNF,BACE1 | PMID:19406203 (up) |
| 44 | has-miR-320 | NTF3,NCSTN,APH1A | PMID:20936480 (dn) |

**Supplementary Table S14: Experimentally validated targets of miRNAs deregulated in AD. The miRNAs are selected from Supplementary table 10 and targets were identified using miRWalk.**

| **Targets of  downregulated miRNAs** | **Targets of  upregulated miRNAs** |
| --- | --- |
| NTRK3 | CYR61 |
| BCL2 | NPAS2 |
| NFYB | CDKN1A |
| RHD | COL1A1 |
| TOP2A | COL19A1 |
| SPRED2 | COL3A1 |
| SYNE1 | ENAM |
| KIT | COL4A1 |
| MITF | EZH2 |
| EPHB2 | COL4A5 |
| AKT1 | AMELX |
| LRRC4 | COL11A2 |
| ID1 | PRNP |
| SRC | CALB1 |
| BRCA1 | FOSB |
| NFYC | JUND |
| STX2 | GADD45B |
| KIF2A | DICER1 |
| PRSS8 | CD4 |
| TPM4 | MAX |
| MAPK14 | FASN |
| CDK6 | FAF1 |
| ME1 | CPLX2 |
| BACE1 | CPAMD8 |
| DVL2 | IL1B |
| HADHB | NEDD9 |
| FYCO1 | SPP1 |
| DEPDC1 | CD8A |
| DICER1 | IRAK1 |
| CDC42 | NFKB1 |
| ARPC1B | TRAF6 |
| PTBP1 | COL2A1 |
| AK2 | EGFR |
| ARF3 | TNF |
| PBK | ASPRV1 |
| C12orf23 | UNC5C |
| E2F5 | PTBP1 |
| BANF1 | DIAPH2 |
| SLC15A4 | HDAC9 |
| HLA-E | MYL9 |
| GBP3 | MECP2 |
| SLC22A5 | MAPK1 |
| HRSP12 | DCC |
| KIAA1598 | CCND1 |
| CD164 | TLR4 |
| RDH10 | IL1A |
| DNMT1 | NFKBIA |
| BDNF | PTBP2 |
| TUSC2 | FOS |
| GCA | PAK3 |
| RBMS1 | IL17A |
| GLTP | MAPK8 |
| PLOD3 | EPHA3 |
| CHODL | BRAF |
| TGFBR2 | PDGFD |
| ATP8A2 | SAMD3 |
| TWIST2 | ANG |
| LUC7L2 | FOSL2 |
| QSER1 | IL10 |
| ELOVL5 | CYCS |
| G3BP1 | MAP2K1 |
| PRKD1 | GYPE |
| FAM81A | MYC |
| GCLC | CCL5 |
| RBM47 | IL6 |
| CDCA7 | SMAD3 |
| CDKN2D | MMP9 |
| GEMIN4 | JUN |
| MXD4 | HSH2D |
| RPIA | IGF1R |
| NME4 | TGFBR2 |
| NETO2 | MET |
| TNRC6B | SMAD7 |
| TSC22D3 | MMP16 |
| ADAR | RELA |
| PTPRC | MMP13 |
| OAF | AHSA1 |
| SMAD5 | PRB1 |
| HIST1H3B | NELL1 |
| ITGB1 | RET |
| CCL2 | RAB22A |
| CAP1 | TP53 |
| CREB1 | BEST1 |
| CHSY1 | TIMP3 |
| CNOT6 | BRMS1 |
| VAMP3 | SEC14L2 |
| SCPEP1 | CD68 |
| VIM | TLR2 |
| ANKRD29 | MAS1 |
| EML4 | MYB |
| EPB41L4B | SOCS1 |
| FABP4 | IRAK4 |
| TMBIM1 | MAPK3 |
| ARHGEF1 | CD79A |
| RHOG | PTCH1 |
| FOXO1 | BRCA1 |
| UHMK1 | DMD |
| TRAPPC3 | TNFSF10 |
| PLEKHG2 | FAS |
| SHC3 | RARA |
| CD83 | RHOC |
| TJP2 | BRCA2 |
| FAM104A | FGF2 |
| SH2D3C | SLC7A5 |
| LARP4 | IRAK2 |
| CYP1A1 | NR1I2 |
| RNF149 | ROBO1 |
| TFAP4 | POLR1A |
| C1orf56 | PLAU |
| CD59 | IL8 |
| CEBPA | BCL2 |
| DDX20 | TCEAL1 |
| EFNB1 | RUNX1 |
| DFFB | NBN |
| GSN | EWSR1 |
| LRRC1 | KLK10 |
| STX10 | SOAT1 |
| PTBP2 | STAT1 |
| ZHX1 | API5 |
| XPO5 | IL2RA |
| MKRN1 | IL7R |
| CYB5R4 | CREB1 |
| RYK | HTR3A |
| ZBED3 | SPDEF |
| FOXM1 | ERBB4 |
| ACTL6B | AKT1 |
| NEK6 | AKT2 |
| XPO6 | VEGFA |
| CHST11 | STMN1 |
| CAV1 | PI3 |
| DPF1 | MYCN |
| BAZ1A | TNFAIP2 |
| MMD | GEMIN4 |
| ABHD5 | CD19 |
| TLN1 | PIK3CA |
| NUPL1 | RNPC3 |
| STMN1 | IL2 |
| RHOV | CA3 |
| LIN7C | NFATC2 |
| NPPA | INPPL1 |
| STK4 | BIN3 |
| CDK4 | DCT |
| EYA4 | NFYC |
| LAMC1 | DSPP |
| CLDND1 | BMP5 |
| PTTG1IP | KRT10 |
| JAK2 | PNCK |
| CCND1 | BAD |
| RRBP1 | COX8A |
| FBXW7 | DUSP9 |
| PHC2 | DDX20 |
| HADH | EPHB2 |
| C12orf49 | TMEM132D |
| NECAP2 | PDGFA |
| RAVER2 | BMP4 |
| SLC1A3 | CYP3A4 |
| CTDSP2 | VDR |
| STOM | BACE1 |
| ARF4 | DPT |
| LRRC8A | TBC1D9 |
| PRC1 | SYNE1 |
| ERH | MCL1 |
| TTC8 | CDC42 |
| GCH1 | NOS3 |
| PGM1 | SH2B2 |
| SNAI2 | RAG2 |
| PTPN12 | BIRC3 |
| IQGAP1 | ACTA2 |
| IMPDH1 | ZNRD1 |
| DCX | STMN2 |
| OSBPL7 | PTEN |
| USP49 | BMP2 |
| RELA | MYOD1 |
| ATP6V0E1 | HDGF |
| JAKMIP1 | RUNX2 |
| ABCE1 | TUBE1 |
| CLCN3 | ATP2A2 |
| GBAS | CDKN2A |
| C9orf78 | MAP2K3 |
| FAM57A | PITX2 |
| SURF4 | SRGAP2 |
| NPAT | TCF3 |
| PLSCR3 | KRAS |
| NR3C2 | TOP2B |
| CMTM4 | ZEB1 |
| APEX2 | ROBO2 |
| MTMR12 | CCND2 |
| TAGLN2 | CD28 |
| CTDSP1 | ZEB2 |
| DGCR8 | EGFL7 |
| POLR3G | KLF4 |
| ENDOD1 | JAG1 |
| PREX1 | IKBKB |
| HIST1H3A | FUT4 |
| FMR1 | SOX9 |
| NR3C1 | PAK1 |
| PGRMC2 | ITGB1 |
| FOXG1 | NRAS |
| SLC16A9 | CTNNB1 |
| MAN2A1 | TWIST1 |
| RASSF5 | MOCOS |
| RNPEPL1 | NEUROG3 |
| REST | LBR |
| HTATIP2 | APC |
| INPP5F | TIMM8A |
| NFIC | CD44 |
| AHRR | MYH11 |
| MAPK1IP1L | FSTL1 |
| CAPRIN2 | ACVR1B |
| SLC30A7 | PTPN1 |
| GNAI3 | MAPK14 |
| LZTFL1 | TAF1 |
| SOX9 | ESR1 |
| DNAJC1 | DNMT1 |
| SYNGR2 | GJA1 |
| MYO10 | RAPGEF5 |
| SPHK1 | GRIA2 |
| SERP1 | ELF4 |
| SLC16A1 | MYLIP |
| PLP2 | TUSC2 |
| TRIP11 | NOTCH1 |
| SYPL1 | CCL7 |
| ZNF264 | FUS |
| PDLIM7 | ARID4B |
| RAB40B | DYRK2 |
| CNN3 | TAC1 |
| CYP1B1 | SOCS3 |
| SH3PXD2B | HIPK3 |
| TIMP3 | UTRN |
| HERPUD1 | BIRC7 |
| EBP | ATF2 |
| EPHA1 | VSNL1 |
| ZNF384 | YWHAZ |
| GPSM2 | S100A8 |
| PGM2 | ITPR1 |
| EXOSC1 | MEIS1 |
| TARBP2 | DECR1 |
| TRIM29 | GATA1 |
| HSPA14 | S100A9 |
| SDC4 | ZFP36 |
| PCGF5 | NIT1 |
| FOXA2 | BCR |
| PPP1R13L | MLLT3 |
| TRIM2 | PAH |
| ARHGEF3 | ROS1 |
| STX11 | SSSCA1 |
| ACTL6A | BBS9 |
| KLK8 | ADM |
| HEBP2 | MED1 |
| INSIG2 | HEMGN |
| TSPAN4 | ABCB1 |
| RABGAP1L | MAPK9 |
| DHCR24 | ATP8A2 |
| MDFIC | TAL1 |
| TBC1D2 | CAB39 |
| CD28 | CHD4 |
| ELSPBP1 | PRKAA1 |
| MPHOSPH9 | WT1 |
| LASP1 | FBXW7 |
| TMEM9B | AP2M1 |
| ZNF226 | STK11 |
| C1QBP | RASA1 |
| TOM1L1 | PTGS2 |
| AHR | CD33 |
| TDP1 | FOXO3 |
| PAPSS2 | PROM1 |
| TSPAN15 | DDX5 |
| ANKRD13B | RAB14 |
| SLC17A5 | EPOR |
| SERPINB5 | TAF10 |
| SP1 | GLYAT |
| CPNE3 | IFNA1 |
| GAS2L1 | GATA2 |
| GFAP | EPHA2 |
| TMEM132D | FGFR1 |
| OSBPL8 | CGA |
| TSKU | FLT3 |
| RNH1 | ARPC5L |
| RNF138 | HMBS |
| SMYD3 | NCOA6 |
| MED28 | GATA3 |
| MAGT1 | PIAS1 |
| ANKRD27 | AMH |
| CENPF | LITAF |
| SWAP70 | SCN5A |
| OAT | ANK1 |
| POGK | SYNGAP1 |
| HIST1H3C | RSL1D1 |
| MOV10 | RAB8A |
| ACAA2 | MIF |
| MMP1 | HOXB3 |
| TTC7A | MBD4 |
| SOX2 | JAK2 |
| ACTR8 | HBB |
| ZFP36L2 | MAP3K8 |
| HUWE1 | RB1 |
| CTGF | POLD3 |
| ANKRD52 | PTGS1 |
| CDKN1A | ALG2 |
| CREB3L2 | CYP26A1 |
| KIAA1919 | PLAG1 |
| NFATC1 | GABPA |
| C16orf82 | DGCR8 |
| GNPDA2 | APOE |
| ARFIP1 | ENPP6 |
| PODXL | GRM7 |
| TARBP1 | ELOVL7 |
| KIF23 | SNAI2 |
| ARRDC1 | ATXN1 |
| RASSF1 | CDK4 |
| DCTD | CLCN3 |
| FAM83H | LATS2 |
| PLEKHB2 | BCL6 |
| OCA2 | KDR |
| ADAM9 | WEE1 |
| SYCP1 | CBFB |
| CHST14 | E2F3 |
| PIWIL1 | LGALS3 |
| SLC25A30 | RXRA |
| NIN | SIAH1 |
| SH2D4A | ITGAL |
| TUBB6 | CDC14A |
| PDX1 | HOXA4 |
| PHF10 | NDUFA2 |
| BLCAP | MUC1 |
| ARHGEF18 | GTF2IRD1 |
| F11R | ITGAM |
| TNFAIP1 | CCNE1 |
| MTPN | PDXP |
| RNPC3 | NFE2L1 |
| DNM2 | CDK6 |
| H3F3B | CDC25A |
| CDCA7L | CHEK1 |
| FBLN2 | CCNF |
| LMNB1 | AADAC |
| PSG1 | NFE2L2 |
| IFRD2 | ETS1 |
| USP1 | CCNE2 |
| RHOD | IBSP |
| AP1M2 | SP3 |
| GATA1 | SPINK1 |
| DAND5 | ATF6 |
| ARAF | BMPER |
| AXL | KIF23 |
| NCAM1 | USF2 |
| MTMR6 | CCND3 |
| LOC285908 | E2F1 |
| B4GALT1 | YY1 |
| SERPINB6 | SETD2 |
| SSFA2 | CAMTA1 |
| RARG | CEBPB |
| HIST1H3F | HIF1A |
| RFFL | ANLN |
| S100B | KCNH8 |
| FCHO2 | NUDT6 |
| LDLRAP1 | CUL2 |
| TSC22D4 | POU2F2 |
| SUCLG2 | CXCL12 |
| POLR2K | TGFB3 |
| HES1 | PLOD3 |
| USP48 | CEBPZ |
| CFL2 | TGFB1 |
| KANK1 | CDC34 |
| OCRL | HMGA2 |
| HIST1H3I | TUT1 |
| NLRP1 | RPIA |
| ALDH9A1 | IGF2BP1 |
| MYBL1 | IFI44 |
| EZH2 | ATN1 |
| ARHGAP29 | HNRNPA1 |
| FA2H | PARP8 |
| CTNND1 | GPI |
| PARP16 | SCPEP1 |
| CDKN1B | DCN |
| ELOVL1 | SOX2 |
| NEK9 | TRIM32 |
| NID1 | DNMT3A |
| SRXN1 | UHRF2 |
| TNFRSF21 | SMO |
| LITAF | NPC1 |
| ELK3 | SRC |
| DDX5 | PRDM1 |
| TMEM14A | MMP14 |
| VPS37C | ZNF362 |
| SLC2A1 | ACVR2A |
| BIN3 | FBXW11 |
| TNS4 | CYFIP2 |
| KCNJ8 | LIF |
| BTG3 | DDIT3 |
| SLC44A1 | TNRC6A |
| LOC51035 | IL13 |
| TMEM109 | ZCCHC6 |
| ADIPOR2 | CD46 |
| RAC1 | PSMA7 |
| UST | FN1 |
| ELF4 | LIN28B |
| ARCN1 | AMACR |
| CAND1 | ARC |
| NEUROD1 | GADD45A |
| KLHL12 | AKT1S1 |
| HPS4 | PPARGC1A |
| SENP8 | CD40LG |
| JAG1 | CSAD |
| PHF19 | HDAC4 |
| ANKIB1 | TMEM184B |
| SETD2 | NPAT |
| HIST1H3G | TNRC6B |
| BBS9 | STAT3 |
| MAD2L2 | NPM1 |
| RAB11FIP2 | HMOX1 |
| MYH9 | DDX6 |
| ISY1 | NES |
| ROCK2 | KIT |
| FAM129B | ZCCHC11 |
| ACPL2 | GRB2 |
| PGF | CHAF1A |
| STAT3 | SMC1A |
| PDCD4 | ERBB2 |
| TEAD1 | CKAP4 |
| VPS26A | HOXA10 |
| TNFSF10 | RDX |
| ATP6V1B2 | NUMA1 |
| DHX15 | TP63 |
| GTPBP8 | EIF4G1 |
| PEA15 | CDKN2D |
| CDC14B | EPB41L3 |
| SMARCA1 | NCBP1 |
| KATNA1 | ITGB3 |
| SEC11A | CCNA2 |
| IFT52 | BASP1 |
| TWIST1 | GPD1 |
| MAP3K12 | XPO1 |
| SOX4 | SLC25A10 |
| ABCG2 | TRAT1 |
| AADAC | HMGA1 |
| CAMTA1 | LARP4 |
| CD38 | GAB2 |
| IBSP | CCR4 |
| SMAD6 | KHSRP |
| GALNT1 | STK24 |
| HDAC9 | ABCG4 |
| MLH1 | E2F2 |
| NOTCH1 | FOXP1 |
| SRY | UBE2I |
| TP53 | ECD |
| POLD3 | DNMT3B |
| TNF | PDHA2 |
| VCAN | CNN3 |
| PCK2 | TMOD3 |
| FOXO3 | TCF19 |
| MAPK7 | SPRY2 |
| MMP9 | KISS1R |
| ERBB4 | NRIP1 |
| MYO6 | HK1 |
| APC | RPE |
| COX8A | COPS8 |
| KLF4 | TRIM17 |
| FASLG | CASP3 |
| LEF1 | CCNT2 |
| MYH11 | CBS |
| PPARG | RBBP6 |
| ACE | TMSB10 |
| KRT14 | TREH |
| MYCN | TNRC6C |
| SLC2A2 | CYLD |
| SPI1 | CNTN3 |
| ACTA2 | LTBP1 |
| CLU | TM9SF3 |
| UCP1 | LAMC2 |
| LCT | ALDH1A1 |
| ANG | CXCR4 |
| KLF5 | SRM |
| LIF | CSN1S1 |
| FNDC3B | PGC |
| ITGAM | CASP8 |
| SMTN | MBL2 |
| NTS | CEBPA |
| DNMT3A | SLC27A4 |
| ACE2 | CLU |
| IRAK2 | TGFBR1 |
| NPC1 | NOL3 |
| MAP3K7 | DOK4 |
| KCTD11 | ELSPBP1 |
| FSCN1 | POLR2A |
| TAGLN | RTN4 |
| KIR3DL1 | ELK3 |
| ADD3 | TPPP3 |
| SRF | PSD4 |
| FOS | LSM1 |
| KRAS | CASP9 |
| MAEA | GLI1 |
| KCNH8 | DMTF1 |
| TNNI3 | EIF3A |
| PIK3CA | IGF1 |
| JUN | NKX2-1 |
| CKAP4 | TP73 |
| NFKB1 | COL4A2 |
| PTEN | ESR2 |
| RREB1 | NCALD |
| PSTPIP1 | TBX5 |
| NKX2-5 | CLEC5A |
| MAPK3 | PGRMC1 |
| VEGFA | NCAPG |
| PDCD5 | ATP6V1B2 |
| NKX2-3 | STK3 |
| HNF4A | MMP8 |
| RBBP6 | AIRE |
| RABEP2 | PAX7 |
| MYC | IFNA2 |
| LPIN1 | MSTN |
| FOSL1 | CSF1 |
| SOX18 | NCOA4 |
| ERCC8 | CISH |
| HOOK2 | ERBB3 |
| RAB27A | DUSP2 |
| HMGA2 | WFS1 |
| TP73 | PRTN3 |
| PYCR2 | BCL2L1 |
| PTN | XRN1 |
| FASN | KITLG |
| BEX1 | CACNA2D2 |
| MMP3 | SLC7A1 |
| ERBB3 | EIF4E |
| MTDH | HNF4A |
| GYPA | ZNF512B |
| TFRC | KLF5 |
| CD36 | CCNG1 |
| LATS2 | PDCD6 |
| PPP2R2A | WNT1 |
| PPP2R4 | IFNG |
| RB1 | GHR |
| MSTN | OPRM1 |
| RTF1 | TAF1C |
| EGFL7 | RORA |
| PDGFRA | CYP27B1 |
| MTG1 | SOCS4 |
| RHOC | COL5A3 |
| GNAI2 | DERL1 |
| CLNS1A | SKP1 |
| EGF | MTSS1 |
| PITX2 | HAND1 |
| RB1CC1 | RBPJL |
| PAF1 | SMARCA5 |
| CCKBR | ACTG1 |
| SOX6 | RABEPK |
| EIF4EBP1 | LAMP3 |
| NUAK1 | EGR1 |
| MET | TARBP2 |
| ZEB2 | SMOX |
| SLC45A3 | LRRK2 |
| LYPLA1 | FOXA1 |
| RET | FMR1 |
| SLC20A1 | CTNNBIP1 |
| LIMK1 | WIBG |
| BRAF | SHH |
| EID1 | FXR1 |
| EGFR | NCOA3 |
| HTR2C | MEST |
| PCSK9 | RASSF1 |
| POMC | EXOSC1 |
| FAS | HAND2 |
| FOXJ3 | MOV10 |
| RGS2 | CSF1R |
| RHAG | CDC25C |
| ANGPTL6 | IGF2BP3 |
| PI3 | IGF2R |
| DNAI1 | AFP |
| CXCR4 | SLC17A5 |
| PROM1 | MESDC1 |
| RELN | MYH6 |
| ADORA2B | MYH7 |
| BAX | TNNI3 |
| CLTC | MRGPRX3 |
| TGFB1 | MED13 |
| IFNA1 | GATA4 |
| ROS1 | COL1A2 |
| FLT3 | MDM2 |
| TGFBR1 | CTGF |
| SNAP25 | ALCAM |
| AFF1 | SLC9A3R2 |
| H2AFX | DTL |
| PAK3 | TYMS |
| R3HDM1 | GRIN2B |
| APOBEC3C | FOXP2 |
| NR1I2 | GAPDH |
| HGF | BDNF |
| NF2 | PLEKHM1 |
| PDGFA | CBX5 |
| MAS1 | CTLA4 |
| MDM4 | SLC25A5 |
| ABCC1 | RELB |
| MSH3 | EP300 |
| ABCC2 | REST |
| CDKN2A | PPARG |
| PECAM1 | SIRT1 |
| SPP1 | PHGDH |
| CSH1 | HRSP12 |
| ERBB2 | ARNTL |
| MAFG | GRM3 |
| COL1A1 | GRIN2A |
| BIRC5 | PDGFRA |
| ROBO1 | RPS6KA5 |
| UBASH3A | GRIA1 |
| IKBKB | PER1 |
| SOCS1 | PAIP2 |
| MBNL2 | MYD88 |
| NFE2L1 | HBEGF |
| PXN | ANKS1B |
| LAMB3 | RAC1 |
| SLIT1 | CLOCK |
| CD19 | RFX4 |
| BAZ2A | ACE |
| PDXP | BTG2 |
| PAM | PECAM1 |
| SLIT2 | DDT |
| SH3D19 | TLR5 |
| ZNF135 | CCL2 |
| IKBKE | BRAP |
| EFNA1 | MITF |
| THAP2 | ARIH2 |
| ROBO2 | TRPM1 |
| GLCE | KCNJ13 |
| MRPS27 | NFAT5 |
| SMARCA5 | BBC3 |
| SPRED1 | DKK2 |
| GLB1 | TBK1 |
| HPS1 | MYBL1 |
| HLA-G | ANGPT1 |
| PIK3R2 | DDIT4 |
| NUP93 | SLC1A1 |
| CRK | POU3F2 |
| GJA1 | CREBZF |
| SOAT1 | PRAME |
| IGFBP5 | NANS |
| RIT2 | BNIP3L |
| IGFBP6 | KCNMA1 |
| CISH | SMAD2 |
| BRCA2 | NOTCH2 |
| TBP | ZNF135 |
| AATF | AXL |
| INS | POU4F2 |
| E2F1 | TOM1 |
| PAWR | HIST1H2AE |
| KPNA2 | SLC12A1 |
| BAP1 | MDM4 |
| KCNMA1 | MAP1LC3A |
| MRE11A | ZAP70 |
| MYBL2 | IRS1 |
| LOX | CDC20B |
| HOXB8 | NDRG1 |
| ATF1 | WNT3 |
| CDH17 | MAPKAPK2 |
| SEMA6A | TCF21 |
| NBN | FOXR1 |
| PSAT1 | CD3EAP |
| PTPN13 | OCA2 |
| ATM | SND1 |
| HIF1A | MGST1 |
| SFRP1 | AXIN2 |
| PCNA | CFD |
| HPRT1 | SLC2A1 |
| SLC12A1 | BANP |
| CTSD | RGS3 |
| KLK3 | CAV1 |
| TCF21 | DDX4 |
| IGF1R | PPP1R13L |
| PPIA | HES1 |
| FBN1 | DLL1 |
| ALOX5 | FOXO1 |
| DLK1 | SEC24D |
| HOXA1 | VAMP2 |
| ALOX5AP | HELLS |
| ITGA2B | PEA15 |
| MYEF2 | PAWR |
| MTSS1 | ANP32B |
| MAFB | RNMT |
| BMP2 | RTEL1 |
| RUNX2 | SKP2 |
| SLC24A4 | UBL4A |
| DBP | TESC |
| SLC27A4 | PRMT5 |
| ATP2B1 | ACTB |
| SIRT1 | BAMBI |
| SLC8A1 | EGR2 |
| PROC | NANOS2 |
| SMAD3 | MAP3K9 |
| MAPRE2 | ING2 |
| COL1A2 | RPS19 |
| BCL2L1 | ACSL1 |
| SMAD9 | HDAC2 |
| COL3A1 | CRIM1 |
| DMPK | G6PD |
| IMPACT | TTF2 |
| TLR4 | PTPN12 |
| ARSF | E2F5 |
| FOXC1 | LOX |
| QKI | BAX |
| PAK1 | PRAP1 |
| MCM2 | ARCN1 |
| DAP | PDLIM7 |
| VSNL1 | REL |
| CBX5 | CCNB1 |
| MBL2 | ST3GAL4 |
| PIK3R3 | SLC11A1 |
| CDKN1C | STK39 |
| IRS2 | SEMA6A |
| IRS1 | LEF1 |
| BCL2L11 | LRP6 |
| SSSCA1 | NR1H4 |
| TLR9 | FOXF1 |
| TNK2 | HOXA5 |
| SLC11A1 | AURKB |
| PTGS2 | CYP2A6 |
| ARC | PTPN13 |
| MOCOS | TCL1A |
| MAEL | SPI1 |
| SIRT2 | FOXE1 |
| CXCL12 | ZNF77 |
| PIK3CD | PPIG |
| CCT4 | FOXJ1 |
| FUT4 | HGF |
| PAX6 | MDC1 |
| TIAL1 | CD47 |
| SNCA | SFRP1 |
| ERF | FOXM1 |
| SLC3A2 | XAF1 |
| E2F3 | MTUS1 |
| LRRK2 | FOXP3 |
| IGF1 | FGFR1OP |
| HOXD10 | RNASEH2A |
| RAF1 | PRKCH |
| TIMP2 | GLI2 |
| ETS2 | ZFPM2 |
| HELLS | FZR1 |
| ERCC4 | IGF2BP2 |
| FGFR3 | TNFSF11 |
| CCNE2 | GLI3 |
| BAD | SUFU |
| CEBPZ | KLF8 |
| CYP2A6 | CDH1 |
| PDGFB | NRK |
| PAH | INHBE |
| TNC | G3BP2 |
| PLAC1 | FOXA2 |
| KDR | POU3F1 |
| SMO | TWIST2 |
| IL3 | WASF3 |
| PPIG | MMAB |
| NPM1 | ETS2 |
| CD4 | SNAI3 |
| CRLS1 | CDKN1B |
| KITLG | CSNK1A1 |
| MYB | ATM |
| MRGPRX3 | RAB30 |
| CD70 | TFRC |
| TP63 | IHH |
| PLK2 | SULT2A1 |
| STAT5A | THBS1 |
| CASP3 | PDGFB |
| MERTK | JUNB |
| CD44 | INHBC |
| ACVR2A | EGF |
| ENAH | FOXL1 |
| FOXJ2 | SOX13 |
| RUNX1 | JAG2 |
| PTPRN2 | PLCG1 |
| IK | FOXC2 |
| HOXA9 | PCDH8 |
| CD8A | MARCKS |
| RASA1 | MAP3K10 |
| RUNX1T1 | SNAI1 |
| AHSA1 | RASGRP3 |
| BRD8 | PDGFRB |
| POLR2A | PTPN14 |
| TOM1 | DHH |
| NOS3 | PSAT1 |
| KLF2 | MED6 |
| GLI1 | TBX2 |
| IDUA | GPR160 |
| HMOX1 | PSMD2 |
| ETS1 | CDON |
| GATA3 | BCL2L11 |
| IL1B | CD69 |
| NRAS | KLK2 |
| MAPK8 | KLK1 |
| CD2AP | KLK12 |
| THBS1 | CYP19A1 |
| RGS3 | FABP4 |
| VCAM1 | AR |
| TLR2 | LARP6 |
| SMOX | KLK4 |
| WT1 | ADIPOQ |
| FLI1 | KLK8 |
| ITGAL | HIST1H2BB |
| CTSC | IL23A |
| CSF1 | KLK5 |
| FOSB | HSF1 |
| SYNGAP1 | KLK13 |
| INPPL1 | SYCP3 |
| MEST | SELL |
| MBD4 | KLK3 |
| ITPR1 | SMAD1 |
| NIT1 | DNAH8 |
| LRG1 | ME1 |
| MED1 | RRBP1 |
| MAPK9 | MARK2 |
| CHD4 | KLF2 |
| MLC1 | GARS |
| MAP2K1 | HUWE1 |
| AP2M1 | CPEB2 |
| ESR1 | PIWIL1 |
| TAF10 | CDC6 |
| ID4 | CPEB4 |
| GULP1 | CHFR |
| FGFR1 | MTDH |
| MAPK1 | XPO5 |
| ARPC5L | SOX6 |
| CCPG1 | TAF12 |
| NCOA6 | COMP |
| ROCK1 | FH |
| PIAS1 | PTP4A1 |
| MSC | GHRHR |
| AKR1B1 | NPHS2 |
| ESRRG | CD40 |
| NPNT | CHRD |
| IFNGR1 | METAP2 |
| SERPINE1 | PPFIBP2 |
| B3GALNT1 | ALPL |
| GALNT7 | NOG |
| GFPT2 | CIB1 |
| IL1A | DES |
| SUFU | MAP2 |
| CYP2E1 | GSK3B |
| TOB1 | MAP3K12 |
| ODC1 | CDK8 |
| DECR1 | CTDSP2 |
| TOB2 | TAF9 |
| PGR | SH3BP4 |
| BTG2 | R3HDM1 |
| FCGR3B | MAP3K2 |
| PARP1 | MYT1 |
| AGER | PPARA |
| SULT2A1 | ZBTB10 |
| MYOD1 | HLA-E |
| WDTC1 | PDX1 |
| CD69 | POLD1 |
| PPARGC1B | CSF3 |
| PTK2 | HIPK2 |
| XBP1 | SP4 |
| NR0B2 | C9orf3 |
| WDR20 | CCT4 |
| NTRK1 | PCNA |
| BCL6 | DPYD |
| PRDM1 | CTSD |
| PBRM1 | MEF2C |
| CDH18 | POLR2K |
| GREM1 | RYBP |
| BCR | IGFBP5 |
| AFP | FADD |
| EPHB6 | PAX3 |
| ZNF513 | PKMYT1 |
| PRPS1L1 | IGFBP6 |
| SNX19 | AIFM1 |
| PRPS1 | ACE2 |
| TTK | ADORA2B |
| ACVR1C | LRPAP1 |
| LAMA1 | HOXD13 |
| CHRD | INS |
| NOG | BRD2 |
| CYP7A1 | KPNA2 |
| IFNG | TRIB1 |
| PPM1A | BAP1 |
| PKN1 | AGTR2 |
| SOD1 | CYP1B1 |
| SOD2 | MRE11A |
| COMP | PHB |
| GHRHR | ATF1 |
| GEMIN5 | PBK |
| ARIH2 | GEMIN5 |
| TRIM31 | LRRC4 |
| FES | ID1 |
| REL | CYP7A1 |
| CORO1A | CYP8B1 |
| CDC14A | TPT1 |
| RECK | CAB39L |
| SAA4 | DNAJB7 |
| AKT3 | CREBBP |
| SKP2 | NOTCH4 |
| BBC3 | ST14 |
| ADAM10 | G3BP1 |
| TAF9 | BID |
| CCNE1 | ITGA6 |
| JAK1 | OAT |
| CHEK1 | TBX1 |
| CCNF | CDH5 |
| HIST1H2AE | LHFP |
| AGTR1 | RHD |
| SDC1 | NTRK2 |
| SLAIN1 | MCAM |
| CIB1 | CDH17 |
| HMGA1 | BIN1 |
| ICAM1 | TFE3 |
| PUM1 | HNF1A |
| HIST1H2BB | RND3 |
| DKK2 | USF1 |
| JAK3 | QKI |
| ATF6 | PDPN |
| DPT | BRD7 |
| STAT1 | MAF |
| HSF1 | TGFB2 |
| TBK1 | NQO1 |
| FOXP2 | HPRT1 |
| STYK1 | FBLN5 |
| GPI | CHST3 |
| CDK2 | TCF7L2 |
| ADAM17 | CXCL3 |
| DCTN6 | FHOD1 |
| CDC25A | MBNL2 |
| STAT2 | SLC22A3 |
| BMX | RAD23B |
| HSD11B1 | MKL1 |
| NGF | PPIA |
| DDIT4 | KLF13 |
| SYK | SRF |
| TMEM11 | RIT2 |
| ZEB1 | PPP2R1B |
| APOE | TIMP2 |
| ANLN | TBP |
| NFIB | TRPS1 |
| PSMD9 | ABCC1 |
| CREBZF | PDCD4 |
| BTK | PPM1F |
| POU2F2 | TUBB3 |
| RTN4 | HOXD10 |
| ZBTB16 | ROCK1 |
| ATR | NRGN |
| PDC | CD83 |
| BNIP3L | DST |
| CDC73 | F8 |
| CSK | SMAD4 |
| EGR2 | CCR5 |
| TTF1 | STAT6 |
| HNF1A | KCNG1 |
| BRAP | ZBTB16 |
| EPOR | CD80 |
| TYK2 | RPS14 |
| WEE1 | HLA-A |
| TPM1 | RC3H1 |
| ATP6V0A1 | CCL8 |
| IRAK1 | SOCS2 |
| MECP2 | MMP2 |
| PUM2 | CD86 |
| PTP4A3 | L1CAM |
| COL11A2 | DDIT4L |
| PROCR | TRPV1 |
| FLOT2 | TSPO |
| MTUS1 | CCDC6 |
| SST | NOX4 |
| RNASEH2A | CFH |
| RARA | TBCE |
| IGF2BP2 | CXCL9 |
| TNFSF11 | ADAM8 |
| ARL2 | IL17D |
| KRT7 | TLR9 |
| G3BP2 | CAP1 |
| GRN | CRYGC |
| CDC6 | HERC5 |
| CHST3 | GSS |
| SLC26A3 | TBX21 |
| EGR3 | B3GALNT1 |
| RAB30 | BANK1 |
| SCD | TSPAN12 |
| EGR1 | SERPINE1 |
| JUNB | PA2G4 |
| CCL4 | MPI |
| BCL2L2 | DDX58 |
| FGF1 | CXCL11 |
| TNRC6A | TMEM11 |
| SPINK1 | HOXB8 |
| CCND3 | NLRP3 |
| PCDH8 | KRT12 |
| DOK4 | CHST14 |
| IL6 | TNPO1 |
| PDGFRB | ELOVL6 |
| ALPL | CTSG |
| NPY | CCR7 |
| MED6 | DSG2 |
| EDN1 | GYS1 |
| PSMD2 | TAF9B |
| NEUROG3 | WNT5A |
| NRIP1 | YIF1B |
| GARS | IQGAP3 |
| AP2A1 | HMBOX1 |
| SLC25A24 | ARFIP1 |
| ANKFY1 | SCYL1 |
| SLC1A4 | SLC12A2 |
| GFM1 | TMED10 |
| TMED2 | TRIM27 |
| ZAP70 | IFIT5 |
| SH3BP4 | TACSTD2 |
| LPL | ABHD10 |
| PPP3CA | BCKDHB |
| GEMIN7 | ABHD11 |
| TRIP13 | SYNE2 |
| WNT5A | FAM105A |
| SNX6 | RNF213 |
| COIL | AGMAT |
| MPDU1 | SNAP29 |
| ARL5B | SLC6A3 |
| IDH1 | EHMT2 |
| PLK1 | POLA2 |
| TNFAIP2 | FADS1 |
| NRP1 | PPP1R7 |
| LYPLA2 | RAB27B |
| TMEM87A | CAPG |
| MYO1E | SFI1 |
| CCL13 | CDIPT |
| UTP15 | FGF13 |
| SLC12A2 | AMIGO2 |
| PDLIM5 | POLE3 |
| SLC6A3 | DHX40 |
| TMEM189 | GPR56 |
| LY6K | MOSPD2 |
| PRKCI | SAC3D1 |
| CACNA2D1 | NT5C3A |
| SLC16A3 | PURA |
| SMAD2 | TPM2 |
| PTRH1 | AP3D1 |
| GPD2 | PTPMT1 |
| NT5E | RAI14 |
| ZIC1 | GNPNAT1 |
| PTPLAD1 | LMNB2 |
| SLC7A1 | SYPL1 |
| SSNA1 | TNFRSF10B |
| MLLT11 | TXN2 |
| PISD | GNA13 |
| LRP1 | GLIS2 |
| PPP3R1 | HARS2 |
| WDFY1 | KRT85 |
| EHMT1 | CDKAL1 |
| RAB6A | AARSD1 |
| UNC93B1 | SLC4A7 |
| TMEM59 | HSPA1B |
| BET1 | SERPINE2 |
| FGF2 | LRRC8A |
| IFRD1 | IFRD2 |
| SQSTM1 | RFT1 |
| ARID4B | CPNE8 |
| FMNL2 | SPRYD4 |
| SDCBP | PAFAH1B2 |
| STX7 | SNCA |
| IGF2BP1 | DSP |
| TMED3 | ANXA2 |
| RHOB | MRPS33 |
| PPIB | CA12 |
| PKN2 | ASH2L |
| PXDN | DNM1L |
| RQCD1 | DNAJC19 |
| KDELC2 | CHMP2A |
| TTC9C | SLC12A4 |
| NUCB1 | FERMT2 |
| SNAP29 | NAT6 |
| DSG2 | PPP5C |
| GYS1 | RCN2 |
| TAF9B | HSDL1 |
| TMED10 | VPS39 |
| IFIT5 | AGRN |
| TACSTD2 | TMCO1 |
| ABHD10 | GPAM |
| RNF213 | MRM1 |
| AGMAT | PRSS21 |
| RAB27B | SLC7A11 |
| CAPG | FADS3 |
| SFI1 | PPP2R4 |
| CDIPT | RAB5C |
| FGF13 | ARID1A |
| AMIGO2 | MRC2 |
| POLE3 | C1D |
| TPM2 | FNDC3A |
| AP3D1 | PODXL |
| TAC1 | IPO4 |
| PTPMT1 | MARS2 |
| RAI14 | ADSS |
| NCAPG | SNX15 |
| GNA13 | PDE3A |
| HARS2 | TPM3 |
| KRT85 | P4HA2 |
| CDKAL1 | THEM4 |
| RFT1 | CALCOCO2 |
| CPNE8 | NARS |
| SPRYD4 | CDCP1 |
| PAFAH1B2 | DOCK7 |
| SLC9A3R2 | TNFRSF10A |
| CHAF1A | PRAF2 |
| DMD | METTL7A |
| DSP | TMEM41B |
| ANXA2 | CEP72 |
| MRPS33 | NXN |
| SLC12A4 | FAM96A |
| CBFB | CTSC |
| HIPK3 | TRAM1 |
| FERMT2 | RHOG |
| NAT6 | PPL |
| SMAD1 | SLC25A32 |
| PPP5C | FXR2 |
| GPAM | PTPLB |
| MRM1 | DNAJB1 |
| RAB5C | ATP6V1C1 |
| ARID1A | POLE4 |
| MRC2 | ATG9A |
| C1D | CLTC |
| FNDC3A | CSNK1D |
| IGF2R | ARL10 |
| TPM3 | TMED7 |
| P4HA2 | UBE4A |
| UTRN | ABCF2 |
| THEM4 | HARS |
| NARS | CDK5RAP1 |
| NXN | SLC30A1 |
| FAM96A | CLDN1 |
| LHX1 | GRPEL2 |
| TRAM1 | NUFIP2 |
| PPL | SLC25A1 |
| POLE4 | CHD1 |
| ATG9A | FRG1 |
| AURKB | MAT2A |
| PAX3 | PRIM1 |
| CSNK1D | RHEB |
| ARL10 | ATAD3B |
| TMED7 | RPP38 |
| DOCK5 | KCNN4 |
| UBE4A | GNL3L |
| ABCF2 | DOCK5 |
| DSPP | AP3B1 |
| HARS | TOMM34 |
| CDK5RAP1 | TMEM2 |
| SLC25A1 | ARL2 |
| RNMT | PPP2R5C |
| CHD1 | PICALM |
| FRG1 | RBM19 |
| MAT2A | SEC23A |
| PRIM1 | TICAM2 |
| RAD23B | POLD2 |
| PICALM | SLC25A22 |
| RBM19 | ATP6V1F |
| SEC23A | CYP51A1 |
| TRIM32 | DPP7 |
| TICAM2 | UBE2J1 |
| POLD2 | SRPRB |
| TXNRD1 | TXNRD1 |
| PHLDB2 | PHLDB2 |
| GFPT1 | GFPT1 |
| NCL | NCL |
| ATG3 | MRPS24 |
| LRRC8C | PEX11B |
| MTHFD2 | DUSP12 |
| CARHSP1 | GALNT1 |
| DUSP23 | MTRR |
| ITGB3 | CAND1 |
| CSRP1 | ATG3 |
| ANPEP | LRRC8C |
| TXNDC12 | MTHFD2 |
| PTPRF | CARHSP1 |
| SFXN1 | LAMC1 |
| PPIF | DUSP23 |
| RAB23 | SLC38A1 |
| UAP1 | SCAMP1 |
| TNFSF9 | AKAP8 |
| FSTL1 | F2 |
| DNAJB4 | HYAL1 |
| MBNL1 | ACP2 |
| TRMT1 | TMEM109 |
| RARS | CSRP1 |
| ADPGK | ANPEP |
| OAS1 | TXNDC12 |
| VTI1B | SFXN1 |
| PLXND1 | PPIF |
| ITGA2 | PHC2 |
| POLR2C | RAB23 |
| TYMS | LUZP1 |
| MPZL1 | DHX57 |
| ACVR1B | CPOX |
| CLN5 | GNAI2 |
| CHORDC1 | NPR3 |
| NEDD4 | UAP1 |
| CUL4B | TNFSF9 |
| SEC24A | DNAJB4 |
| CORO1C | MBNL1 |
| LCP1 | TRMT1 |
| MRPL20 | SPCS3 |
| DTD1 | RAB34 |
| TRIM27 | SPTLC1 |
| UBE2I | KPNA3 |
| CTNNB1 | ATRX |
| COMMD9 | PTPRF |
| GOLGA7 | SLC1A4 |
| PTPRJ | GFM1 |
| CDK5RAP3 | TMED2 |
| FADS2 | LPL |
| SLC25A13 | PPP3CA |
| SRPR | RARS |
| SNAP23 | ADPGK |
| PWP1 | OAS1 |
| ELMOD2 | VTI1B |
| PRPF40A | PLXND1 |
| SH3BGRL3 | ITGA2 |
| GAPDH | POLR2C |
| CEBPB | GEMIN7 |
| RCOR1 | TRIP13 |
| ITGB4 | AP2A1 |
| SLC38A2 | SLC25A24 |
| UGT8 | ANKFY1 |
| ZNF622 | SNX6 |
| MSI2 | MPZL1 |
| BRPF3 | CLN5 |
| NELL1 | CHORDC1 |
| GTPBP3 | NEDD4 |
| DNM1L | NRP1 |
| PGRMC1 | LYPLA2 |
| ARFIP2 | TMEM87A |
| ARHGDIA | MYO1E |
| PTGFRN | PRKCI |
| TMEM43 | CUL4B |
| SLC38A5 | KCTD11 |
| SHH | SEC24A |
| GSTM4 | TPPP |
| HSD17B12 | CORO1C |
| PTPRK | LCP1 |
| SHOC2 | MRPL20 |
| FHL5 | COIL |
| RCAN2 | MPDU1 |
| ARID2 | ARL5B |
| HSPA1A | IDH1 |
| STRN | PLK1 |
| NAPG | DTD1 |
| SEC23IP | COMMD9 |
| GAK | GOLGA7 |
| MLLT1 | CDK5RAP3 |
| TRMU | FADS2 |
| BCKDHB | SLC25A13 |
| ABHD11 | GALNT7 |
| ANP32B | CACNA2D1 |
| NOS1 | SLC16A3 |
| SYNE2 | PTRH1 |
| FAM105A | GPD2 |
| YIF1B | NT5E |
| IQGAP3 | WDFY1 |
| NOTCH2 | SRPR |
| NT5C3 (Mus musculus) | SNAP23 |
| GNPNAT1 | XIAP |
| LMNB2 | PWP1 |
| EHMT2 | ELMOD2 |
| LILRB4 | PRPF40A |
| POLA2 | CCL13 |
| FADS1 | UTP15 |
| PPP1R7 | PDLIM5 |
| SCYL1 | TMEM189 |
| DHX40 | LY6K |
| GPR56 | PTPRJ |
| MOSPD2 | SSNA1 |
| SAC3D1 | MLLT11 |
| PURA | PISD |
| AARSD1 | LRP1 |
| SLC4A7 | PPP3R1 |
| EIF4E | RBMS1 |
| HSPA1B | SH3BGRL3 |
| SERPINE2 | RCOR1 |
| TNFRSF10B | ITGB4 |
| TXN2 | SLC38A2 |
| RCN2 | EHMT1 |
| HSDL1 | VAMP3 |
| REG3A | ZNF384 |
| VPS39 | PTPLAD1 |
| AGRN | UGT8 |
| TMCO1 | ZNF622 |
| CA12 | MSI2 |
| ASH2L | BRPF3 |
| XIAP | TPM1 |
| DNAJC19 | SQSTM1 |
| CHMP2A | FMNL2 |
| SNX15 | MYO10 |
| PDE3A | PKN2 |
| CDCP1 | FNDC3B |
| DOCK7 | GTPBP3 |
| PRSS21 | NKX2-3 |
| SLC7A11 | ARFIP2 |
| FADS3 | ARHGDIA |
| IPO4 | RAB6A |
| MARS2 | UNC93B1 |
| GRIA2 | TMEM59 |
| ADSS | BET1 |
| SLC25A32 | IFRD1 |
| FXR2 | ATP6V0A1 |
| CALCOCO2 | PTGFRN |
| PTPLB | TMEM43 |
| DNAJB1 | SLC38A5 |
| ATP6V1C1 | PAX6 |
| TNFRSF10A | NOS1 |
| PRAF2 | RDH10 |
| METTL7A | GSTM4 |
| TMEM41B | HSD17B12 |
| CEP72 | PTPRK |
| RHEB | SHOC2 |
| ATAD3B | FHL5 |
| RPP38 | RCAN2 |
| KCNN4 | ARID2 |
| GNL3L | HSPA1A |
| SLC30A1 | PGM1 |
| CLDN1 | PXDN |
| TPPP | RQCD1 |
| GRPEL2 | KDELC2 |
| ATP2A2 | TTC9C |
| DPP7 | NUCB1 |
| UBE2J1 | STRN |
| MRPS24 | NAPG |
| AP3B1 | SEC23IP |
| TOMM34 | GAK |
| TMEM2 | MLLT1 |
| PPP2R5C | TRMU |
| NUFIP2 | SDCBP |
| SLC25A22 | STX7 |
| ATP6V1F | TMED3 |
| CYP51A1 | RHOB |
| SRPRB | PPIB |
| SLC38A1 | LHX1 |
| SCAMP1 | REG3A |
| AKAP8 | ACVR1 |
| F2 | MYBL2 |
| HYAL1 | CD226 |
| ACP2 | PGR |
| PEX11B | BMPR2 |
| MYLIP | ACOT7 |
| DUSP12 | PTCRA |
| MTRR | BAZ2A |
| SPCS3 | THAP2 |
| RAB34 | IGFBP3 |
| SPTLC1 | FGFR3 |
| KPNA3 | CDH3 |
| ATRX | MUC16 |
| LUZP1 | USP25 |
| DHX57 | IKBKE |
| CPOX | LMO2 |
| NPR3 | JARID2 |
| MDM2 | TMCC3 |
| AMACR | NFIB |
| NFKBIA | SCARB1 |
| HOXB5 | HIST1H4H |
| MMP14 | ELAVL1 |
| TEK | APAF1 |
| CSF1R | HIST1H4D |
| MEOX2 | KIF14 |
| GRM7 | CYP1A1 |
| RNF123 | ZNF281 |
| ADM | PCDHB10 |
| PDGFD | BMF |
| ENO2 | HOMER2 |
| ITGA3 | ETV6 |
| MDH2 | RCHY1 |
| ERG | FAM19A1 |
| PDZD2 | CYP24A1 |
| THRB | ARRDC3 |
| ITGAV | MSR1 |
| OPRM1 | HIST1H4B |
| TMPRSS2 | MAP2K7 |
| CCNG1 | FATE1 |
| PNPT1 | H3F3B |
| BMF | HIST1H4F |
| AXIN2 | BMPR1B |
| PANK1 | DIO3 |
| RAB3GAP1 | RGS17 |
| PLAG1 | ADAMTS1 |
| KNTC1 | HIST1H4E |
| PDCD10 | IL3 |
| ZCCHC11 | SAMSN1 |
| RBL2 | MAN1A1 |
| CASP8AP2 | IRF4 |
| RXRA | EIF3M |
| GTF2IRD1 | HIST1H4C |
| ALAS1 | GFAP |
| CYP2C19 | CDX2 |
| CYP2C8 | LRP1B |
| CYP2C9 | CBX7 |
| PDK4 | KRT7 |
| MMP13 | CTNNA3 |
| PRB1 | PERP |
| EPHA3 | RBM8A |
| RAB22A | TIAL1 |
| SAMD3 | HIST1H4L |
| BEST1 | PIGR |
| IL10 | ID2 |
| BRMS1 | PTPRD |
| SEC14L2 | TNFSF4 |
| GYPE | ST18 |
| CD68 | GYPC |
| CD79A | SMARCD1 |
| PTCH1 | RECK |
| SMAD7 | TSPAN8 |
| MMP16 | CASP6 |
| PLAU | UBE2N |
| NDUFA2 | CEBPG |
| IL8 | STK10 |
| IRAK4 | SLAMF1 |
| TCEAL1 | FGFR2 |
| TRAF6 | ID3 |
| COL2A1 | NKIRAS2 |
| ASPRV1 | DHDDS |
| CREBBP | IDH3A |
| SLC7A5 | CASP7 |
| POLR1A | USP7 |
| IL17A | MAP3K7 |
| TIMM8A | ISL1 |
| NEDD9 | UGT2B17 |
| FOSL2 | B3GALT4 |
| CYCS | BLID |
| CCL5 | NR2F1 |
| UNC5C | WDR5 |
| DIAPH2 | IL1RN |
| MYL9 | EIF4EBP1 |
| HSH2D | PELO |
| DCC | SIDT1 |
| RPS19 | HIST1H4A |
| GSTP1 | SGPL1 |
| RTN1 | CBLN2 |
| DUSP2 | TRPC6 |
| DNMT3B | PALM2 |
| COL4A1 | JTB |
| CD276 | HSD11B1 |
| MCL1 | SUV39H1 |
| PRKCA | IRX1 |
| ATN1 | PIGF |
| COL5A3 | FHIT |
| CRYGC | FERMT1 |
| COL4A2 | TEF |
| SMURF2 | APRT |
| KIDINS220 | CHPT1 |
| TCL1A | CARS |
| SPEN | PDZRN3 |
| CTNNBIP1 | ZFP64 |
| HSPB6 | POMC |
| ELN | BOC |
| COL15A1 | FCER2 |
| SPARC | FSCN1 |
| FH | MMP3 |
| GATA4 | CRLS1 |
| NCKIPSD | RPS6KB1 |
| PLXNA3 | CD2AP |
| ONECUT2 | BIRC2 |
| TGFB3 | BLM |
| TCF4 | DFFA |
| CCND2 | CLEC4G |
| TFPI2 | BNIP3 |
| HOXA10 | SMPD1 |
| ADIPOQ | FLI1 |
| IL1RAP | KIR3DL1 |
| DNAH8 | DFFB |
| HDAC2 | TSC2 |
| MEIS1 | CLINT1 |
| HDAC4 | H2AFX |
| FGA | ADAM17 |
| YY1 | MYO6 |
| ACSF3 | SWAP70 |
| TET1 | SMTN |
| ATP1B1 | RHOF |
| PMP22 | YES1 |
| NANOS2 | MAPK7 |
| PPARA | TAGLN |
| PRB3 | RREB1 |
| FGB | VANGL1 |
| CSHL1 | CDH11 |
| ABCD1 | RTKN |
| TGFB2 | HOXA9 |
| PXMP2 | SOX18 |
| PAX7 | SOX4 |
| FZD5 | UTP14A |
| IL4 | F11R |
| IGFBP1 | IRS2 |
| FHIT | BEX1 |
| LRRFIP1 | CFTR |
| ADAM12 | MYO5A |
| WWOX | UTP14C |
| APRT | SOD2 |
| SPRY1 | ERCC8 |
| E2F7 | PGK1 |
| PRKAA1 | RAB27A |
| MT4 | NKX2-5 |
| PPM1D | CCKBR |
| PPP1R13B | TGIF2 |
| MAP1LC3A | TLR3 |
| ABL1 | ERG |
| SNAI1 | RASGRP1 |
| BECN1 | EPAS1 |
| DPYD | HLA-G |
| CTLA4 | RBL2 |
| PARP8 | BAALC |
| ACOT7 | HTR2C |
| FBXW11 | MLC1 |
| ICOS | CD70 |
| TMEM184B | RGS2 |
| SMC1A | NOV |
| KLF6 | S100B |
| GPD1 | TRIM68 |
| STK24 | IMPDH1 |
| PGK1 | TNFRSF4 |
| TMOD3 | ADAM12 |
| CCNT2 | HSPB6 |
| LAMC2 | SPEN |
| TPPP3 | FZD5 |
| DMTF1 | IL4 |
| BMP7 | E2F7 |
| STK3 | TET1 |
| FGF23 | STC1 |
| GDF15 | PRB3 |
| ACTG1 | FASLG |
| ARL2BP | TFPI2 |
| TBX1 | GSTP1 |
| IL2RA | HSPG2 |
| IL7R | IGFBP1 |
| IRF9 | SPARC |
| IRF1 | NAV3 |
| TRIM63 | GLUL |
| GADD45A | DMPK |
| EPB41L3 | ARHGEF1 |
| LTBP1 | SMURF2 |
| AR | CD276 |
| NCALD | KIDINS220 |
| JARID2 | WWOX |
| CACNA2D2 | PPM1D |
| PMAIP1 | NPPA |
| PRKACA | PMP22 |
| ATXN1 | FGA |
| SALL4 | ELN |
| STC1 | NCKIPSD |
| ACVR1 | PXMP2 |
| CD226 | FGB |
| CD40 | RTN1 |
| HMBOX1 | PLXNA3 |
| GLIS2 | LRRFIP1 |
| BID | ADAMTS13 |
| BMPER | IL1RAP |
| BRD2 | SRY |
| MARK2 | ATP1B1 |
| ESR2 | FBN1 |
| MATR3 | DLG4 |
| XRN1 | COL15A1 |
| MYH6 | CSHL1 |
| MYCBP | ONECUT2 |
| RERE | PCK2 |
| NELL2 | PLK2 |
| MCM7 | C1QTNF6 |
| OGN | HIVEP1 |
| MAX | ZFP36L1 |
| CCL28 | BMP1 |
| VDR | CDKN2B |
| TIAM1 | GRN |
| CDC25C | ZFP36L2 |
| VKORC1 | CDH15 |
| NFAT5 | PHB2 |
| HSPG2 | AHR |
| FZD7 | COL5A2 |
| PSMA7 | MYBBP1A |
| CCR7 | LIMK1 |
| SLC9A3R1 | PUM2 |
| CD40LG | SIRT2 |
| PDPN | NR5A2 |
| RCHY1 | MSH3 |
| LIFR | MEF2D |
| CD80 | CA1 |
| ECD | NTRK3 |
| FOXP3 | SIX1 |
| SCARB1 | GYPA |
| CD86 | CD36 |
| DLG4 | ACSF3 |
| ELAVL1 | ABCD1 |
| UBE2N | LBX1 |
| MAP2K7 | BRD8 |
| CD2 | IDUA |
| RHOU | CAPNS1 |
| USP7 | ADAM9 |
| SOX17 | TSPAN3 |
| ETV4 | EFEMP2 |
| TCF7L2 | NR2E1 |
| KLF13 | TRPM3 |
| TLE1 | FGF8 |
| SDC2 | COL9A1 |
| WNT10A | GSX2 |
| SAGE1 | FABP7 |
| PDGFC | ELAVL2 |
| WDR6 | DCX |
| DALRD3 | HGFAC |
| ARNTL | FOXG1 |
| PER1 | SYK |
| CLOCK | C1orf61 |
| NDST1 | MEIS2 |
| FBP1 | NF2 |
| ANXA1 | NEUROD1 |
| CGA | IGFALS |
| NOTCH3 | CXCL10 |
| CPAMD8 | PROS1 |
| SAT1 | TSHZ3 |
| SIM2 | LDOC1 |
| TNFRSF1A | SMAD5 |
| MKI67 | AGTR1 |
| ETV1 | INPP5D |
| TNNT2 | LILRB4 |
| LIN28B | IL13RA1 |
| E2F2 | DET1 |
| EIF4G1 | TRIM69 |
| TRAT1 | AICDA |
| IL13 | IRAK3 |
| TUT1 | PROC |
| SLC25A10 | ZIC3 |
| RDX | PKIA |
| GAB2 | TM6SF1 |
| KHSRP | CDKN1C |
| DYRK2 | HES4 |
| FOXP1 | BACH1 |
| TNRC6C | PRKAR1A |
| TRIM17 | HCN2 |
| CYFIP2 | VSX1 |
| TCF19 | SLC35B2 |
| COPS8 | CCL18 |
| EPHA2 | HHAT |
| CBS | SAA4 |
| CYLD | PSMB9 |
| AIRE | IRF7 |
| KISS1R | TGFBI |
| CDC34 | KCNH1 |
| CNTN3 | PHF17 |
| PGC | RNF123 |
| NKX2-1 | NCAPG2 |
| CSN1S1 | DUSP1 |
| NOL3 | BMP6 |
| CLEC5A | IMPACT |
| BASP1 | CD209 |
| TREH | USP9X |
| LSM1 | ALK |
| NCOA4 | ALDH7A1 |
| PDCD6 | PDC |
| EIF3A | TRIM63 |
| PRTN3 | CACNA1C |
| TBX5 | EBP |
| SPRY2 | FXN |
| PDHA2 | MT4 |
| IFNA2 | MATR3 |
| SKP1 | BMP7 |
| RBPJL | PDE5A |
| ZNF362 | CCL22 |
| TM9SF3 | TMPRSS11D |
| XPO1 | VCAM1 |
| CSAD | GAA |
| ZNF512B | MSH6 |
| TAF1C | PIK3R1 |
| CYP27B1 | IL2RG |
| HAND1 | RHOA |
| HK1 | CCL4 |
| BIRC3 | PTP4A3 |
| ALDH1A1 | SCARA3 |
| RABEPK | WNT2 |
| LAMP3 | MLH1 |
| CCL7 | TP53INP1 |
| GHR | HOXC4 |
| FOXA1 | RALBP1 |
| RORA | EFNB2 |
| FXR1 | CA9 |
| NCOA3 | STAT5A |
| SYCP3 | GPAA1 |
| PSD4 | MSH2 |
| HAND2 | ZNF652 |
| UHRF2 | MME |
| CD46 | DIRC2 |
| WFS1 | SOS1 |
| IFI44 | GPM6B |
| CCR4 | ADAM11 |
| HNRNPA1 | TCF4 |
| MMP8 | OASL |
| DCN | IRF1 |
| SOCS3 | HIVEP2 |
| DDX4 | JAK1 |
| SRM | HYLS1 |
| ZCCHC6 | GABRA1 |
| DERL1 | PAM |
| FN1 | FGF7 |
| PPARGC1A | NFATC1 |
| DDX6 | CXCR3 |
| PEBP1 | MT2A |
| PPP2R1B | CYR61 |
| WIBG |  |
| NES |  |
| NCBP1 |  |
| GRB2 |  |
| AKT2 |  |
| SOCS4 |  |
| CCNA2 |  |
| FUS |  |
| SARS2 |  |
| MEF2C |  |
| LRP1B |  |
| RASGRP3 |  |
| PTPRD |  |
| GPR160 |  |
| DHDDS |  |
| XAF1 |  |
| PRKCH |  |
| USP25 |  |
| RELB |  |
| SAMSN1 |  |
| SMARCD1 |  |
| PDCD2 |  |
| DUSP16 |  |
| EP300 |  |
| ALK |  |
| VAMP2 |  |
| CSN2 |  |
| G6PD |  |
| DUSP1 |  |
| ASPN |  |
| PTGES3 |  |
| UBL4A |  |
| ATP5B |  |
| CSN3 |  |
| DLL1 |  |
| USF2 |  |
| HSP90AA1 |  |
| APP |  |
| HOXA5 |  |
| RC3H1 |  |
| TERF2IP |  |
| POU4F2 |  |
| RAP1GAP |  |
| MAGI2 |  |
| RC3H2 |  |
| CACNA1C |  |
| DCP1A |  |
| CACNB1 |  |
| TIA1 |  |
| IL17D |  |
| PLEKHM1 |  |
| RPS6KA5 |  |
| HBEGF |  |
| FIGLA |  |
| ANKS1B |  |
| NOV |  |
| KCNJ2 |  |
| TJP1 |  |
| MYD88 |  |
| REM1 |  |
| RPS6KB1 |  |
| SLC25A5 |  |
| UBASH3B |  |
| ABCB1 |  |
| TLR5 |  |
| SERTAD2 |  |
| MDGA1 |  |
| PTPN1 |  |
| NPTX1 |  |
| MMP7 |  |
| MNT |  |
| ISCU |  |
| MIB1 |  |
| DUSP9 |  |
| AIFM3 |  |
| ATP11C |  |
| EPAS1 |  |
| UBQLN1 |  |
| VWF |  |
| CTRL |  |
| MID1IP1 |  |
| CLASP2 |  |
| COX4I1 |  |
| HOXA3 |  |
| CA9 |  |
| GPD1L |  |
| KRT12 |  |
| MIA3 |  |
| DIRC2 |  |
| RAD52 |  |
| PIM1 |  |
| ACVRL1 |  |
| SMCHD1 |  |
| PNLIP |  |
| PTAR1 |  |
| NR1H2 |  |
| FGFRL1 |  |
| ABCB9 |  |
| HIF3A |  |
| TNPO1 |  |
| LONP1 |  |
| CHD9 |  |
| BNIP3 |  |
| TP53I11 |  |
| LARP6 |  |
| SEH1L |  |
| DDAH1 |  |
| COX4I2 |  |
| OSR1 |  |
| P4HB |  |
| CDK10 |  |
| STS |  |
| HECTD1 |  |
| EFNA3 |  |
| HSD17B1 |  |
| DCT |  |
| CPEB2 |  |
| NIPBL |  |
| ITLN1 |  |
| COX10 |  |
| KIAA1161 |  |
| PNCK |  |
| HACE1 |  |
| PPARD |  |
| HDHD2 |  |
| C4orf27 |  |
| ATP6V0A4 |  |
| RAD51C |  |
| CREBL2 |  |
| PKHD1 |  |
| HSDL2 |  |
| VPS36 |  |
| CCNB1 |  |
| HERC6 |  |
| PRKCSH |  |
| SLC35B2 |  |
| CCDC111 |  |
| ZNF559 |  |
| ABCB4 |  |
| C17orf80 |  |
| RNASEL |  |
| ATP7B |  |
| HLA-A |  |
| GOLPH3L |  |
| FZR1 |  |
| ILK |  |
| CD5 |  |
| C2orf43 |  |
| NT5DC1 |  |
| SKAP2 |  |
| GDE1 |  |
| TSPO |  |
| ASXL2 |  |
| PDCD6IP |  |
| OSGEPL1 |  |
| CARD8 |  |
| GOLGA5 |  |
| DNTT |  |
| MSH2 |  |
| RHOT1 |  |
| USP9X |  |
| RAB21 |  |
| DHRS12 |  |
| SLC35B3 |  |
| SLC35A1 |  |
| HSP90B1 |  |
| CADM1 |  |
| PHKB |  |
| PAK7 |  |
| CENPJ |  |
| VPS45 |  |
| UGDH |  |
| PMS1 |  |
| EDNRA |  |
| ECHDC1 |  |
| PWWP2A |  |
| RAPH1 |  |
| UGP2 |  |
| TSG101 |  |
| PNN |  |
| EDNRB |  |
| PLSCR4 |  |
| OMA1 |  |
| WIPF1 |  |
| TBC1D9 |  |
| GTF2H1 |  |
| CD63 |  |
| TP53INP1 |  |
| PAX5 |  |
| NR4A2 |  |
| CRIM1 |  |
| ZFPM2 |  |
| NFE2 |  |
| NBEA |  |
| SREBF1 |  |
| FBN2 |  |
| ETV6 |  |
| CFTR |  |
| POLD1 |  |
| ADAM8 |  |
| PTTG1 |  |
| FGFR2 |  |
| CDC20 |  |
| HRAS |  |
| MYBBP1A |  |
| BAMBI |  |
| PML |  |
| ALDH1A3 |  |
| NLK |  |
| SMAD4 |  |
| HOXA11 |  |
| IL2 |  |
| BAALC |  |
| FASTK |  |
| CTTNBP2 |  |
| HSPA5 |  |
| CD9 |  |
| CCL27 |  |
| CDX2 |  |
| RGS4 |  |
| NOTCH4 |  |
| TGFBI |  |
| ZFPM1 |  |
| GATA6 |  |
| LYN |  |
| TRIM41 |  |
| SOX13 |  |
| MUC1 |  |
| SEMA4F |  |
| BTBD3 |  |
| RPUSD2 |  |
| SLC25A4 |  |
| PLEKHG6 |  |
| CPEB4 |  |
| TAF12 |  |
| TRPC3 |  |
| PTP4A1 |  |
| DDIT3 |  |
| PPFIBP2 |  |
| RPE |  |
| UCN3 |  |
| GSK3B |  |
| ACTA1 |  |
| POU1F1 |  |
| CHFR |  |
| GLI3 |  |
| TBX3 |  |
| FAF1 |  |
| MICB |  |
| SP7 |  |
| ZNFX1 |  |
| HDAC5 |  |
| CCL1 |  |
| GPR137B |  |
| YES1 |  |
| GABPA |  |
| MGST1 |  |
| KLRK1 |  |
| NFE2L2 |  |
| BTG4 |  |
| ING2 |  |
| WNT1 |  |
| CDC20B |  |
| NUMA1 |  |
| ACTB |  |
| WNT3 |  |
| ABCG4 |  |
| STK39 |  |
| VPS4A |  |
| TMSB10 |  |
| MDC1 |  |
| TGIF2 |  |
| PARK7 |  |
| CREM |  |
| ACSL1 |  |
| PARK2 |  |
| NCOA2 |  |
| LRP6 |  |
| CYP8B1 |  |
| HSPA9 |  |
| MAPKAPK2 |  |
| PHB2 |  |
| GRIN1 |  |
| CAMK2G |  |
| ID3 |  |
| RFX4 |  |
| IGFALS |  |
| TRIM67 |  |
| HES5 |  |
| DAB2IP |  |
| MAP1A |  |
| EIF4E3 |  |
| SOX10 |  |
| NOVA1 |  |
| NAV3 |  |
| AATK |  |
| UBE2Q1 |  |
| SYF2 |  |
| ATG5 |  |
| ITGAX |  |
| CASP4 |  |
| LMX1A |  |
| YAP1 |  |
| ASCL1 |  |
| TRIM13 |  |
| ZNF501 |  |
| CD3EAP |  |
| CARS |  |
| TGFBRAP1 |  |
| PITX3 |  |
| CFD |  |
| LYZ |  |
| POU3F2 |  |
| STIP1 |  |
| HIPK2 |  |
| SEMA4C |  |
| RALA |  |
| F8 |  |
| SHANK2 |  |
| AICDA |  |
| SHANK3 |  |
| HEY1 |  |
| NRXN1 |  |
| NLGN3 |  |
| NUDT6 |  |
| SCARA3 |  |
| NLGN4X |  |
| HOXC4 |  |
| CCL8 |  |
| TRIM21 |  |
| CDA |  |
| MAP3K10 |  |
| PTCHD1 |  |
| BCL7A |  |
| PRKAR1A |  |
| KRT5 |  |
| WISP2 |  |
| MPL |  |
| TSPAN3 |  |
| EFEMP2 |  |
| CAPNS1 |  |
| RAB14 |  |
| CDKN2C |  |
| TPT1 |  |
| CAB39L |  |
| CUL5 |  |
| BMPR2 |  |
| IL6R |  |
| CYP19A1 |  |
| ITGA6 |  |
| IL6ST |  |
| SIDT1 |  |
| PLP1 |  |
| BMP4 |  |
| BNIP2 |  |
| RAG2 |  |
| MEF2D |  |
| ULK1 |  |
| SH2B2 |  |
| BSG |  |
| ISL1 |  |
| FGF7 |  |
| SP3 |  |
| UVRAG |  |
| ITGA5 |  |
| LTF |  |
| TSHZ3 |  |
| SFTPC |  |
| SPIB |  |
| ZIC3 |  |
| TBCE |  |
| TYRP1 |  |
| PRKD2 |  |
| CNP |  |
| BICC1 |  |
| PKD1 |  |
| EPO |  |
| RND3 |  |
| S100A8 |  |
| PTPRU |  |
| PKD2 |  |
| TLX3 |  |
| NPAS3 |  |
| S100A9 |  |
| AURKA |  |
| TLX1 |  |
| ATF2 |  |
| PTPRO |  |
| ADAMDEC1 |  |
| CYBB |  |
| RASSF2 |  |
| TXNRD2 |  |
| PADI4 |  |
| HOXA4 |  |
| TXNIP |  |
| GPX2 |  |
| SELE |  |
| TNFAIP3 |  |
| EDC4 |  |
| MXI1 |  |
| HCN2 |  |
| CD47 |  |
| LCN2 |  |
| PIWIL4 |  |
| ENPP6 |  |
| ELOVL7 |  |
| MTAP |  |
| IL12A |  |
| SESN1 |  |
| INPP5D |  |
| ILF2 |  |
| SOCS5 |  |
| STRAP |  |
| MAP4K1 |  |
| FMOD |  |
| SOX5 |  |
| ILF3 |  |
| PPP2CA |  |
| SRFBP1 |  |
| PRMT5 |  |
| MARCKS |  |
| PTGER4 |  |
| APAF1 |  |
| EIF4A2 |  |
| SERPINI1 |  |
| LRPPRC |  |
| RASGRP1 |  |
| TRPV1 |  |
| NQO1 |  |
| CA3 |  |
| PPT1 |  |
| AKR1C2 |  |
| APCS |  |
| CXCL3 |  |
| KRT15 |  |
| ADI1 |  |
| IL17C |  |
| IL23A |  |
| NUDT19 |  |
| RSL1D1 |  |
| MIA |  |
| SMARCA4 |  |
| MUC4 |  |
| KIF1B |  |
| TSPYL2 |  |
| SRGN |  |
| MELK |  |
| ADRB2 |  |
| ANP32A |  |
| IL11 |  |
| NDUFA13 |  |
| NHS |  |
| TGIF1 |  |
| CRTC1 |  |
| PELI1 |  |
| HIC2 |  |
| CCL20 |  |
| FABP7 |  |
| TOP2B |  |
| HLA-DRA |  |
| OLIG2 |  |
| TFB1M |  |
| PRKCE |  |
| IRF4 |  |
| RP2 |  |
| LPA |  |
| PTCRA |  |
| TNMD |  |
| GFI1 |  |
| TFB2M |  |
| IARS |  |
| SLC16A10 |  |
| DLL3 |  |
| THY1 |  |
| AKT1S1 |  |
| NODAL |  |
| SAMD4A |  |
| IL17F |  |
| BMPR1A |  |
| TFAM |  |
| DDT |  |
| BCAN |  |
| VEGFC |  |
| BMPR1B |  |
| SPAG11B |  |
| SGK3 |  |
| MARCKSL1 |  |
| CHKA |  |
| STAT6 |  |
| PRRG4 |  |
| UBB |  |
| ENPP3 |  |
| TLR3 |  |
| FZD6 |  |
| WNK1 |  |
| PYCARD |  |
| ALB |  |
| IL22 |  |
| TLR1 |  |
| PTGS1 |  |
| BMP5 |  |
| C5AR1 |  |
| ALG2 |  |
| IRF6 |  |
| MMP11 |  |
| MAP3K8 |  |
| AGTR2 |  |
| DNAJB7 |  |
| RYBP |  |
| CSF3 |  |
| ST14 |  |
| ZBTB10 |  |
| C9orf3 |  |
| CYP3A4 |  |
| G6PC |  |
| ADAMTS5 |  |
| PRAP1 |  |
| ITCH |  |
| GTF2A1L |  |
| HEXIM1 |  |
| LOC283871 |  |
| HEXIM2 |  |
| APOBEC3G |  |
| IL5 |  |
| MAP2K4 |  |
| CD1A |  |
| SCAP |  |
| MVD |  |
| SELL |  |
| MMAB |  |
| CAPRIN1 |  |
| RPS6KB2 |  |
| GH2 |  |
| PSMD13 |  |
| SEPN1 |  |
| PRODH |  |
| SDHB |  |
| DDX58 |  |
| TERT |  |
| SDHC |  |
| LDLR |  |
| TXNL1 |  |
| LRP5 |  |
| NPHS2 |  |
| PRDX3 |  |
| VLDLR |  |
| GLRX3 |  |
| NR4A3 |  |
| EPS15 |  |
| GPX3 |  |
| MAPKAPK5 |  |
| EIF5 |  |
| CYP11A1 |  |
| F3 |  |
| SLC22A3 |  |
| WNT2 |  |
| LIN9 |  |
| POLR3D |  |
| AQP1 |  |
| AQP4 |  |
| CPT1A |  |
| ABCA1 |  |
| ABCG1 |  |
| SREBF2 |  |
| SIRT6 |  |
| CROT |  |
| UHRF1BP1 |  |
| NKIRAS2 |  |
| CYB5R3 |  |
| RAB40C |  |
| WWP2 |  |
| OVGP1 |  |
| LBR |  |
| DNPEP |  |
| CDKN2B |  |
| UCP2 |  |
| NME1 |  |
| TOP1 |  |
| HN1L |  |
| FURIN |  |
| XYLT2 |  |
| FEN1 |  |
| SCN5A |  |
| MYOG |  |
| FADD |  |
| APPBP2 |  |
| TAS2R13 |  |
| AIFM1 |  |
| SNF8 |  |
| SLITRK1 |  |
| TRIB3 |  |
| ANXA6 |  |
| DCXR |  |
| METAP2 |  |
| DES |  |
| MAP2 |  |
| CDK8 |  |
| MAP3K2 |  |
| CD274 |  |
| NT5C1A |  |
| IFIT3 |  |
| DDIT4L |  |
| MX1 |  |
| ST8SIA4 |  |
| ADARB1 |  |
| CXCL9 |  |
| HERC5 |  |
| BANK1 |  |
| CD84 |  |
| CXCL11 |  |
| LMO2 |  |
| ELOVL6 |  |
| ENTPD1 |  |
| TNFRSF4 |  |
| FMNL1 |  |
| ADCY9 |  |
| EXTL3 |  |
| COTL1 |  |
| SLC39A5 |  |
| SLC15A1 |  |
| AKAP12 |  |
| CSNK2A1 |  |
| P2RX7 |  |
| NLRP3 |  |
| SLC2A4 |  |
| HMGB1 |  |
| TCF3 |  |
| NKRF |  |
| LBX1 |  |
| FANCG |  |
| NFATC3 |  |
| RING1 |  |
| FBXO32 |  |
| EWSR1 |  |
| LRPAP1 |  |
